# Electroreductive room-temperature C–H activations with RuCl_3_·*n*H_2_O precatalyst *via* cathodic ruthenium(iii/ii) manifold†

**DOI:** 10.1039/d5sc02780b

**Published:** 2025-06-27

**Authors:** Takuya Michiyuki, Tristan von Münchow, Zhipeng Lin, Binbin Yuan, João C. A. Oliveira, Lutz Ackermann

**Affiliations:** a Wöhler Research Institute for Sustainable Chemistry, Georg-August-Universität Göttingen Tammannstraße 2 37077 Göttingen Germany Lutz.Ackermann@chemie.uni-goettingen.de; b DZHK (German Centre for Cardiovascular Research) Potsdamer Straße 58 10875 Berlin Germany

## Abstract

We, herein, disclose a strategy to directly utilize user-friendly RuCl_3_·*n*H_2_O for *ortho*- as well as *meta*-C–H functionalizations at low temperatures. The key to success was the *in situ* formation of the active ruthenium catalyst through cathodic electron transfer, setting the stage for C–H activations under exceedingly mild reaction conditions. The robustness of our electrocatalysis process was highlighted by the late-stage diversification of compounds of relevance to chemical, agrochemical, and pharmaceutical industries, as well as simple amines as terminal reductants for the electroreduction. Detailed mechanistic studies by, among others, spectroelectrochemical analysis provided strong evidence for a cathodic reduction manifold.

## Abstract 2

Electroreductive C–H activations with RuCl_3_·*n*H_2_O precatalyst proceed at exceedingly mild temperatures. Detailed mechanistic studies offer evidence for a cathodic ruthenium(iii/ii) manifold, along with the involvement of a biscyclometalated species.

## Graphical abstract

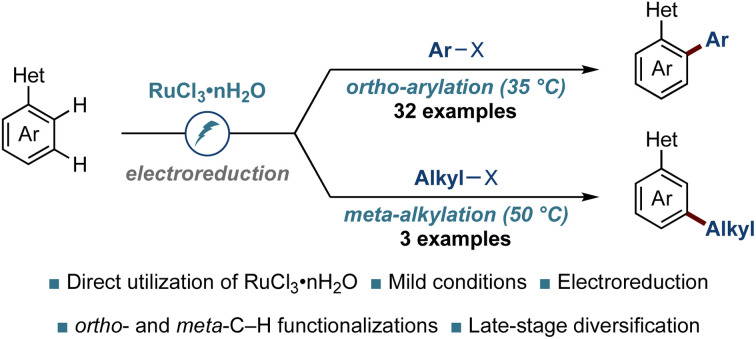

## Introduction

Ruthenium-catalyzed C–H activation chemistry has evolved into an indispensable chemical toolbox for selectively functionalizing arene scaffolds.^1–10^ For instance, site-selectivity can be precisely modulated^11–17^ without exploiting elaborate auxiliaries, providing instant access to *ortho*-^18–27^ or *meta*-decorated frameworks.^28–41^ Our group indeed discovered that the addition of phosphine ligands can alter the site-selectivity in C–H benzylations from *ortho*- to unusual *meta*-selectivities.^14^ Precise selectivity control can also be achieved depending on the nature of the electrophilic substrates.^13,16^ Among such transformations, C–H arylations hold a significant potential for the assembly of valuable biaryl scaffolds,^6,9,10^ with carboxylate-assisted ruthenium(ii/iv)-catalyzed C–H activations representing the most robust and efficient strategy.^23^ In recent years, substantial efforts have been made to render reaction conditions milder. Notable advancements include the use of light irradiation, as disclosed by Ackermann and Greaney,^42–45^ as well as the employment of arene ligand-free ruthenium precatalysts.^46,47^

**Scheme 1:**
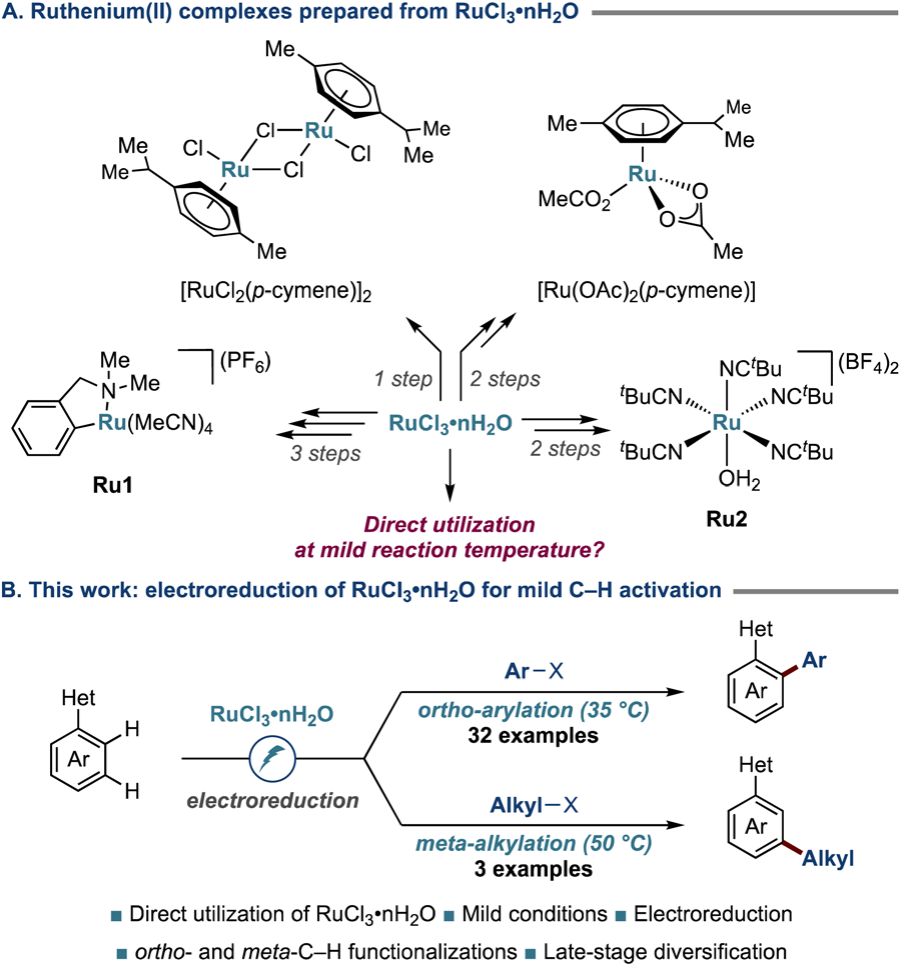
(A) Ruthenium(ii) complexes prepared from RuCl_3_·*n*H_2_O. (B) This study. Het: heterocycle.

Despite the considerable progress,^48–52^ the majority of existing approaches heavily depend on ruthenium(ii) precatalysts, which require lengthy organometallic syntheses by experts with glovebox techniques. This reliance jeopardizes the resource efficiency and user-friendly nature of the overall processes. Importantly, when tracing back the synthesis routes, one can realize that ruthenium(ii) complexes, such as [RuCl_2_(*p*-cymene)]_2_, [Ru(OAc)_2_(*p*-cymene)], arene ligand-free complexes Ru1 and Ru2, originate from RuCl_3_·*n*H_2_O (Scheme 1A).^46,47,53^ Therefore, the direct employment of RuCl_3_·*n*H_2_O as the catalyst offers a more sustainable and user-friendly strategy for executing C–H activation. In 2007, our group first disclosed the use of arene ligand-free RuCl_3_·*n*H_2_O for *ortho*-C–H arylation.^24^ Subsequent studies also demonstrated that RuCl_3_·*n*H_2_O can be utilized for *ortho*-^54–62^ as well as *meta*-C–H functionalizations.^63–65^ Nevertheless, rather harsh conditions with reaction temperatures from 100–140 °C are generally required, representing a considerable limitation.

To directly unlock the inherent reactivity of ruthenium catalysis using RuCl_3_·*n*H_2_O, we hypothesized whether, upon the electroreduction of a ruthenium(iii) species, an active ruthenium(ii) intermediate could be formed,^54,57,59^ setting the stage for economically-attractive, yet user-friendly C–H activation. As a result of our studies, we, herein, disclose the direct use of RuCl_3_·*n*H_2_O for C–H activations at mild reaction temperatures, enabling *ortho*-C–H arylations and *meta*-C–H alkylations across a wide array of substrates, including challenging pharmaceutical, agrochemical, and naturally-occurring molecules (Scheme 1B). Thus, we harness electricity as a sustainable reductant, eliminating the need for potentially hazardous chemical redox agents.^66–69^ A notable feature of our findings is that both a sacrificial anode or a simple amine could be employed as the terminal oxidant. Detailed spectroelectrochemical studies uncovered the key reduction of the ruthenium(iii) precatalyst.

## Results and discussion

### Reaction optimization

To probe our original hypothesis, we performed the C–H arylation under electroreductive conditions using transformable oxazoline 1a and aryl bromide 2a as the model substrates in the presence of RuCl_3_·*n*H_2_O as the bench-stable and commercially available precatalyst (Table 1). We thus employed NaOAc for a base-assisted internal electrophilic substitution (BIES) C–H activation manifold^7,10^ with K_2_CO_3_ as the stoichiometric base. We probed dipolar DMA as the solvent to ensure conductivity, along with a zinc plate as the sacrificial anode. Gratifyingly, we indeed obtained the desired arylation product 3 in 85% yield after electrolysis using a non-precious zinc plate anode and a nickel foam cathode (*j* = 2.5 mA cm^−2^, 1.8 F mol^−1^, entries 1 and 2). We were delighted to observe that the reaction proceeded at 35 °C, significantly lower than the temperatures required in previous studies using RuCl_3_·*n*H_2_O.^24,54–62^*N*-Methyl-2-pyrrolidone (NMP) as the reaction medium led to a lower yield (entry 3). Other leaving groups of aryl halides, such as chloro and iodo groups, proved viable, with the bromo group being superior in the ruthenium electrocatalysis (entry 4). Thus far, chloroarenes gave inferior results, likely due to their higher bond dissociation energies (BDE).^24,62^ A control experiment without electricity highlighted the indispensable role of the electroreduction (entry 5). Likewise, no product formation or a diminished yield was observed in the absence of either RuCl_3_·*n*H_2_O or NaOAc (entries 6 and 7). Particularly, the crucial presence of NaOAc suggests that carboxylate-assisted C–H activation is the operative working mode.^23^ We also tested other bench-stable ruthenium(ii) complexes, such as [RuCl_2_(*p*-cymene)]_2_ or [Ru(OAc)_2_(PPh_3_)_2_], albeit with limited success (entry 8).

**Table 1:** Reaction optimization^a^

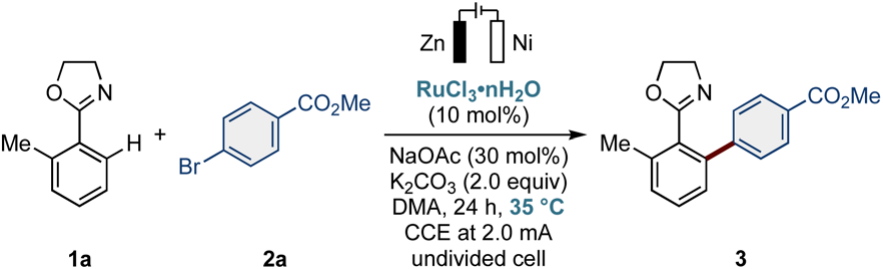		
Entry	Deviations from the above conditions	3 (%)^b^
1	None	90 (85)^c^
2	Room temperature	37
3	NMP instead of DMA	78
4	Aryl iodide or aryl chloride instead of 2a	60/73
5	No electricity but with electrodes	N.D.
6	No RuCl_3_·*n*H_2_O	N.D.
7	No NaOAc	23
8	[RuCl_2_(*p*-cymene)]_2_^d^ or [Ru(OAc)_2_(PPh_3_)_2_] instead of RuCl_3_·*n*H_2_O and no electricity	N.D.

a Reaction conditions: 1a (0.50 mmol), 2a (1.5 equiv.), RuCl_3_·*n*H_2_O (10 mol%), NaOAc (30 mol%), K_2_CO_3_ (2.0 equiv.), DMA (3.0 mL), 24 h, 35 °C, CCE at 2.0 mA, Zn anode, Ni cathode, undivided cell.

b Determined by ^1^H-NMR analysis using dibromomethane as the internal standard.

c Isolated yield.

d Catalyst loading: 5.0 mol%. CCE: constant current electrolysis, DMA: *N*,*N*-dimethylacetamide, N.D.: not detected.

### Electrocatalysis robustness

**Fig. 1:**
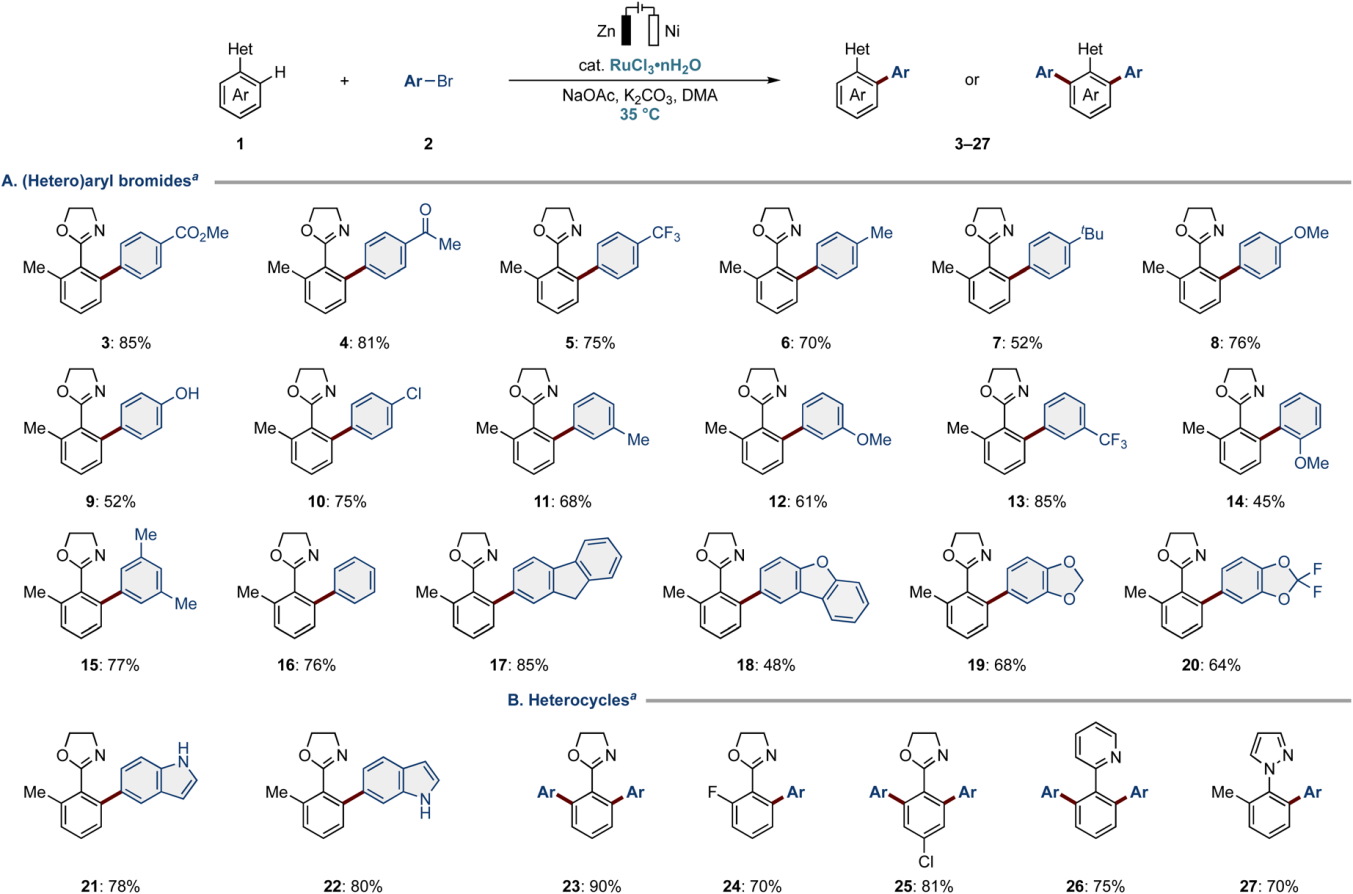
(Hetero)aryl bromide and heterocycle scope. ^*a*^Reaction conditions: 1 (0.50 mmol), 2 (1.5–3.0 equiv.), RuCl_3_·*n*H_2_O (10 mol%), NaOAc (30 mol%), K_2_CO_3_ (2.0 equiv.), DMA (3.0 mL), 24 h, 35 °C, CCE at 2.0 mA, Zn anode, Ni cathode, undivided cell. Ar: 4-(CO_2_Me)C_6_H_4_ for products 23–27.

With the optimal conditions in hand, we next explored the viable (hetero)aryl bromide scope for the electro-enabled C–H arylation (Fig. 1A). Various *para*-substituted aryl bromides were well tolerated, regardless of the electronic nature of their substituents (3–10). We were particularly pleased to find that valuable electrophilic functional groups, such as ester (3), ketone (4), hydroxy (9), and chloro (10) groups, were fully tolerated by the electrocatalysis, without any significant signs of cross-electrophile couplings (CEC)^70–80^ induced by the electricity. Furthermore, *meta*- and more challenging *ortho*-substituted aryl bromides gave the desired arylation products likewise (11–17). To our delight, the ruthena-electrocatalysis proved compatible with hetaryl bromides, such as dibenzofuran, 1,3-benzodioxoles, and NH-free indoles (18–22).

Next, we turned our attention to evaluating heterocycle-attached arenes (Fig. 1B). The benzene-substituted oxazoline yielded the difunctionalization product 23 in excellent yield. The electrocatalysis proved tolerant of the fluoro-attached aryl oxazoline (24). Notably, the chloro group on the *para*-position of the aromatic ring remained intact (25), demonstrating the high electrocatalysis chemo-selectivity and its potential for further diversification. We were pleased to selectively obtain arylation products using pyridine and pyrazole-derived substrates (26 and 27).

**Fig. 2:**
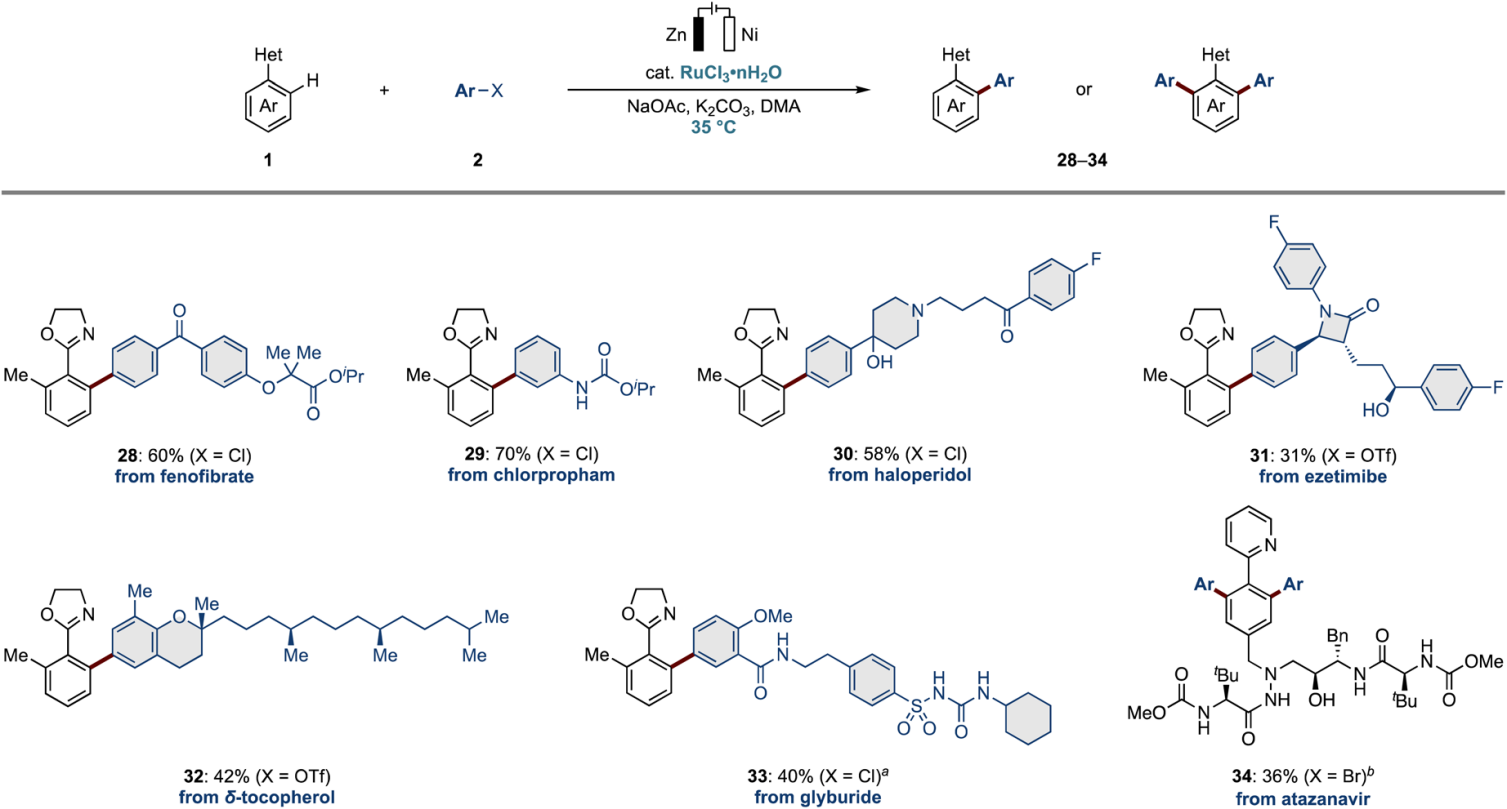
Electro-late-stage diversification. General reaction conditions: 1 (0.50 mmol), 2 (1.5–3.0 equiv.), RuCl_3_·*n*H_2_O (10 mol%), NaOAc (30 mol%), K_2_CO_3_ (2.0 equiv.), DMA (3.0 mL), 24 h, 35 °C, CCE at 2.0 mA, Zn anode, Ni cathode, undivided cell. ^*a*^ Reaction temperature: 50 °C. ^*b*^RuCl_3_·*n*H_2_O: 20 mol%, NaOAc: 60 mol%, K_2_CO_3_: 3.0 equiv., 70 °C. Ar: *m*-xylyl for product 34. Bn: benzyl.

We have confirmed, thus far, that our electrochemical C–H arylation efficiently proceeded under mild conditions to furnish various arylation products. Hence, we next explored the challenging late-stage diversification of complex molecules (Fig. 2). Thus, we employed commercially available pharmaceutical and agrochemical compounds bearing chloro leaving groups. For instance, fenofibrate and chlorpropham were successfully incorporated into the aryl oxazoline scaffolds, even when sensitive ester and carbamate functional groups were present (28 and 29). The drug haloperidol with a free OH-hydroxyl group was fully tolerated by the ruthenium electrocatalysis (30). To our delight, ezetimibe and δ-tocopherol furnished the target arylation products 31 and 32. Furthermore, glyburide was also tolerated, leading to the arylation product bearing a sulfonylurea fragment, one of the important building blocks in medicinal chemistry (33).^81^ We were also pleased that the azapeptide drug, atazanavir, underwent selective functionalization on the phenylpyridine scaffold (34).

**Fig. 3:**
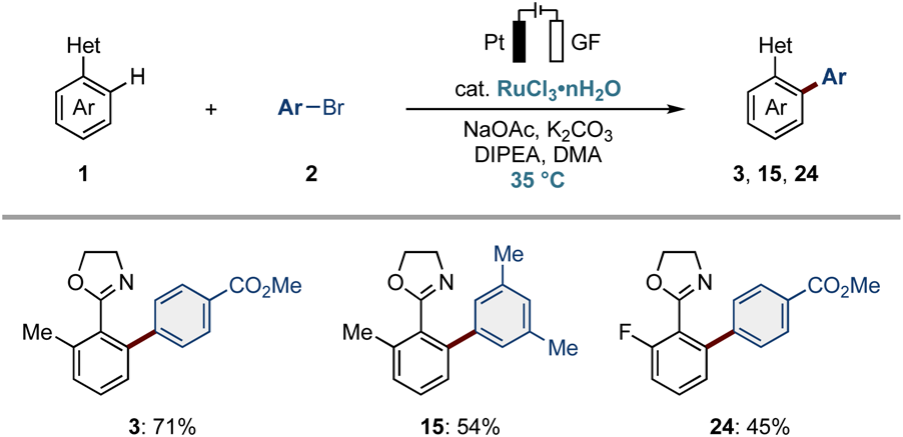
Sacrificial anode-free electrochemical ruthenium-catalyzed *ortho*-C–H arylation. Reaction conditions: 1 (1.2 equiv.), 2 (0.50 mmol), RuCl_3_·*n*H_2_O (10 mol%), NaOAc (30 mol%), K_2_CO_3_ (2.0 equiv.), DIPEA (2.0 equiv.), DMA (3.0 mL), 24 h, 35 °C, CCE at 2.0 mA, platinum anode, GF cathode, undivided cell.

To replace the zinc anode, we evaluated *N*,*N*-diisopropylethylamine (DIPEA) as a terminal reductant with a stable anode material.^82^ Upon experimentation,^83^ the desired products 3, 15, and 24 were selectively obtained without a sacrificial anode (Fig. 3). Detailed mass-spectrometry analysis confirmed the Hünig base (DIPEA) serving as the terminal reductant.

**Fig. 4:**
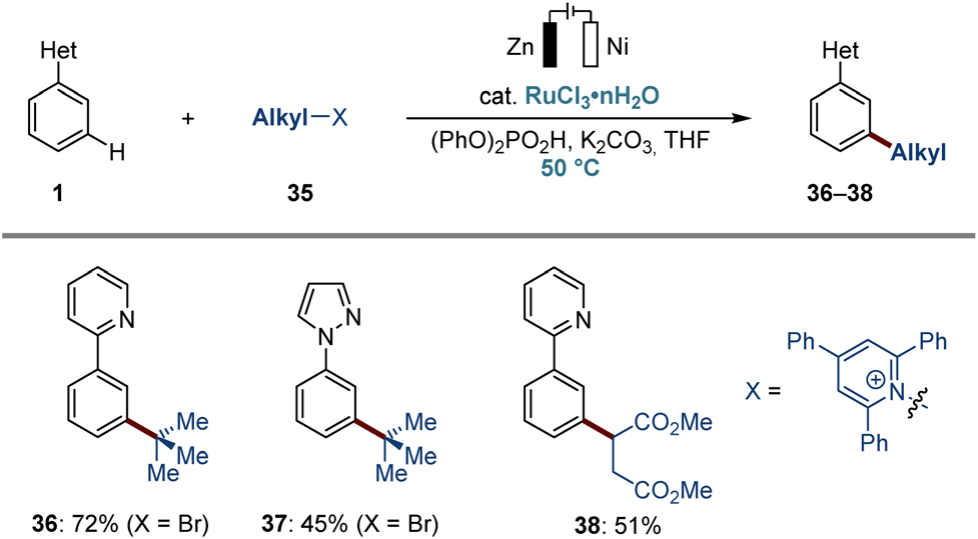
Electrochemical ruthenium-catalyzed *meta*-C–H alkylation. Reaction conditions: 1 (0.50 mmol), 35 (2.0–3.0 equiv.), RuCl_3_·*n*H_2_O (10 mol%), (PhO)_2_PO_2_H (30 mol%), K_2_CO_3_ (2.0 equiv.), ^*n*^Bu_4_NPF_6_ (50 mM), THF (3.0 mL), 50 °C, CCE at 1.0 mA, 4 h, and 13 h at 50 °C, Zn anode, Ni cathode, undivided cell. THF: tetrahydrofuran.

The ruthena-electrocatalysis was not limited to *ortho*-C–H arylations. Indeed, it also proved to be applicable to challenging *meta*-C–H alkylations, when employing secondary and tertiary alkyl (pseudo)halides (Fig. 4). By the judicious choice of additive and solvent, we accomplished the *meta*-C–H alkylations of arenes, thus giving the desired products 36 and 37. Inspired by our previous findings,^84^ we also tested the pyridinium salt derived from aspartic acid and successfully isolated the corresponding *meta*-secondary alkylation product 38.

### Mechanistic studies

**Fig. 5:**
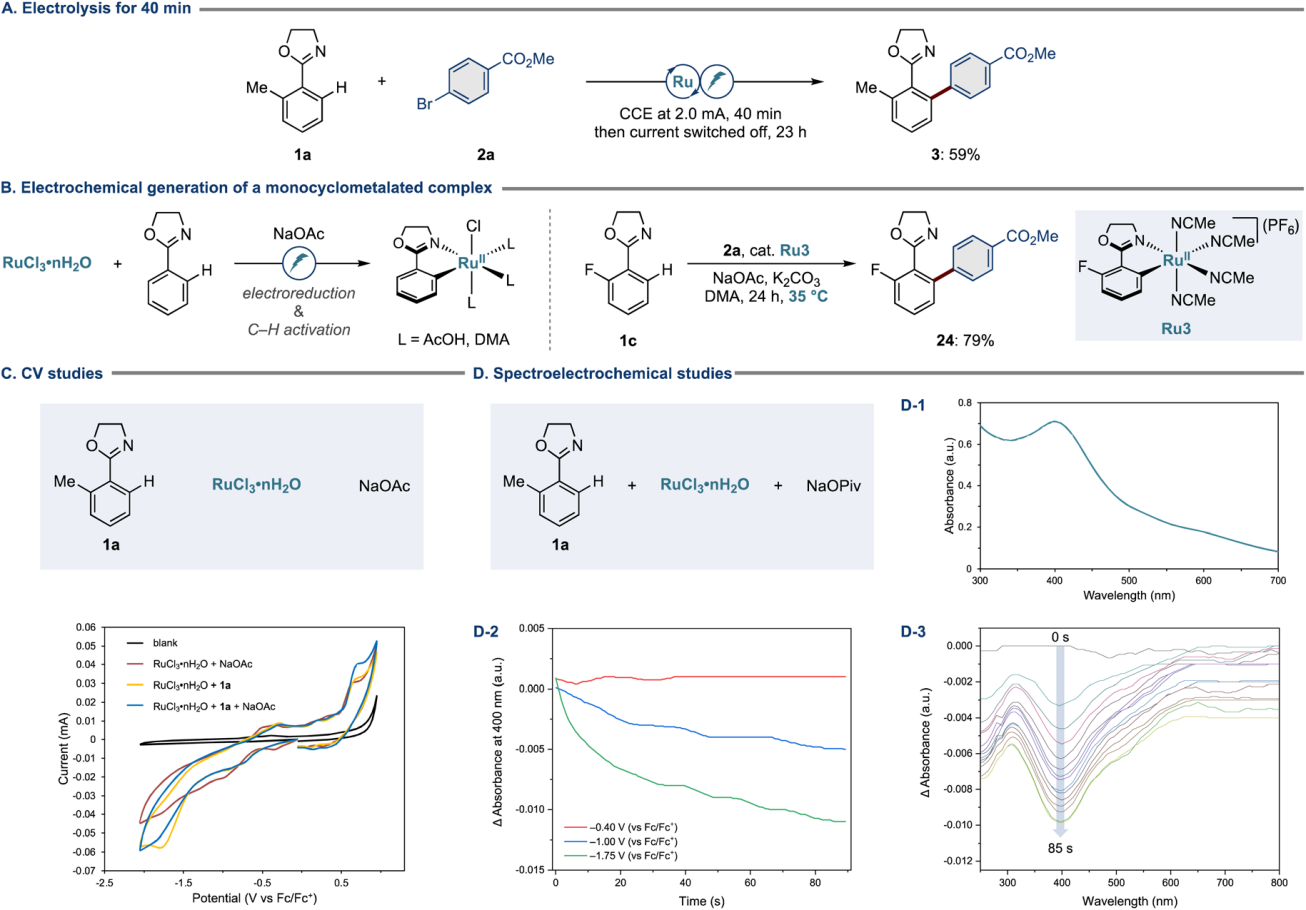
(A) Electrolysis for 40 min. (B) Electrochemical generation of a monocyclometalated complex. (C) CV studies. (D-1) UV/vis absorption spectrum of the DMA solution containing substrate 1a (0.13 mM), RuCl_3_·*n*H_2_O (0.13 mM), and NaOPiv (0.13 mM). (D-2) Spectroelectrochemical analysis of the DMA solution containing substrate 1a (0.2 mM), RuCl_3_·*n*H_2_O (0.2 mM), NaOPiv (0.2 mM), and ^*n*^Bu_4_NPF_6_ (0.1 M) at 35 °C under N_2_. Measurements were performed at −0.40, −1.00, or −1.75 V. (D-3) Spectroelectrochemical analysis performed at −1.75 V (*vs.* Fc/Fc^+^) over 85 s.

Having validated the robustness of the new ruthenium electrocatalysis, we turned our attention to elucidating the modus operandi. First, we performed a control experiment wherein 10 mol% of electrons were supplied within the initial 40 minutes, and the current was switched off thereafter (Fig. 5A). Here, the arylation product 3 was obtained in 59% yield. This outcome suggests that electricity is primarily required to induce the formation of the key monocyclometalated ruthenium(ii) intermediate (Fig. 5B, left). The involvement of such a species was verified by the experiment using a well-defined monocyclometalated complex Ru3, in which we obtained the desired arylation product 24 in 79% yield, further corroborating the electrochemical initiation being operative (Fig. 5B, right).

To rationalize the electrochemical features of our electrocatalysis, we conducted detailed cyclic voltammetry (CV) studies using RuCl_3_·*n*H_2_O, substrate 1a, and NaOAc (Fig. 5C). Interestingly, the presence of substrate 1a resulted in a pronounced irreversible reduction event at −1.7 V, implying coordination of arene 1a to ruthenium. In all cases, oxidation events were observed at 0.7 V. To gain more insights, we selected specific voltage values from the cyclic voltammograms and sought to observe the potential-dependent consumption of the ruthenium(iii) precatalyst by means of spectroelectrochemistry. Thus, we conducted potentiostatic analysis mode and focused on the absorption of the ruthenium(iii) precatalyst at 400 nm (Fig. 5D-1). As depicted in Fig. 5D-2, whereas there was no significant change in the absorbance at 400 nm during the measurements at −0.40 and −1.00 V, spectroelectrochemical analysis at −1.75 V allowed us to detect a notable decrease in absorbance, which was also visible from the spectra with entire wavelengths (Fig. 5D-3). Overall, these observations are indicative of an electrochemical reduction of the ruthenium(iii) precatalyst to afford a catalytically relevant ruthenium(ii) complex. Also, electrochemical analysis by a rotating disk electrode was supportive of a ruthenium(iii)/ruthenium(ii) scenario.^83,85,86^

### DFT calculations

**Fig. 6:**
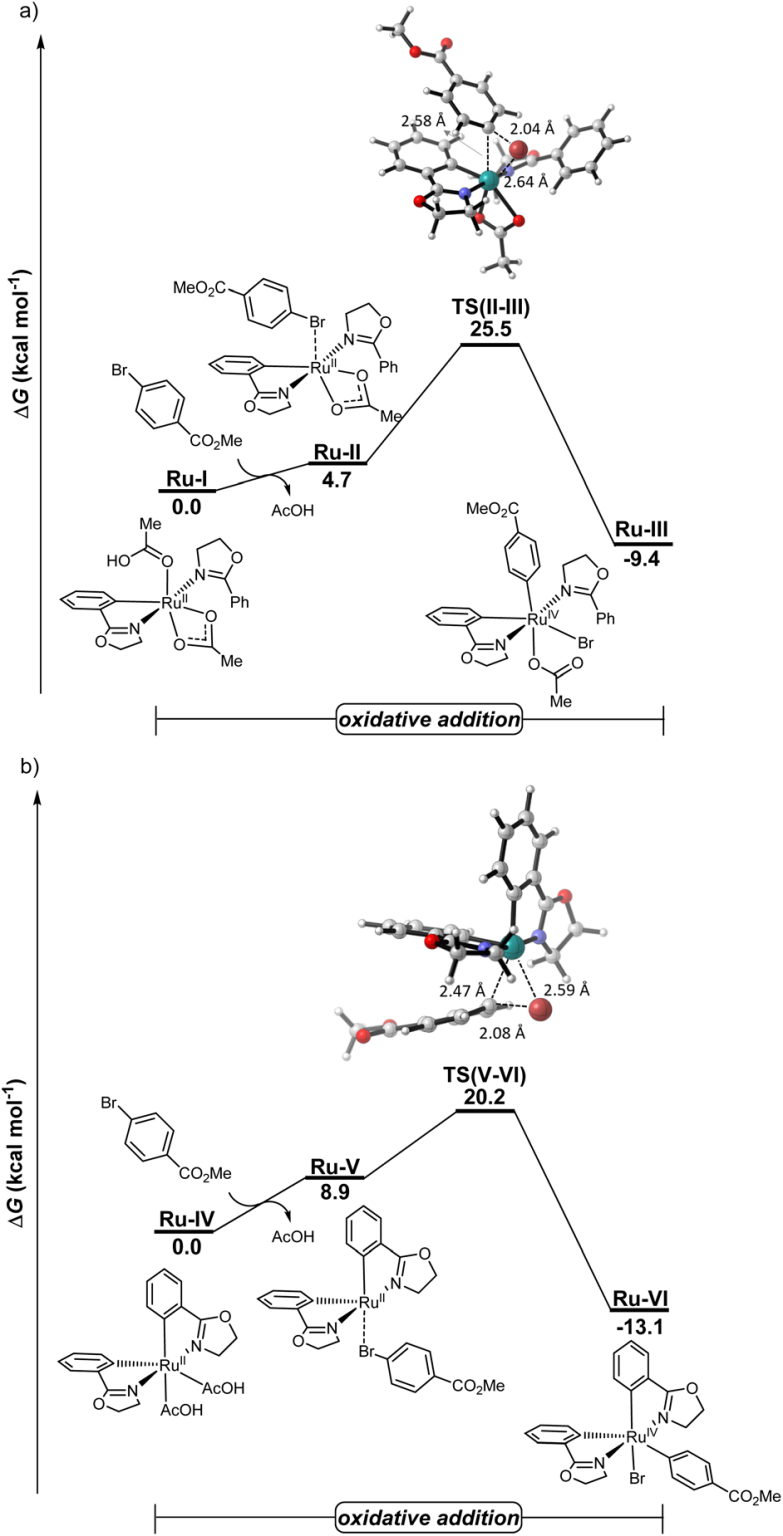
Computed relative Gibbs free energies on the oxidative addition step for both (a) monocycloruthenated and (b) biscycloruthenated species at the PBE0-D4/def2-TZVPP-SMD(DMA)//TPSS-D3(BJ)/def2-SVP level of theory.

To rationalize the *modus operandi* of the electrocatalysis, detailed density functional theory (DFT) calculations were performed for the key oxidative addition step, considering both mono- and biscycloruthenated species Ru-I and Ru-IV, respectively, at the PBE0-D4/def2-TZVPP-SMD(DMA)//TPSS-D3(BJ)/def2-SVP level of theory (Fig. 6). The biscycloruthenated complex Ru-IV, generated *via* two C–H ruthenations, exhibits an energy barrier of 20.2 kcal mol^−1^ for the oxidative addition with aryl bromide 2a, leading to the ruthenium(iv)-aryl species Ru-VI. Notably, the oxidative addition pathway involving the monocycloruthenated species is energetically disfavored with a considerably higher activation barrier of 25.5 kcal mol^−1^. These results are consistent with earlier computational studies by Ackermann^19^ and very recent findings by Macgregor^87^ for the oxidative addition occurring on the biscyclometalated ruthenium(ii) intermediates.

### Proposed mechanism

**Fig. 7:**
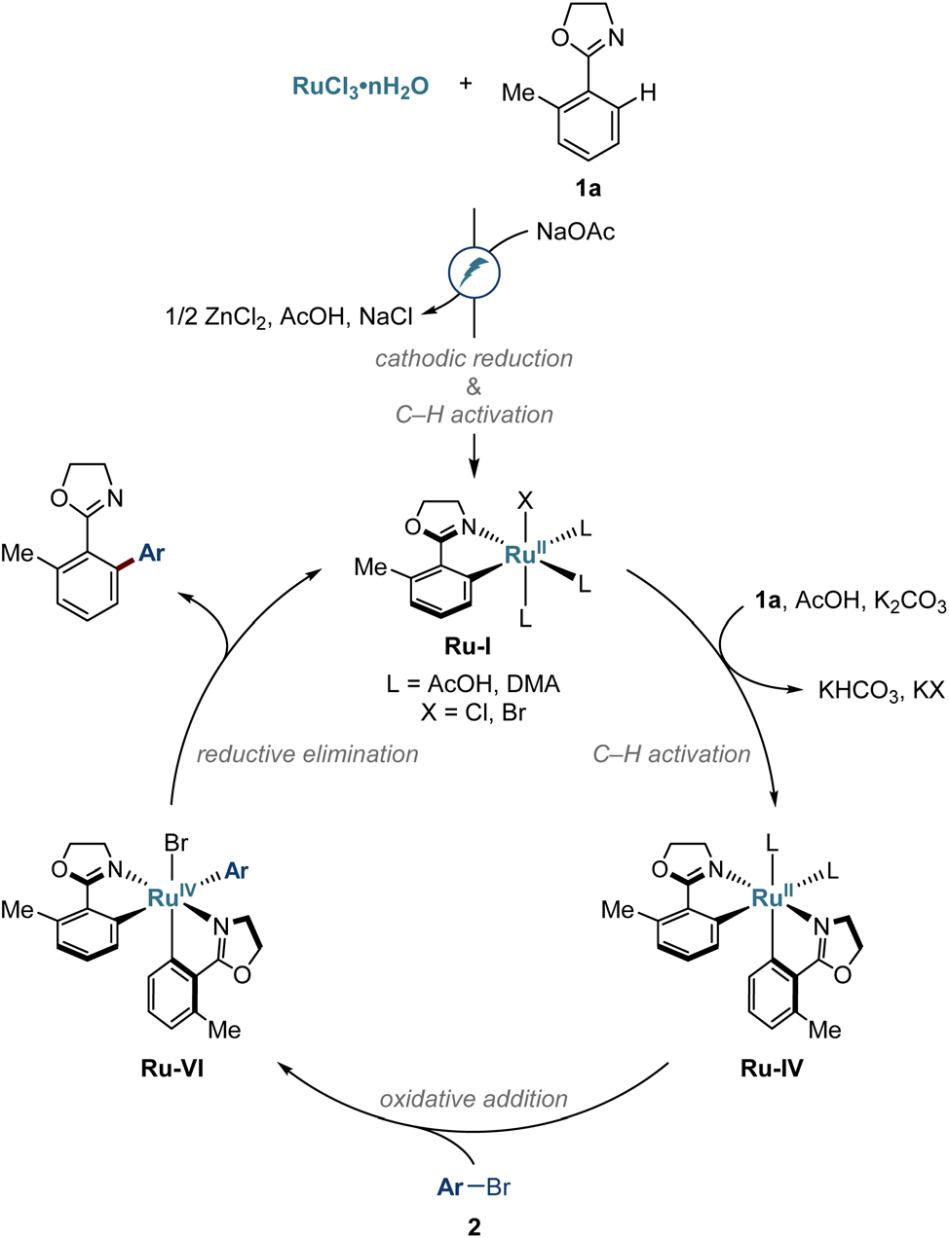
Proposed mechanism.

On the basis of our experimental and computational findings, we propose a catalytic cycle for the electro-induced C–H arylations as depicted in Fig. 7. The reaction commences upon cathodic ruthenium(iii) reduction, along with carboxylate-assisted C–H activation to form complex Ru-I. Next, biscyclometalated intermediate Ru-IV is formed and undergoes oxidative addition with aryl bromide 2. Reductive elimination from the thus formed ruthenium complex Ru-IV releases the arylation product and regenerates the complex Ru-I.

## Conclusions

In conclusion, we have realized a low-temperature ruthenium-catalyzed C–H activation directly employing commercially-available RuCl_3_·*n*H_2_O as a resource-economical precatalyst. The reaction provided selective access to *ortho*- or *meta*-functionalized products from a wide array of substrates, including structurally-complex drug molecules and natural products. Detailed electroanalysis provided strong support for a ruthenium(iii)/ruthenium(ii) manifold enabled by cathodic reduction. A sacrificial anode could be avoided. Overall, our electrocatalysis strategy offers a user-friendly and general strategy to replace the established ruthenium(ii) precatalysts in the field of C–H activation chemistry to enable catalysis under exceedingly mild conditions.

## Author contributions

Conceptualization, L. A.; methodology, T. M.; investigation, T. M.; spectroelectrochemical analysis, T. v. M.; rotating disk electrode experiments, T. M. and Z. L.; DFT calculations, B. Y. and J. C. A. O.; writing – original draft, all authors; writing – review & editing, all authors; funding acquisition, L. A.; resources, L. A.; supervision, L. A.

## Conflicts of interest

There are no conflicts to declare.

## Supplementary Material

SC-016-D5SC02780B-s001

## Data Availability

The data supporting this article have been uploaded as part of the ESI.†
