# Supplementary material for: Electroreductive room-temperature C–H activations with RuCl3·nH2O precatalyst via cathodic ruthenium(iii/ii) manifold
Source: Chem Sci. 2025 Jun 27;16(29):13468–76. doi: 10.1039/d5sc02780b (PMC12203598; doi:10.1039/d5sc02780b)
Supplement: SC-016-D5SC02780B-s001 [file SC-016-D5SC02780B-s001.pdf]

Supplementary Information (SI) for Chemical Science.
This journal is © The Royal Society of Chemistry 2025

Supporting Information

Electroreductive Room-Temperature C–H Activations with $RuCl_3 \cdot nH_2O$ Precatalyst *via* Cathodic Ruthenium(III/II) Manifold

Takuya Michiyuki, Tristan von Münchow, Zhipeng Lin, Binbin Yuan, João C. A. Oliveira, and Lutz Ackermann*

Table of Contents

1. General Remarks 3

2. Reaction Optimization 4

3. Electrochemical Setup 6

4. Experimental Procedures 6

4-1. General procedure A: ruthenium-catalyzed electrochemical *ortho*-C–H arylation using a zinc anode and a nickel foam cathode 6

4-2. General procedure B: ruthenium-catalyzed electrochemical *ortho*-C–H arylation using a platinum anode and a GF cathode 7

4-3. General procedure C: ruthenium-catalyzed electrochemical *meta*-C–H alkylation 7

4-4. Characterization data 7

5. Unsuccessful Examples 24

6. Mechanistic Studies 24

6-1. Control experiment: electrolysis for 40 min 24

6-2. Cyclic voltammetry 24

6-3. Spectroelectrochemistry (SEC) 26

6-4. Monocyclometalated complex as the catalyst 27

6-5. Stoichiometric reactions 27

6-6. Determination of electron transfer number 28

7. Computational Studies 34

7-1. Computation methods 34

7-2. Cartesian coordinates of the optimized structure 34

8. NMR Spectra 45

9. References 86

1. General Remarks

Catalytic reactions were carried out in undivided electrochemical cells under N_2 unless otherwise noted. *N*,*N*-dimethylacetamide (DMA) and *N*-methyl-2-pyrrolidone (NMP) were dried over calcium hydride and distilled under reduced pressure. Tetrahydrofuran (THF) and acetonitrile (MeCN) were purified by an MBRAUN solvent purification system and degassed by freeze-pump-thaw cycles three times. Aryl oxazolines were synthesized using the method previously described.[1] Aryl triflates were prepared based on the previously reported procedure.[2] The pyridinium salt for product **38** was synthesized by following the known procedure.[3] The other substrates were obtained from commercial sources and used without further purification. **Ru2** and **Ru3** were synthesized by using the previously described method.[4-6] Zinc electrodes (25 mm × 10 mm × 0.50 mm, obtained from abcr GmbH, Germany), nickel foam electrodes (25 mm × 10 mm × 1.4 mm, RCM-Ni5763, obtained from Recemat BV, Germany), platinum electrodes (25 mm × 10 mm × 0.125 mm, 99.9%, obtained from ESG Edelmetall-Handel GmbH & Co. KG, Rheinstetten, Germany), and graphite felt (GF) electrodes (25 mm × 10 mm × 5 mm, SIGRACELL GFA6, obtained from SGL Carbon GmbH, Meitlingen, Germany) were connected using stainless steel adapters. Electrocatalysis was conducted using a Rohde & Schwarz galvanostat (HMP4040) in a constant current mode.

Column chromatography was carried out on Merck silica gel 60 (40–63 µm). Ethyl acetate (EtOAc), *n*-hexane, and dichloromethane (DCM) were distilled prior to use.

NMR spectra were recorded on a Bruker Avance 300 and a Bruker Avance Neo 400 at 300 MHz, 400 MHz, (^{1}H-NMR), 75 MHz, 101 MHz (^{13}C-NMR), and 282 MHz, 377 MHz (^{19}F-NMR) in the solvent indicated. Chemical shifts (δ) are given in ppm relative to the residual solvent peak. ^{1}H-NMR data are reported as follows: chemical shift, multiplicity (s = singlet, d = doublet, t = triplet, q = quartet, quint = quintet, h = heptet, dd = doublet of doublets, tt = triplet of triplets, td = triplet of doublets, m = multiplet, *etc.*), coupling constants (Hz), and the number of protons.

IR spectra were recorded on a Bruker FT-IR Alpha device.

EI-MS spectra were recorded on a Jeol AccuTOF at 70 eV and ESI-MS-spectra on a Bruker microTOF and a maXis.

Melting points were determined on a Stuart melting point apparatus SMP3 from Barloworld Scientific.

Cyclic voltammetry and rotating disk electrode (RDE) studies were conducted using a Metrohm Autolab PGSTAT204 workstation, Nova 2.1, and an RRDE-3A Rotating Ring Disk Electrode Apparatus Ver.2.0 from ALS JAPAN Co., Ltd. (for RDE studies).

UV-vis spectra were recorded on a JASCO V-770.

Spectroelectrochemical (SEC) studies were performed using an AUTOLAB potentiostat with the TSC Spectro cell from rhd instruments (Pt mesh working electrode; Glassy carbon counter electrode; silver wire pseudo reference electrode). Temperature control was ensured with the AUTOLAB Microcell HC. Avantes AvaLight-DH-S-BAL was used as the light source, and an Avantes AvaSpec-ULS2048x64-EVO was used for absorption measurement. The evaluation of the data was carried out with the software AvaSoft 8 from Avantes and Nova 2.1.

2. Reaction Optimization

Table S1. Reaction optimization for the *ortho*-C–H arylation using a zinc/nickel electrode system.[a]

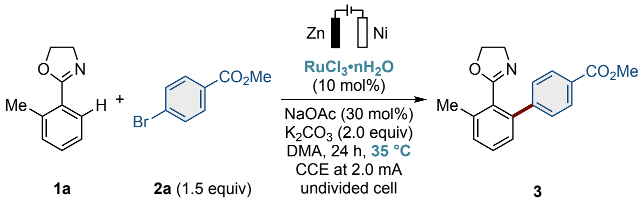

Entry	Deviations	Yield of **3** (%)[b]
1	None	90 (85)[c]
2	Room temperature	37
3	NMP instead of DMA	78
4	Aryl iodide or aryl chloride instead of **2a**	60 / 73
5	No electricity but with electrodes	N.D.
6	No $RuCl_3$•nH_2O	N.D.
7	No NaOAc	23
8	$[RuCl_2(p\text{-cymene})]_2$[d] or $[Ru(OAc)_2(PPh_3)_2]$ instead of $RuCl_3$•nH_2O<br>No electricity	N.D.
9	**Ru2** instead of $RuCl_3$•nH_2O<br>No electricity	75
10[e]	DMF instead of DMA	45
11[e]	THF instead of DMA	72
12[e]	MeCN instead of DMA	N.D.
13	NaOPiv instead of NaOAc	71
14	Fe anode instead of Zn anode	35
15	Al anode instead of Zn anode	Trace

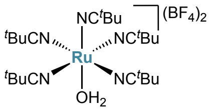

Ru2

[a] Reaction conditions: **1a** (0.50 mmol), **2a** (1.5 equiv), $RuCl_3$•nH_2O (10 mol%), NaOAc (30 mol%), K_2CO_3 (2.0 equiv), DMA (3.0 mL), 24 h, 35 °C, CCE at 2.0 mA, zinc plate anode, nickel foam cathode, undivided cell. [b] Determined by ^{1}H-NMR analysis using dibromomethane as the internal standard. [c] Isolated yield. [d] Catalyst loading: 5.0 mol%. [e] $^n Bu_4NPF_6$ (50 mM) was added as the electrolyte. CCE: constant current electrolysis, N.D.: not detected.

Table S2. Reaction optimization for the *ortho*-C–H arylation under sacrificial anode-free conditions.[a]

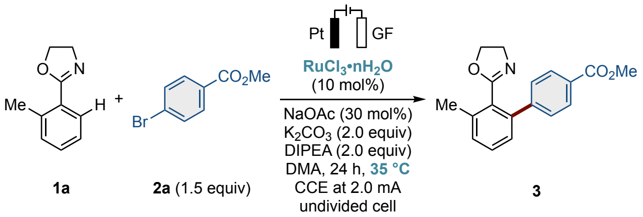

Entry	Deviations	Yield of **3** (%)[b]
1	None	52
2	DIPEA (3.0 equiv)	40
3	DIPEA (1.0 equiv)	29
4	MeCN instead of DMA	N.D.
5	50 °C	23
6	Room temperature	11
7	**2a** (3.0 equiv)	33
8	**1a** : **2a** = 1.2 : 1	75 (71)[c]

[a] Reaction conditions: **1a** (0.50 mmol), **2a** (1.5 equiv), $RuCl_3 \cdot nH_2O$ (10 mol%), NaOAc (30 mol%), K_2CO_3 (2.0 equiv), DIPEA (2.0 equiv), DMA (3.0 mL), 24 h, 35 °C, CCE at 2.0 mA, platinum anode, graphite felt (GF) cathode, undivided cell. [b] Determined by ^{1}H-NMR analysis using dibromomethane as the internal standard. [c] Isolated yield. DIPEA: *N*,*N*-diisopropylethylamine.

Table S3. Reaction optimization for the *meta*-C–H alkylation.[a]

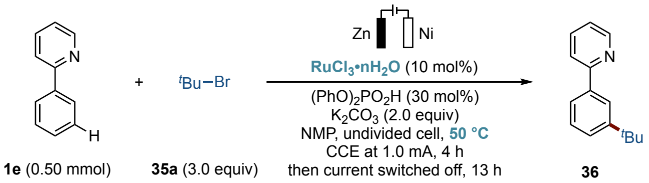

Entry	Deviations	Yield of **36** (%)[b]
1	None	58
2	17 h of electrolysis	49
3[c]	THF instead of NMP	74 (72)[d]
Deviations from entry 3		
4[c]	35 °C	49
5[c]	35 °C, 2 h of electrolysis	31

[a] Reaction conditions: **1e** (0.50 mmol), **35a** (3.0 equiv), $RuCl_3 \cdot nH_2O$ (10 mol%), $(PhO)_2PO_2H$ (30 mol%), K_2CO_3 (2.0 equiv), NMP (3.0 mL), 50 °C, CCE at 1.0 mA, 4 h, then current switched off, 13 h, zinc plate anode, nickel foam cathode, undivided cell. [b] Determined by ^{1}H-NMR analysis using dibromomethane as the internal standard. [c] $^nBu_4NPF_6$ (50 mM) was added as the electrolyte. [d] Isolated yield.

3. Electrochemical Setup

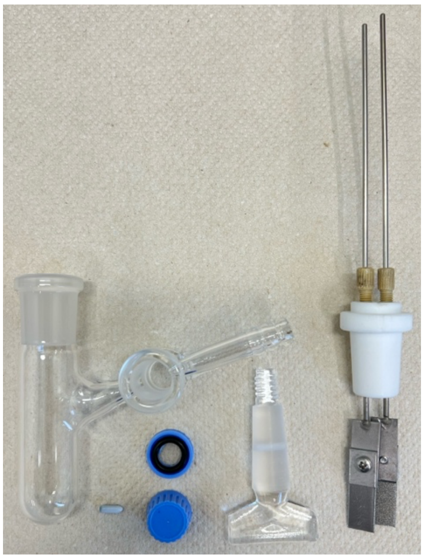

Figure S1. Picture of the electrochemical cell, stirring bar, and Teflon cap with a zinc plate and a nickel foam.

4. Experimental Procedures

4-1. General procedure A: ruthenium-catalyzed electrochemical *ortho*-C–H arylation using a zinc anode and a nickel foam cathode

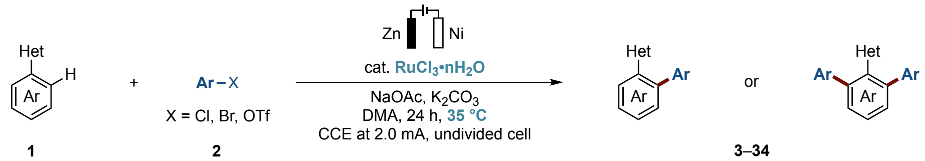

The electrolysis was carried out in an undivided cell with a zinc anode (25 mm × 10 mm × 0.50 mm) and a nickel foam cathode (25 mm × 10 mm × 1.4 mm). Arene **1** (0.50 mmol, 1.0 equiv), aryl (pseudo)halide **2** (0.75–1.5 mmol, 1.5–3.0 equiv), $RuCl_3$•nH_2O (13 mg, 50 µmol, 10 mol%), NaOAc (12 mg, 0.15 mmol, 0.30 equiv), K_2CO_3 (138 mg, 1.00 mmol, 2.00 equiv), and DMA (3.0 mL) were charged in the cell under N_2. The electrolysis was performed in a constant current mode at 2.0 mA at 35 °C (relevant electrode surface area = 0.80 cm^2, j = 2.5 mA cm^{-2}). After 24 h (1.8 F mol^{-1}), the reaction mixture was transferred into a separatory funnel, and the electrodes were rinsed with EtOAc (5 mL). Saturated NH_4Cl aqueous solution (10 mL) was added, and the aqueous layer was extracted with EtOAc (3 × 20 mL). The combined organic layers were washed with water (2 × 20 mL) and brine (20 mL). Afterward, the organic phase was dried over Na_2SO_4, filtered, and concentrated. The crude reaction mixture was purified by silica gel column chromatography (eluent: *n*-hexane/DCM/EtOAc) to yield the desired product.

4-2. General procedure B: ruthenium-catalyzed electrochemical *ortho*-C–H arylation using a platinum anode and a GF cathode

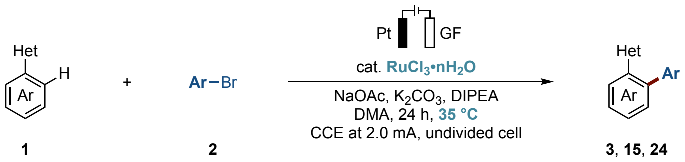

The electrolysis was carried out in an undivided cell with a platinum anode (25 mm × 10 mm × 0.50 mm) and a GF cathode (25 mm × 10 mm × 1.4 mm). Arene **1** (0.60 mmol, 1.2 equiv), aryl bromide **2** (0.50 mmol, 1.0 equiv), $RuCl_3$•nH_2O (13 mg, 50 μmol, 10 mol%), NaOAc (12 mg, 0.15 mmol, 0.30 equiv), K_2CO_3 (138 mg, 1.00 mmol, 2.00 equiv), *N*,*N*-diisopropylethylamine (DIPEA) (174 μL, 1.00 mmol, 2.00 equiv), and DMA (3.0 mL) were charged in the cell under N_2. The electrolysis was performed in a constant current mode at 2.0 mA at 35 °C (relevant electrode surface area = 0.80 cm^2, j = 2.5 mA cm^{-2}). After 24 h (1.8 F mol^{-1}), the reaction mixture was transferred into a separatory funnel, and the electrodes were rinsed with EtOAc (5 mL). Saturated NH_4Cl aqueous solution (10 mL) was added, and the aqueous layer was extracted with EtOAc (3 × 20 mL). The combined organic layers were washed with water (2 × 20 mL) and brine (20 mL). Afterward, the organic phase was dried over Na_2SO_4, filtered, and concentrated. The crude reaction mixture was purified by silica gel column chromatography (eluent: *n*-hexane/EtOAc) to yield the desired product.

4-3. General procedure C: ruthenium-catalyzed electrochemical *meta*-C–H alkylation

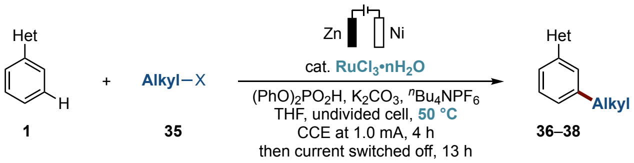

The electrolysis was carried out in an undivided cell with a zinc anode (25 mm × 10 mm × 0.50 mm) and a nickel foam cathode (25 mm × 10 mm × 1.4 mm). Arene **1** (0.50 mmol, 1.0 equiv), alkyl electrophile **35** (1.0–1.5 mmol, 2.0–3.0 equiv), $RuCl_3$•nH_2O (13 mg, 50 μmol, 10 mol%), $(PhO)_2PO_2H$ (38 mg, 0.15 mmol, 0.30 equiv), K_2CO_3 (138 mg, 1.00 mmol, 2.00 equiv), ^{n}Bu$_4$NPF$_6$ (58 mg, 50 mM), and THF (3.0 mL) were charged in the cell under N_2. The electrolysis was performed in a constant current mode at 1.0 mA at 50 °C (relevant electrode surface area = 0.80 cm^2, j = 1.3 mA cm^{-2}). After 4 h (0.30 F mol^{-1}), the current was switched off, and the reaction was continued for the next 13 h at 50 °C. The reaction mixture was filtered through a pad of Celite with DCM, and the solvent was removed under reduced pressure. The crude reaction mixture was purified by silica gel column chromatography (eluent: *n*-hexane/EtOAc) to yield the desired product.

4-4. Characterization data

methyl 2'-(4,5-dihydrooxazol-2-yl)-3'-methyl-[1,1'-biphenyl]-4-carboxylate (3)

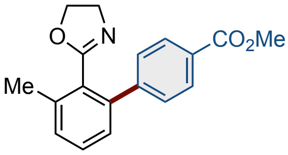

General procedure **A** was followed using 2-(*o*-tolyl)-4,5-dihydrooxazole (80.6 mg, 0.50 mmol) and methyl 4-bromobenzoate (161.3 mg, 0.75 mmol). Purification by column chromatography on silica gel (*n*-hexane/EtOAc = 1:1) yielded product **3** (126.1 mg, 85%) as a white solid. General procedure **B** afforded product **3** in 71% yield.

^{1}H-NMR (400 MHz, $CDCl_3$): δ = 8.09 – 7.99 (m, 2H), 7.52 – 7.43 (m, 2H), 7.37 (t, J = 7.7 Hz, 1H), 7.29 – 7.17 (m, 2H), 4.13 (t, J = 9.5 Hz, 2H), 3.92 (s, 3H), 3.84 (t, J = 9.5 Hz, 2H), 2.42 (s, 3H).

^{13}**C-NMR** (101 MHz, $CDCl_3$): δ = 167.1 (C_q), 164.2 (C_q), 146.1 (C_q), 141.1 (C_q), 137.9 (C_q), 129.8 (CH), 129.7 (CH), 129.4 (CH), 128.9 (C_q), 128.6 (CH), 128.2 (C_q), 127.1 (CH), 67.3 (CH_2), 55.3 (CH_2), 52.2 (CH_3), 19.9 (CH_3).

IR (ATR): $\tilde{\nu}$ = 1720, 1663, 1435, 1276, 1251, 1117, 1102, 1043, 937, 770 cm^{-1}.
m.p.: 118–119 °C.
HR-MS (ESI): *m*/*z* calcd for $C_{18}H_{18}NO_3^+$ $[M+H]^+$ 296.1281, found 296.1290.
The characterization data are consistent with those reported in the literature.[7]

1-(2'-(4,5-dihydrooxazol-2-yl)-3'-methyl-[1,1'-biphenyl]-4-yl)ethan-1-one (4)

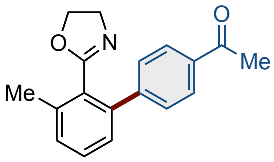

General procedure **A** was followed using 2-(*o*-tolyl)-4,5-dihydrooxazole (80.6 mg, 0.50 mmol) and 1-(4-bromophenyl)ethan-1-one (149.3 mg, 0.75 mmol). Purification by column chromatography on silica gel (*n*-hexane/EtOAc = 1:1) yielded product **4** (113.0 mg, 81%) as a white solid.

^{1}**H-NMR** (400 MHz, $CDCl_3$): δ = 8.00 – 7.93 (m, 2H), 7.54 – 7.46 (m, 2H), 7.37 (t, *J* = 7.7 Hz, 1H), 7.29 – 7.24 (m, 1H), 7.23 – 7.17 (m, 1H), 4.15 (t, *J* = 9.5 Hz, 2H), 3.85 (t, *J* = 9.5 Hz, 2H), 2.62 (s, 3H), 2.42 (s, 3H).

^{13}**C-NMR** (101 MHz, $CDCl_3$): δ = 198.0 (C_q), 164.2 (C_q), 146.3 (C_q), 140.9 (C_q), 138.0 (C_q), 135.9 (C_q), 129.9 (CH), 129.8 (CH), 128.8 (CH), 128.3 (CH), 128.2 (C_q), 127.1 (CH), 67.4 (CH_2), 55.3 (CH_2), 26.8 (CH_3), 19.9 (CH_3).

IR (ATR): $\tilde{\nu}$ = 1681, 1660, 1606, 1356, 1268, 1250, 1043, 937, 790, 602 cm^{-1}.
m.p.: 89–90 °C.
HR-MS (ESI): *m*/*z* calcd for $C_{18}H_{18}NO_2^+$ $[M+H]^+$ 280.1332, found 280.1342.
The characterization data are consistent with those reported in the literature.[7]

2-(3-methyl-4'-(trifluoromethyl)-[1,1'-biphenyl]-2-yl)-4,5-dihydrooxazole (5)

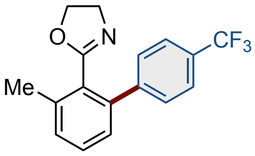

General procedure **A** was followed using 2-(*o*-tolyl)-4,5-dihydrooxazole (80.6 mg, 0.50 mmol) and 1-bromo-4-(trifluoromethyl)benzene (168.8 mg, 0.75 mmol). Purification by column chromatography on silica gel (*n*-hexane/EtOAc = 3:1) yielded product **5** (115.2 mg, 75%) as a colorless oil.

^{1}**H-NMR** (400 MHz, $CDCl_3$): δ = 7.67 – 7.60 (m, 2H), 7.58 – 7.47 (m, 2H), 7.38 (t, *J* = 7.6 Hz, 1H), 7.30 – 7.25 (m, 1H), 7.23 – 7.16 (m, 1H), 4.20 – 4.10 (m, 2H), 3.91 – 3.82 (m, 2H), 2.43 (s, 3H).

^{13}**C-NMR** (101 MHz, $CDCl_3$): δ = 164.2 (C_q), 145.0 (q, J_{CF} = 1.3 Hz, C_q), 140.7 (C_q), 138.0 (C_q), 129.9 (CH), 129.8 (CH), 129.4 (q, J_{CF} = 32.5 Hz, C_q), 128.9 (CH), 128.3 (C_q), 127.2 (CH), 125.1 (q, J_{CF} = 3.8 Hz, CH), 124.4 (q, J_{CF} = 272.0 Hz, C_q), 67.4 (CH_2), 55.3 (CH_2), 19.9 (CH_3).

^{19}**F-NMR** (282 MHz, $CDCl_3$): δ = −62.4.

IR (ATR): $\tilde{\nu}$ = 1321, 1161, 1122, 1106, 1062, 1041, 1018, 937, 844, 789 cm^{-1}.
HR-MS (ESI): *m*/*z* calcd for $C_{17}H_{15}NOF_3^+$ $[M+H]^+$ 306.1100, found 306.1107.
The characterization data are consistent with those reported in the literature.[8]

2-(3,4'-dimethyl-[1,1'-biphenyl]-2-yl)-4,5-dihydrooxazole (6)

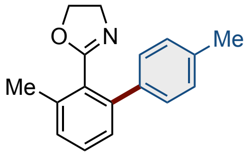

General procedure **A** was followed using 2-(*o*-tolyl)-4,5-dihydrooxazole (80.6 mg, 0.50 mmol) and 1-bromo-4-methylbenzene (128.3 mg, 0.75 mmol). Purification by column chromatography on silica gel (*n*-hexane/EtOAc = 3:1) yielded product **6** (88.0 mg, 70%) as a colorless oil.

^{1}H-NMR (400 MHz, $CDCl_3$): δ = 7.39 – 7.29 (m, 3H), 7.25 – 7.14 (m, 4H), 4.21 – 4.11 (m, 2H), 3.93 – 3.83 (m, 2H), 2.42 (s, 3H), 2.38 (s, 3H).

^{13}C-NMR (101 MHz, $CDCl_3$): δ = 164.7 (C_q), 142.1 (C_q), 138.4 (C_q), 137.6 (C_q), 136.8 (C_q), 129.6 (CH), 128.9 (CH), 128.9 (CH), 128.4 (CH), 128.2 (C_q), 127.4 (CH), 67.3 (CH_2), 55.3 (CH_2), 21.3 (CH_3), 19.9 (CH_3).

IR (ATR): $\tilde{\nu}$ = 1664, 1461, 1346, 1251, 1234, 1043, 938, 824, 786, 758 cm^{-1}.
HR-MS (ESI): *m*/*z* calcd for $C_{17}H_{18}NO^+$ $[M+H]^+$ 252.1383, found 252.1392.
The characterization data are consistent with those reported in the literature.[9]

2-(4'-(*tert*-butyl)-3-methyl-[1,1'-biphenyl]-2-yl)-4,5-dihydrooxazole (7)

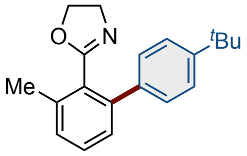

General procedure **A** was followed using 2-(*o*-tolyl)-4,5-dihydrooxazole (80.6 mg, 0.50 mmol) and 1-bromo-4-(*tert*-butyl)benzene (159.8 mg, 0.75 mmol). Purification by column chromatography on silica gel (*n*-hexane/EtOAc = 5:1) yielded product **7** (76.5 mg, 52%) as a pale-yellow oil.

^{1}H-NMR (400 MHz, $CDCl_3$): δ = 7.42 – 7.32 (m, 5H), 7.25 – 7.19 (m, 2H), 4.14 (td, *J* = 9.5, 0.9 Hz, 2H), 3.88 (td, *J* = 9.5, 0.9 Hz, 2H), 2.42 (s, 3H), 1.35 (s, 9H).

^{13}C-NMR (101 MHz, $CDCl_3$): δ = 164.7 (C_q), 150.0 (C_q), 142.1 (C_q), 138.3 (C_q), 137.6 (C_q), 129.6 (CH), 128.9 (CH), 128.2 (C_q), 128.1 (CH), 127.4 (CH), 125.1 (CH), 67.3 (CH_2), 55.3 (CH_2), 34.6 (C_q), 31.5 (CH_3), 20.0 (CH_3).

IR (ATR): $\tilde{\nu}$ = 2963, 1664, 1461, 1251, 1043, 938, 836, 789, 759, 587 cm^{-1}.
HR-MS (ESI): *m*/*z* calcd for $C_{20}H_{23}NONa^+$ $[M+Na]^+$ 316.1672, found 316.1680.

2-(4'-methoxy-3-methyl-[1,1'-biphenyl]-2-yl)-4,5-dihydrooxazole (8)

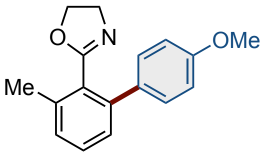

General procedure **A** was followed using 2-(*o*-tolyl)-4,5-dihydrooxazole (80.6 mg, 0.50 mmol) and 1-bromo-4-methoxybenzene (140.3 mg, 0.75 mmol). Purification by column chromatography on silica gel (*n*-hexane/EtOAc = 2:1→1:1) yielded product **8** (101.1 mg, 76%) as a colorless oil.

^{1}H-NMR (400 MHz, $CDCl_3$): δ = 7.39 – 7.29 (m, 3H), 7.19 (d, J = 7.7 Hz, 2H), 6.95 – 6.87 (m, 2H), 4.16 (td, J = 9.5, 0.8 Hz, 2H), 3.88 (td, J = 9.5, 0.8 Hz, 2H), 3.84 (s, 3H), 2.41 (s, 3H).

^{13}C-NMR (101 MHz, $CDCl_3$): δ = 164.7 (C_q), 159.0 (C_q), 141.7 (C_q), 137.6 (C_q), 133.9 (C_q), 129.7 (CH), 129.6 (CH), 128.7 (CH), 128.3 (C_q), 127.3 (CH), 113.6 (CH), 67.3 (CH_2), 55.4 (CH_3), 55.3 (CH_2), 20.0 (CH_3).

IR (ATR): $\tilde{\nu}$ = 1666, 1610, 1514, 1461, 1240, 1180, 1044, 1032, 835, 790 cm^{-1}.
HR-MS (ESI): *m*/*z* calcd for $C_{17}H_{18}NO_2^+$ $[M+H]^+$ 268.1332, found 268.1343.
The characterization data are consistent with those reported in the literature.[7]

2'-(4,5-dihydrooxazol-2-yl)-3'-methyl-[1,1'-biphenyl]-4-ol (9)

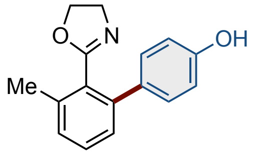

General procedure **A** was followed using 2-(*o*-tolyl)-4,5-dihydrooxazole (80.6 mg, 0.50 mmol) and 4-bromophenol (129.8 mg, 0.75 mmol). Purification by column chromatography on silica gel (*n*-hexane/EtOAc = 1:1) yielded product **9** (65.8 mg, 52%) as a white solid.

^{1}H-NMR (400 MHz, CD_3OD): δ = 7.37 (t, J = 7.7 Hz, 1H), 7.25 – 7.14 (m, 4H), 6.83 – 6.73 (m, 2H), 4.29 (t, J = 9.6 Hz, 2H), 3.83 (t, J = 9.6 Hz, 2H), 2.36 (s, 3H).

^{13}C-NMR (101 MHz, CD_3OD): δ = 168.0 (C_q), 158.2 (C_q), 143.4 (C_q), 138.4 (C_q), 133.3 (C_q), 130.9 (CH), 130.7 (CH), 129.4 (CH), 129.0 (C_q), 128.3 (CH), 116.0 (CH), 68.9 (CH_2), 55.0 (CH_2), 19.7 (CH_3).

IR (ATR): $\tilde{\nu}$ = 1657, 1612, 1516, 1461, 1274, 1253, 1241, 1054, 936, 790 cm^{-1}.
m.p.: 208–209 °C.
HR-MS (ESI): *m*/*z* calcd for $C_{16}H_{16}NO_2^+$ $[M+H]^+$ 254.1176, found 254.1180.

2-(4'-chloro-3-methyl-[1,1'-biphenyl]-2-yl)-4,5-dihydrooxazole (10)

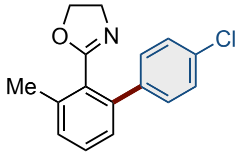

General procedure **A** was followed using 2-(*o*-tolyl)-4,5-dihydrooxazole (80.6 mg, 0.50 mmol) and 1-bromo-4-chlorobenzene (143.6 mg, 0.75 mmol). Purification by column chromatography on silica gel (*n*-hexane/EtOAc = 4:1) yielded product **10** (101.7 mg, 75%) as a colorless oil.

^{1}H-NMR (400 MHz, $CDCl_3$): δ = 7.40 – 7.30 (m, 5H), 7.25 – 7.21 (m, 1H), 7.20 – 7.14 (m, 1H), 4.16 (td, J = 9.5, 0.8 Hz, 2H), 3.87 (td, J = 9.5, 0.8 Hz, 2H), 2.41 (s, 3H).

^{13}C-NMR (101 MHz, $CDCl_3$): δ = 164.3 (C_q), 140.8 (C_q), 139.8 (C_q), 137.8 (C_q), 133.3 (C_q), 129.9 (CH), 129.7 (CH), 129.4 (CH), 128.3 (CH), 128.2 (C_q), 127.2 (CH), 67.3 (CH_2), 55.3 (CH_2), 19.9 (CH_3).

IR (ATR): $\tilde{\nu}$ = 1664, 1494, 1459, 1250, 1092, 1043, 1015, 937, 833, 787 cm^{-1}.
HR-MS (ESI): *m*/*z* calcd for $C_{16}H_{15}NOCl^+$ $[M+H]^+$ 272.0837, found 272.0847.

2-(3,3'-dimethyl-[1,1'-biphenyl]-2-yl)-4,5-dihydrooxazole (11)

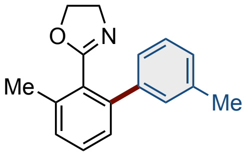

General procedure **A** was followed using 2-(*o*-tolyl)-4,5-dihydrooxazole (80.6 mg, 0.50 mmol) and 1-bromo-3-methylbenzene (128.3 mg, 0.75 mmol). Purification by column chromatography on silica gel (*n*-hexane/EtOAc = 3:1) yielded product **11** (84.9 mg, 68%) as a pale-yellow oil.

^{1}H-NMR (400 MHz, $CDCl_3$): δ = 7.35 (t, *J* = 7.6 Hz, 1H), 7.30 – 7.18 (m, 5H), 7.17 – 7.10 (m, 1H), 4.15 (td, *J* = 9.5, 0.8 Hz, 2H), 3.87 (td, *J* = 9.5, 0.8 Hz, 2H), 2.43 (s, 3H), 2.38 (s, 3H).

^{13}C-NMR (101 MHz, $CDCl_3$): δ = 164.7 (C_q), 142.2 (C_q), 141.2 (C_q), 137.7 (C_q), 137.5 (C_q), 129.5 (CH), 129.3 (CH), 129.0 (CH), 128.2 (C_q), 128.0 (CH), 128.0 (CH), 127.3 (CH), 125.5 (CH), 67.3 (CH_2), 55.3 (CH_2), 21.6 (CH_3), 19.9 (CH_3).

IR (ATR): $\tilde{\nu}$ = 1663, 1658, 1460, 1252, 1235, 1043, 938, 896, 778, 700 cm^{-1}.
HR-MS (ESI): *m*/*z* calcd for $C_{17}H_{18}NO^+$ $[M+H]^+$ 252.1383, found 252.1389.
The characterization data are consistent with those reported in the literature.[7]

2-(3'-methoxy-3-methyl-[1,1'-biphenyl]-2-yl)-4,5-dihydrooxazole (12)

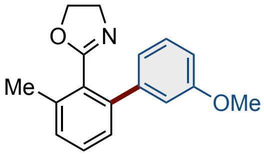

General procedure **A** was followed using 2-(*o*-tolyl)-4,5-dihydrooxazole (80.6 mg, 0.50 mmol) and 1-bromo-3-methoxybenzene (140.3 mg, 0.75 mmol). Purification by column chromatography on silica gel (*n*-hexane/EtOAc = 3:1→1:1) yielded product **12** (82.0 mg, 61%) as a pale-yellow oil.

^{1}H-NMR (400 MHz, $CDCl_3$): δ = 7.40 – 7.32 (m, 1H), 7.31 – 7.25 (m, 1H), 7.25 – 7.19 (m, 2H), 7.04 – 6.96 (m, 2H), 6.87 (ddd, *J* = 8.3, 2.6, 1.0 Hz, 1H), 4.16 (td, *J* = 9.5, 0.8 Hz, 2H), 3.88 (td, *J* = 9.5, 0.8 Hz, 2H), 3.81 (s, 3H), 2.42 (s, 3H).

^{13}C-NMR (101 MHz, $CDCl_3$): δ = 164.6 (C_q), 159.4 (C_q), 142.7 (C_q), 141.9 (C_q), 137.6 (C_q), 129.6 (CH), 129.2 (CH), 129.1 (CH), 128.2 (C_q), 127.3 (CH), 121.0 (CH), 113.8 (CH), 113.3 (CH), 67.4 (CH_2), 55.3 (CH_3), 55.3 (CH_2), 19.9 (CH_3).

IR (ATR): $\tilde{\nu}$ = 1658, 1574, 1468, 1319, 1228, 1215, 1007, 937, 778, 696 cm^{-1}.
HR-MS (ESI): *m*/*z* calcd for $C_{17}H_{18}NO_2^+$ $[M+H]^+$ 268.1332, found 268.1336.
The characterization data are consistent with those reported in the literature.[8]

2-(3-methyl-3'-(trifluoromethyl)-[1,1'-biphenyl]-2-yl)-4,5-dihydrooxazole (13)

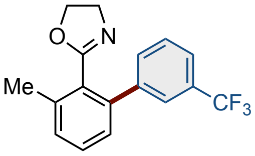

General procedure **A** was followed using 2-(*o*-tolyl)-4,5-dihydrooxazole (80.6 mg, 0.50 mmol) and 1-bromo-3-(trifluoromethyl)benzene (168.8 mg, 0.75 mmol). Purification by column chromatography on silica gel (*n*-hexane/EtOAc = 4:1) yielded product **13** (130.0 mg, 85%) as a pale-yellow oil.

^{1}**H-NMR** (400 MHz, $CDCl_3$): δ = 7.76 – 7.70 (m, 1H), 7.65 – 7.55 (m, 2H), 7.50 (t, J = 7.7 Hz, 1H), 7.39 (t, J = 7.6 Hz, 1H), 7.30 – 7.21 (m, 2H), 4.17 (td, J = 9.6, 0.7 Hz, 2H), 3.86 (td, J = 9.5, 0.7 Hz, 2H), 2.43 (s, 3H).

^{13}**C-NMR** (101 MHz, $CDCl_3$): δ = 164.2 (C_q), 142.0 (C_q), 140.5 (C_q), 138.0 (C_q), 132.0 (q, J_{CF} = 1.4 Hz, CH), 130.4 (q, J_{CF} = 32.2 Hz, C_q), 129.8 (CH), 129.8 (CH), 128.8 (CH), 128.4 (C_q), 127.2 (CH), 125.4 (q, J_{CF} = 3.8 Hz, CH), 124.3 (q, J_{CF} = 272.4 Hz, C_q), 124.0 (q, J_{CF} = 3.9 Hz, CH), 67.4 (CH_2), 55.3 (CH_2), 19.9 (CH_3).

^{19}**F-NMR** (282 MHz, $CDCl_3$): δ = −62.5.

IR (ATR): $\tilde{\nu}$ = 1335, 1276, 1163, 1115, 1096, 1070, 1044, 938, 783, 702 cm^{-1}.
HR-MS (ESI): m/z calcd for $C_{17}H_{15}F_3NO^+$ $[M+H]^+$ 306.1100, found 306.1108.
The characterization data are consistent with those reported in the literature.[7]

2-(2'-methoxy-3-methyl-[1,1'-biphenyl]-2-yl)-4,5-dihydrooxazole (14)

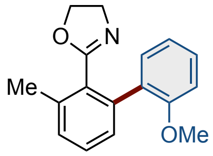

General procedure **A** was followed using 2-(*o*-tolyl)-4,5-dihydrooxazole (80.6 mg, 0.50 mmol) and 1-bromo-2-methoxybenzene (140.3 mg, 0.75 mmol). Purification by column chromatography on silica gel (*n*-hexane/EtOAc = 3:1→1:1) yielded product **14** (60.8 mg, 45%) as a colorless oil.

^{1}**H-NMR** (400 MHz, $CDCl_3$): δ = 7.38 – 7.26 (m, 2H), 7.25 – 7.16 (m, 3H), 7.00 – 6.89 (m, 2H), 4.05 (t, J = 9.5 Hz, 2H), 3.83 – 3.76 (m, 2H), 3.75 (s, 3H), 2.43 (s, 3H).

^{13}**C-NMR** (101 MHz, $CDCl_3$): δ = 164.6 (C_q), 156.6 (C_q), 138.8 (C_q), 137.3 (C_q), 130.8 (CH), 130.3 (C_q), 129.2 (CH), 129.1 (CH), 129.0 (C_q), 128.7 (CH), 128.3 (CH), 120.2 (CH), 110.8 (CH), 67.1 (CH_2), 55.7 (CH_3), 55.2 (CH_2), 20.3 (CH_3).

IR (ATR): $\tilde{\nu}$ = 1658, 1497, 1467, 1451, 1252, 1238, 1042, 1025, 939, 755 cm^{-1}.
HR-MS (ESI): m/z calcd for $C_{17}H_{18}NO_2^+$ $[M+H]^+$ 268.1332, found 268.1337.
The characterization data are consistent with those reported in the literature.[7]

2-(3,3',5'-trimethyl-[1,1'-biphenyl]-2-yl)-4,5-dihydrooxazole (15)

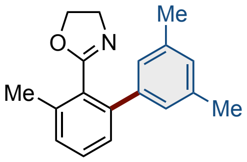

General procedure **A** was followed using 2-(*o*-tolyl)-4,5-dihydrooxazole (80.6 mg, 0.50 mmol) and 1-bromo-3,5-dimethylbenzene (138.8 mg, 0.75 mmol). Purification by column chromatography on silica gel (*n*-hexane/EtOAc = 3:1) yielded product **15** (101.7 mg, 77%) as a colorless oil. General procedure **B** afforded product **15** in 54% yield.

^{1}**H-NMR** (400 MHz, $CDCl_3$): δ = 7.34 (t, J = 7.6 Hz, 1H), 7.25 – 7.17 (m, 2H), 7.09 – 7.04 (m, 2H), 6.99 – 6.93 (m, 1H), 4.17 (td, J = 9.4, 0.8 Hz, 2H), 3.88 (td, J = 9.5, 0.8 Hz, 2H), 2.42 (s, 3H), 2.34 (s, 6H).

^{13}**C-NMR** (101 MHz, $CDCl_3$): δ = 164.7 (C_q), 142.2 (C_q), 141.1 (C_q), 137.5 (C_q), 137.5 (C_q), 129.5 (CH), 128.9 (CH), 128.9 (CH), 128.2 (C_q), 127.3 (CH), 126.4 (CH), 67.3 (CH_2), 55.3 (CH_2), 21.5 (CH_3), 19.9 (CH_3).

IR (ATR): $\tilde{\nu}$ = 1663, 1603, 1462, 1252, 1235, 1044, 939, 852, 789, 700 cm^{-1}.
HR-MS (ESI): m/z calcd for $C_{18}H_{20}NO^+$ $[M+H]^+$ 266.1539, found 266.1542.

2-(3-methyl-[1,1'-biphenyl]-2-yl)-4,5-dihydrooxazole (16)

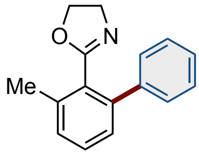

General procedure **A** was followed using 2-(*o*-tolyl)-4,5-dihydrooxazole (80.6 mg, 0.50 mmol) and bromobenzene (117.8 mg, 0.75 mmol). Purification by column chromatography on silica gel (*n*-hexane/EtOAc = 2:1) yielded product **16** (90.3 mg, 76%) as a colorless oil.

^{1}H-NMR (400 MHz, $CDCl_3$): δ = 7.46 – 7.28 (m, 6H), 7.25 – 7.20 (m, 2H), 4.13 (td, J = 9.5, 0.8 Hz, 2H), 3.86 (td, J = 9.4, 0.8 Hz, 2H), 2.43 (s, 3H).

^{13}C-NMR (101 MHz, $CDCl_3$): δ = 164.6 (C_q), 142.1 (C_q), 141.3 (C_q), 137.6 (C_q), 129.6 (CH), 129.1 (CH), 128.5 (CH), 128.3 (C_q), 128.1 (CH), 127.3 (CH), 127.2 (CH), 67.3 (CH_2), 55.2 (CH_2), 19.9 (CH_3).

IR (ATR): $\tilde{\nu}$ = 1659, 1461, 1251, 1235, 1042, 937, 896, 796, 761, 699 cm^{-1}.
HR-MS (ESI): *m*/*z* calcd for $C_{16}H_{16}NO^+$ $[M+H]^+$ 238.1226, found 238.1236.
The characterization data are consistent with those reported in the literature.[10]

2-(2-(9*H*-fluoren-2-yl)-6-methylphenyl)-4,5-dihydrooxazole (17)

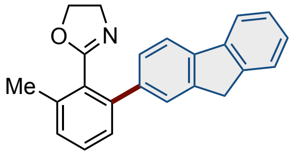

General procedure **A** was followed using 2-(*o*-tolyl)-4,5-dihydrooxazole (80.6 mg, 0.50 mmol) and 2-bromo-9*H*-fluorene (183.8 mg, 0.75 mmol). Purification by column chromatography on silica gel (*n*-hexane/EtOAc = 3:1) yielded product **17** (137.5 mg, 85%) as a white solid.

^{1}H-NMR (400 MHz, $CDCl_3$): δ = 7.84 – 7.75 (m, 2H), 7.63 – 7.59 (m, 1H), 7.58 – 7.53 (m, 1H), 7.48 – 7.35 (m, 3H), 7.34 – 7.27 (m, 2H), 7.25 – 7.21 (m, 1H), 4.14 (td, J = 9.5, 0.8 Hz, 2H), 3.93 (s, 2H), 3.86 (td, J = 9.4, 0.8 Hz, 2H), 2.44 (s, 3H).

^{13}C-NMR (101 MHz, $CDCl_3$): δ = 164.7 (C_q), 143.6 (C_q), 143.3 (C_q), 142.4 (C_q), 141.6 (C_q), 140.8 (C_q), 140.0 (C_q), 137.7 (C_q), 129.6 (CH), 129.0 (CH), 128.4 (C_q), 127.4 (CH), 127.3 (CH), 126.9 (CH), 126.8 (CH), 125.3 (CH), 125.2 (CH), 120.0 (CH), 119.5 (CH), 67.3 (CH_2), 55.3 (CH_2), 37.1 (CH_2), 20.0 (CH_3).

IR (ATR): $\tilde{\nu}$ = 1664, 1469, 1452, 1251, 1235, 1044, 938, 770, 755, 736 cm^{-1}.
m.p.: 140–141 °C.
HR-MS (ESI): *m*/*z* calcd for $C_{23}H_{19}NONa^+$ $[M+Na]^+$ 348.1359, found 348.1367.

2-(2-(dibenzo[*b*,*d*]furan-2-yl)-6-methylphenyl)-4,5-dihydrooxazole (18)

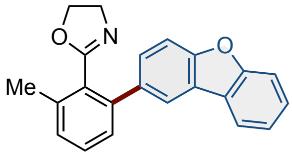

General procedure **A** was followed using 2-(*o*-tolyl)-4,5-dihydrooxazole (80.6 mg, 0.50 mmol) and 2-bromodibenzo[*b*,*d*]furan (185.3 mg, 0.75 mmol). Purification by column chromatography on silica gel (*n*-hexane/EtOAc = 4:1) yielded product **18** (79.2 mg, 48%) as a colorless oil.

^{1}H-NMR (400 MHz, $CDCl_3$): δ = 8.04 – 7.99 (m, 1H), 7.97 – 7.90 (m, 1H), 7.62 – 7.54 (m, 2H), 7.51 (dd, J = 8.5, 1.7 Hz, 1H), 7.47 (ddd, J = 8.4, 7.4, 1.3 Hz, 1H), 7.40 (t, J = 7.6 Hz, 1H), 7.35 (td, J = 7.5, 1.0 Hz, 1H), 7.33 – 7.29 (m, 1H), 7.28 – 7.23 (m, 1H), 4.10 (td, J = 9.5, 0.8 Hz, 2H), 3.83 (td, J = 9.4, 0.8 Hz, 2H), 2.45 (s, 3H).

^{13}C-NMR (101 MHz, $CDCl_3$): δ = 164.7 (C_q), 156.7 (C_q), 155.7 (C_q), 142.0 (C_q), 137.7 (C_q), 136.3 (C_q), 129.6 (CH), 129.1 (CH), 128.7 (C_q), 127.9 (CH), 127.7 (CH), 127.4 (CH), 124.4 (C_q), 124.2 (C_q), 122.9 (CH), 120.7 (CH), 120.7 (CH), 111.9 (CH), 111.2 (CH), 67.4 (CH_2), 55.3 (CH_2), 20.0 (CH_3).

IR (ATR): $\tilde{\nu}$ = 1665, 1461, 1447, 1197, 1188, 1043, 937, 791, 748, 730 cm^{-1}.
HR-MS (ESI): *m*/*z* calcd for $C_{22}H_{18}NO_2^+$ [M+H]$^+$ 328.1332, found 328.1335.

2-(2-(benzo[*d*][1,3]dioxol-5-yl)-6-methylphenyl)-4,5-dihydrooxazole (19)

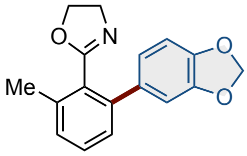

General procedure **A** was followed using 2-(*o*-tolyl)-4,5-dihydrooxazole (80.6 mg, 0.50 mmol) and 5-bromobenzo[*d*][1,3]dioxole (150.8 mg, 0.75 mmol). Purification by column chromatography on silica gel (*n*-hexane/EtOAc = 3:1) yielded product **19** (96.3 mg, 68%) as a pale-yellow oil.

^{1}H-NMR (400 MHz, $CDCl_3$): δ = 7.32 (t, J = 7.6 Hz, 1H), 7.23 – 7.14 (m, 2H), 6.94 – 6.85 (m, 2H), 6.81 (d, J = 8.0 Hz, 1H), 5.97 (s, 2H), 4.20 (td, J = 9.5, 0.7 Hz, 2H), 3.90 (td, J = 9.4, 0.7 Hz, 2H), 2.40 (s, 3H).

^{13}C-NMR (101 MHz, $CDCl_3$): δ = 164.6 (C_q), 147.5 (C_q), 146.9 (C_q), 141.6 (C_q), 137.6 (C_q), 135.3 (C_q), 129.6 (CH), 128.9 (CH), 128.3 (C_q), 127.3 (CH), 122.0 (CH), 109.2 (CH), 108.1 (CH), 101.1 (CH_2), 67.3 (CH_2), 55.3 (CH_2), 19.9 (CH_3).

IR (ATR): $\tilde{\nu}$ = 1663, 1505, 1491, 1462, 1249, 1226, 1035, 938, 787, 758 cm^{-1}.
HR-MS (ESI): *m*/*z* calcd for $C_{17}H_{16}NO_3^+$ [M+H]$^+$ 282.1125, found 282.1129.

2-(2-(2,2-difluorobenzo[*d*][1,3]dioxol-5-yl)-6-methylphenyl)-4,5-dihydrooxazole (20)

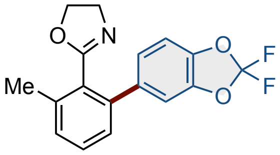

General procedure **A** was followed using 2-(*o*-tolyl)-4,5-dihydrooxazole (80.6 mg, 0.50 mmol) and 5-bromo-2,2-difluorobenzo[*d*][1,3]dioxole (177.8 mg, 0.75 mmol). Purification by column chromatography on silica gel (*n*-hexane/EtOAc = 3:1) yielded product **20** (101.3 mg, 64%) as a colorless semisolid.

^{1}H-NMR (400 MHz, $CDCl_3$): δ = 7.36 (t, J = 7.7 Hz, 1H), 7.27 – 7.22 (m, 1H), 7.19 – 7.09 (m, 3H), 7.07 – 7.03 (m, 1H), 4.20 (td, J = 9.5, 0.7 Hz, 2H), 3.89 (td, J = 9.5, 0.7 Hz, 2H), 2.41 (s, 3H).

^{13}C-NMR (101 MHz, $CDCl_3$): δ = 164.3 (C_q), 143.7 (C_q), 143.1 (C_q), 140.6 (C_q), 138.0 (C_q), 137.5 (C_q), 131.8 (t, J_{CF} = 255.1 Hz, C_q), 129.8 (CH), 129.6 (CH), 128.4 (C_q), 127.3 (CH), 124.0 (CH), 110.0 (CH), 109.0 (CH), 67.4 (CH_2), 55.3 (CH_2), 19.9 (CH_3).

^{19}F-NMR (282 MHz, $CDCl_3$): δ = −49.9.

IR (ATR): $\tilde{\nu}$ = 1664, 1500, 1466, 1235, 1215, 1148, 1128, 1045, 1033, 939 cm^{-1}.
HR-MS (ESI): *m*/*z* calcd for $C_{17}H_{14}F_2NO_3^+$ [M+H]$^+$ 318.0936, found 318.0932.

2-(2-(1*H*-indol-5-yl)-6-methylphenyl)-4,5-dihydrooxazole (21)

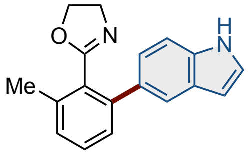

General procedure **A** was followed using 2-(*o*-tolyl)-4,5-dihydrooxazole (80.6 mg, 0.50 mmol) and 5-bromo-1*H*-indole (147.0 mg, 0.75 mmol). Purification by column chromatography on silica gel (*n*-hexane/EtOAc = 1:1) yielded product **21** (107.8 mg, 78%) as a brown solid.

^{1}**H-NMR** (400 MHz, DMSO-d_6): δ = 11.12 (s, 1H), 7.57 – 7.53 (m, 1H), 7.44 – 7.33 (m, 3H), 7.31 – 7.21 (m, 2H), 7.12 (dd, *J* = 8.5, 1.7 Hz, 1H), 6.46 (ddd, *J* = 2.9, 1.8, 0.7 Hz, 1H), 4.12 (t, *J* = 9.4 Hz, 2H), 3.71 (t, *J* = 9.4 Hz, 2H), 2.32 (s, 3H).

^{13}**C-NMR** (101 MHz, DMSO-d_6): δ = 163.4 (C_q), 142.6 (C_q), 136.8 (C_q), 135.1 (C_q), 131.4 (C_q), 129.4 (CH), 128.4 (C_q), 127.9 (CH), 127.7 (C_q), 127.5 (CH), 125.8 (CH), 121.6 (CH), 119.6 (CH), 111.0 (CH), 101.4 (CH), 66.7 (CH_2), 54.7 (CH_2), 19.4 (CH_3).

IR (ATR): $\tilde{\nu}$ = 1650, 1455, 1344, 1253, 1049, 936, 791, 768, 728, 711 cm^{-1}.
m.p.: >250 °C.
HR-MS (ESI): *m*/*z* calcd for $C_{18}H_{16}N_2ONa^+$ [M+Na]$^+$ 299.1155, found 299.1154.

2-(2-(1*H*-indol-6-yl)-6-methylphenyl)-4,5-dihydrooxazole (22)

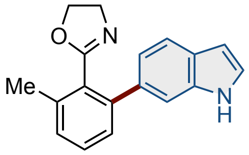

General procedure **A** was followed using 2-(*o*-tolyl)-4,5-dihydrooxazole (80.6 mg, 0.50 mmol) and 6-bromo-1*H*-indole (147.0 mg, 0.75 mmol). Purification by column chromatography on silica gel (*n*-hexane/EtOAc = 1:1) yielded product **22** (111.2 mg, 80%) as a brown solid.

^{1}**H-NMR** (400 MHz, $CDCl_3$): δ = 9.12 (s, 1H), 7.59 (d, *J* = 8.2 Hz, 1H), 7.35 (t, *J* = 7.6 Hz, 1H), 7.26 – 7.20 (m, 3H), 7.15 (dd, *J* = 8.2, 1.6 Hz, 1H), 6.97 (dd, *J* = 3.1, 2.4 Hz, 1H), 6.47 (ddd, *J* = 3.0, 1.9, 0.8 Hz, 1H), 4.14 (t, *J* = 9.5 Hz, 2H), 3.82 (t, *J* = 9.4 Hz, 2H), 2.46 (s, 3H).

^{13}**C-NMR** (101 MHz, $CDCl_3$): δ = 165.4 (C_q), 143.4 (C_q), 137.3 (C_q), 136.0 (C_q), 135.0 (C_q), 129.6 (CH), 128.5 (CH), 128.4 (C_q), 127.9 (CH), 127.0 (C_q), 124.8 (CH), 120.7 (CH), 120.0 (CH), 111.4 (CH), 101.9 (CH), 67.4 (CH_2), 54.9 (CH_2), 20.0 (CH_3).

IR (ATR): $\tilde{\nu}$ = 1658, 1453, 1349, 1253, 1049, 936, 792, 774, 755, 726 cm^{-1}.
m.p.: 171–172 °C.
HR-MS (ESI): *m*/*z* calcd for $C_{18}H_{17}N_2O^+$ [M+H]$^+$ 277.1335, found 277.1337.

dimethyl 2'-(4,5-dihydrooxazol-2-yl)-[1,1':3',1''-terphenyl]-4,4''-dicarboxylate (23)

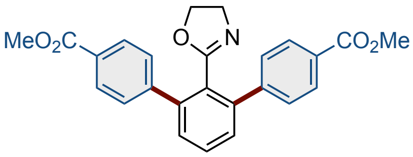

General procedure **A** was followed using 2-phenyl-4,5-dihydrooxazole (73.6 mg, 0.50 mmol) and methyl 4-bromobenzoate (322.6 mg, 1.5 mmol). Purification by column chromatography on silica gel (*n*-hexane/EtOAc = 1:1) yielded product **23** (186.0 mg, 90%) as a white solid.

^{1}**H-NMR** (400 MHz, $CDCl_3$): δ = 8.10 – 8.03 (m, 4H), 7.57 (dd, J = 8.2, 7.2 Hz, 1H), 7.53 – 7.48 (m, 4H), 7.45 – 7.40 (m, 2H), 3.94 (s, 6H), 3.89 (t, J = 9.5 Hz, 2H), 3.58 (t, J = 9.5 Hz, 2H).

^{13}**C-NMR** (101 MHz, $CDCl_3$): δ = 167.1 (C_q), 163.5 (C_q), 145.5 (C_q), 141.6 (C_q), 130.0 (CH), 129.5 (CH), 129.4 (CH), 129.3 (C_q), 128.7 (CH), 127.6 (C_q), 67.6 (CH_2), 55.2 (CH_2), 52.3 (CH_3).

IR (ATR): $\tilde{\nu}$ = 1727, 1710, 1665, 1312, 1290, 1279, 1247, 1121, 1042, 769 cm^{-1}.
m.p.: 226–227 °C.
HR-MS (ESI): *m/z* calcd for $C_{25}H_{22}NO_5^+$ $[M+H]^+$ 416.1492, found 416.1487.
The characterization data are consistent with those reported in the literature.[11]

methyl 2'-(4,5-dihydrooxazol-2-yl)-3'-fluoro-[1,1'-biphenyl]-4-carboxylate (24)

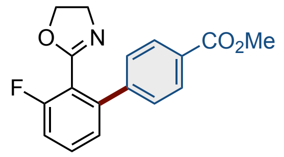

General procedure **A** was followed using 2-(2-fluorophenyl)-4,5-dihydrooxazole (82.6 mg, 0.50 mmol) and methyl 4-bromobenzoate (161.3 mg, 0.75 mmol). Purification by column chromatography on silica gel (*n*-hexane/EtOAc = 2:1→1:1) yielded product **24** (104.7 mg, 70%) as a gray solid. General procedure **B** afforded product **24** in 45% yield.

^{1}**H-NMR** (400 MHz, $CDCl_3$): δ = 8.10 – 8.02 (m, 2H), 7.54 – 7.41 (m, 3H), 7.24 – 7.11 (m, 2H), 4.18 (t, J = 9.6 Hz, 2H), 3.97 – 3.85 (m, 5H).

^{13}**C-NMR** (101 MHz, $CDCl_3$): δ = 167.0 (C_q), 161.0 (d, J_{CF} = 251.9 Hz, C_q), 160.3 (C_q), 144.3 (d, J_{CF} = 2.3 Hz, C_q), 143.0 (d, J_{CF} = 2.3 Hz, C_q), 131.6 (d, J_{CF} = 9.1 Hz, CH), 129.6 (CH), 129.5 (C_q), 128.5 (CH), 125.5 (d, J_{CF} = 3.2 Hz, CH), 116.9 (d, J_{CF} = 15.7 Hz, C_q), 115.4 (d, J_{CF} = 21.8 Hz, CH), 67.7 (CH_2), 55.4 (CH_2), 52.3 (CH_3).

^{19}**F-NMR** (282 MHz, $CDCl_3$): δ = −112.8.

IR (ATR): $\tilde{\nu}$ = 1723, 1666, 1272, 1238, 1096, 1044, 937, 771, 744, 711 cm^{-1}.
m.p.: 121–122 °C.
HR-MS (ESI): *m/z* calcd for $C_{17}H_{15}FNO_3^+$ $[M+H]^+$ 300.1030, found 300.1030.

dimethyl 5'-chloro-2'-(4,5-dihydrooxazol-2-yl)-[1,1':3',1''-terphenyl]-4,4''-dicarboxylate (25)

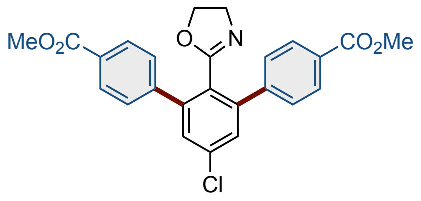

General procedure **A** was followed using 2-(4-chlorophenyl)-4,5-dihydrooxazole (90.8 mg, 0.50 mmol) and methyl 4-bromobenzoate (322.6 mg, 1.5 mmol). Purification by column chromatography on silica gel (*n*-hexane/EtOAc = 1:1) yielded product **25** (183.1 mg, 81%) as a dark green solid.

^{1}**H-NMR** (400 MHz, $CDCl_3$): δ = 8.11 – 8.02 (m, 4H), 7.53 – 7.45 (m, 4H), 7.42 (s, 2H), 3.93 (s, 6H), 3.87 (t, J = 9.5 Hz, 2H), 3.56 (t, J = 9.5 Hz, 2H).

^{13}**C-NMR** (101 MHz, $CDCl_3$): δ = 166.9 (C_q), 162.8 (C_q), 144.1 (C_q), 143.3 (C_q), 135.8 (C_q), 129.7 (C_q), 129.6 (CH), 129.2 (CH), 128.6 (CH), 126.2 (C_q), 67.7 (CH_2), 55.2 (CH_2), 52.3 (CH_3).

IR (ATR): $\tilde{\nu}$ = 1722, 1665, 1435, 1291, 1274, 1113, 1103, 1034, 772, 752 cm^{-1}.

m.p.: 218–219 °C.
HR-MS (ESI): *m*/*z* calcd for $C_{25}H_{21}ClNO_5^+$ $[M+H]^+$ 450.1103, found 450.1118.

dimethyl 2'-(pyridin-2-yl)-[1,1':3',1''-terphenyl]-4,4''-dicarboxylate (26)

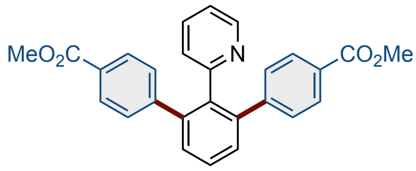

General procedure **A** was followed using 2-phenylpyridine (77.6 mg, 0.50 mmol) and methyl 4-bromobenzoate (322.6 mg, 1.5 mmol). Purification by column chromatography on silica gel (*n*-hexane/EtOAc = 5:1→2:1) yielded product **26** (158.5 mg, 75%) as a white solid.

^{1}H-NMR (400 MHz, $CDCl_3$): δ = 8.29 (ddd, *J* = 4.9, 1.8, 1.0 Hz, 1H), 7.87 – 7.79 (m, 4H), 7.56 (dd, *J* = 8.5, 6.7 Hz, 1H), 7.51 – 7.44 (m, 2H), 7.30 (td, *J* = 7.7, 1.8 Hz, 1H), 7.19 – 7.12 (m, 4H), 6.93 (ddd, *J* = 7.6, 4.9, 1.2 Hz, 1H), 6.85 (dt, *J* = 7.8, 1.1 Hz, 1H), 3.87 (s, 6H).

^{13}C-NMR (101 MHz, $CDCl_3$): δ = 167.1 (C_q), 158.1 (C_q), 148.9 (CH), 146.3 (C_q), 141.2 (C_q), 138.6 (C_q), 135.4 (CH), 130.0 (CH), 129.7 (CH), 129.2 (CH), 128.6 (CH), 128.3 (C_q), 126.8 (CH), 121.5 (CH), 52.2 (CH_3).

IR (ATR): $\tilde{\nu}$ = 1721, 1608, 1435, 1274, 1179, 1122, 1101, 770, 749, 706 cm^{-1}.
m.p.: 195–196 °C.
HR-MS (ESI): *m*/*z* calcd for $C_{27}H_{22}NO_4^+$ $[M+H]^+$ 424.1543, found 424.1539.
The characterization data are consistent with those reported in the literature.[12]

methyl 3'-methyl-2'-(1*H*-pyrazol-1-yl)-[1,1'-biphenyl]-4-carboxylate (27)

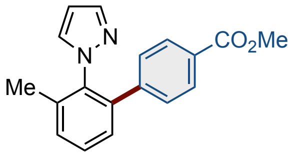

General procedure **A** was followed using 1-(*o*-tolyl)-1*H*-pyrazole (79.1 mg, 0.50 mmol) and methyl 4-bromobenzoate (161.3 mg, 0.75 mmol). Purification by column chromatography on silica gel (*n*-hexane/EtOAc = 10:1→5:1) yielded product **27** (101.8 mg, 70%) as a white solid.

^{1}H-NMR (400 MHz, $CDCl_3$): δ = 7.91 – 7.84 (m, 2H), 7.63 (d, *J* = 2.0 Hz, 1H), 7.42 (t, *J* = 7.6 Hz, 1H), 7.38 – 7.29 (m, 2H), 7.19 – 7.12 (m, 2H), 7.08 (d, *J* = 2.1 Hz, 1H), 6.18 (t, *J* = 2.1 Hz, 1H), 3.88 (s, 3H), 2.14 (s, 3H).

^{13}C-NMR (101 MHz, $CDCl_3$): δ = 167.0 (C_q), 143.5 (C_q), 140.0 (CH), 139.0 (C_q), 137.9 (C_q), 137.3 (C_q), 131.6 (CH), 130.8 (CH), 129.4 (CH), 129.3 (CH), 128.9 (CH), 128.4 (C_q), 128.1 (CH), 106.3 (CH), 52.2 (CH_3), 17.8 (CH_3).

IR (ATR): $\tilde{\nu}$ = 1722, 1434, 1401, 1276, 1114, 1096, 938, 770, 748, 707 cm^{-1}.
m.p.: 111–112 °C.
HR-MS (ESI): *m*/*z* calcd for $C_{18}H_{17}N_2O_2^+$ $[M+H]^+$ 293.1285, found 293.1291.
The characterization data are consistent with those reported in the literature.[13]

isopropyl 2-(4-(2'-(4,5-dihydrooxazol-2-yl)-3'-methyl-[1,1'-biphenyl]-4-carbonyl)phenoxy)-2-methylpropanoate (28)

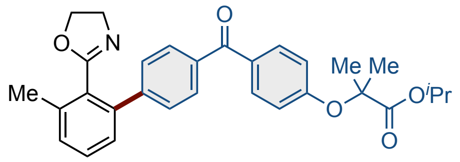

General procedure **A** was followed using 2-(*o*-tolyl)-4,5-dihydrooxazole (80.6 mg, 0.50 mmol) and isopropyl 2-(4-(4-chlorobenzoyl)phenoxy)-2-methylpropanoate (270.6 mg, 0.75 mmol). Purification by column chromatography on silica gel (*n*-hexane/EtOAc = 2:1→1:1) yielded product **28** (146.1 mg, 60%) as a colorless oil.

^{1}H-NMR (400 MHz, $CDCl_3$): δ = 7.81 – 7.74 (m, 4H), 7.55 – 7.48 (m, 2H), 7.39 (t, J = 7.7 Hz, 1H), 7.30 – 7.21 (m, 2H), 6.92 – 6.84 (m, 2H), 5.09 (hept, J = 6.3 Hz, 1H), 4.17 (t, J = 9.5 Hz, 2H), 3.88 (t, J = 9.5 Hz, 2H), 2.43 (s, 3H), 1.67 (s, 6H), 1.21 (d, J = 6.3 Hz, 6H).

^{13}C-NMR (101 MHz, $CDCl_3$): δ = 195.4 (C_q), 173.3 (C_q), 164.3 (C_q), 159.7 (C_q), 145.3 (C_q), 141.1 (C_q), 138.0 (C_q), 136.8 (C_q), 132.1 (CH), 130.9 (C_q), 129.8 (CH), 129.8 (CH), 129.8 (CH), 128.4 (CH), 128.3 (C_q), 127.3 (CH), 117.3 (CH), 79.5 (C_q), 69.5 (CH), 67.4 (CH_2), 55.4 (CH_2), 25.5 (CH_3), 21.7 (CH_3), 20.0 (CH_3).

IR (ATR): $\tilde{v}$ = 1651, 1599, 1289, 1275, 1251, 1176, 1147, 1102, 930, 769 cm^{-1}.
HR-MS (ESI): *m*/*z* calcd for $C_{30}H_{32}NO_5^+$ [M+H]$^+$ 486.2275, found 486.2286.

isopropyl (2'-(4,5-dihydrooxazol-2-yl)-3'-methyl-[1,1'-biphenyl]-3-yl)carbamate (29)

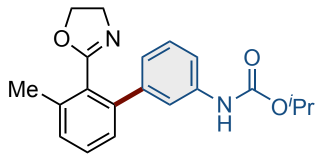

General procedure **A** was followed using 2-(*o*-tolyl)-4,5-dihydrooxazole (80.6 mg, 0.50 mmol) and isopropyl (3-chlorophenyl)carbamate (160.2 mg, 0.75 mmol). Purification by column chromatography on silica gel (*n*-hexane/EtOAc = 2:1→1:1) yielded product **29** (119.0 mg, 70%) as a colorless oil.

^{1}H-NMR (400 MHz, $CDCl_3$): δ = 7.41 – 7.34 (m, 1H), 7.32 (t, J = 7.6 Hz, 1H), 7.25 – 7.11 (m, 5H), 7.09 – 7.02 (m, 1H), 4.99 (hept, J = 6.3 Hz, 1H), 4.19 (t, J = 9.5 Hz, 2H), 3.87 (t, J = 9.5 Hz, 2H), 2.40 (s, 3H), 1.26 (d, J = 6.2 Hz, 6H).

^{13}C-NMR (101 MHz, $CDCl_3$): δ = 164.6 (C_q), 153.4 (C_q), 141.9 (C_q), 141.7 (C_q), 138.3 (C_q), 137.5 (C_q), 129.6 (CH), 129.1 (CH), 128.6 (CH), 128.1 (C_q), 127.3 (CH), 123.2 (CH), 118.9 (CH), 117.5 (CH), 68.5 (CH), 67.4 (CH_2), 55.1 (CH_2), 22.2 (CH_3), 19.9 (CH_3).

IR (ATR): $\tilde{v}$ = 1725, 1658, 1556, 1252, 1243, 1218, 1112, 1046, 783, 753 cm^{-1}.
HR-MS (ESI): *m*/*z* calcd for $C_{20}H_{23}N_2O_3^+$ [M+H]$^+$ 339.1703, found 339.1707.

4-(4-(2'-(4,5-dihydrooxazol-2-yl)-3'-methyl-[1,1'-biphenyl]-4-yl)-4-hydroxypiperidin-1-yl)-1-(4-fluorophenyl)butan-1-one (30)

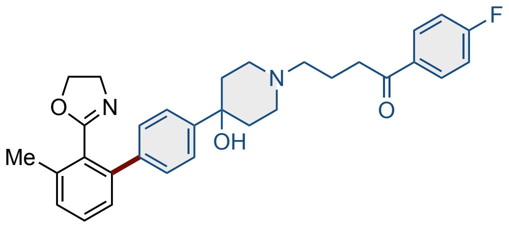

General procedure **A** was followed using 2-(*o*-tolyl)-4,5-dihydrooxazole (80.6 mg, 0.50 mmol) and 4-(4-(4-chlorophenyl)-4-hydroxypiperidin-1-yl)-1-(4-fluorophenyl)butan-1-one (281.9 mg, 0.75 mmol). Purification by column chromatography on silica gel (DCM/MeOH = 20:1→10:1) yielded product **30** (145.4 mg, 58%) as a brown oil.

^{1}H-NMR (400 MHz, $CDCl_3$): δ = 8.07 – 7.97 (m, 2H), 7.46 – 7.39 (m, 2H), 7.39 – 7.30 (m, 3H), 7.25 – 7.16 (m, 2H), 7.19 – 7.08 (m, 2H), 4.16 (t, J = 9.5 Hz, 2H), 3.83 (t, J = 9.5 Hz, 2H), 3.06 (t, J = 6.8 Hz, 2H), 3.01 – 2.86 (m, 2H), 2.77 – 2.54 (m, 4H), 2.40 (s, 3H), 2.35 – 2.17 (m, 2H), 2.09 (quint, J = 6.9 Hz, 2H), 1.79 – 1.68 (m, 2H).

^{13}C-NMR (101 MHz, $CDCl_3$): δ = 198.1 (C_q), 165.9 (d, J_{CF} = 254.7 Hz, C_q), 164.8 (C_q), 146.7 (C_q), 141.6 (C_q), 139.9 (C_q), 137.7 (C_q), 133.6 (d, J_{CF} = 3.0 Hz, C_q), 130.9 (d, J_{CF} = 9.2 Hz, CH), 129.7 (CH), 129.1 (CH), 128.5 (CH), 128.1 (C_q), 127.4 (CH), 124.4 (CH), 115.8 (d, J_{CF} = 21.8 Hz, CH), 70.7 (C_q), 67.4 (CH_2), 57.6 (CH_2), 55.1 (CH_2), 49.4 (CH_2), 37.6 (CH_2), 36.2 (CH_2), 21.0 (CH_2), 19.9 (CH_3).

^{19}F-NMR (282 MHz, $CDCl_3$): δ = −105.3.

IR (ATR): $\tilde{\nu}$ = 1650, 1598, 1251, 1176, 1146, 1101, 1044, 929, 769, 746 cm^{-1}.
HR-MS (ESI): m/z calcd for $C_{31}H_{34}N_2O_3F^+$ [M+H]$^+$ 501.2548, found 501.2557.

(3*R*,4*S*)-4-(2'-(4,5-dihydrooxazol-2-yl)-3'-methyl-[1,1'-biphenyl]-4-yl)-1-(4-fluorophenyl)-3-((*S*)-3-(4-fluorophenyl)-3-hydroxypropyl)azetidin-2-one (31)

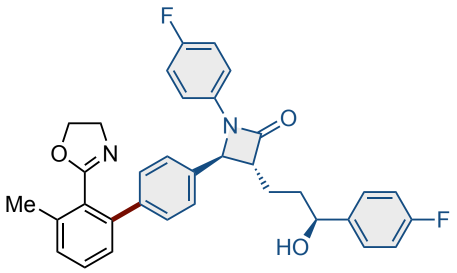

General procedure **A** was followed using 2-(*o*-tolyl)-4,5-dihydrooxazole (80.6 mg, 0.50 mmol) and 4-((2*S*,3*R*)-1-(4-fluorophenyl)-3-((*S*)-3-(4-fluorophenyl)-3-hydroxypropyl)-4-oxoazetidin-2-yl)phenyl trifluoromethanesulfonate (406.1 mg, 0.75 mmol). Purification by column chromatography on silica gel (*n*-hexane/EtOAc = 1:2→1:3) yielded product **31** (85.2 mg, 31%) as a colorless semisolid.

^{1}H-NMR (400 MHz, $CDCl_3$): δ = 7.42 – 7.36 (m, 2H), 7.35 – 7.21 (m, 8H), 7.20 – 7.16 (m, 1H), 7.03 – 6.96 (m, 2H), 6.95 – 6.89 (m, 2H), 4.71 – 4.64 (m, 1H), 4.63 (d, J = 2.4 Hz, 1H), 4.14 – 3.96 (m, 2H), 3.86 – 3.65 (m, 2H), 3.12 (td, J = 7.9, 2.4 Hz, 1H), 2.96 – 2.82 (m, 1H), 2.39 (s, 3H), 2.07 – 1.86 (m, 4H).

^{13}C-NMR (101 MHz, $CDCl_3$): δ = 167.6 (C_q), 164.6 (C_q), 162.2 (d, J_{CF} = 245.3 Hz, C_q), 159.1 (d, J_{CF} = 243.4 Hz, C_q), 141.8 (C_q), 141.2 (C_q), 140.4 (d, J_{CF} = 3.2 Hz, C_q), 137.8 (C_q), 136.4 (C_q), 134.0 (d, J_{CF} = 2.6 Hz, C_q), 129.8 (CH), 129.5 (CH), 129.4 (CH), 128.1 (C_q), 127.5 (d, J_{CF} = 8.0 Hz, CH), 127.2 (CH), 125.7 (CH), 118.5 (d, J_{CF} = 7.7 Hz, CH), 115.9 (d, J_{CF} = 22.8 Hz, CH), 115.4 (d, J_{CF} = 21.4 Hz, CH), 73.0 (CH), 67.3 (CH_2), 61.4 (CH), 60.4 (CH), 55.1 (CH_2), 36.8 (CH_2), 25.2 (CH_2), 19.9 (CH_3).

^{19}F-NMR (282 MHz, $CDCl_3$): δ = −115.0, −117.9.

IR (ATR): $\tilde{\nu}$ = 1747, 1658, 1509, 1385, 1349, 1218, 1156, 1062, 834, 753 cm^{-1}.
HR-MS (ESI): m/z calcd for $C_{34}H_{31}F_2N_2O_3^+$ [M+H]$^+$ 553.2297, found 553.2276.

2-(2-((*R*)-2,8-dimethyl-2-((4*R*,8*R*)-4,8,12-trimethyltridecyl)chroman-6-yl)-6-methylphenyl)-4,5-dihydrooxazole (32)

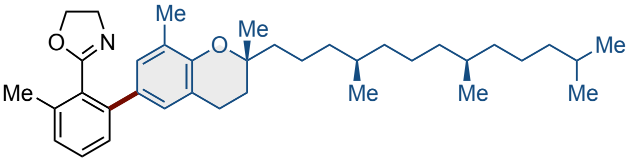

General procedure **A** was followed using 2-(*o*-tolyl)-4,5-dihydrooxazole (80.6 mg, 0.50 mmol) and (*R*)-2,8-dimethyl-2-((4*R*,8*R*)-4,8,12-trimethyltridecyl)chroman-6-yl trifluoromethanesulfonate (401.0 mg, 0.75 mmol). Purification by column chromatography on silica gel (*n*-hexane/EtOAc = 10:1→5:1) yielded product **32** (114.4 mg, 42%) as a colorless oil.

^{1}H-NMR (400 MHz, $CDCl_3$): δ = 7.31 (t, *J* = 7.6 Hz, 1H), 7.25 – 7.18 (m, 1H), 7.19 – 7.12 (m, 1H), 7.04 (d, *J* = 1.7 Hz, 1H), 6.98 (d, *J* = 1.7 Hz, 1H), 4.18 (t, *J* = 9.5 Hz, 2H), 3.89 (t, *J* = 9.5 Hz, 2H), 2.82 – 2.69 (m, 2H), 2.40 (s, 3H), 2.17 (s, 3H), 1.89 – 1.71 (m, 2H), 1.65 – 0.99 (m, 24H), 0.89 – 0.83 (m, 12H).

^{13}C-NMR (101 MHz, $CDCl_3$): δ = 165.0 (C_q), 151.7 (C_q), 142.2 (C_q), 137.4 (C_q), 131.8 (C_q), 129.5 (CH), 128.5 (CH), 128.3 (CH), 128.0 (C_q), 127.3 (CH), 127.0 (CH), 125.8 (C_q), 120.1 (C_q), 76.3 (C_q), 67.3 (CH_2), 55.3 (CH_2), 40.4 (CH_2), 39.5 (CH_2), 37.6 (CH_2), 37.6 (CH_2), 37.4 (CH_2), 32.9 (CH), 32.8 (CH), 31.4 (CH_2), 28.1 (CH), 24.9 (CH_2), 24.6 (CH_2), 24.4 (CH_3), 22.9 (CH_3), 22.8 (CH_3), 22.6 (CH_2), 21.1 (CH_2), 20.0 (CH_3), 19.9 (CH_3), 19.8 (CH_3), 16.3 (CH_3). One CH_2 resonance is missing due to overlap.

IR (ATR): $\tilde{\nu}$ = 2951, 2925, 2896, 2869, 1665, 1460, 1235, 1202, 1043, 940 cm^{-1}.
HR-MS (ESI): *m*/*z* calcd for $C_{37}H_{56}NO_2^+$ $[M+H]^+$ 546.4306, found 546.4298.

N-(4-(*N*-(cyclohexylcarbamoyl)sulfamoyl)phenethyl)-2'-(4,5-dihydrooxazol-2-yl)-4-methoxy-3'-methyl-[1,1'-biphenyl]-3-carboxamide (33)

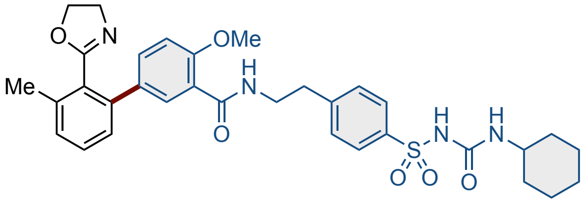

General procedure **A** was followed using 2-(*o*-tolyl)-4,5-dihydrooxazole (80.6 mg, 0.50 mmol) and 5-chloro-*N*-(4-(*N*-(cyclohexylcarbamoyl)sulfamoyl)phenethyl)-2-methoxybenzamide (370.5 mg, 0.75 mmol) at 50 °C. Purification by column chromatography on silica gel (DCM/MeOH = 40:1→20:1) yielded product **33** (124.2 mg, 40%) as a colorless oil.

^{1}H-NMR (400 MHz, $CDCl_3$): δ = 8.23 (d, *J* = 2.5 Hz, 1H), 7.93 (t, *J* = 5.8 Hz, 1H), 7.88 – 7.81 (m, 2H), 7.50 (dd, *J* = 8.6, 2.5 Hz, 1H), 7.41 – 7.30 (m, 3H), 7.24 – 7.17 (m, 2H), 6.94 (d, *J* = 8.7 Hz, 1H), 6.22 (d, *J* = 8.0 Hz, 1H), 4.27 (t, *J* = 9.6 Hz, 2H), 3.86 (t, *J* = 9.5 Hz, 2H), 3.82 – 3.72 (m, 5H), 3.56 – 3.43 (m, 1H), 3.00 (t, *J* = 6.9 Hz, 2H), 2.39 (s, 3H), 1.78 – 1.69 (m, 2H), 1.62 – 1.46 (m, 3H), 1.30 – 1.16 (m, 2H), 1.10 – 0.93 (m, 3H).

^{13}C-NMR (101 MHz, $CDCl_3$): δ = 165.6 (C_q), 165.0 (C_q), 156.9 (C_q), 151.0 (C_q), 145.6 (C_q), 140.5 (C_q), 138.3 (C_q), 137.8 (C_q), 134.1 (C_q), 132.9 (CH), 132.1 (CH), 129.8 (CH), 129.7 (CH), 129.2 (CH), 128.1 (C_q), 127.6 (CH), 127.2 (CH), 120.9 (C_q), 111.5 (CH), 67.5 (CH_2), 56.1 (CH_3), 55.1 (CH_2), 49.1 (CH), 40.6 (CH_2), 35.7 (CH_2), 33.0 (CH_2), 25.4 (CH_2), 24.7 (CH_2), 19.9 (CH_3).

IR (ATR): $\tilde{\nu}$ = 1655, 1629, 1536, 1526, 1498, 1462, 1252, 1237, 1161, 750 cm^{-1}.
HR-MS (ESI): *m*/*z* calcd for $C_{33}H_{38}N_4O_6SNa^+$ $[M+Na]^+$ 641.2404, found 641.2429.

methyl ((5*S*,10*S*,11*S*,14*S*)-11-benzyl-5-(*tert*-butyl)-10-hydroxy-15,15-dimethyl-3,6,13-trioxo-8-((3,3'',5,5''-tetramethyl-2'-(pyridin-2-yl)-[1,1':3',1''-terphenyl]-5'-yl)methyl)-2-oxa-4,7,8,12-tetraazahexadecan-14-yl)carbamate (34)

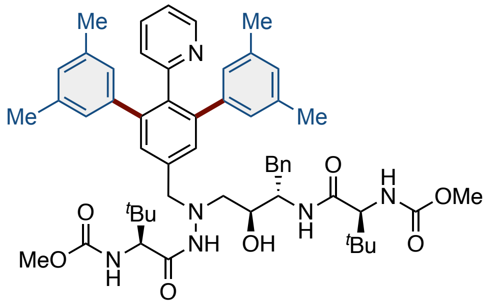

General procedure **A** was followed using methyl ((5*S*,10*S*,11*S*,14*S*)-11-benzyl-5-(*tert*-butyl)-10-hydroxy-15,15-dimethyl-3,6,13-trioxo-8-(4-(pyridin-2-yl)benzyl)-2-oxa-4,7,8,12-tetraazahexadecan-14-yl)carbamate (352.4 mg, 0.50 mmol), 1-bromo-3,5-dimethylbenzene (277.6 mg, 1.5 mmol), $RuCl_3$•nH_2O (25.2 mg, 20 mol%), NaOAc (24.6 mg, 0.30 mmol), and K_2CO_3 (207.3 mg, 1.5 mmol) at 70 °C. Purification by column chromatography on silica gel (*n*-hexane/DCM/EtOAc = 1:1:2→1:1:3) yielded product **34** (166.0 mg, 36%) as an off-white solid.

^{1}H-NMR (400 MHz, $CDCl_3$): δ = 8.33 (ddd, *J* = 4.9, 1.9, 1.0 Hz, 1H), 7.39 (s, 2H), 7.31 (td, *J* = 7.7, 1.9 Hz, 1H), 7.24 – 7.17 (m, 4H), 7.16 – 7.10 (m, 1H), 6.91 (ddd, *J* = 7.6, 5.0, 1.0 Hz, 1H), 6.83 – 6.72 (m, 4H), 6.66 (s, 4H), 6.48 (d, *J* = 9.5 Hz, 1H), 5.40 (d, *J* = 9.3 Hz, 1H), 5.23 (d, *J* = 8.4 Hz, 1H), 4.91 (s, 1H), 4.15 (d, *J* = 13.8 Hz, 1H), 4.07 (q, *J* = 8.1 Hz, 1H), 3.95 (d, *J* = 13.8 Hz, 1H), 3.79 (d, *J* = 8.5 Hz, 1H), 3.70 – 3.57 (m, 8H), 3.02 – 2.86 (m, 3H), 2.61 (dd, *J* = 12.3, 2.8 Hz, 1H), 2.13 (s, 12H), 0.86 (s, 9H), 0.80 (s, 9H).

^{13}C-NMR (101 MHz, $CDCl_3$): δ = 171.0 (C_q), 170.8 (C_q), 159.2 (C_q), 157.1 (C_q), 157.0 (C_q), 148.4 (CH), 142.4 (C_q), 141.1 (C_q), 138.3 (C_q), 138.2 (C_q), 137.0 (C_q), 135.7 (C_q), 134.9 (CH), 129.7 (CH), 129.5 (CH), 128.4 (CH), 128.1 (CH), 127.6 (CH), 126.7 (CH), 126.4 (CH), 120.8 (CH), 67.4 (CH), 63.8 (CH), 62.7 (CH_2), 61.6 (CH_2), 61.6 (CH), 52.6 (CH_3), 52.4 (CH_3), 52.2 (CH), 38.8 (CH_2), 34.5 (C_q), 34.0 (C_q), 26.7 (CH_3), 26.3 (CH_3), 21.3 (CH_3).

IR (ATR): $\tilde{\nu}$ = 2958, 1713, 1650, 1508, 1251, 1215, 1070, 850, 745, 706 cm^{-1}.
m.p.: 145–146 °C.
HR-MS (ESI): *m*/*z* calcd for $C_{54}H_{69}N_6O_7^+$ [M+H]$^+$ 913.5222, found 913.5231.
The characterization data are consistent with those reported in the literature.[14]

2-(3-(*tert*-butyl)phenyl)pyridine (36)

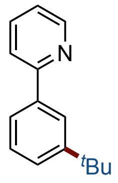

General procedure **C** was followed using 2-phenylpyridine (77.6 mg, 0.50 mmol) and 2-bromo-2-methylpropane (205.5 mg, 1.5 mmol). Purification by column chromatography on silica gel (*n*-hexane/EtOAc = 20:1) yielded product **36** (76.2 mg, 72%) as a colorless oil.

^{1}H-NMR (400 MHz, $CDCl_3$): δ = 8.71 (dt, *J* = 4.8, 1.4 Hz, 1H), 8.04 (t, *J* = 1.9 Hz, 1H), 7.79 – 7.71 (m, 3H), 7.49 – 7.44 (m, 1H), 7.41 (t, *J* = 7.6 Hz, 1H), 7.22 (ddd, *J* = 6.7, 4.8, 2.0 Hz, 1H), 1.40 (s, 9H).

^{13}C-NMR (101 MHz, $CDCl_3$): δ = 158.2 (C_q), 151.8 (C_q), 149.8 (CH), 139.4 (C_q), 136.8 (CH), 128.6 (CH), 126.2 (CH), 124.3 (CH), 124.1 (CH), 122.1 (CH), 120.9 (CH), 35.0 (C_q), 31.6 (CH_3).

IR (ATR): $\tilde{\nu}$ = 2964, 2951, 1584, 1565, 1461, 1431, 1252, 772, 742, 700 cm^{-1}.
HR-MS (ESI): *m*/*z* calcd for $C_{15}H_{18}N^+$ [M+H]$^+$ 212.1434, found 212.1440.
The characterization data are consistent with those reported in the literature.[15]

1-(3-(*tert*-butyl)phenyl)-1*H*-pyrazole (37)

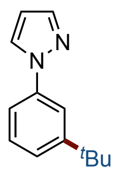

General procedure **C** was followed using 1-phenyl-1*H*-pyrazole (72.1 mg, 0.50 mmol) and 2-bromo-2-methylpropane (205.5 mg, 1.5 mmol). Purification by column chromatography on silica gel (*n*-hexane/EtOAc = 20:1) yielded product **37** (45.3 mg, 45%) as a colorless oil.

^{1}H-NMR (400 MHz, $CDCl_3$): δ = 7.92 (d, *J* = 2.4 Hz, 1H), 7.76 (t, *J* = 2.0 Hz, 1H), 7.73 (d, *J* = 1.8 Hz, 1H), 7.45 (ddd, *J* = 7.7, 2.0, 1.2 Hz, 1H), 7.38 (t, *J* = 7.8 Hz, 1H), 7.33 (ddd, *J* = 7.8, 1.9, 1.3 Hz, 1H), 6.46 (t, *J* = 2.1 Hz, 1H), 1.37 (s, 9H).

^{13}C-NMR (101 MHz, $CDCl_3$): δ = 153.1 (C_q), 141.1 (CH), 140.2 (C_q), 129.1 (CH), 127.1 (CH), 123.8 (CH), 117.0 (CH), 116.6 (CH), 107.5 (CH), 35.1 (C_q), 31.4 (CH_3).

IR (ATR): $\tilde{v}$ = 1608, 1589, 1518, 1391, 1043, 947, 787, 757, 743, 699 cm^{-1}.
HR-MS (ESI): *m/z* calcd for $C_{13}H_{17}N_2^+$ $[M+H]^+$ 201.1386, found 201.1391.
The characterization data are consistent with those reported in the literature.[16]

dimethyl 2-(3-(pyridin-2-yl)phenyl)succinate (38)

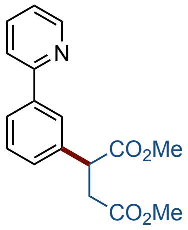

General procedure **C** was followed using 2-phenylpyridine (77.6 mg, 0.50 mmol) and 1-(1,4-dimethoxy-1,4-dioxobutan-2-yl)-2,4,6-triphenylpyridin-1-ium tetrafluoroborate (539.3 mg, 1.0 mmol). Purification by column chromatography on silica gel (*n*-hexane/EtOAc = 3:1) yielded product **38** (75.6 mg, 51%) as a yellow oil.

^{1}H-NMR (400 MHz, $CDCl_3$): δ = 8.69 (ddd, *J* = 4.8, 1.8, 1.0 Hz, 1H), 7.95 – 7.86 (m, 2H), 7.78 – 7.68 (m, 2H), 7.43 (t, *J* = 7.7 Hz, 1H), 7.33 (dt, *J* = 7.6, 1.5 Hz, 1H), 7.23 (ddd, *J* = 7.1, 4.8, 1.5 Hz, 1H), 4.19 (dd, *J* = 10.2, 5.1 Hz, 1H), 3.68 (s, 3H), 3.68 (s, 3H), 3.28 (dd, *J* = 17.0, 10.2 Hz, 1H), 2.73 (dd, *J* = 17.0, 5.1 Hz, 1H).

^{13}C-NMR (101 MHz, $CDCl_3$): δ = 173.5 (C_q), 172.1 (C_q), 157.0 (C_q), 149.8 (CH), 140.2 (C_q), 138.3 (C_q), 136.9 (CH), 129.4 (CH), 128.3 (CH), 126.6 (CH), 126.4 (CH), 122.4 (CH), 120.8 (CH), 52.5 (CH_3), 52.0 (CH_3), 47.3 (CH), 37.8 (CH_2).

IR (ATR): $\tilde{v}$ = 1729, 1584, 1462, 1435, 1225, 1196, 1160, 1005, 773, 745 cm^{-1}.
HR-MS (EI): *m/z* calcd for $C_{17}H_{17}NO_4^+$ $[M]^+$ 299.1152, found 299.1155.
The characterization data are consistent with those reported in the literature.[17]

[Ru(^{t}BuCN)$_5$(H_2O)](BF_4)$_2$ (Ru2)

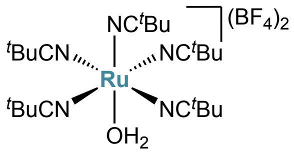

The previously reported procedure[4] yielded product **Ru2** (616.3 mg, 44%) as a pale-yellow powder.

^{1}**H-NMR** (400 MHz, CD_2Cl_2): δ = 1.51 (s, 36H), 1.46 (s, 9H).

^{13}**C-NMR** (101 MHz, CD_2Cl_2): δ = 136.2 (C_q), 134.2 (C_q), 31.2 (C_q), 30.8 (C_q), 28.7 (CH_3), 28.3 (CH_3).

^{19}**F-NMR** (377 MHz, CD_2Cl_2): δ = −150.5, −150.6.

IR (ATR): $\tilde{v}$ = 3389, 2977, 2939, 2270, 1481, 1462, 1372, 1243, 1063, 993 cm^{-1}.
m.p.: 182–183 °C (decomp.).
HR-MS (ESI): *m*/*z* calcd for $C_{25}H_{47}N_5ORuBF_4^+$ $[M-BF_4]^+$ 622.2850, found 622.2850.
The characterization data are consistent with those reported in the literature.[4]

5. Unsuccessful Examples

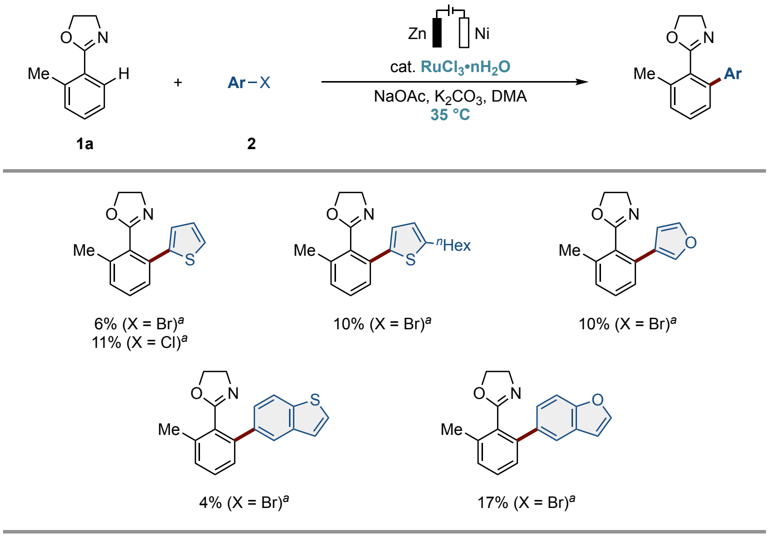

[a] Yields were determined by ^{1}H-NMR analysis using dibromomethane as the internal standard.

Figure S2. Unsuccessful examples

6. Mechanistic Studies

6-1. Control experiment: electrolysis for 40 min

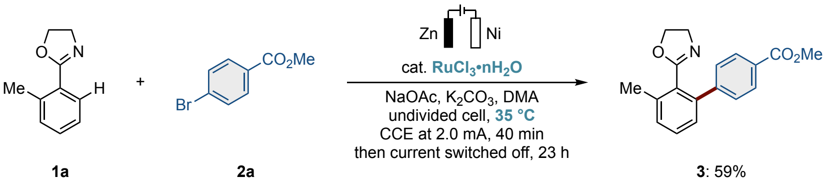

The reaction was carried out in an undivided cell with a zinc anode (25 mm × 10 mm × 0.50 mm) and a nickel foam cathode (25 mm × 10 mm × 1.4 mm). Under N_2, 2-aryloxazoline **1a** (81 mg, 0.50 mmol), aryl bromide **2a** (161 mg, 0.750 mmol, 1.50 equiv), $RuCl_3$•nH_2O (13 mg, 50 μmol, 10 mol%), NaOAc (12 mg, 0.15 mmol, 0.30 equiv), K_2CO_3 (138 mg, 1.00 mmol, 2.00 equiv), and DMA (3.0 mL) were charged in the cell. The electrolysis was performed in a constant current mode at 2.0 mA at 35 °C. After 40 min, the current was switched off, and the reaction was continued for 23 h. Next, the reaction mixture was transferred into a separatory funnel, and the electrodes were rinsed with EtOAc (5 mL). Saturated NH_4Cl aqueous solution (10 mL) was added, and the aqueous layer was extracted with EtOAc (3 × 20 mL). The combined organic layers were washed with water (2 × 20 mL) and brine (20 mL). The organic phase was dried over Na_2SO_4, filtered, and concentrated. The crude reaction mixture was analyzed by ^{1}H-NMR using dibromomethane as the internal standard to determine the yield.

6-2. Cyclic voltammetry

A glassy carbon disk electrode (diameter: 3 mm) was used as the working electrode, a platinum wire as the counter electrode, and a silver wire as the pseudo reference electrode. The measurements were carried out at a scan rate of 50 or 100 mV/s at 35 °C under N_2. Potentials were calibrated using ferrocene as the internal standard.

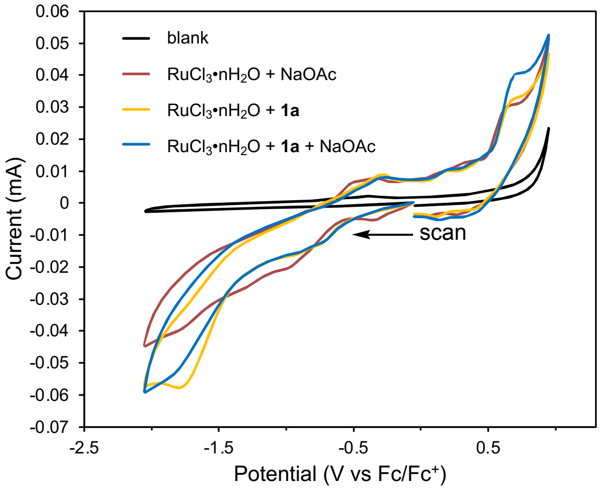

Figure S3. Cyclic voltammograms at 50 mV/s using ^{n}Bu$_4$NPF$_6$ as the electrolyte (0.1 M in DMA). Analyte concentrations: 5 mM. Red: $RuCl_3$•nH_2O and NaOAc. Yellow: $RuCl_3$•nH_2O and **1a**. Blue: $RuCl_3$•nH_2O, **1a**, and NaOAc.

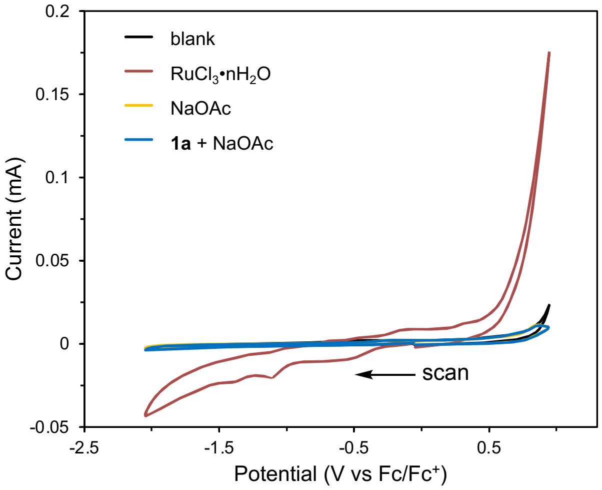

Figure S4. Cyclic voltammograms at 50 mV/s using ^{n}Bu$_4$NPF$_6$ as the electrolyte (0.1 M in DMA). Analyte concentrations: 5 mM. Red: $RuCl_3$•nH_2O. Yellow: NaOAc. Blue: **1a** and NaOAc.

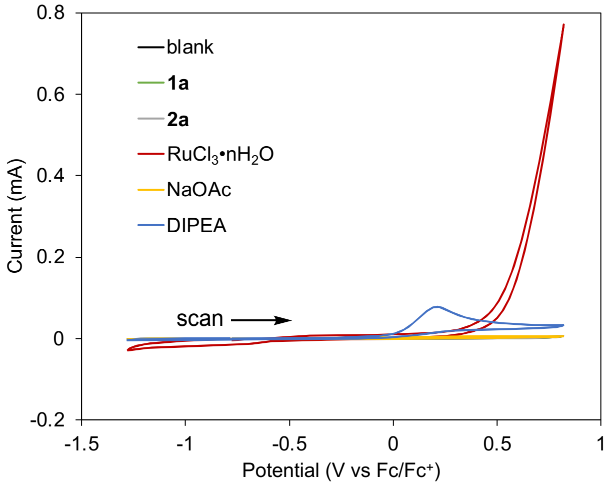

Figure S5. Cyclic voltammograms at 100 mV/s using ^{n}Bu$_4$NPF$_6$ as the electrolyte (0.1 M in DMA). Analyte concentrations: 5 mM. Green: **1a**. Gray: **2a**. Red: $RuCl_3$•nH_2O. Yellow: NaOAc. Blue: DIPEA. Cyclic voltammograms of compounds **1a** and **2a** are not visible due to overlap with the one of NaOAc.

6-3. Spectroelectrochemistry (SEC)

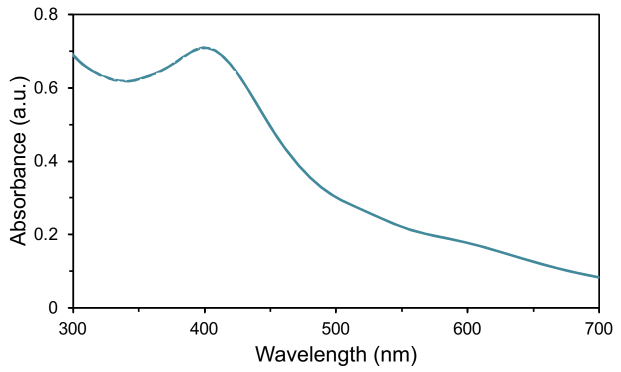

Figure S6. UV/Vis absorption spectrum of the DMA solution containing **1a** (0.13 mM), $RuCl_3$•nH_2O (0.13 mM), and NaOPiv (0.13 mM).

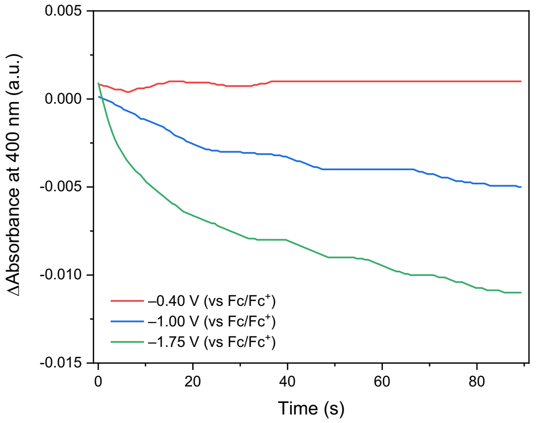

Figure S7. Spectroelectrochemical analysis of the DMA solution containing **1a** (0.2 mM), $RuCl_3$•nH_2O (0.2 mM), NaOPiv (0.2 mM), and $^{n}Bu_4NPF_6$ (0.1 M) at 35 °C under N_2. Measurements were performed at −0.40, −1.00, or −1.75 V (vs Fc/Fc^+).

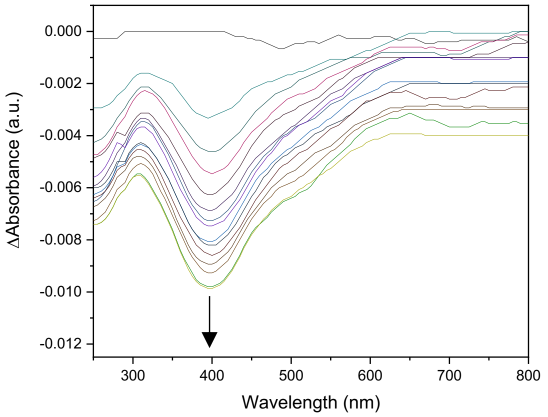

Figure S8. Spectroelectrochemical analysis of the DMA solution containing **1a** (0.2 mM), $RuCl_3$•nH_2O (0.2 mM), NaOPiv (0.2 mM), and $^{n}Bu_4NPF_6$ (0.1 M) at 35 °C under N_2. A potential of −1.75 V (vs Fc/Fc^+) was applied over 85 s.

6-4. Monocyclometalated complex as the catalyst

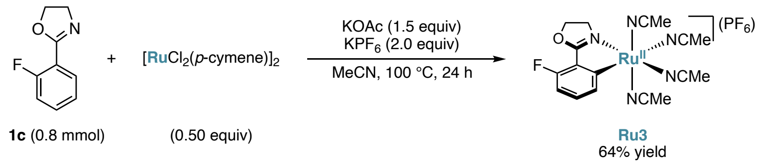

The previously reported procedure[5,6] yielded monocyclometalated complex **Ru3** (294.3 mg, 64%) as a yellow solid.

^{1}H-NMR (400 MHz, MeCN-d_3): δ = 7.69 (dt, J = 7.4, 1.0 Hz, 1H), 7.13 (ddd, J = 8.1, 7.4, 5.6 Hz, 1H), 6.56 (ddd, J = 11.1, 8.1, 0.9 Hz, 1H), 4.79 (t, J = 9.5 Hz, 2H), 3.79 (t, J = 9.5 Hz, 2H), 2.48 (s, 3H), 2.11 (s, 6H), 1.96 (s, 3H).

^{13}C-NMR (101 MHz, MeCN-d_3): δ = 192.2 (d, J_{CF} = 3.9 Hz, C$_q$), 174.1 (d, J_{CF} = 4.7 Hz, C$_q$), 160.8 (d, J_{CF} = 258.6 Hz, C$_q$), 135.0 (d, J_{CF} = 2.9 Hz, CH), 131.0 (d, J_{CF} = 7.4 Hz, CH), 123.6 (C$_q$), 122.5 (C$_q$), 107.8 (d, J_{CF} = 20.0 Hz, CH), 71.7 (CH_2), 52.1 (CH_2), 4.3 (CH_3), 3.9 (CH_3). Resonances corresponding to one C$_q$ and one of the MeCN ligands are missing due to low intensity or overlap.

^{19}F-NMR (282 MHz, MeCN-d_3): δ = −71.7, −74.2, −117.5.

IR (ATR): $\tilde{\nu}$ = 2273, 1626, 1591, 1537, 1425, 1383, 1276, 1228, 1108, 783 cm^{-1}.
HR-MS (ESI): *m*/*z* calcd for $C_{15}H_{16}FN_4ORu^+$ [M−MeCN−PF_6]$^+$ 389.0350, found 389.0340.

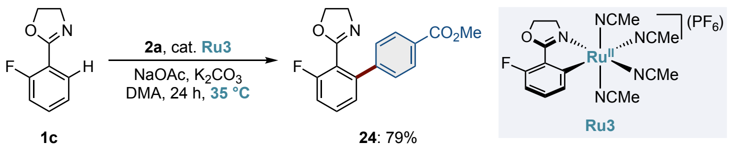

In an N_2 filled glovebox, 2-aryloxazoline **1c** (83 mg, 0.50 mmol, 1.0 equiv), aryl bromide **2a** (161 mg, 0.750 mmol, 1.50 equiv), complex **Ru3** (29 mg, 50 µmol, 10 mol%), NaOAc (12 mg, 0.15 mmol, 0.30 equiv), K_2CO_3 (138 mg, 1.00 mmol, 2.00 equiv), and DMA (3.0 mL) were charged in a 10 mL vial. The vial was closed with a screw cap, and the reaction mixture was stirred at 35 °C. After 24 h, the mixture was transferred to a separatory funnel, and saturated NH_4Cl aqueous solution (10 mL) was added. The aqueous phase was extracted with EtOAc (3 × 20 mL). The combined organic layers were washed with water (20 mL) and brine (20 mL). The organic phase was then dried over Na_2SO_4, filtered, and concentrated. The crude reaction mixture was purified by silica gel column chromatography (eluent: *n*-hexane/EtOAc = 2:1→1:1) to yield the desired product.

6-5. Stoichiometric reactions

In an N_2 filled glovebox, complex **Ru3** (57 mg, 0.10 mmol, 1.0 equiv), aryl bromide **2a** (32 mg, 0.15 mmol, 1.5 equiv), 2-aryloxazoline **1c** (17 mg, 0.10 mmol, 1.0 equiv, for entries 2 and 3), NaOAc (12 mg, 0.30 mmol, 3.0 equiv, for entries 3 and 4), pyridine (8.1 µL, 0.10 mmol, 1.0 equiv, for entry 4), and DMA (0.60 mL) were charged in a 10 mL vial. The vial was closed with a screw cap, and the reaction mixture was stirred at 35 °C. After 24 h, the mixture was transferred to a separatory funnel, and saturated NH_4Cl aqueous solution (10 mL) was added. The aqueous phase was extracted with Et_2O (3 × 20 mL). The combined organic layers were washed with water (20 mL) and brine (20 mL). The organic phase was then dried over Na_2SO_4, filtered, and concentrated. The crude reaction mixture was analyzed by ^{1}H-NMR using dibromomethane as the internal standard to determine the yield.

Table S4. Stoichiometric reactions.[a]

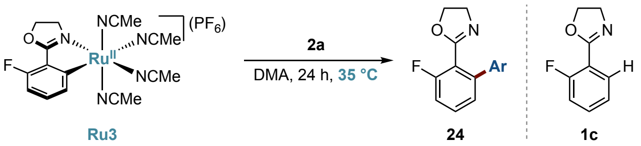

Entry	Deviations	Yield of **24** (%)[b]
1	None	N.D.
2	**1c** (1.0 equiv)	N.D.
3	**1c** (1.0 equiv), NaOAc (3.0 equiv)	35
4	Pyridine (1.0 equiv), NaOAc (3.0 equiv)	N.D.

[a] Reaction conditions: **Ru3** (0.10 mmol), **2a** (1.5 equiv), DMA (0.60 mL), 24 h, 35 °C. [b] Determined by ^{1}H-NMR analysis using dibromomethane as the internal standard.

6-6. Determination of electron transfer number

The number of electrons transferred during the electroreduction of ruthenium(III) species was determined by using the procedure described by Amatore.[18] Two solutions were prepared as follows:

Solution A: substrate **1a** (5.0 mM), $RuCl_3$•nH_2O (5.0 mM), NaOPiv (5.0 mM), and $^n Bu_4NPF_6$ (0.10 M) in DMF.

Solution B: Ferrocene (5.0 mM) and $^n Bu_4NPF_6$ (0.10 M) in DMF.

Solution A was stirred overnight to dissolve the analytes. Distilled and degassed DMF was used for measurements to observe clear redox events of ferrocene. A glassy carbon disc electrode (*d* = 4 mm), a coiled platinum wire (*d* = 1 mm), and a saturated calomel electrode (SCE) were used as the working electrode, the counter electrode, and the reference electrode, respectively. The operation temperature was 25 °C. The solution was degassed before the measurements, and an overpressure of protective gas was maintained throughout the measurements. For the calculation of the number of electrons, the combination of chronoamperometry and linear sweep voltammetry with a rotating disc electrode (RDE) was employed. Chronoamperometric measurements of solutions A and B were performed for 10 sec at −2.1 V and 0.85 V, respectively. Linear sweep voltammetric measurements of solutions A and B were conducted at the rotation speed of 2600 rpm and the scan rate of 50 mV/s.

From the measurements, electrochemical parameters a_{trans} (from chronoamperometry) and a_{RDE} (from linear sweep voltammetry) were calculated as follows:

$$a_{trans} = \left[n \times \left(\frac{D}{D_{Fc}} \right)^{\frac{1}{2}} \right]_{trans} = \left| \frac{i}{i_{Fc}} \times \frac{c^0_{Fc}}{c^0} \right|$$

in which n is the electron transfer number, D is the diffusion coefficient of the ruthenium species, D_{Fc} is the diffusion coefficient of ferrocene, i and i_{Fc} are slopes derived from Cottrell plots, and c^0 and c^0_{Fc} are concentrations of analytes (5.0 mM).

$$a_{RDE} = \left[n \times \left(\frac{D}{D_{Fc}} \right)^{\frac{2}{3}} \right]_{RDE} = \left| \frac{i^{lim}}{i^{lim}_{Fc}} \times \frac{c^0_{Fc}}{c^0} \right|$$

in which n is the electron transfer number, D is the diffusion coefficient of the ruthenium species, D_{Fc} is the diffusion coefficient of ferrocene, i^{lim} and i^{lim}_{Fc} are limiting current values, and c^0 and c^0_{Fc} are concentrations of analytes (5.0 mM).

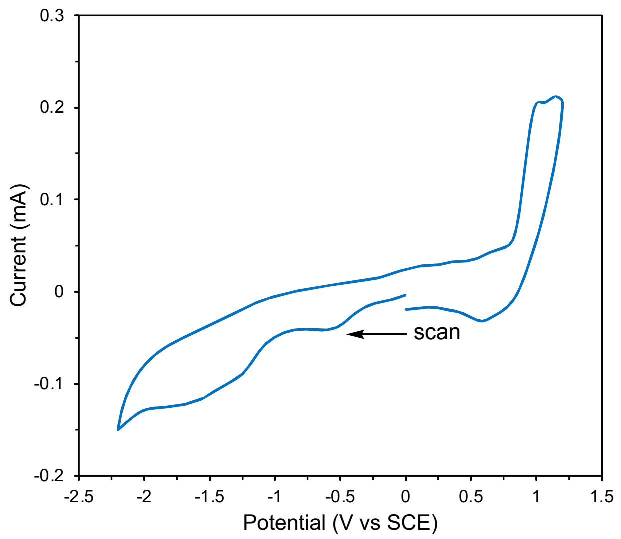

Figure S9. Cyclic voltammogram of solution A (100 mV/s).

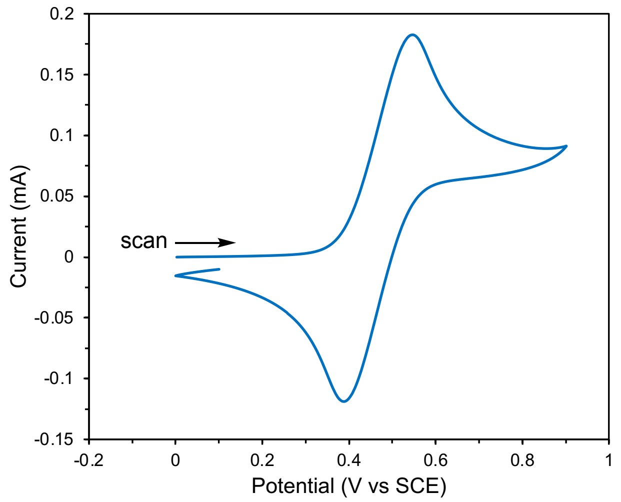

Figure S10. Cyclic voltammogram of solution B (100 mV/s).

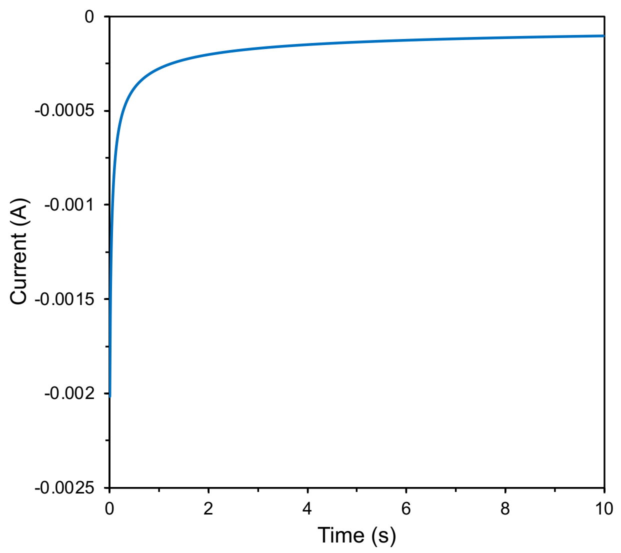

Figure S11. One of the chronoamperograms of solution A (−2.1 V).

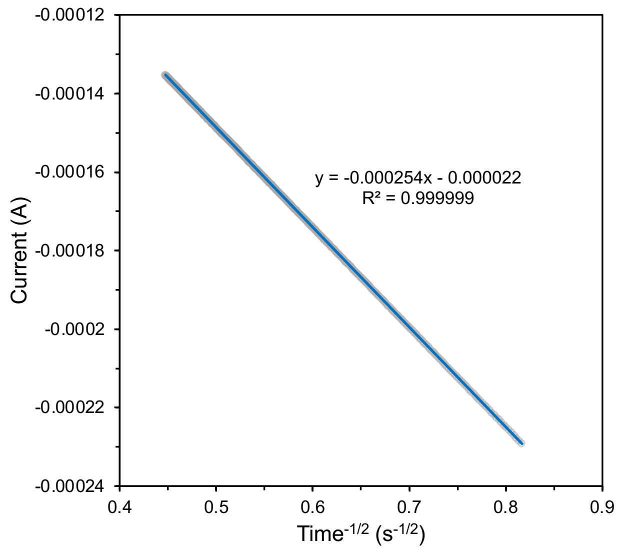

Figure S12. Cottrell plot for the chronoamperogram shown in **Figure S11** (1.5–5.0 s).

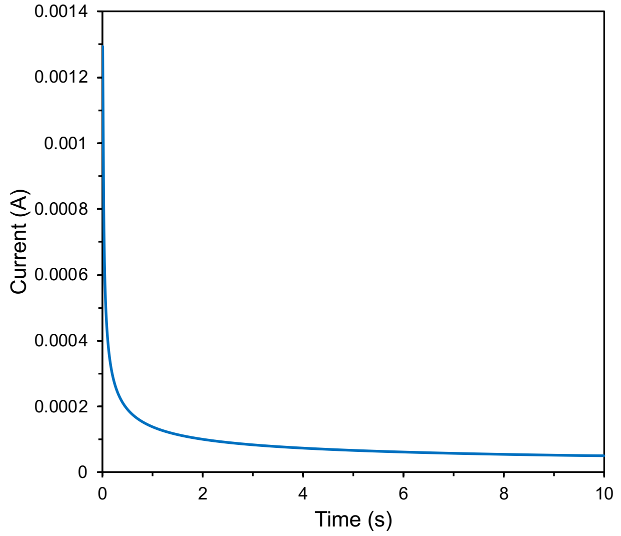

Figure S13. One of the chronoamperograms of solution B (0.85 V).

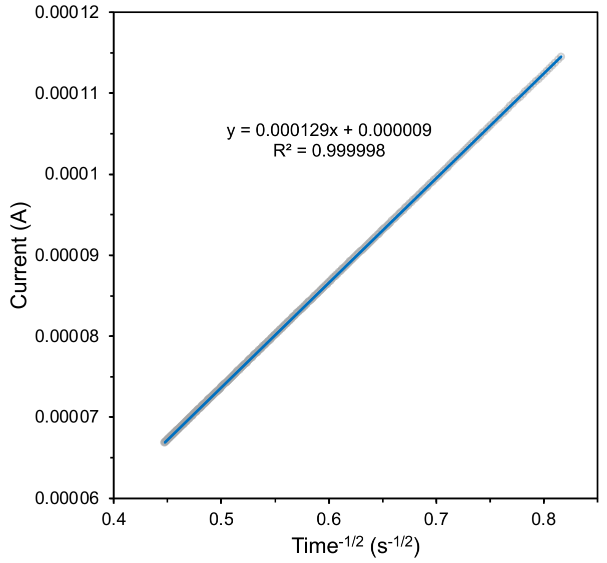

Figure S14. Cottrell plot for the chronoamperogram shown in **Figure S13** (1.5–5.0 s).

Table S5. Calculation of a_{trans}.

Measurement	i (A/s$^{-1/2}$)	i_{Fc} (A/s$^{-1/2}$)	a_{trans}
1	−2.54 × 10^{-4}	1.29 × 10^{-4}	1.97
2	−2.55 × 10^{-4}	1.29 × 10^{-4}	1.98
3	−2.60 × 10^{-4}	1.28 × 10^{-4}	2.03
4	−2.59 × 10^{-4}	1.26 × 10^{-4}	2.05
5	−2.58 × 10^{-4}	1.31 × 10^{-4}	1.97

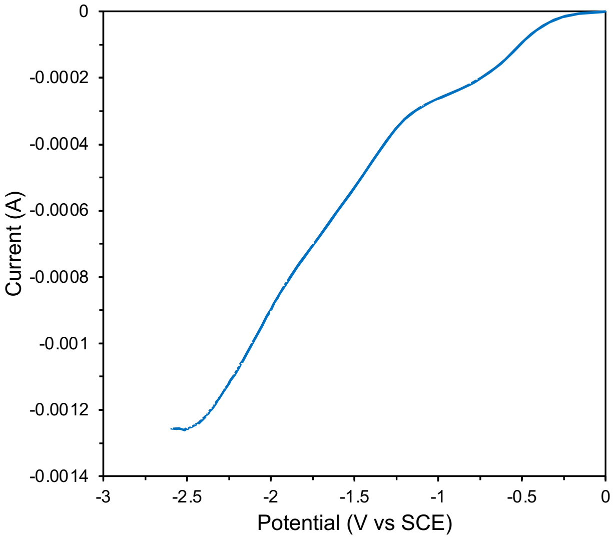

Figure S15. One of the linear sweep voltammograms of solution A (2600 rpm, 50 mV/s, −2.6–0 V).

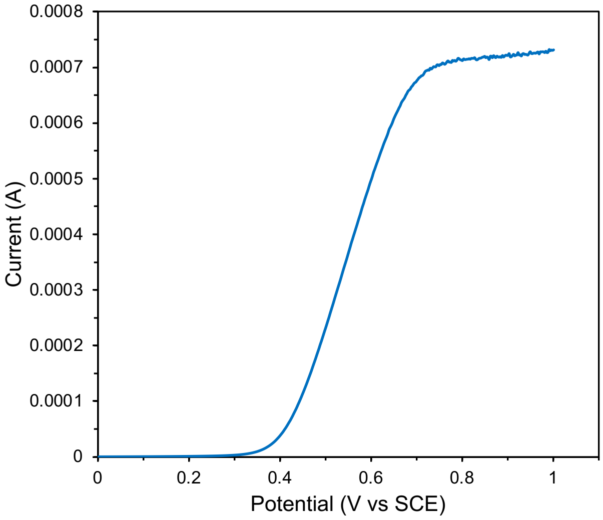

Figure S16. One of the linear sweep voltammograms of solution B (2600 rpm, 50 mV/s, 0–1.0 V).

Table S6. Calculation of a_{RDE}.

Measurement	i^{lim} at −2.45 V (A)	i^{lim}_{Fc} at 0.95 V (A)	a_{RDE}
1	-1.24×10^{-3}	7.25×10^{-4}	1.71
2	-1.25×10^{-3}	7.23×10^{-4}	1.73
3	-1.25×10^{-3}	7.18×10^{-4}	1.75
4	-1.26×10^{-3}	7.20×10^{-4}	1.75
5	-1.27×10^{-3}	7.18×10^{-4}	1.76

Calculation of averages and errors (95% confidence interval) gave parameters a_{trans} and a_{RDE} as follows:

$$a_{trans} = 2.00 \pm 0.05$$

$$a_{RDE} = 1.74 \pm 0.02$$

The electron transfer number and its error were calculated as follows:

$$n = \frac{{a_{trans}}^4}{{a_{RDE}}^3}$$

$$\frac{\Delta n}{n} = \frac{\frac{2}{3} \times \frac{\Delta a_{trans}}{a_{trans}} + \frac{1}{2} \times \frac{\Delta a_{RDE}}{a_{RDE}}}{\left|\frac{1}{2} - \frac{2}{3}\right|}$$

$$n = 3.04 \pm 0.41$$

7. Computational Studies

7-1. Computation methods

All DFT calculations were performed with Gaussian 16, Revision A.03 package.[19] The geometry optimizations were conducted at the TPSS[20] level of theory in combination with Grimme's D3 dispersion corrections with a Becke-Johnson damping scheme (D3BJ)[21,22] in the gas phase. All atoms were described with a def2-SVP basis set,[23-26] while ruthenium and bromine were also described with a SDD pseudopotential.[27,28] To confirm whether each optimized stationary point is an energy minimum or a transition state, the vibrational frequencies were computed at the same level of theory as for the geometry optimizations. The single-point energies were further evaluated with PBE0[29,30] functional in combination with a standalone version of Grimme's D4 dispersion corrections,[31,32] with a def2-TZVPP basis set in combination with a SDD pseudopotential for ruthenium.[27,28] Solvent effects were taken into account using the implicit solvation model SMD[33] with a dielectric constant of ε = 37.781, which corresponds to *N*,*N*-dimethylacetamide. All reported energies are based on gas-phase Gibbs free energies with def2-SVP basis set for which the electronic energies were corrected by PBE0-D4 with a def2-TZVPP basis set and solvent effects.

Table S7. Calculated electronic energies at the PBE0-D4/def2-TZVPP-SMD (DMA) level of theory and Gibbs free energies for all the structures (all in Hartree).

Structure	Electronic Energy	Total Gibbs Free Energy
**Ru-I**	-1507.696294	-1507.336200
**Ru-II**	-4082.837138	-4082.469011
**TS(II-III)**	-4082.819046	-4082.450891
**Ru-III**	-4082.873531	-4082.504101
**Ru-IV**	-1507.694058	-1507.332303
**Ru-V**	-4311.807154	-4311.383009
**TS(V-VI)**	-4311.774129	-4311.349763
**Ru-VI**	-4311.830814	-4311.405355
**AcOH**	-228.946455	-228.911188
**Methyl 4-bromobenzoate**	-3033.062766	-3032.969322

7-2. Cartesian coordinates of the optimized structure

Ru-I
Lowest frequency = 26.6144 cm^{-1}
Charge = 0, Multiplicity = 1

55

C	-2.220197000	-1.403295000	0.221670000
C	-2.993156000	-2.510212000	0.617267000
C	-0.983992000	-1.026820000	0.865154000
C	-2.552697000	-3.304586000	1.680388000
C	-0.574577000	-1.881384000	1.922332000
C	-1.339732000	-2.986940000	2.321946000
H	-3.138734000	-4.170840000	2.004426000
H	0.367751000	-1.676109000	2.444727000
H	-0.985491000	-3.618400000	3.145802000
C	-3.514196000	0.451125000	-2.653872000
N	-1.662969000	0.407613000	-1.192000000
C	3.514074000	0.450001000	2.654237000
N	1.662904000	0.407169000	1.192282000
C	2.220378000	-1.402870000	-0.222429000
C	0.984147000	-1.026191000	-0.865745000

C	2.993522000	-2.509436000	-0.618651000
C	0.574889000	-1.880208000	-1.923428000
C	2.553223000	-3.303247000	-1.682257000
H	3.921802000	-2.743248000	-0.085789000
C	1.340227000	-2.985410000	-2.323663000
H	-0.367457000	-1.674772000	-2.445729000
H	3.139404000	-4.169216000	-2.006797000
H	0.986106000	-3.616443000	-3.147899000
O	0.843779000	2.263232000	-1.061167000
C	1.433519000	2.190732000	-2.150859000
O	1.651972000	1.042522000	-2.770653000
C	1.947300000	3.405179000	-2.876342000
H	3.041562000	3.328698000	-2.999194000
H	1.508477000	3.449805000	-3.888163000
H	1.693202000	4.312652000	-2.312121000
O	3.590946000	-0.663254000	1.716269000
O	-3.590936000	-0.662676000	-1.716536000
C	-2.114788000	1.074893000	-2.417167000
C	-2.516780000	-0.543876000	-0.896993000
C	2.516816000	-0.544063000	0.896741000
C	2.114590000	1.073730000	2.417888000
H	1.403277000	0.860089000	3.238478000
H	2.150470000	2.166995000	2.276097000
H	3.661111000	0.047173000	3.668407000
H	4.336983000	1.146824000	2.416744000
H	-3.921422000	-2.743857000	0.084306000
H	-2.150805000	2.168062000	-2.274675000
H	-1.403478000	0.861868000	-3.237919000
H	-4.337198000	1.147704000	-2.415989000
H	-3.661174000	0.048853000	-3.668271000
H	1.317326000	0.313530000	-2.143173000
Ru	-0.000033000	0.536740000	0.000153000
O	-0.844007000	2.262490000	1.062381000
C	-1.433819000	2.189328000	2.151988000
O	-1.652148000	1.040764000	2.771183000
C	-1.947813000	3.403325000	2.878076000
H	-1.317375000	0.312109000	2.143394000
H	-1.693920000	4.311122000	2.314284000
H	-3.042052000	3.326565000	3.000950000
H	-1.508944000	3.447552000	3.889896000

Ru-II

Lowest frequency = 14.5303 cm^{-1}

Charge = 0, Multiplicity = 1

5

Ru	-1.445436000	-0.121706000	-0.671081000
C	0.862587000	1.113955000	0.631330000
C	1.842468000	1.403656000	1.597895000
C	-0.199986000	0.163676000	0.847725000
C	1.798305000	0.747901000	2.831146000
C	-0.215123000	-0.461035000	2.117716000
C	0.766447000	-0.179636000	3.080176000
H	2.557485000	0.949519000	3.593476000
H	-1.007560000	-1.178092000	2.357879000
H	0.726021000	-0.689394000	4.050763000
C	1.285761000	2.790443000	-2.585559000
N	-0.133931000	1.245853000	-1.502039000

C	-4.374275000	-2.766130000	1.354138000
N	-2.836458000	-1.300429000	0.319916000
C	-3.858208000	0.800594000	0.723199000
C	-4.856768000	1.643510000	1.245176000
C	-2.688851000	1.289776000	0.037634000
C	-4.711681000	3.028267000	1.118319000
C	-2.576233000	2.698664000	-0.039790000
C	-3.566052000	3.545403000	0.482072000
H	-5.474897000	3.704358000	1.517361000
H	-1.695839000	3.146576000	-0.513921000
H	-3.445087000	4.632234000	0.395078000
H	-5.727698000	1.209444000	1.748672000
H	2.631117000	2.128130000	1.367340000
C	0.807715000	1.674683000	-0.693254000
C	-0.038111000	2.000882000	-2.757277000
C	-3.843157000	-0.638133000	0.845669000
C	-2.973242000	-2.713863000	0.693722000
O	-4.762417000	-1.369695000	1.514117000
O	1.640017000	2.623222000	-1.181400000
H	1.196326000	3.869366000	-2.784188000
H	-0.921066000	2.658634000	-2.866446000
H	-0.015729000	1.319233000	-3.625359000
H	2.111795000	2.376401000	-3.189890000
H	-4.384477000	-3.233827000	2.350793000
H	-5.131953000	-3.252801000	0.715753000
H	-2.159799000	-2.999261000	1.387625000
H	-2.897675000	-3.367258000	-0.192558000
Br	-0.073874000	-2.237059000	-1.644019000
C	1.666304000	-1.668935000	-1.052050000
C	2.085483000	-2.054019000	0.228095000
C	2.390822000	-0.768213000	-1.841658000
H	1.486321000	-2.746340000	0.822782000
C	3.559470000	-0.213395000	-1.311065000
H	2.032784000	-0.491979000	-2.836415000
C	3.987787000	-0.558375000	-0.016285000
H	4.145119000	0.517593000	-1.876123000
C	5.188902000	0.136009000	0.523087000
O	5.849566000	0.960611000	-0.086499000
O	5.467558000	-0.237109000	1.799405000
C	6.597964000	0.423388000	2.392222000
H	7.512535000	0.219898000	1.810650000
H	6.433233000	1.513444000	2.421834000
H	6.681911000	0.014025000	3.408458000
C	3.257487000	-1.494433000	0.741821000
H	3.591952000	-1.749808000	1.749304000

TS(II-III)

Lowest frequency = -120.1215 cm^{-1}

Charge = 0, Multiplicity = 1

57

Ru	1.121736000	0.028857000	0.568383000
C	-0.594646000	1.779513000	-1.037170000
C	-1.529902000	2.195750000	-2.003595000
C	-0.125279000	0.429131000	-0.951269000
C	-2.027011000	1.257653000	-2.911321000
C	-0.635649000	-0.484314000	-1.894017000
C	-1.573960000	-0.075036000	-2.853111000

H	-2.765324000	1.555301000	-3.662663000
H	-0.302916000	-1.527055000	-1.872961000
H	-1.966497000	-0.808155000	-3.567671000
C	0.440735000	4.303963000	1.398194000
N	0.746633000	2.023684000	0.886585000
C	2.585577000	-4.086792000	-0.191896000
N	1.728315000	-1.902986000	0.051028000
C	3.200637000	-0.699514000	-1.374472000
C	4.249889000	-0.599481000	-2.307417000
C	2.501573000	0.438977000	-0.856487000
C	4.622841000	0.663848000	-2.774268000
C	2.901383000	1.694012000	-1.367408000
C	3.942973000	1.802766000	-2.300855000
H	5.431935000	0.767761000	-3.504434000
H	2.391477000	2.606798000	-1.042869000
H	4.228821000	2.793506000	-2.674068000
H	4.758034000	-1.505742000	-2.653988000
H	-1.869028000	3.236717000	-2.020764000
C	-0.031361000	2.601578000	0.004386000
C	1.259805000	3.050033000	1.799909000
C	2.714715000	-1.937094000	-0.815730000
C	1.391057000	-3.297679000	0.383888000
O	3.211237000	-3.155668000	-1.123205000
O	-0.226797000	3.931396000	0.153390000
H	1.052782000	5.196108000	1.196109000
H	2.347687000	3.176424000	1.642384000
H	1.100145000	2.753413000	2.850396000
H	-0.342747000	4.557381000	2.133299000
H	2.306256000	-4.990994000	-0.752675000
H	3.335623000	-4.345444000	0.576669000
H	0.439432000	-3.576241000	-0.106501000
H	1.271730000	-3.426772000	1.471891000
Br	0.427760000	-0.680787000	2.963950000
C	-1.077332000	-0.532857000	1.534399000
C	-1.594356000	-1.732113000	1.002144000
C	-1.893713000	0.615906000	1.638110000
H	-1.011650000	-2.651758000	1.045607000
C	-3.122835000	0.627077000	0.985620000
H	-1.528967000	1.503787000	2.157580000
C	-3.583690000	-0.514023000	0.295160000
H	-3.741836000	1.529193000	0.972689000
C	-4.847089000	-0.403474000	-0.467802000
O	-5.142744000	-1.550580000	-1.145596000
O	-5.556227000	0.589965000	-0.517333000
C	-6.342734000	-1.493002000	-1.929941000
H	-7.215682000	-1.279256000	-1.290280000
H	-6.266475000	-0.701398000	-2.694620000
H	-6.438951000	-2.481063000	-2.402067000
C	-2.823552000	-1.703183000	0.345663000
H	-3.198657000	-2.600352000	-0.153817000

Ru-III

Lowest frequency = 20.2880 cm^{-1}

Charge = 0, Multiplicity = 1

57

Ru	-0.685721000	-0.024964000	-0.486575000
C	-0.606202000	2.430477000	1.222553000

C	-0.550880000	3.285143000	2.343359000
C	-0.527368000	1.011727000	1.331040000
C	-0.427582000	2.723816000	3.614157000
C	-0.435699000	0.481323000	2.630699000
C	-0.381360000	1.321592000	3.750458000
H	-0.381593000	3.364844000	4.500122000
H	-0.421774000	-0.604537000	2.775054000
H	-0.307755000	0.879612000	4.750788000
C	-1.146701000	4.088593000	-1.991931000
N	-0.950858000	1.932873000	-1.059985000
C	-0.072030000	-4.330105000	0.208141000
N	-0.466309000	-2.032299000	-0.015156000
C	-2.569741000	-1.783755000	0.987661000
C	-3.745781000	-2.202783000	1.641276000
C	-2.387200000	-0.441154000	0.554659000
C	-4.763228000	-1.273442000	1.860936000
C	-3.425094000	0.477553000	0.790518000
C	-4.601881000	0.057302000	1.425806000
H	-5.686375000	-1.576428000	2.365054000
H	-3.318039000	1.521722000	0.482993000
H	-5.406493000	0.782092000	1.593981000
H	-3.850420000	-3.247213000	1.952103000
H	-0.616318000	4.369030000	2.203132000
C	-0.826168000	2.855904000	-0.135237000
C	-1.270335000	2.583187000	-2.339844000
C	-1.460576000	-2.614621000	0.601516000
C	0.415618000	-3.077335000	-0.552219000
O	-1.386320000	-3.950648000	0.738026000
O	-0.974195000	4.127568000	-0.536352000
H	-2.042306000	4.680879000	-2.233229000
H	-2.282802000	2.286505000	-2.662659000
H	-0.568149000	2.261960000	-3.126434000
H	-0.258861000	4.566540000	-2.437878000
H	0.569073000	-4.583214000	1.070688000
H	-0.213498000	-5.219440000	-0.422893000
H	1.474449000	-2.841623000	-0.364253000
H	0.252309000	-3.140179000	-1.643882000
Br	-1.557945000	-0.811363000	-2.699506000
C	1.266262000	0.022391000	0.031515000
C	1.998529000	-0.685500000	1.004984000
C	1.969080000	0.748638000	-0.960467000
H	1.468602000	-1.228155000	1.792704000
C	3.367575000	0.703264000	-1.008143000
H	1.430971000	1.337213000	-1.711002000
C	4.094491000	-0.023321000	-0.047086000
H	3.920750000	1.246293000	-1.781050000
C	5.575831000	-0.016378000	-0.137597000
O	6.157904000	-0.757047000	0.848362000
O	6.226619000	0.573141000	-0.985848000
C	7.592773000	-0.785622000	0.814478000
H	7.949540000	-1.213130000	-0.137784000
H	8.002928000	0.233233000	0.916762000
H	7.898757000	-1.414756000	1.662305000
C	3.393920000	-0.713763000	0.965640000
H	3.957413000	-1.272025000	1.718637000

Ru-IV

Lowest frequency = 19.2521 cm^{-1}

Charge = 0, Multiplicity = 1

55

Ru	0.446358000	-0.406513000	0.245480000
C	2.932629000	0.994399000	-0.420427000
C	3.930623000	1.980137000	-0.503451000
C	1.590753000	1.243711000	0.074034000
C	3.639483000	3.290998000	-0.110769000
C	1.350372000	2.604574000	0.432993000
C	2.342717000	3.592546000	0.346742000
H	4.403558000	4.072637000	-0.168725000
H	0.349549000	2.909214000	0.763432000
H	2.099148000	4.622348000	0.635907000
C	3.930186000	-2.297643000	-1.668173000
N	2.096090000	-1.199005000	-0.673336000
C	-1.843557000	2.171110000	2.787783000
N	-1.046324000	0.542227000	1.283859000
C	-3.215305000	0.130301000	0.119278000
C	-2.985172000	-1.191426000	-0.322688000
C	-4.338963000	0.843241000	-0.355535000
C	-3.857294000	-1.773644000	-1.247562000
C	-5.199535000	0.253935000	-1.288920000
H	-4.522083000	1.857238000	0.011533000
C	-4.958868000	-1.054009000	-1.739864000
H	-3.678066000	-2.800429000	-1.583056000
H	-6.063671000	0.813882000	-1.662040000
H	-5.635667000	-1.515508000	-2.467107000
O	-0.575239000	-0.321420000	-1.543279000
C	-0.798216000	0.655759000	-2.287645000
O	-0.237124000	1.833993000	-2.096333000
C	-1.778289000	0.564570000	-3.418680000
H	-1.866693000	-0.478852000	-3.751617000
H	-2.767530000	0.890795000	-3.047093000
H	-1.481462000	1.223880000	-4.248682000
H	0.388677000	1.726345000	-1.306143000
O	1.156794000	-1.079475000	2.108874000
C	0.514605000	-2.195274000	2.062257000
O	-0.204059000	-2.452520000	1.046480000
C	0.607472000	-3.155670000	3.224847000
H	0.673713000	-4.190718000	2.852363000
H	1.473509000	-2.917078000	3.860268000
H	-0.313169000	-3.079913000	3.831348000
O	-2.920421000	1.602080000	1.992241000
O	4.237841000	-0.908492000	-1.339810000
C	2.499684000	-2.536340000	-1.113879000
C	3.108331000	-0.380508000	-0.815532000
C	-2.329120000	0.740840000	1.110889000
C	-0.693165000	1.184854000	2.580218000
H	0.298631000	1.652546000	2.520388000
H	-0.653749000	0.397144000	3.352854000
H	-1.618239000	3.177455000	2.387173000
H	-2.198818000	2.257667000	3.825060000
H	4.925422000	1.710762000	-0.875477000
H	2.479400000	-3.237085000	-0.260038000
H	1.800384000	-2.918948000	-1.877001000
H	4.700877000	-2.930658000	-1.200570000
H	3.991724000	-2.401585000	-2.764926000
H	-2.132109000	-1.749227000	0.076495000

Ru-V

Lowest frequency = 16.5514 cm^{-1}

Charge = 0, Multiplicity = 1

65

Ru	1.013362000	0.904630000	0.002965000
C	-1.684508000	1.132976000	1.104694000
C	-2.807826000	0.982075000	1.937723000
C	-0.399563000	0.536358000	1.378925000
C	-2.700069000	0.204478000	3.094695000
C	-0.357301000	-0.271532000	2.544953000
C	-1.472890000	-0.424981000	3.382581000
H	-3.562862000	0.067150000	3.753936000
H	0.561627000	-0.818176000	2.790951000
H	-1.392499000	-1.060362000	4.273657000
C	-2.370774000	2.847083000	-2.051101000
C	3.614949000	-0.078240000	3.268814000
N	2.545667000	0.239415000	1.189278000
C	3.843045000	-1.464022000	-0.079705000
C	3.665699000	-0.940217000	-1.381423000
C	4.390481000	-2.754672000	0.088767000
C	3.994753000	-1.724105000	-2.493234000
C	4.711041000	-3.529361000	-1.030970000
C	4.505782000	-3.020848000	-2.323568000
H	3.858205000	-1.314234000	-3.499327000
H	5.120826000	-4.535965000	-0.896220000
H	4.754987000	-3.631599000	-3.197930000
O	1.639449000	2.834744000	0.498800000
C	2.402655000	2.992806000	-0.529801000
O	2.486777000	2.062611000	-1.385573000
C	3.197285000	4.271223000	-0.663523000
H	3.265021000	4.561706000	-1.723948000
H	2.744706000	5.078126000	-0.067432000
H	4.225303000	4.095986000	-0.297780000
H	-3.756035000	1.456235000	1.661928000
H	3.297195000	0.084701000	-1.503562000
O	4.239554000	-0.862483000	2.213457000
C	2.729240000	0.910490000	2.504680000
C	3.490237000	-0.660709000	1.091285000
C	-1.698498000	1.803927000	-0.172194000
C	-0.860161000	2.506127000	-2.148023000
N	-0.619984000	1.754565000	-0.914362000
O	-2.760487000	2.451686000	-0.701215000
H	-2.596922000	3.918621000	-2.165161000
H	-2.989492000	2.267800000	-2.757916000
H	-0.217927000	3.405053000	-2.174992000
H	-0.612289000	1.893822000	-3.032465000
H	1.753840000	1.093423000	2.976616000
H	3.212349000	1.887027000	2.327240000
H	3.030741000	-0.769004000	3.904809000
H	4.415884000	0.383228000	3.865102000
H	4.546375000	-3.140807000	1.100061000
Br	0.568635000	-1.399639000	-0.933355000
C	-1.353561000	-1.559089000	-0.759744000
C	-2.166196000	-0.898170000	-1.687909000
C	-1.874428000	-2.194673000	0.373901000
H	-1.722753000	-0.424102000	-2.567234000
C	-3.250462000	-2.121687000	0.603363000
H	-1.211804000	-2.699207000	1.079521000

C	-4.085984000	-1.425939000	-0.291154000
H	-3.697141000	-2.572563000	1.493554000
C	-5.532168000	-1.324111000	0.049928000
O	-6.230320000	-0.612908000	-0.874948000
O	-6.049717000	-1.805947000	1.043259000
C	-7.631119000	-0.461112000	-0.591827000
H	-8.040453000	0.129602000	-1.423114000
H	-8.121687000	-1.447047000	-0.533022000
H	-7.774738000	0.062454000	0.368092000
C	-3.542894000	-0.831658000	-1.446040000
H	-4.203001000	-0.302197000	-2.137585000

TS(V-VI)

Lowest frequency = -142.8218 cm^{-1}

Charge = 0, Multiplicity = 1

65

Ru	0.895298000	0.847309000	0.188102000
C	-1.491458000	2.484186000	0.529490000
C	-2.748283000	2.945050000	0.968739000
C	-0.946420000	1.211194000	0.910249000
C	-3.521851000	2.124941000	1.792371000
C	-1.777713000	0.406674000	1.721582000
C	-3.034743000	0.852993000	2.151925000
H	-4.505458000	2.457387000	2.139366000
H	-1.466627000	-0.610013000	1.981214000
H	-3.656755000	0.191290000	2.766388000
C	0.103302000	4.556054000	-2.027973000
C	1.001703000	-1.875124000	3.521275000
N	1.336298000	-0.700011000	1.511691000
C	2.553922000	-2.524822000	0.310373000
C	3.392453000	-1.755017000	-0.522006000
C	2.430894000	-3.914836000	0.098164000
C	4.081614000	-2.374652000	-1.569978000
C	3.109712000	-4.520575000	-0.964041000
C	3.934525000	-3.751233000	-1.800180000
H	4.737347000	-1.777339000	-2.211845000
H	2.999732000	-5.595823000	-1.138677000
H	4.469344000	-4.228557000	-2.628308000
O	1.737184000	2.053214000	1.640731000
C	2.946564000	2.048225000	1.179276000
O	3.202613000	1.405961000	0.121450000
C	4.013711000	2.790765000	1.946611000
H	3.565976000	3.525841000	2.632083000
H	4.601424000	2.064371000	2.536510000
H	4.703742000	3.284970000	1.244234000
H	-3.109599000	3.927341000	0.645739000
H	3.504614000	-0.683377000	-0.328041000
O	1.814592000	-2.636206000	2.580714000
C	1.074201000	-0.457996000	2.961343000
C	1.864816000	-1.893981000	1.439929000
C	-0.656934000	3.159918000	-0.433605000
C	1.112233000	3.395855000	-1.822028000
N	0.423301000	2.546262000	-0.846677000
O	-0.918453000	4.356411000	-1.000438000
H	0.534633000	5.557923000	-1.879337000
H	-0.402339000	4.517805000	-3.007922000
H	2.081389000	3.731360000	-1.411334000

H	1.314387000	2.831545000	-2.747857000
H	0.160316000	0.132311000	3.101169000
H	1.921757000	0.119962000	3.370000000
H	-0.022600000	-2.291568000	3.498521000
H	1.435708000	-2.003925000	4.523039000
H	1.796187000	-4.505587000	0.765238000
Br	0.940265000	-0.352844000	-2.157383000
C	-0.780927000	-0.628267000	-1.106875000
C	-1.901603000	0.113746000	-1.553142000
C	-0.953407000	-1.893231000	-0.502944000
H	-1.753608000	1.061312000	-2.076548000
C	-2.243541000	-2.334424000	-0.217832000
H	-0.084445000	-2.504474000	-0.251904000
C	-3.373734000	-1.569845000	-0.584356000
H	-2.406018000	-3.288597000	0.293624000
C	-3.183293000	-0.349468000	-1.270960000
H	-4.053823000	0.240635000	-1.567739000
C	-4.709366000	-2.077729000	-0.210677000
O	-5.715772000	-1.259319000	-0.638428000
C	-7.035727000	-1.693623000	-0.285796000
H	-7.247218000	-2.687001000	-0.716816000
H	-7.143679000	-1.758858000	0.810443000
H	-7.719395000	-0.937879000	-0.698253000
O	-4.924972000	-3.104503000	0.418505000

Ru-VI
Lowest frequency = 16.3375 cm^{-1}
Charge = 0, Multiplicity = 1

65

Ru	0.665836000	0.714211000	0.057029000
C	-1.135485000	3.060635000	0.313092000
C	-2.002328000	4.037919000	0.842488000
C	-0.874649000	1.820410000	0.971088000
C	-2.641186000	3.795548000	2.057334000
C	-1.575950000	1.593962000	2.168461000
C	-2.425943000	2.567600000	2.710061000
H	-3.317787000	4.540157000	2.488207000
H	-1.516278000	0.626418000	2.672095000
H	-2.950311000	2.351735000	3.647755000
C	0.233624000	3.821790000	-2.986687000
C	1.423295000	-1.751709000	3.621278000
N	1.362490000	-0.702218000	1.496113000
C	2.788670000	-2.449233000	0.341660000
C	3.558145000	-1.708012000	-0.575696000
C	2.757225000	-3.857168000	0.261458000
C	4.282353000	-2.377343000	-1.567986000
C	3.461708000	-4.514758000	-0.751390000
C	4.227750000	-3.775021000	-1.665783000
H	4.881359000	-1.799031000	-2.278603000
H	3.417122000	-5.606115000	-0.825584000
H	4.785583000	-4.291399000	-2.454459000
O	1.667486000	2.036126000	1.432671000
C	2.775325000	2.007818000	0.783927000
O	2.811211000	1.320419000	-0.284621000
C	3.983179000	2.735855000	1.308322000
H	4.639438000	2.012067000	1.824451000
H	4.553482000	3.169393000	0.471613000

H	3.685295000	3.518799000	2.021398000
H	-2.158889000	4.969856000	0.289769000
H	3.585996000	-0.618472000	-0.504552000
O	2.242134000	-2.438026000	2.637257000
C	1.090997000	-0.426187000	2.931417000
C	2.080539000	-1.794066000	1.451772000
C	-0.448436000	3.178624000	-0.944244000
C	0.948028000	2.504985000	-2.591250000
N	0.313046000	2.183104000	-1.306544000
O	-0.530637000	4.214981000	-1.796546000
H	0.916328000	4.650344000	-3.228780000
H	-0.490141000	3.689501000	-3.808276000
H	2.035330000	2.619889000	-2.441044000
H	0.786006000	1.681879000	-3.304625000
H	0.046973000	-0.124332000	3.067668000
H	1.726604000	0.416086000	3.252013000
H	0.532414000	-2.375187000	3.815413000
H	2.013664000	-1.655129000	4.544324000
H	2.167153000	-4.424341000	0.986724000
Br	0.793468000	-0.812155000	-1.930795000
C	-1.200804000	-0.300333000	0.036422000
C	-2.200884000	0.085737000	-0.887066000
C	-1.483429000	-1.393788000	0.881199000
H	-2.006089000	0.917220000	-1.570788000
C	-2.715822000	-2.053341000	0.825052000
H	-0.734951000	-1.759367000	1.586284000
C	-3.709185000	-1.645646000	-0.082522000
H	-2.928990000	-2.902764000	1.482476000
C	-3.431159000	-0.567044000	-0.949196000
H	-4.190535000	-0.246741000	-1.668061000
C	-4.998885000	-2.375647000	-0.087574000
O	-5.867265000	-1.890139000	-1.022427000
C	-7.133496000	-2.560949000	-1.073950000
H	-7.655493000	-2.481997000	-0.105048000
H	-7.706616000	-2.057548000	-1.865702000
H	-6.997929000	-3.629922000	-1.311141000
O	-5.287285000	-3.308476000	0.647153000

AcOH

Lowest frequency = 79.7446 cm^{-1}
Charge = 0, Multiplicity = 1

8

C	-0.092792000	0.121673000	-0.000005000
O	-0.637637000	1.192942000	-0.000033000
O	-0.776657000	-1.033953000	-0.000017000
H	-1.715791000	-0.792741000	-0.000050000
C	1.388081000	-0.110577000	0.000048000
H	1.673345000	-0.698450000	0.884681000
H	1.673400000	-0.698526000	-0.884515000
H	1.911661000	0.851229000	0.000025000

Methyl 4-bromobenzoate

Lowest frequency = 55.7747 cm^{-1}
Charge = 0, Multiplicity = 1

18

Br	3.323536000	-0.116445000	0.000141000
C	1.422922000	0.010131000	0.000040000
C	0.657131000	-1.166131000	-0.000031000
C	0.819217000	1.277726000	0.000038000
H	1.149848000	-2.142004000	-0.000029000
C	-0.575745000	1.361128000	-0.000035000
H	1.435761000	2.180418000	0.000093000
C	-1.362942000	0.193926000	-0.000109000
H	-1.081527000	2.331403000	-0.000039000
C	-2.844894000	0.354147000	-0.000193000
O	-3.490149000	-0.840793000	-0.000131000
O	-3.427443000	1.425175000	-0.000087000
C	-4.925986000	-0.756459000	-0.000081000
H	-5.284142000	-1.795134000	-0.000287000
H	-5.279115000	-0.220000000	0.896210000
H	-5.279159000	-0.219609000	-0.896117000
C	-0.738299000	-1.068240000	-0.000104000
H	-1.353110000	-1.971941000	-0.000161000

8. NMR Spectra

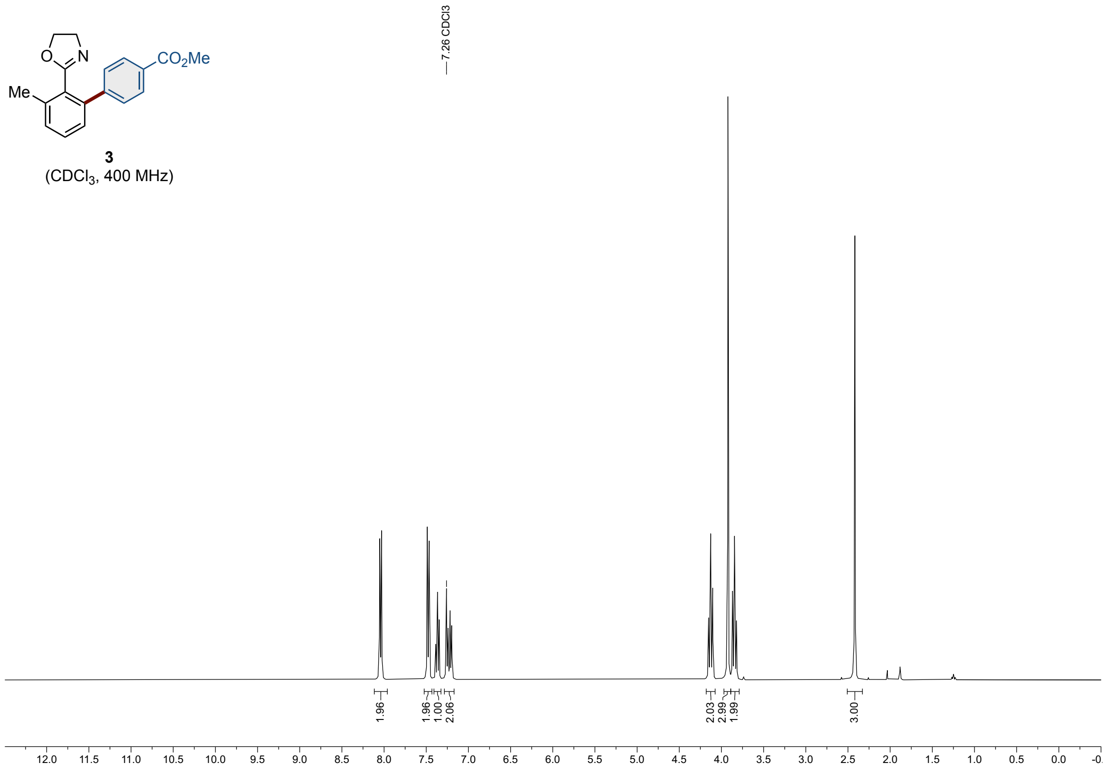

Figure S17. ^{1}H-NMR spectrum of **3**.

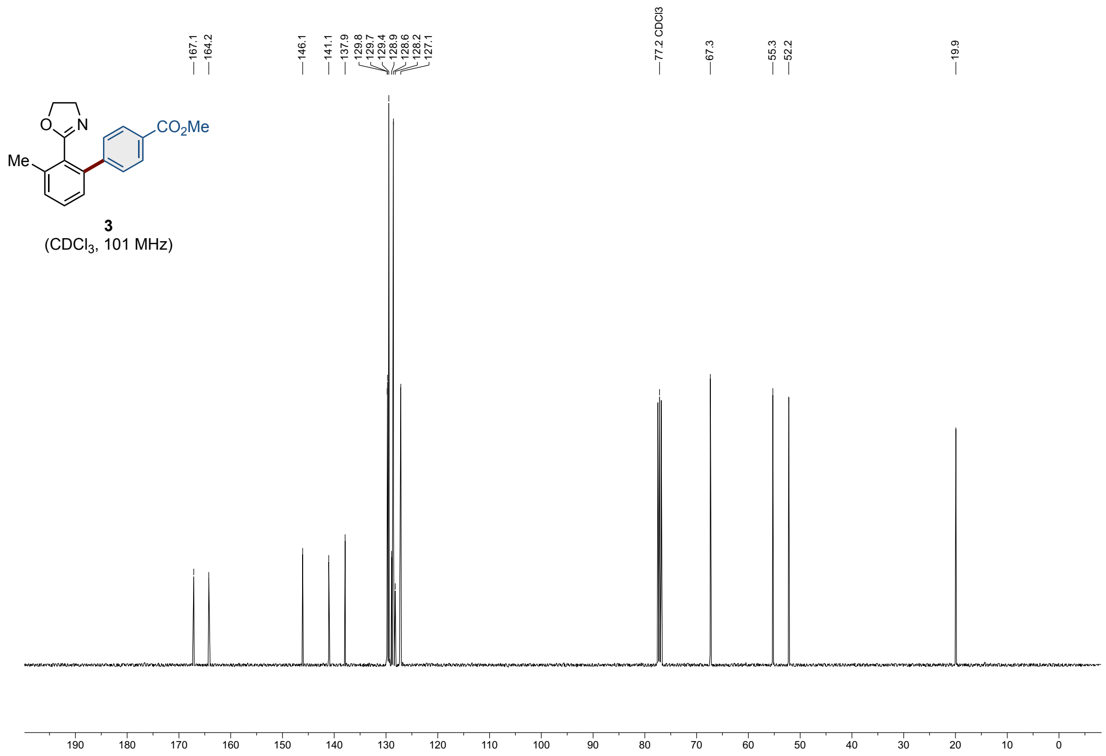

Figure S18. ^{13}C-NMR spectrum of **3**.

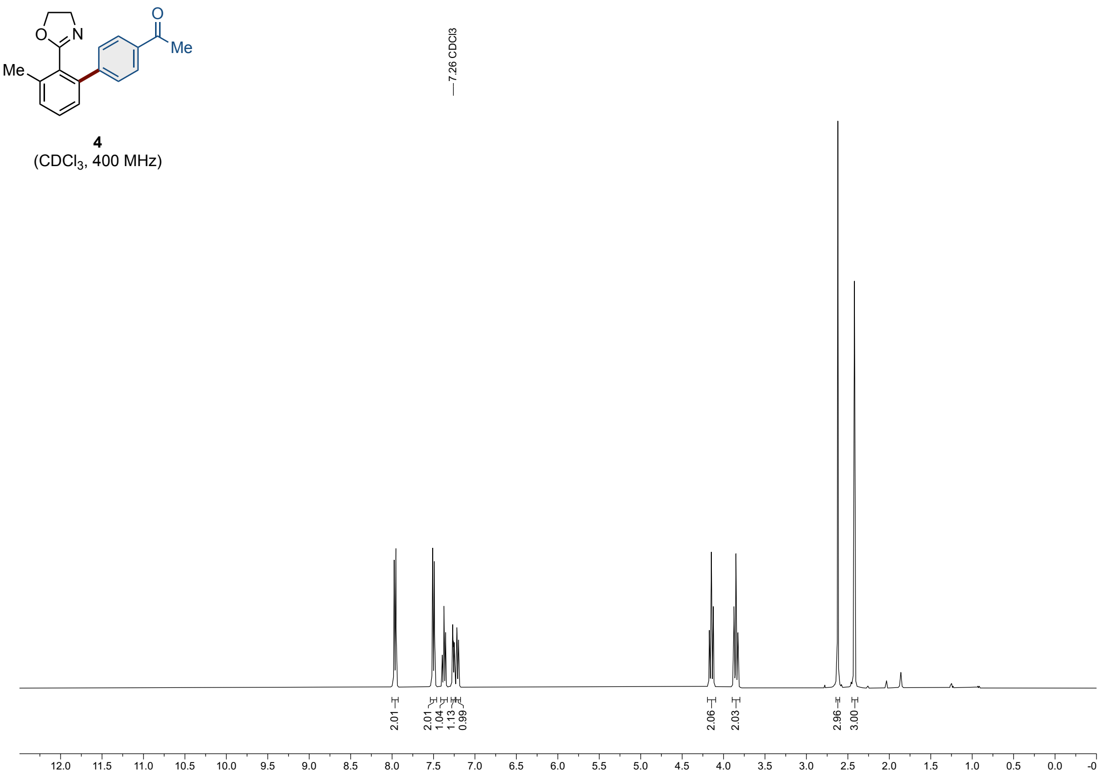

Figure S19. ^{1}H-NMR spectrum of **4**.

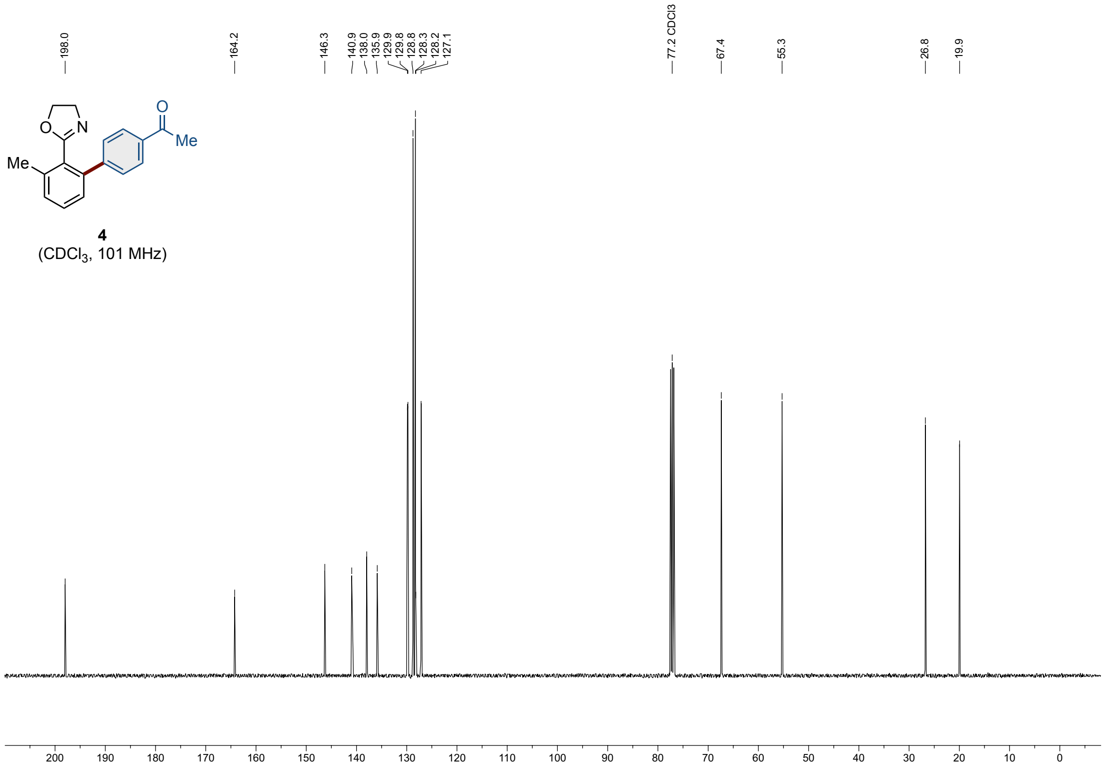

Figure S20. ^{13}C-NMR spectrum of **4**.

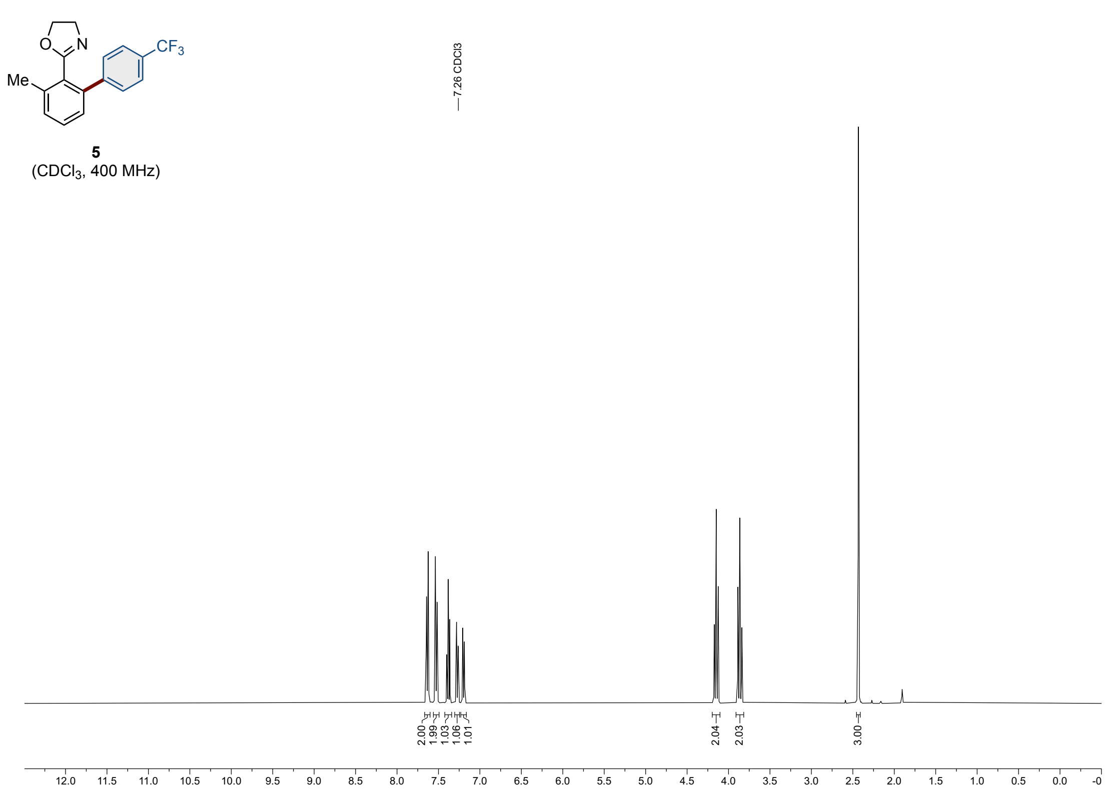

Figure S21. ¹H-NMR spectrum of **5**.

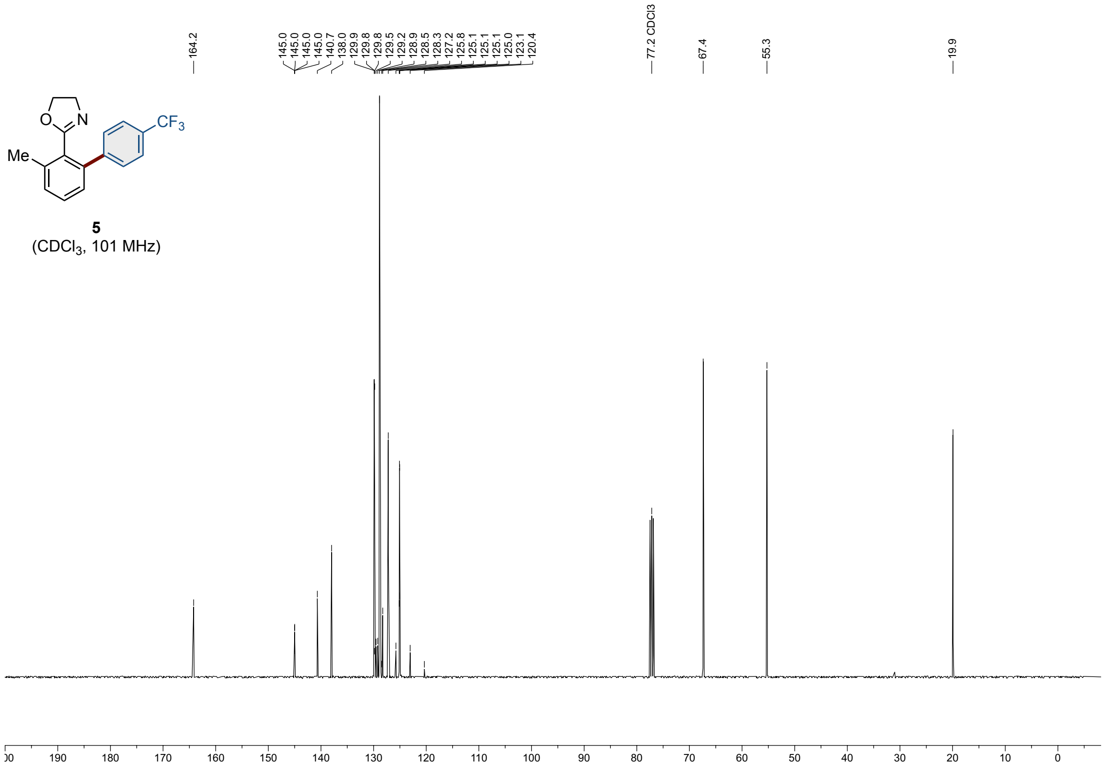

Figure S22. ¹³C-NMR spectrum of **5**.

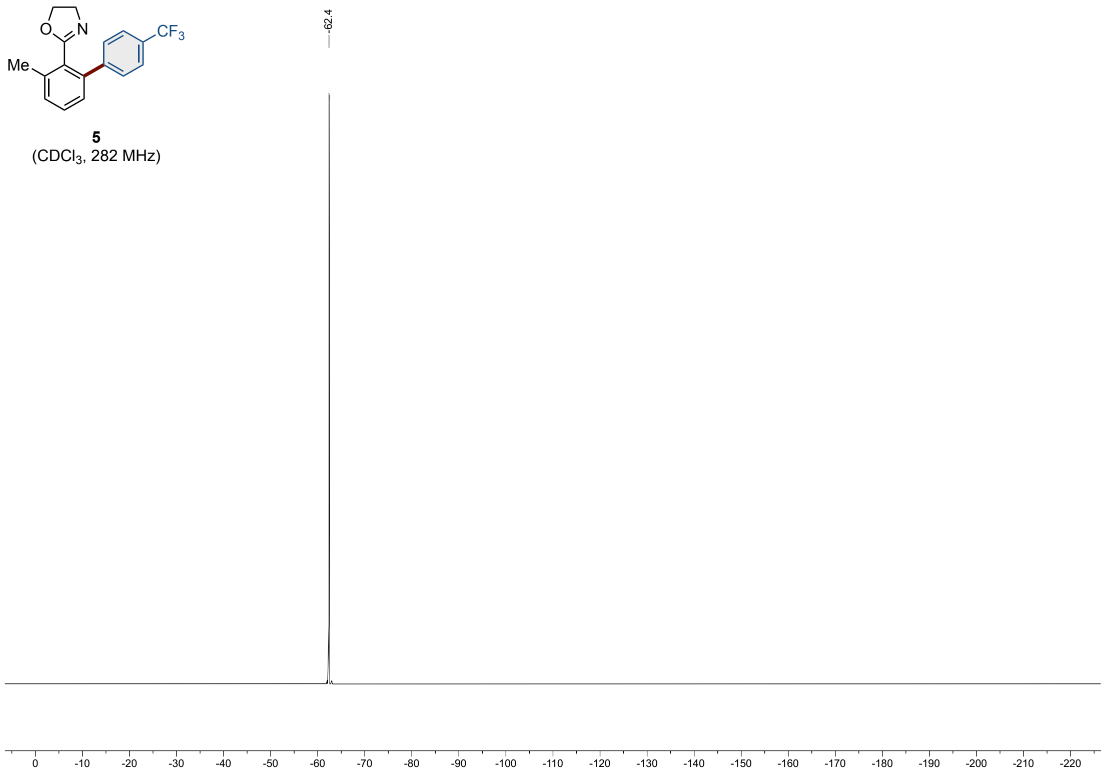

Figure S23. ^{19}F-NMR spectrum of **5**.

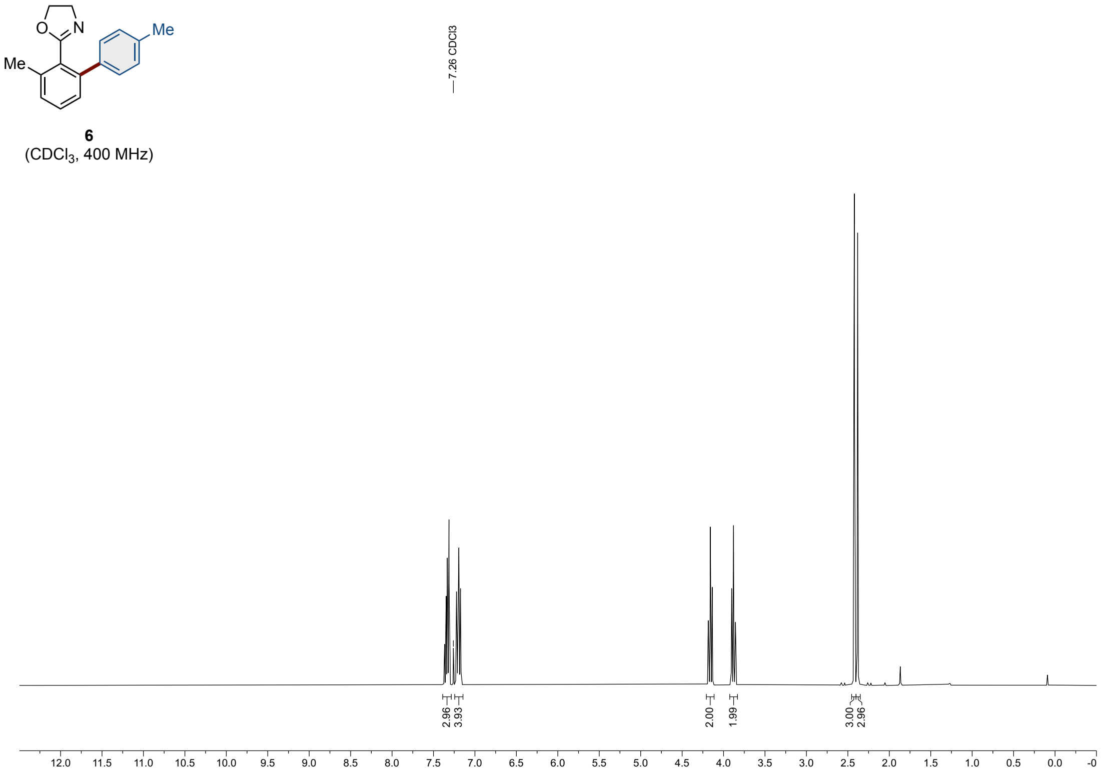

Figure S24. ^{1}H-NMR spectrum of **6**.

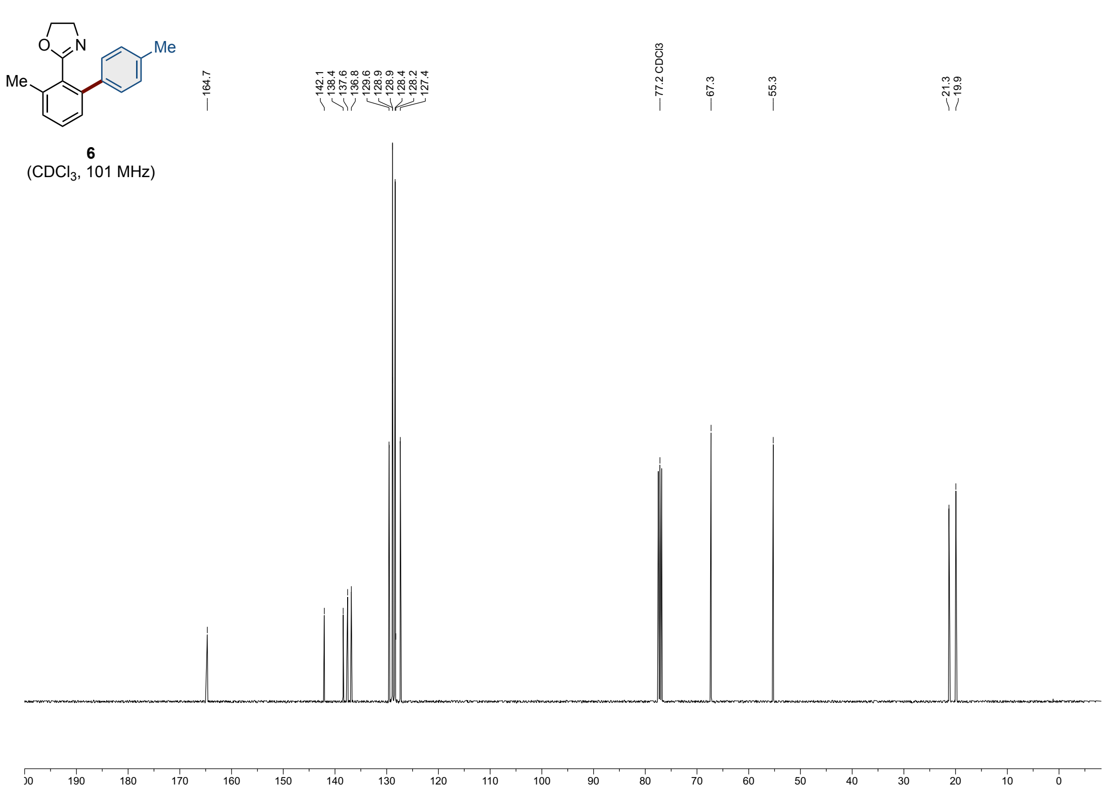

Figure S25. ^{13}C-NMR spectrum of **6**.

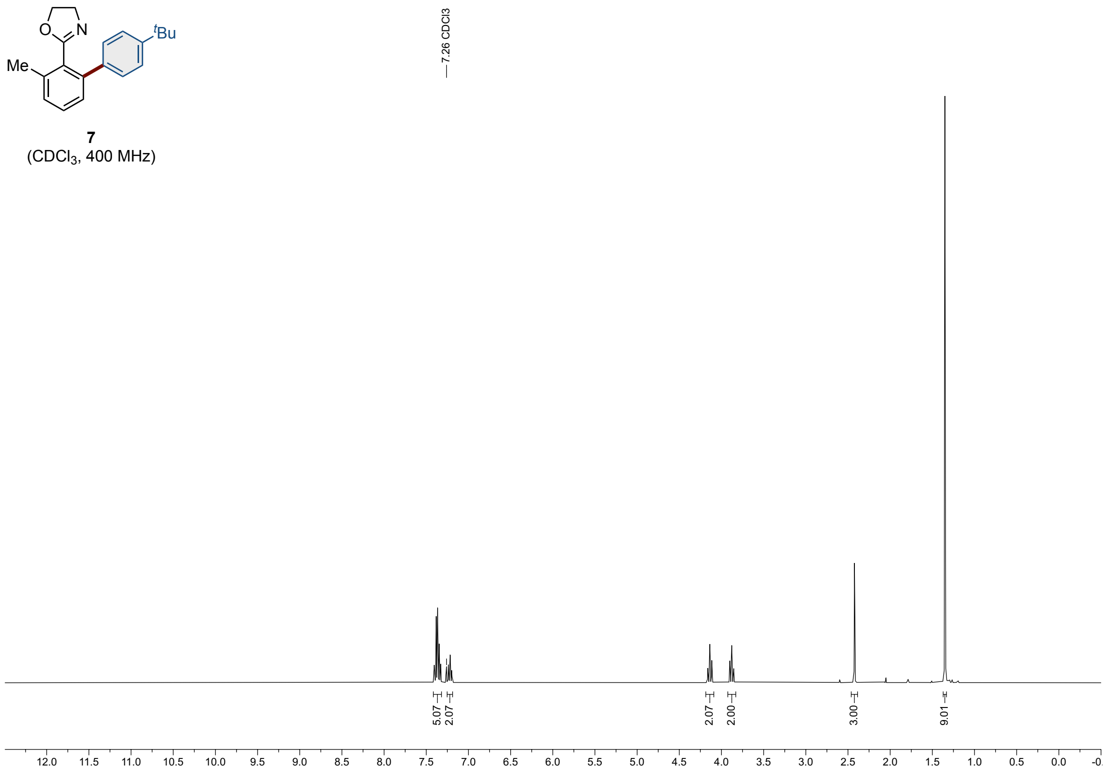

Figure S26. ^{1}H-NMR spectrum of **7**.

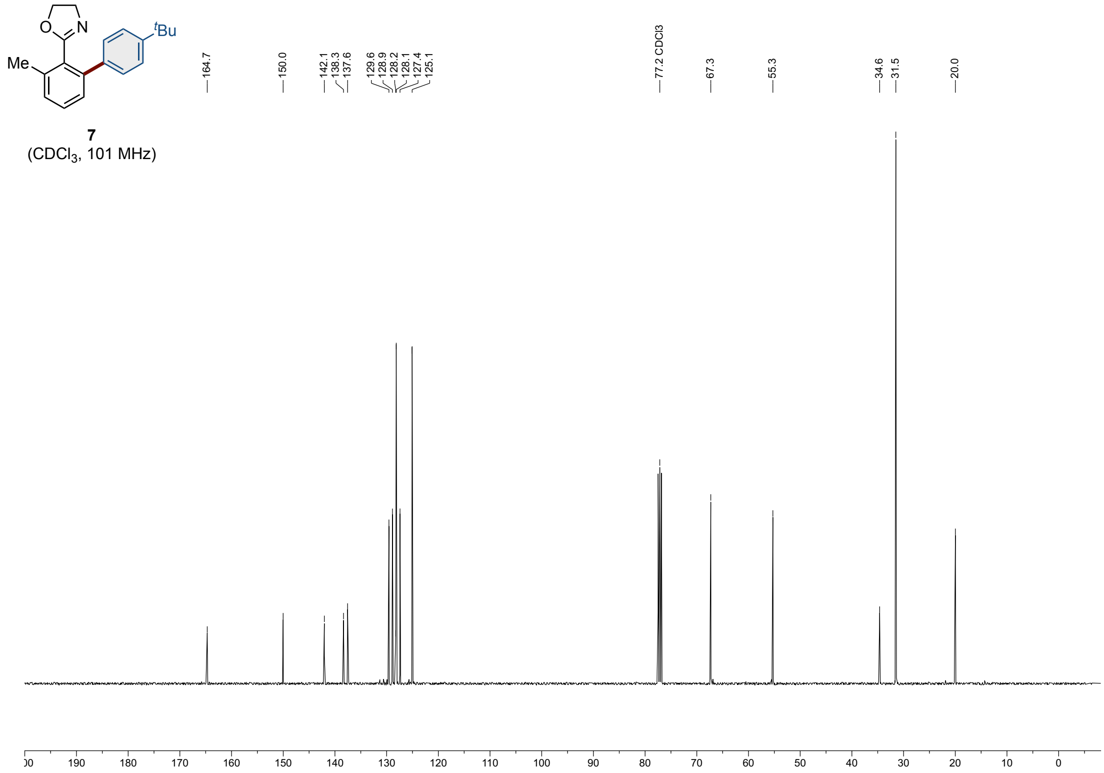

Figure S27. ^{13}C-NMR spectrum of **7**.

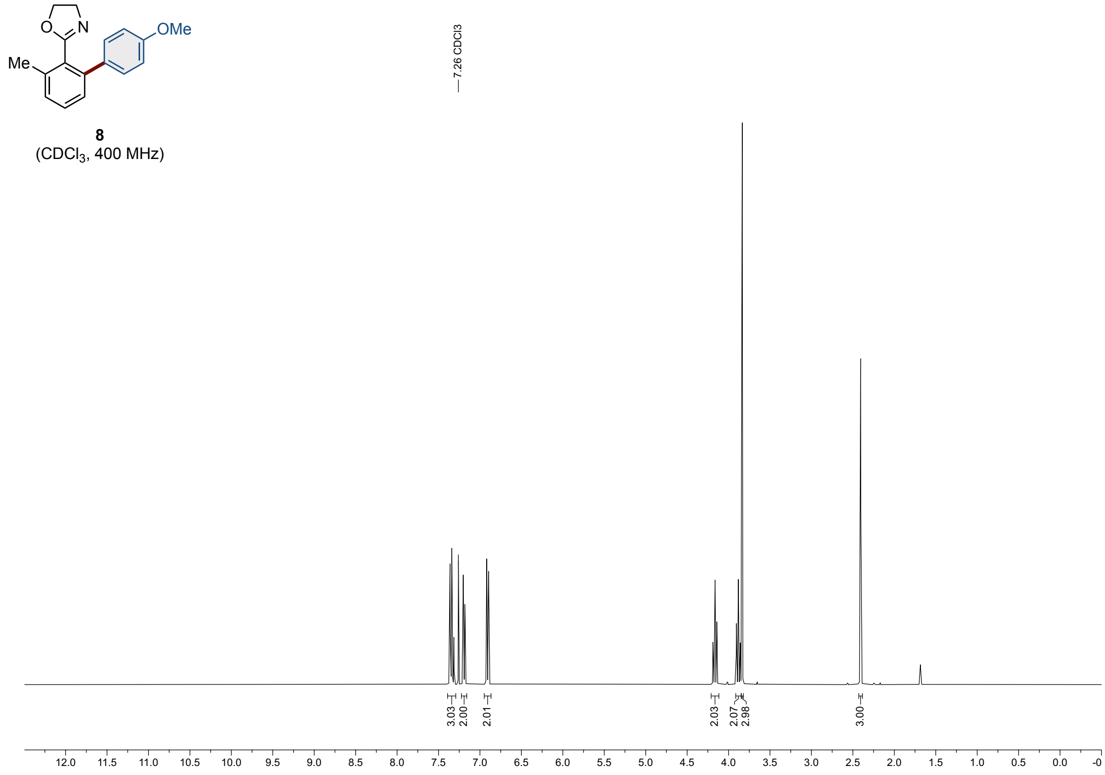

Figure S28. ^{1}H-NMR spectrum of **8**.

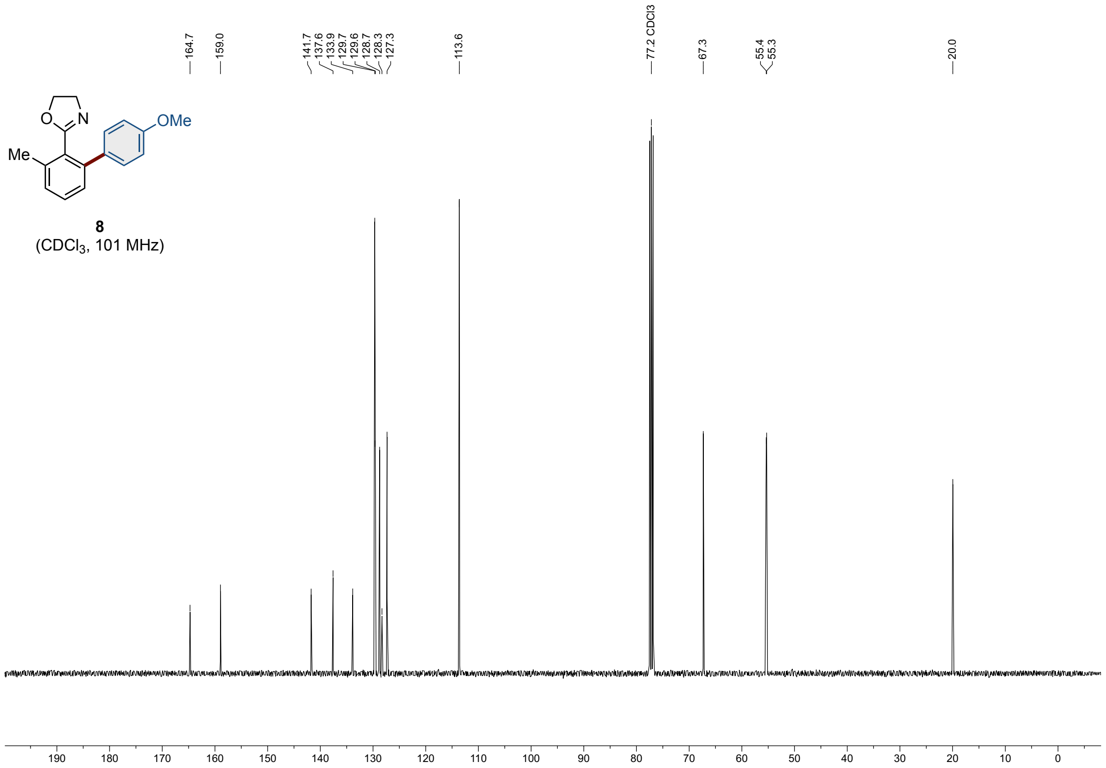

Figure S29. ^{13}C-NMR spectrum of **8**.

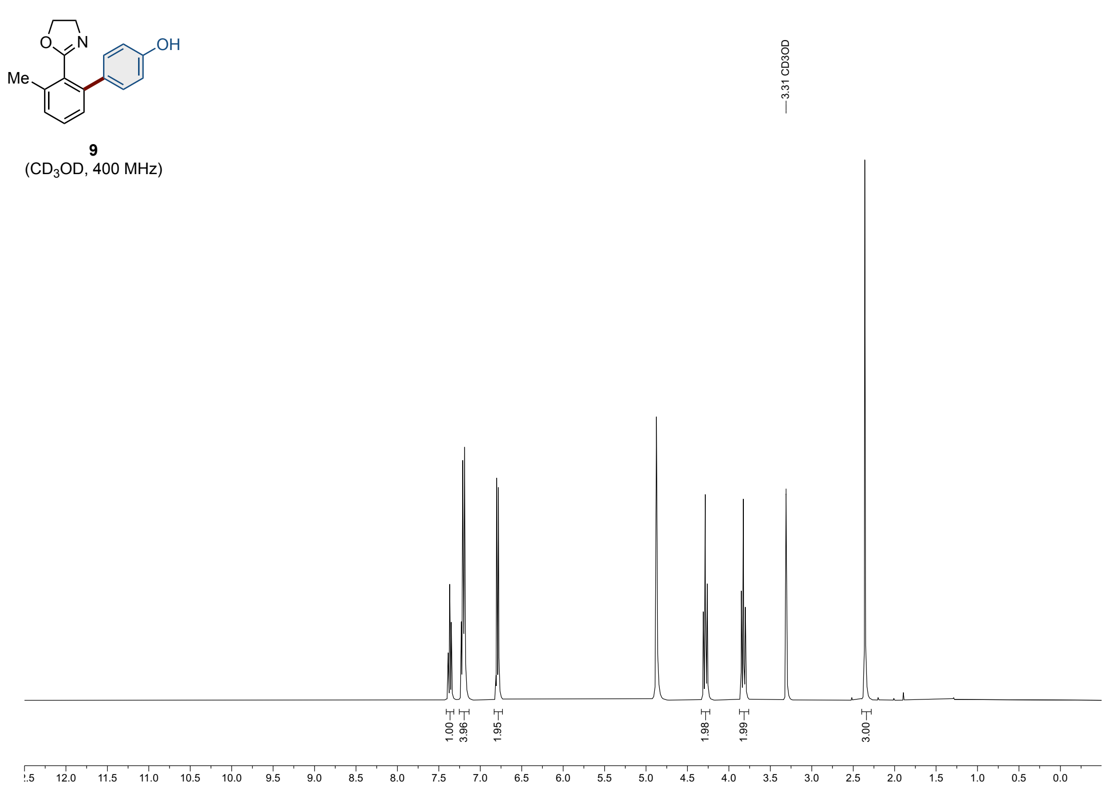

Figure S30. ^{1}H-NMR spectrum of **9**.

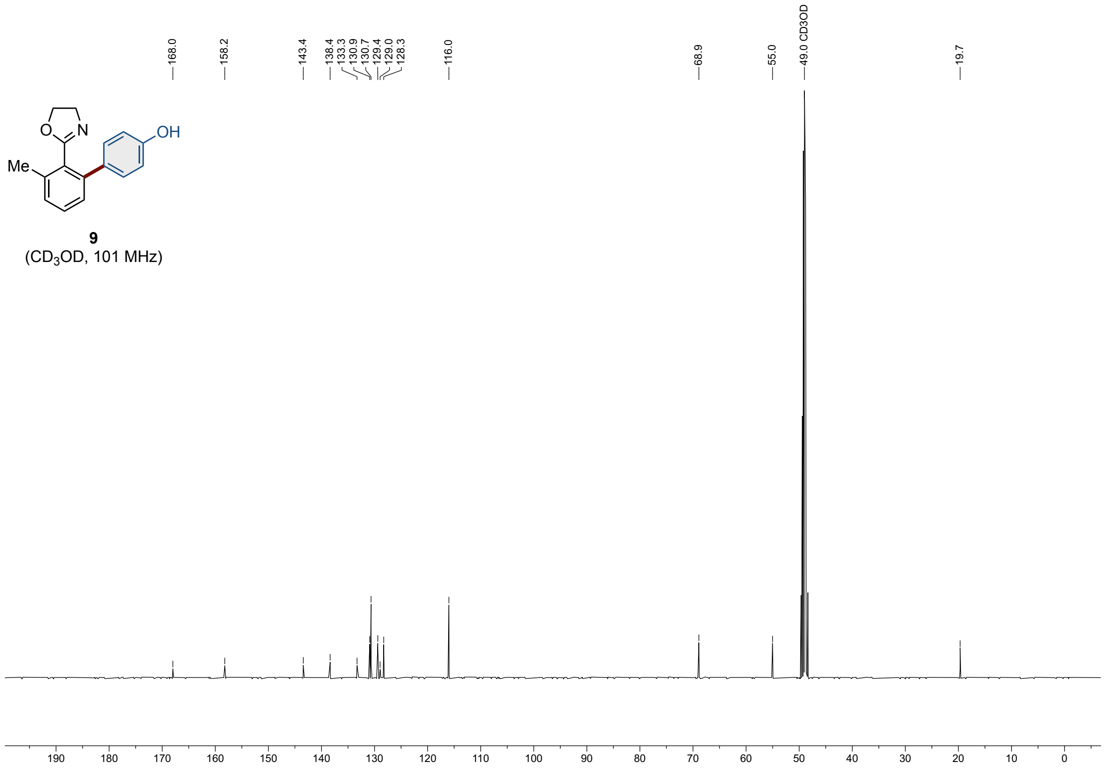

Figure S31. ^{13}C-NMR spectrum of **9**.

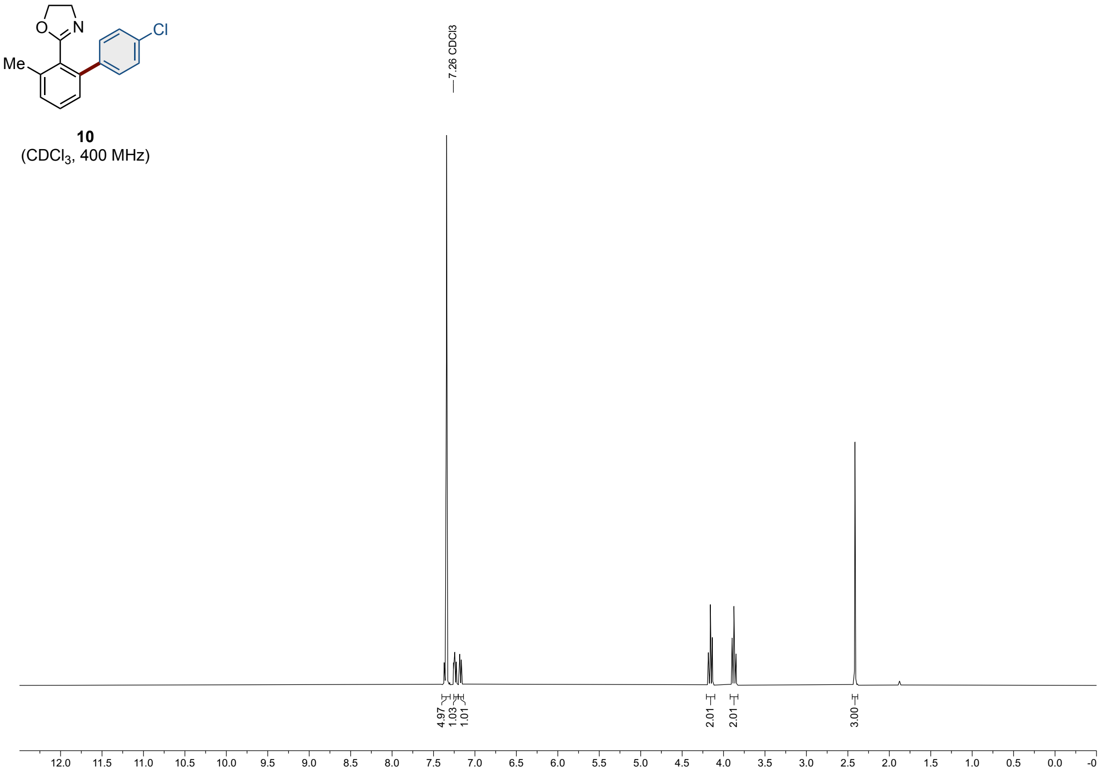

Figure S32. ^{1}H-NMR spectrum of **10**.

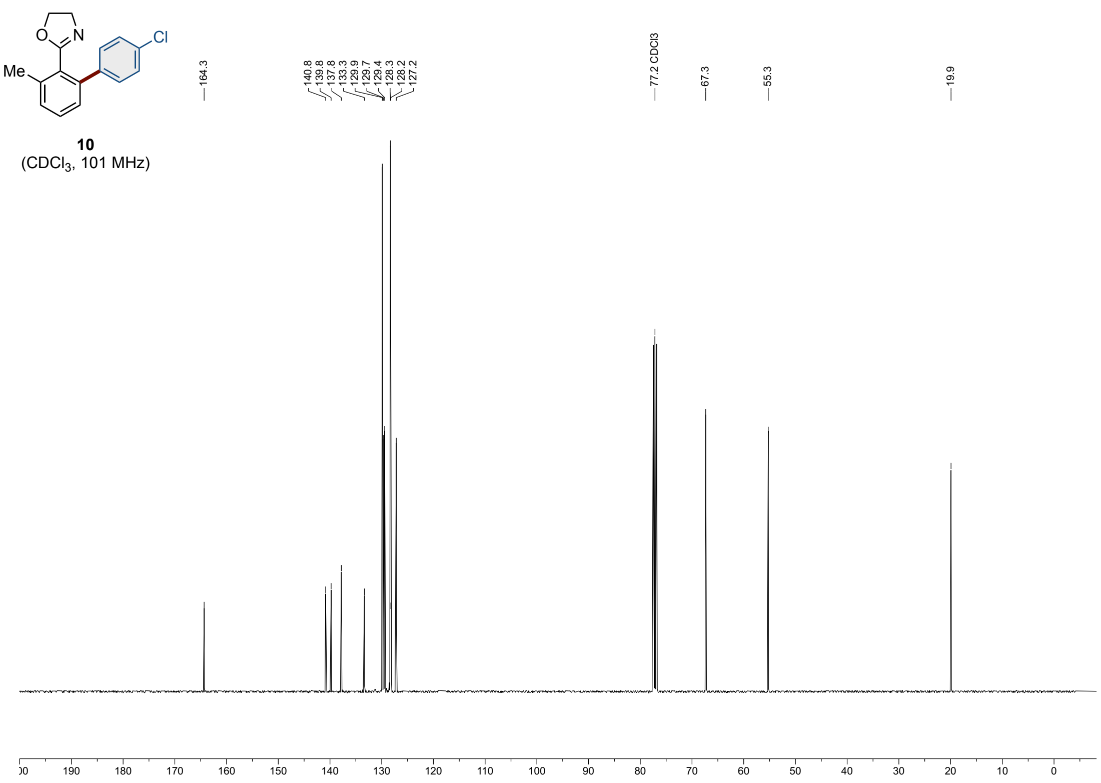

Figure S33. ^{13}C-NMR spectrum of **10**.

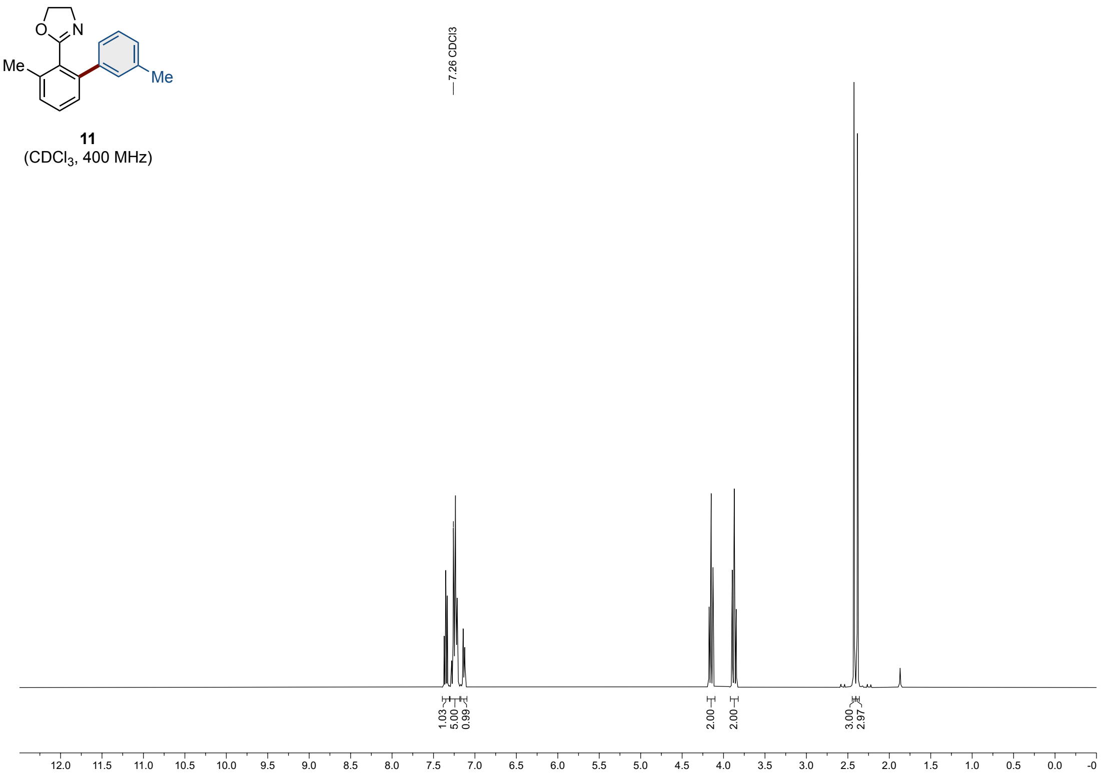

Figure S34. ^{1}H-NMR spectrum of **11**.

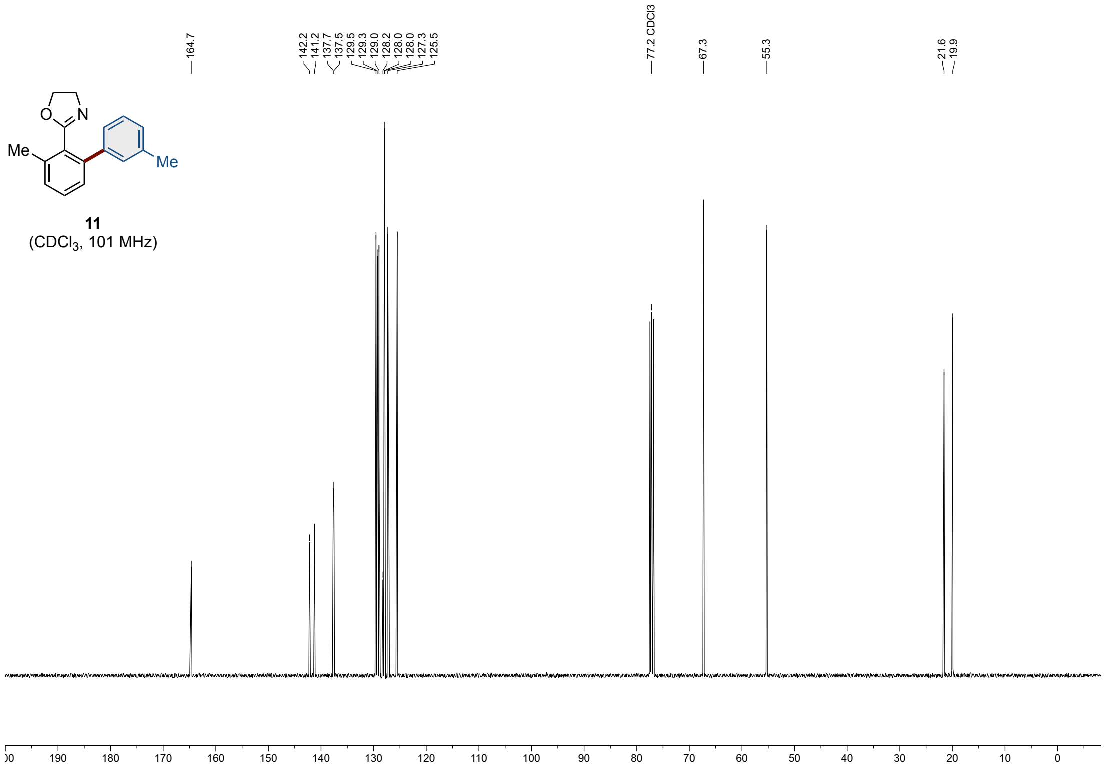

Figure S35. ^{13}C-NMR spectrum of **11**.

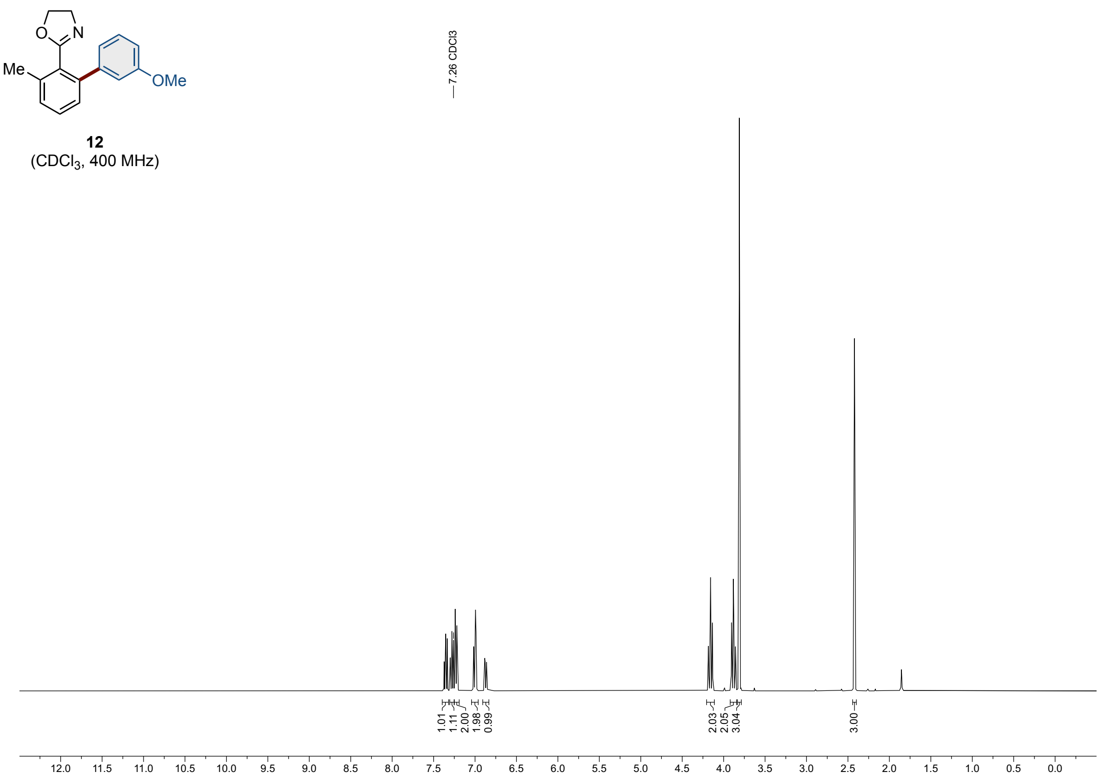

Figure S36. ^{1}H-NMR spectrum of **12**.

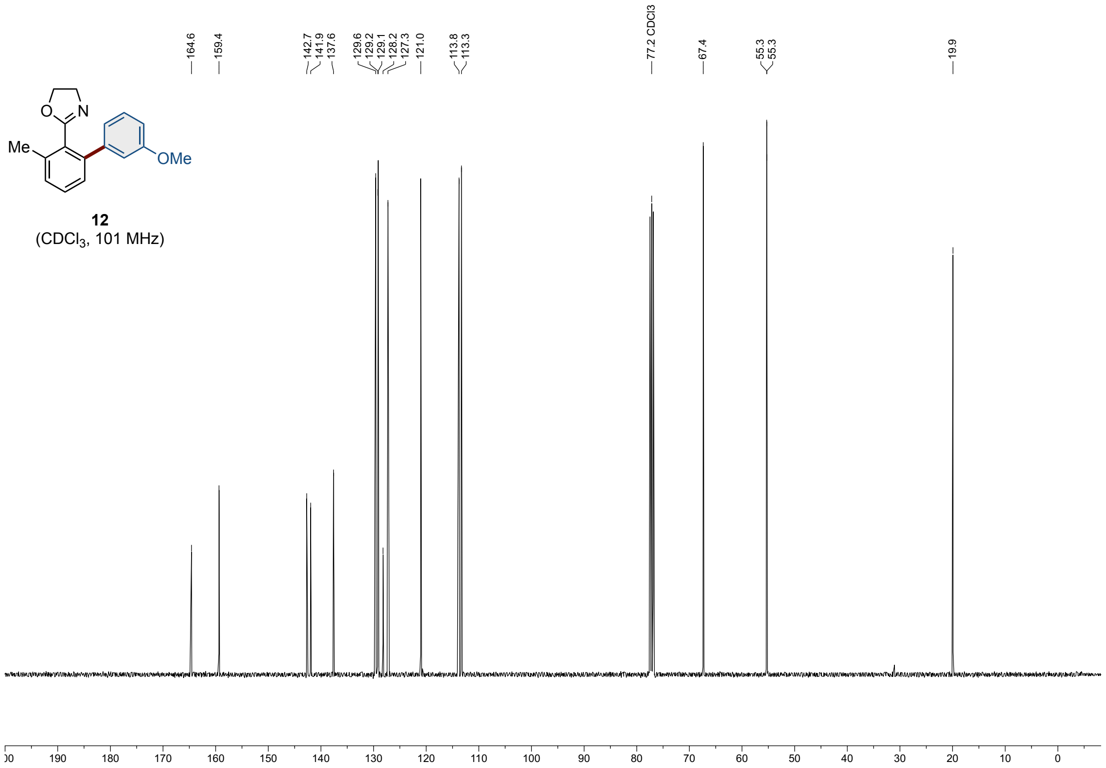

Figure S37. ^{13}C-NMR spectrum of **12**.

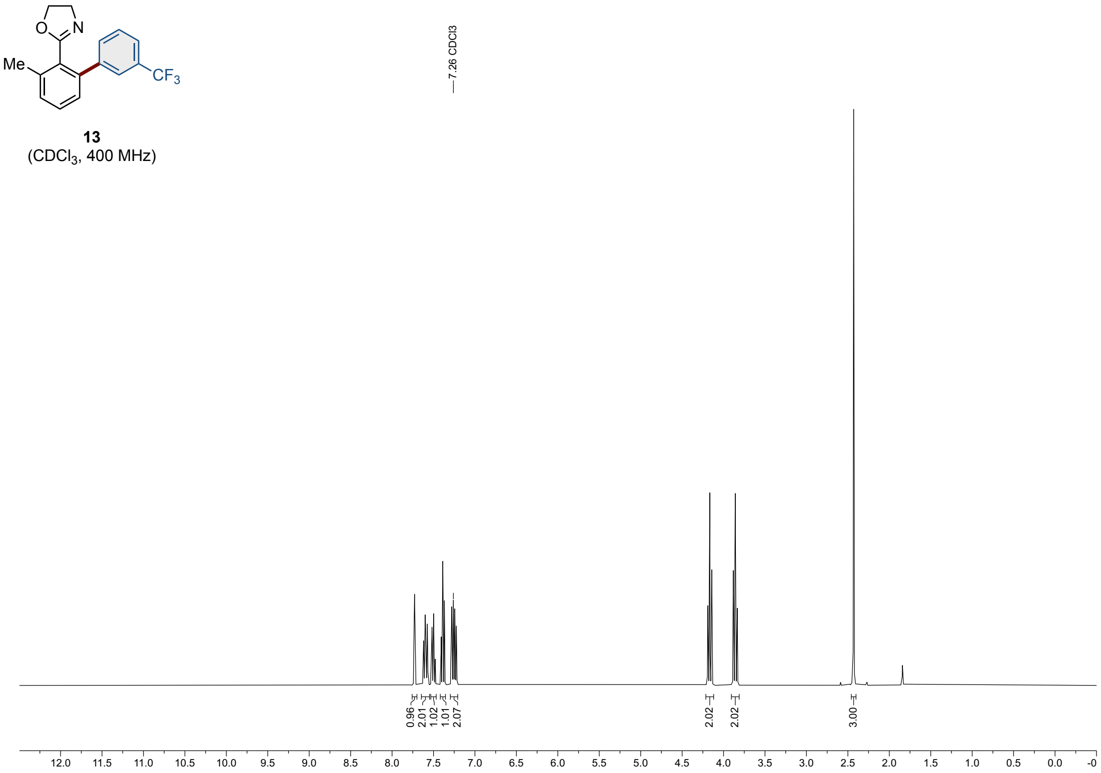

Figure S38. ^{1}H-NMR spectrum of **13**.

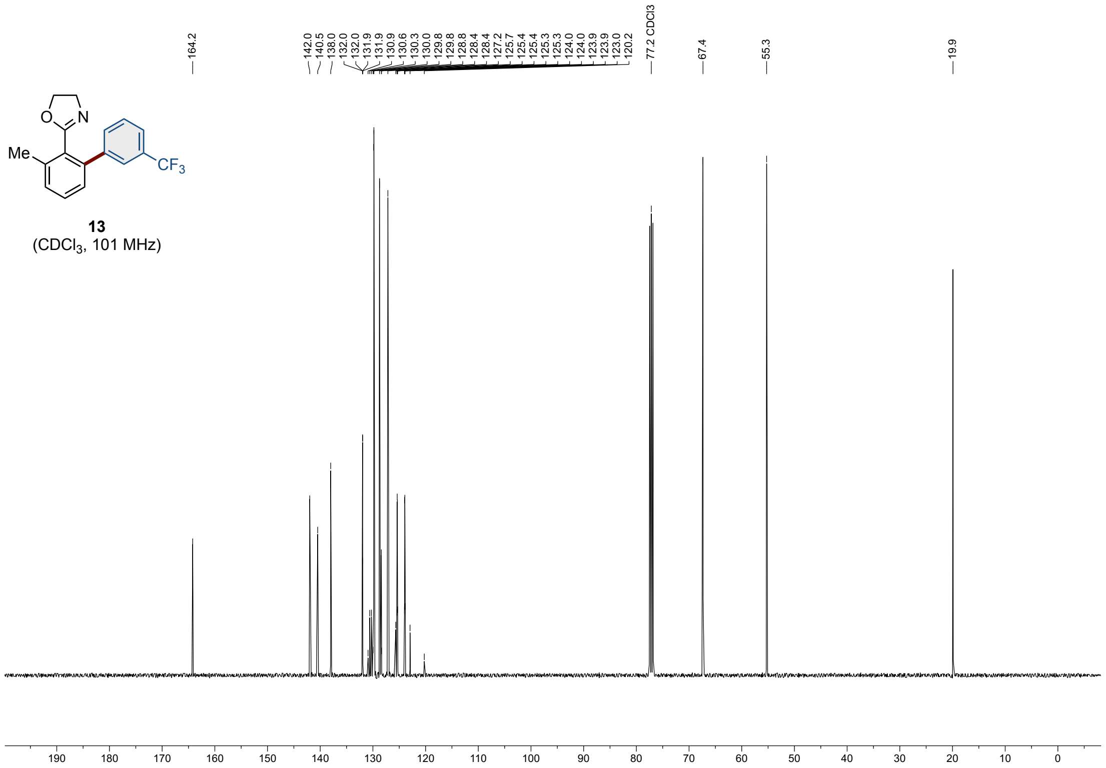

Figure S39. ^{13}C-NMR spectrum of **13**.

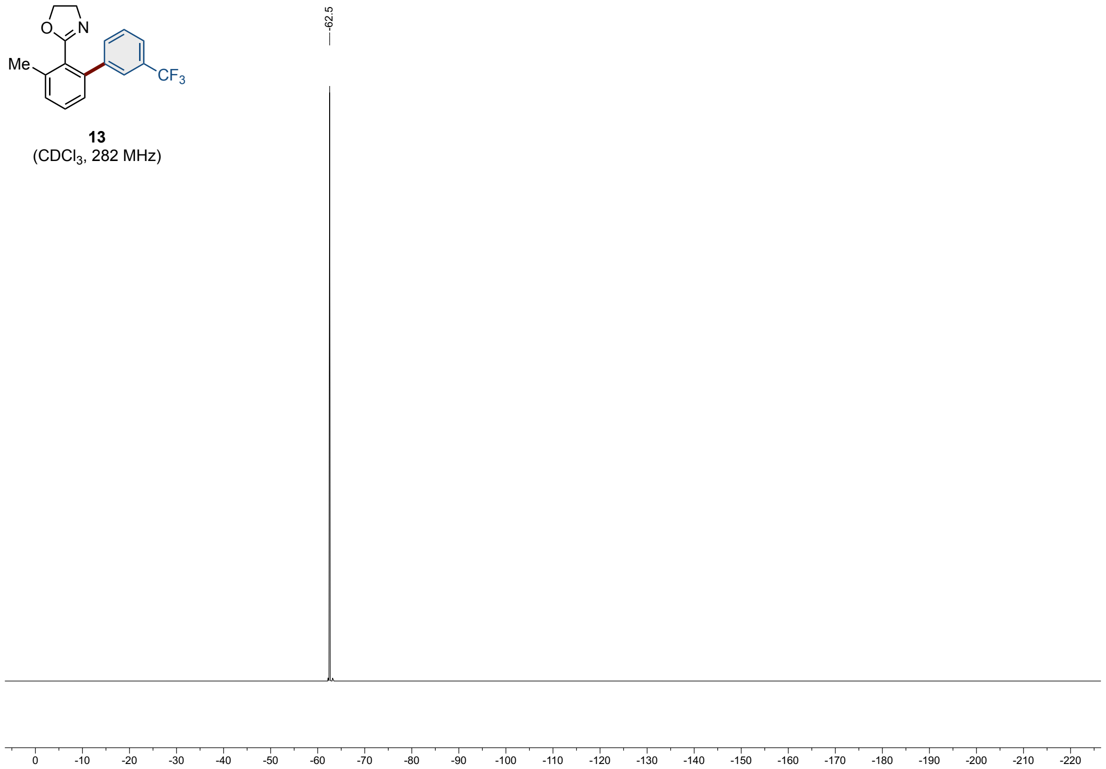

Figure S40. ^{19}F-NMR spectrum of **13**.

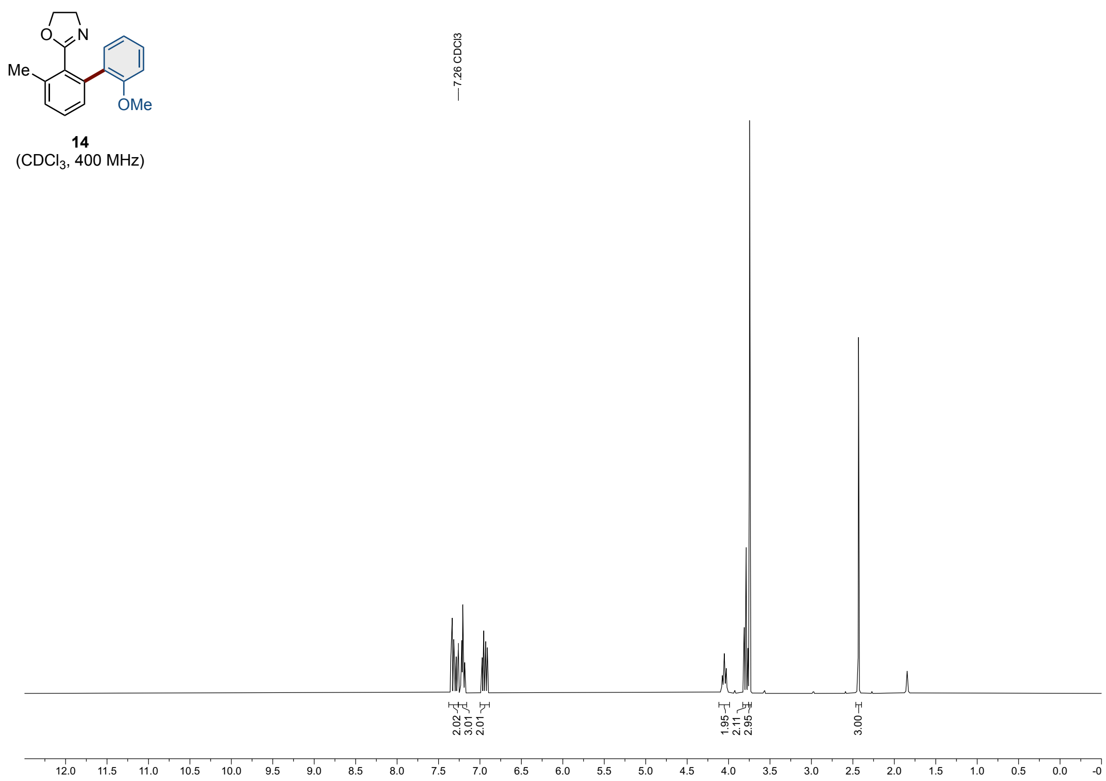

Figure S41. ^{1}H-NMR spectrum of **14**.

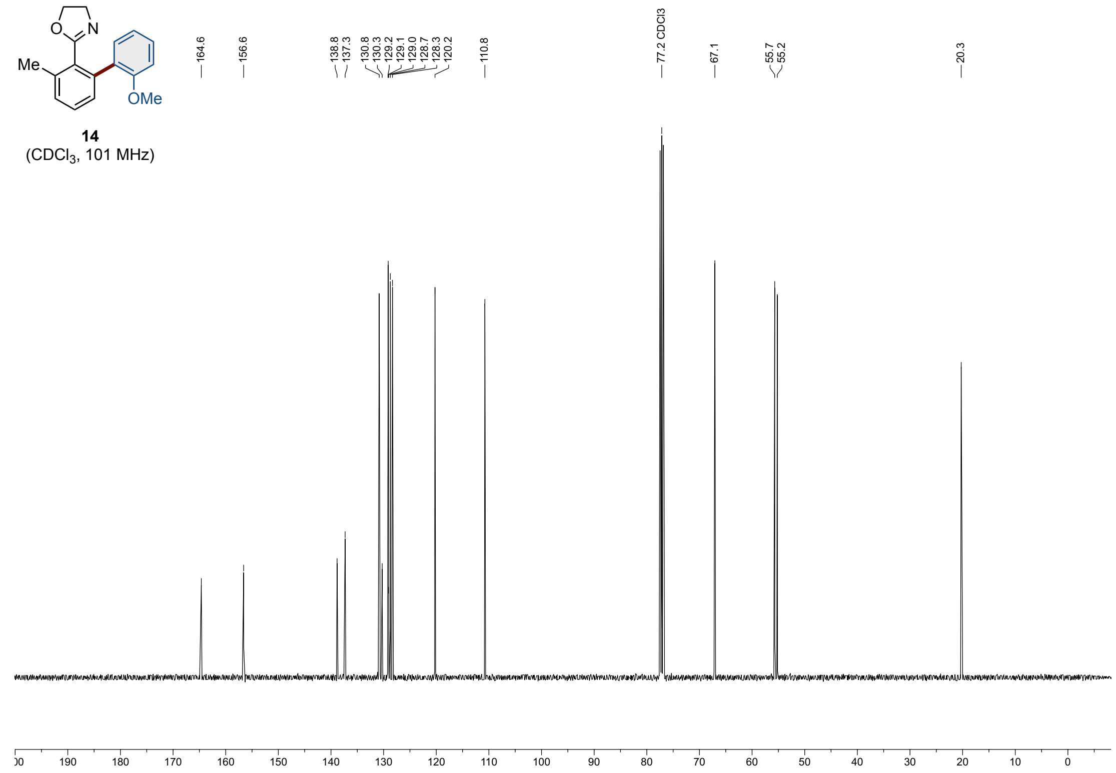

Figure S42. ^{13}C-NMR spectrum of **14**.

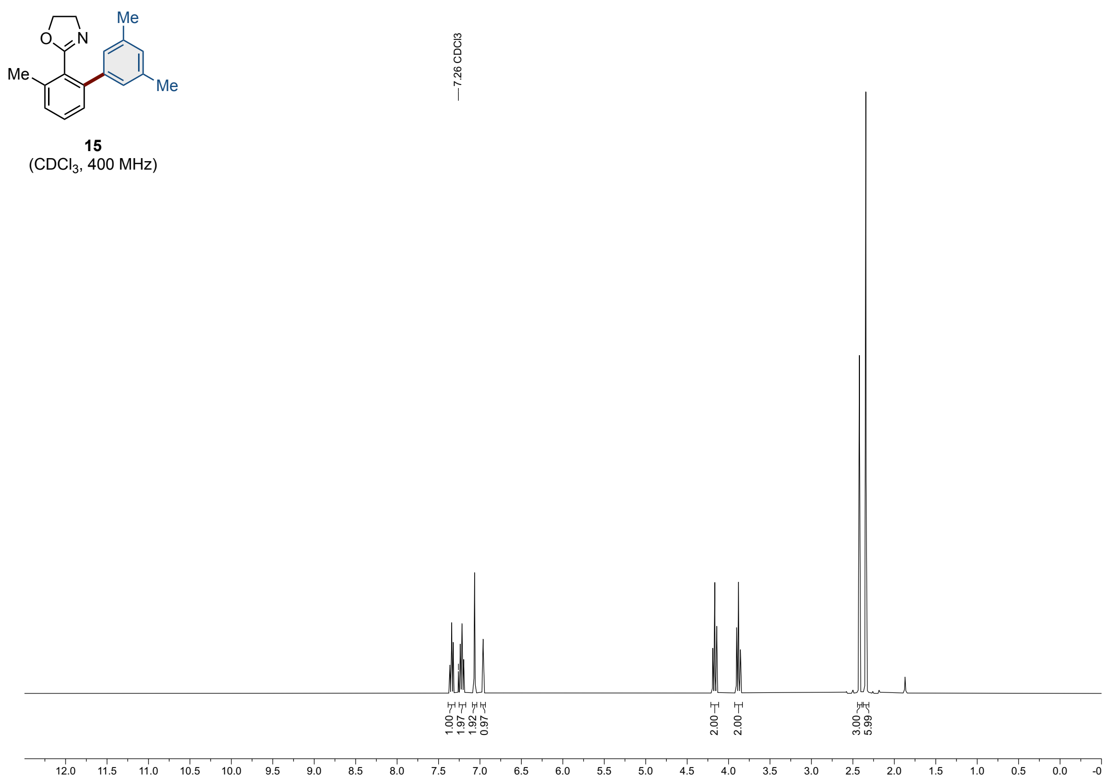

Figure S43. ^{1}H-NMR spectrum of **15**.

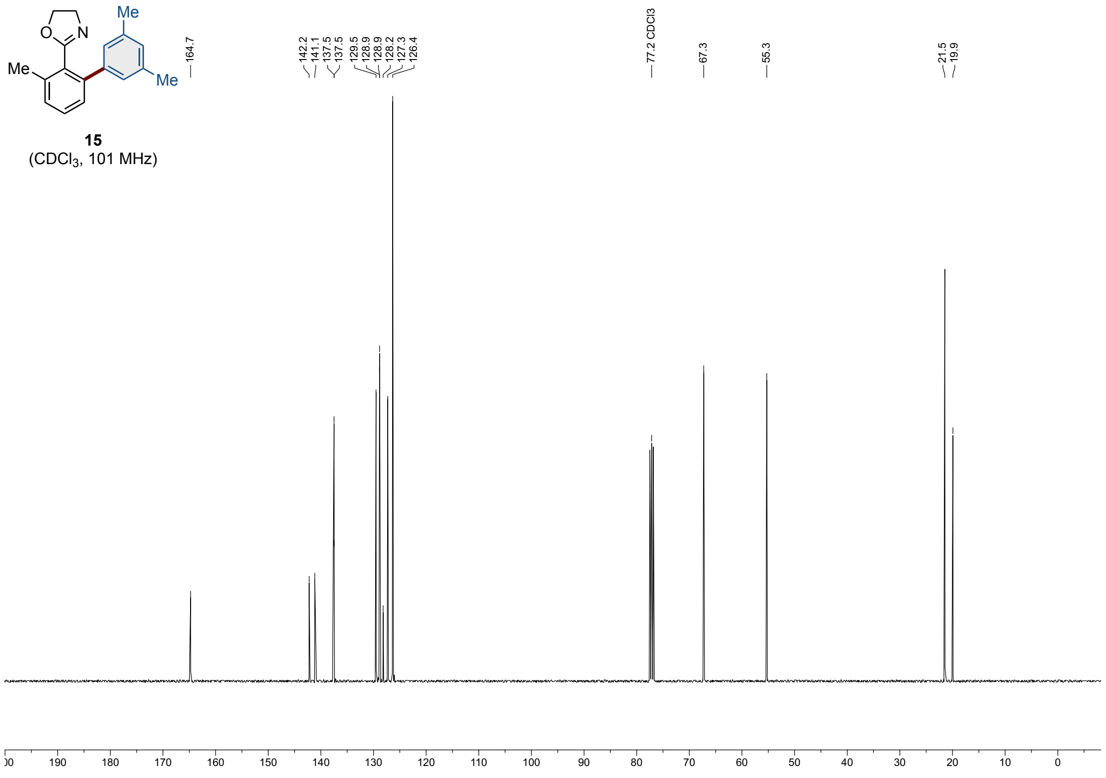

Figure S44. ^{13}C-NMR spectrum of **15**.

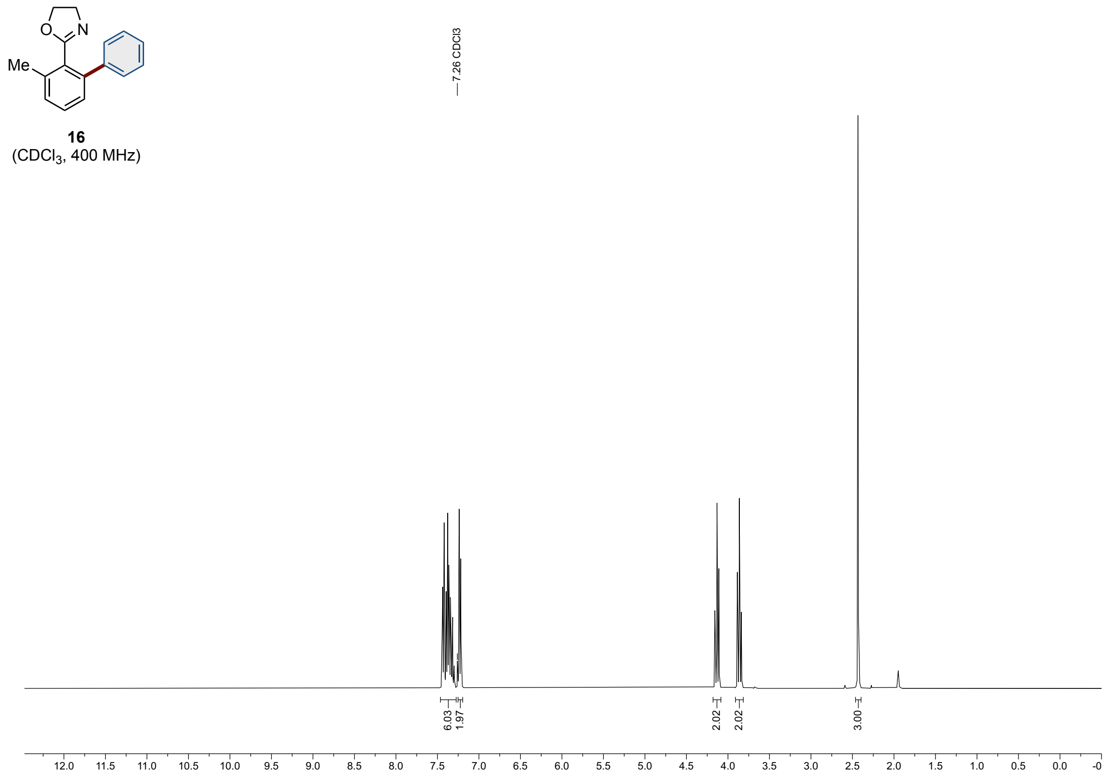

Figure S45. ^{1}H-NMR spectrum of **16**.

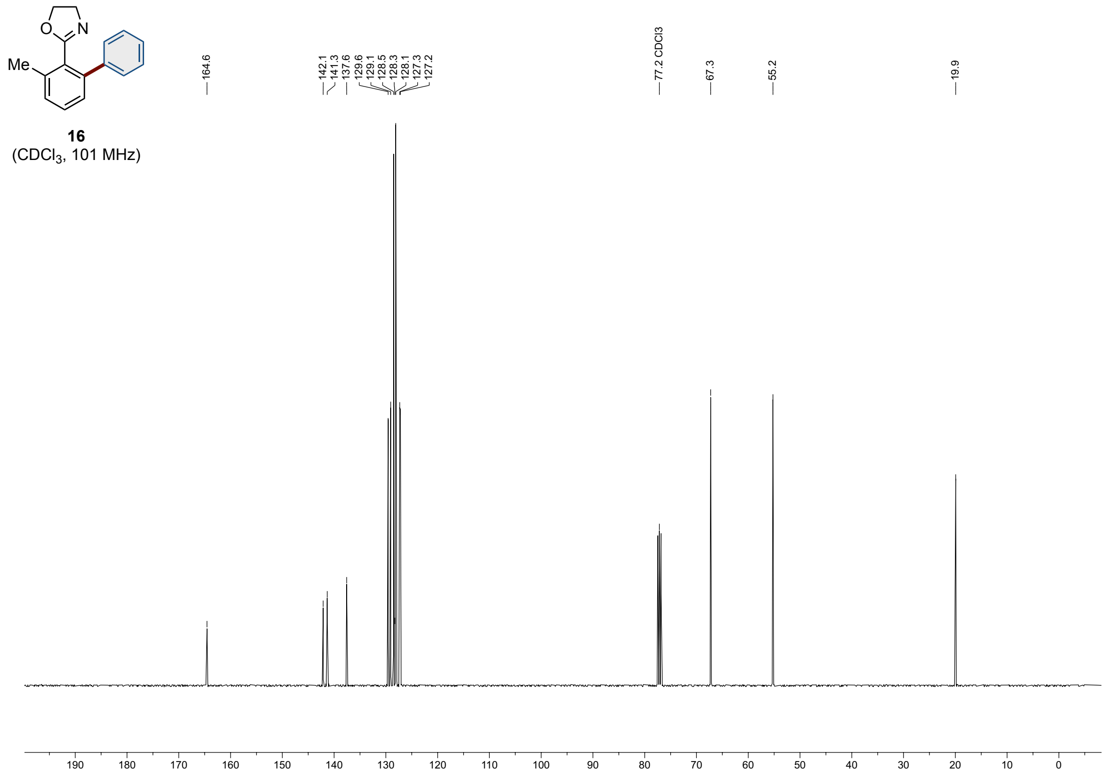

Figure S46. ^{13}C-NMR spectrum of **16**.

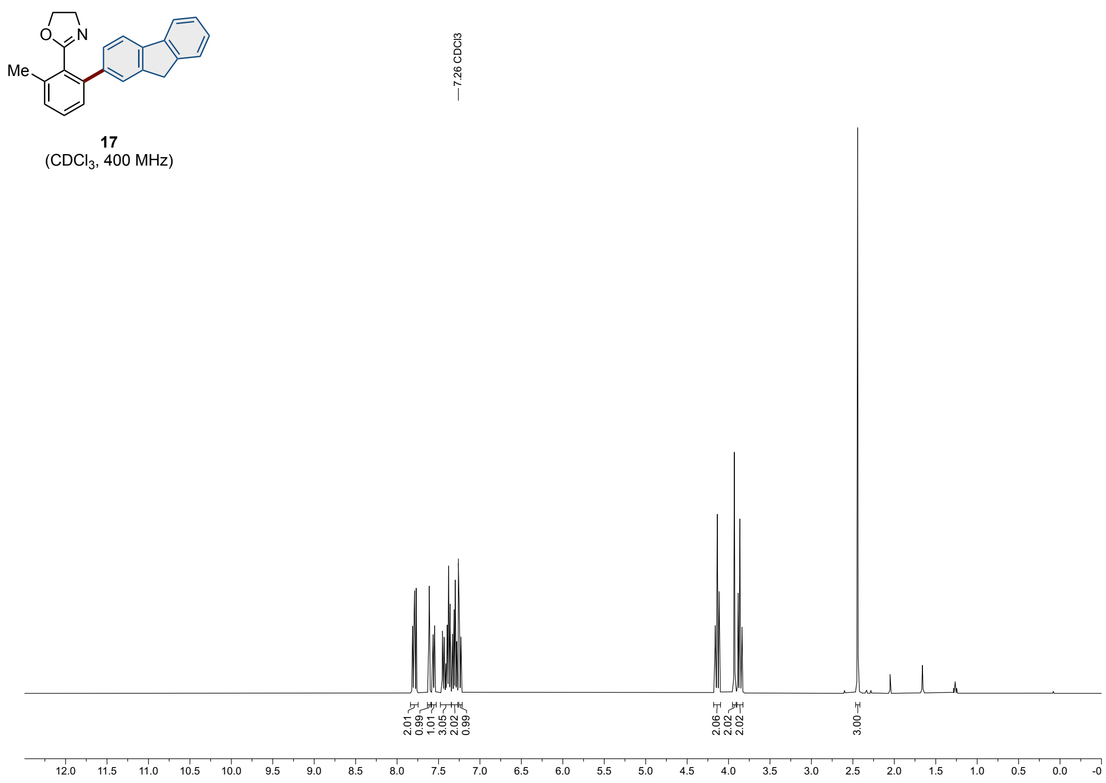

Figure S47. ^{1}H-NMR spectrum of **17**.

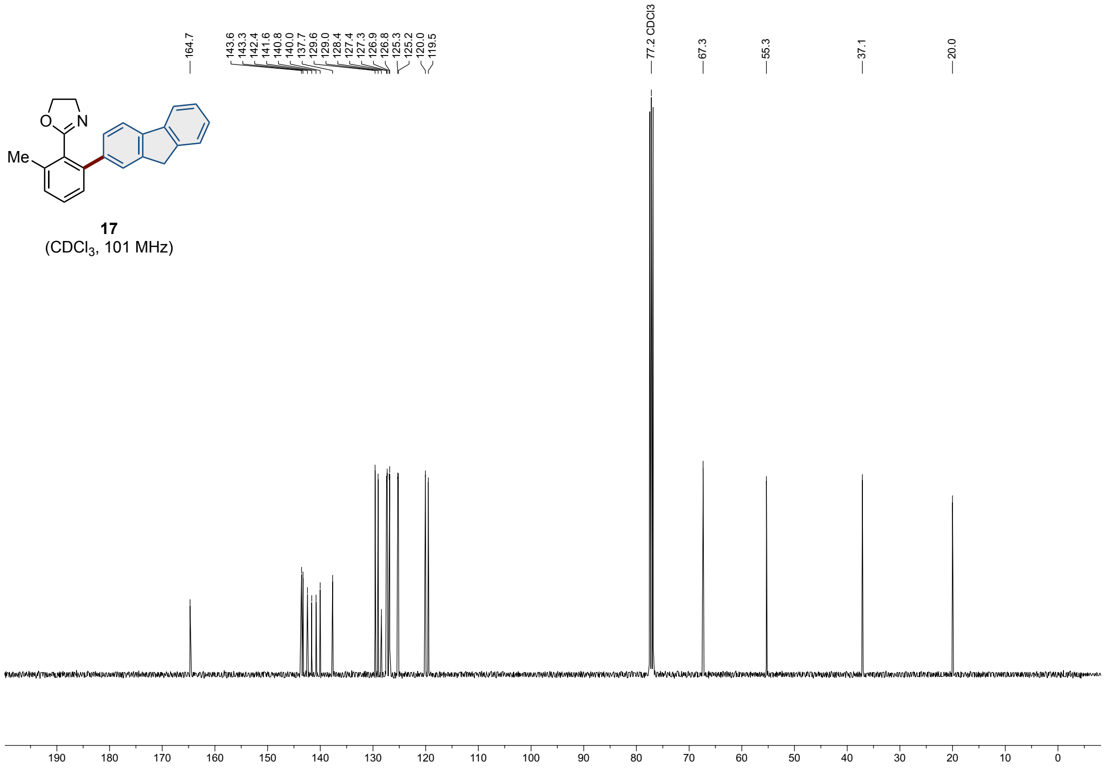

Figure S48. ^{13}C-NMR spectrum of **17**.

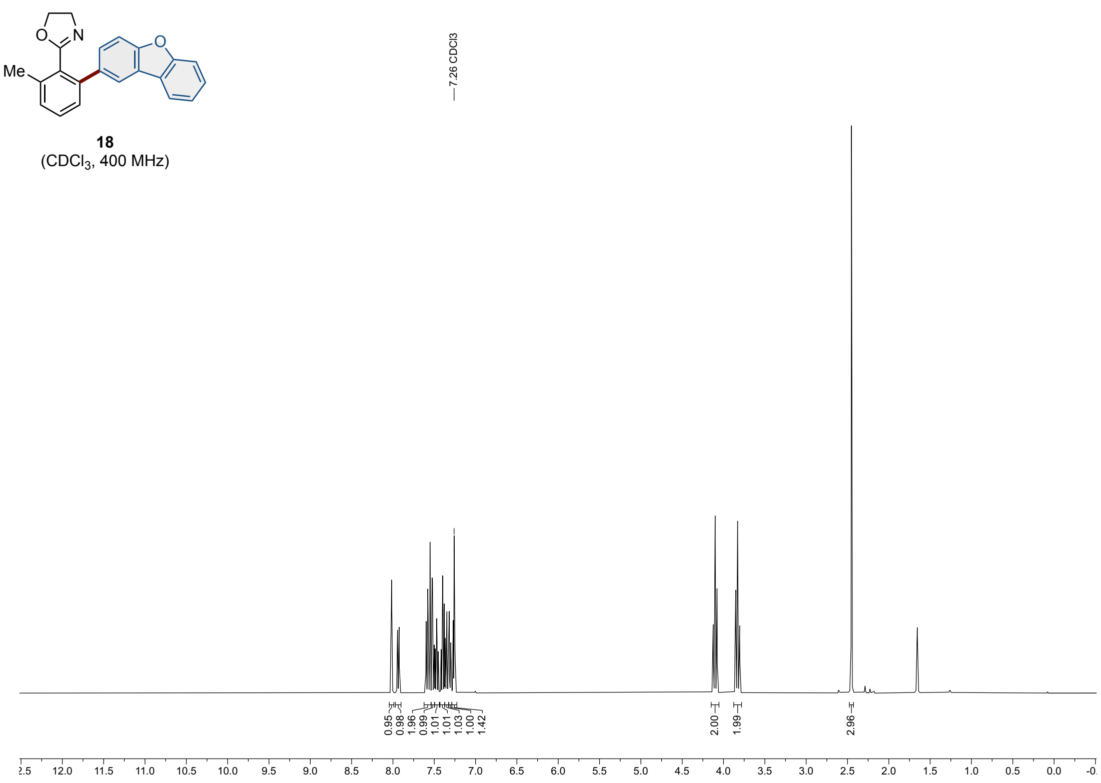

Figure S49. ^{1}H-NMR spectrum of **18**.

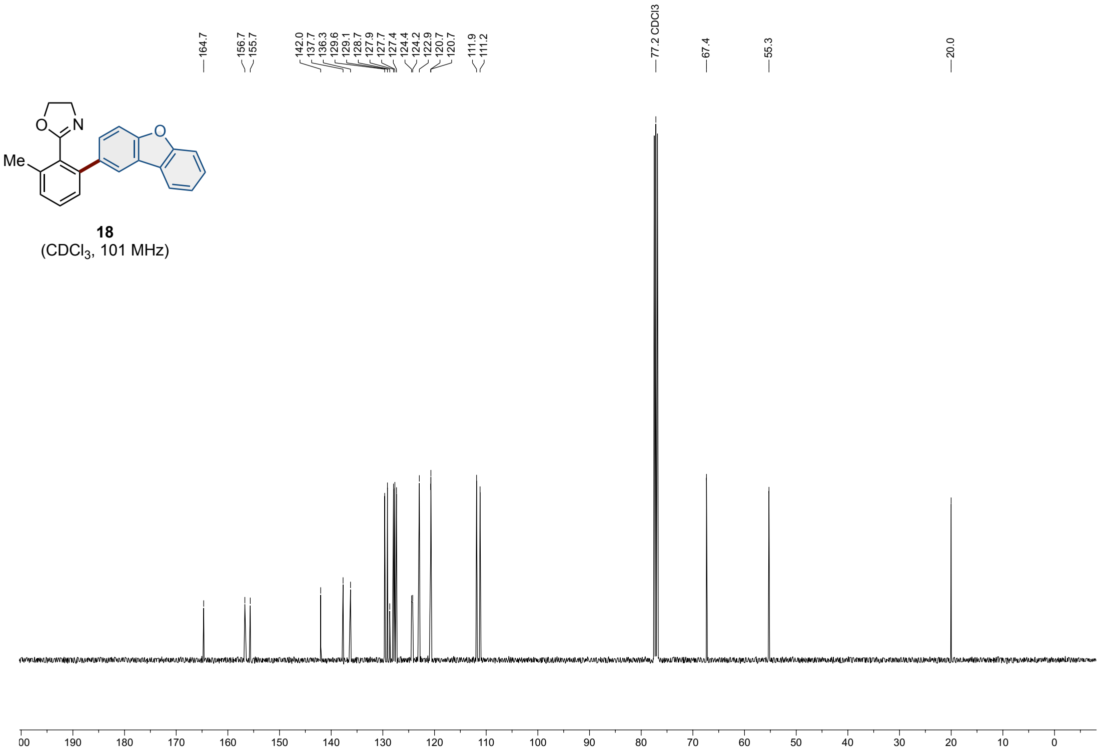

Figure S50. ^{13}C-NMR spectrum of **18**.

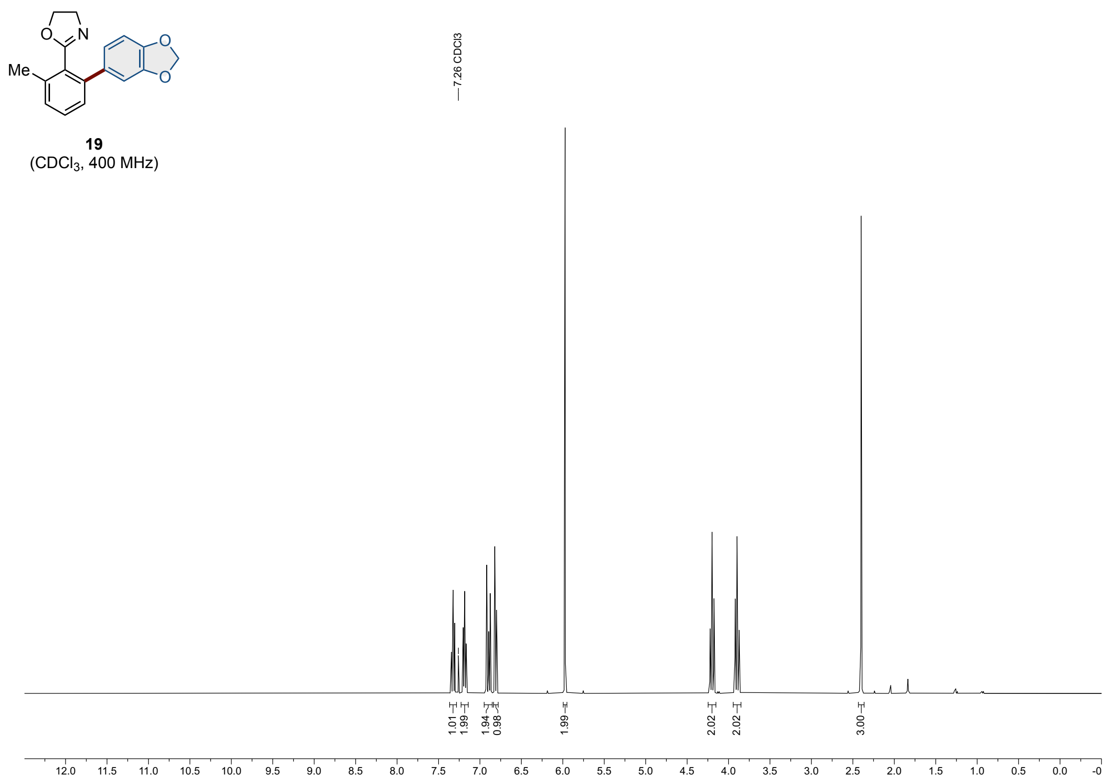

Figure S51. ^{1}H-NMR spectrum of **19**.

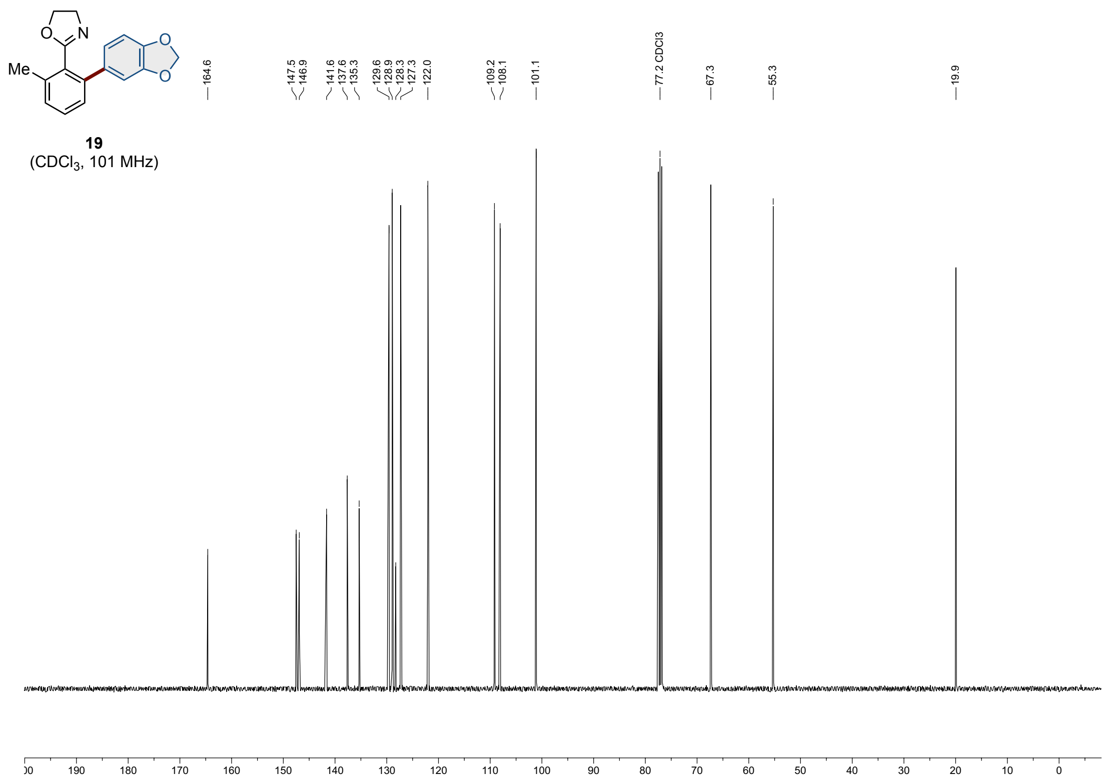

Figure S52. ^{13}C-NMR spectrum of **19**.

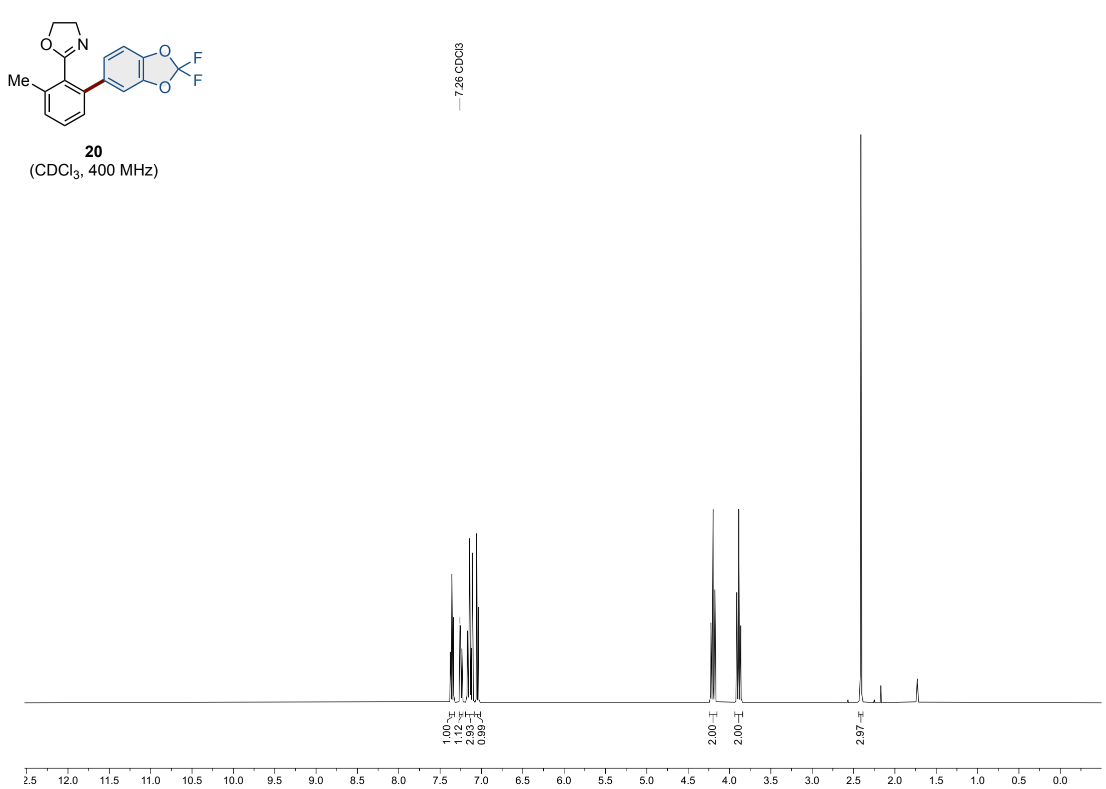

20
(CDCl$_3$, 400 MHz)

Figure S53. ^{1}H-NMR spectrum of **20**.

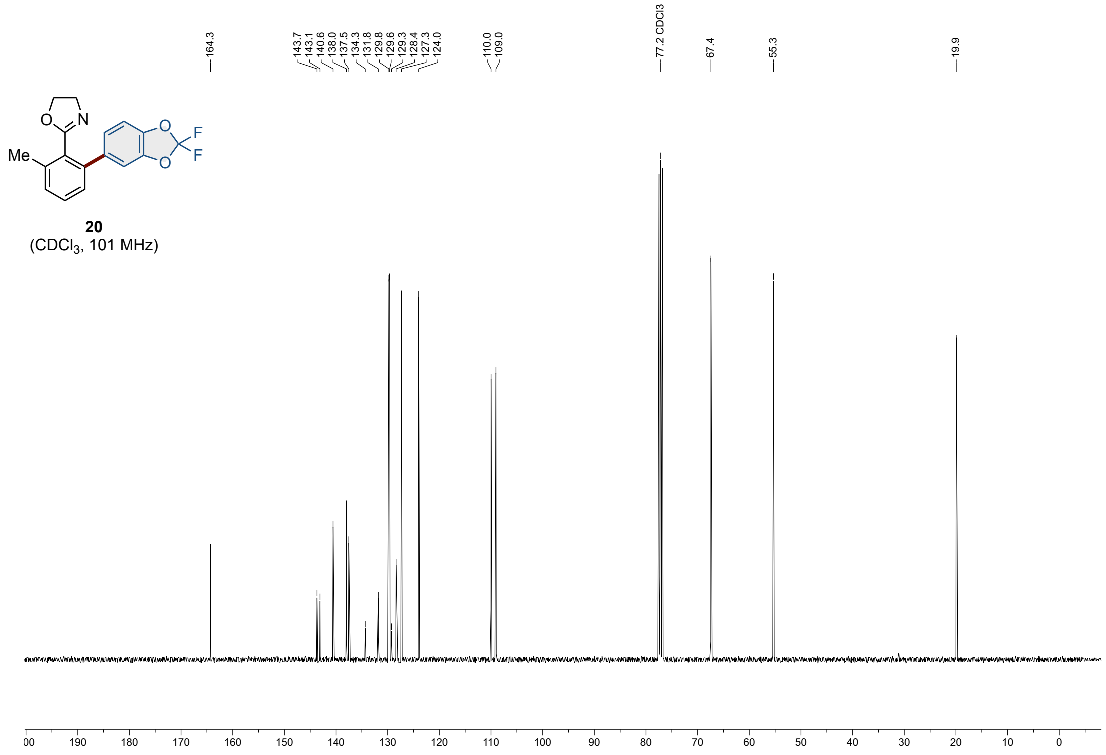

20
(CDCl$_3$, 101 MHz)

Figure S54. ^{13}C-NMR spectrum of **20**.

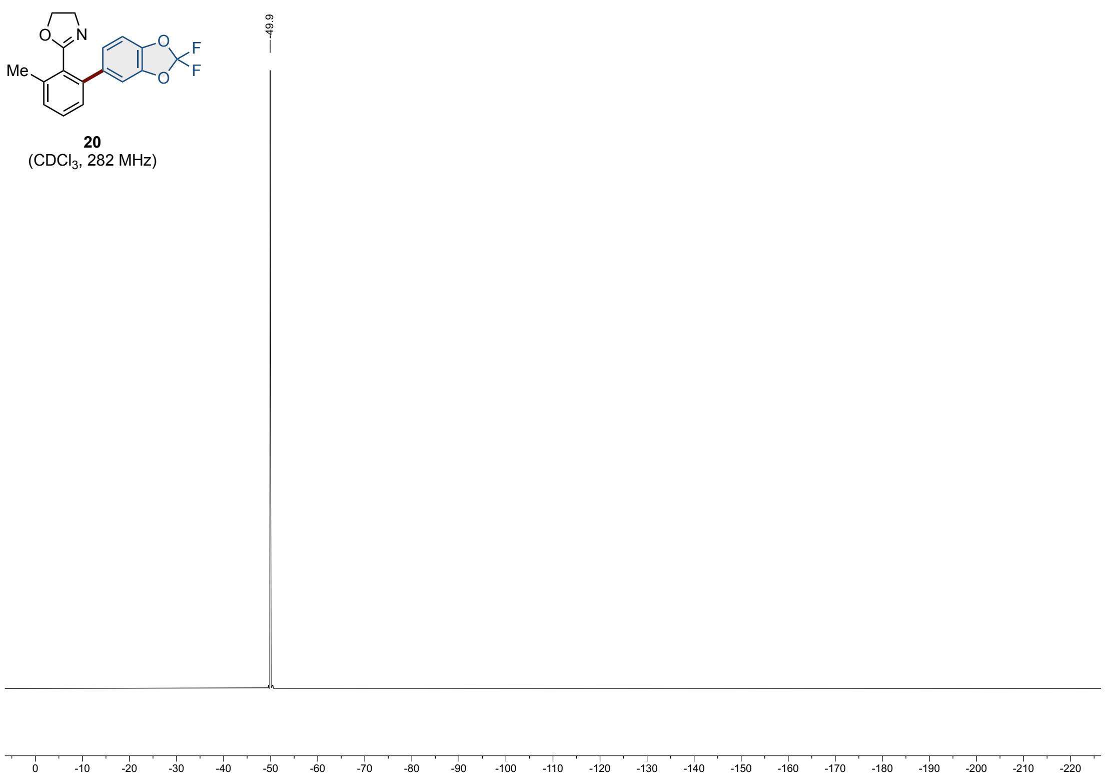

Figure S55. ^{19}F-NMR spectrum of **20**.

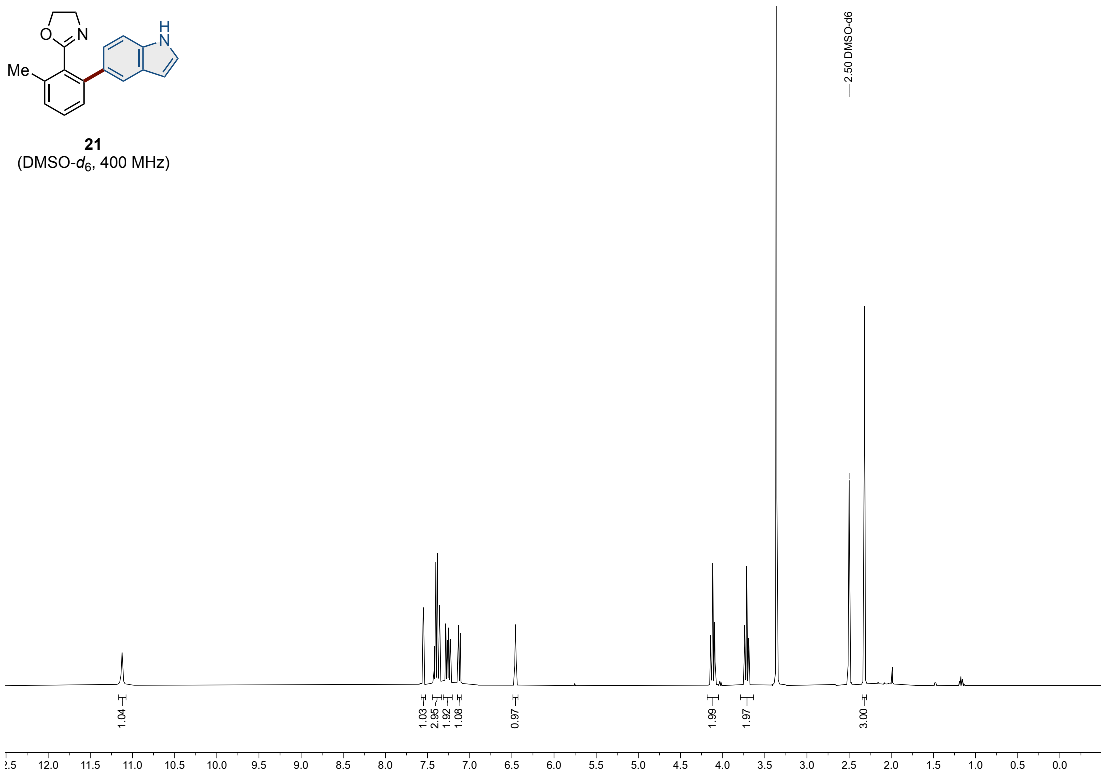

Figure S56. ^{1}H-NMR spectrum of **21**.

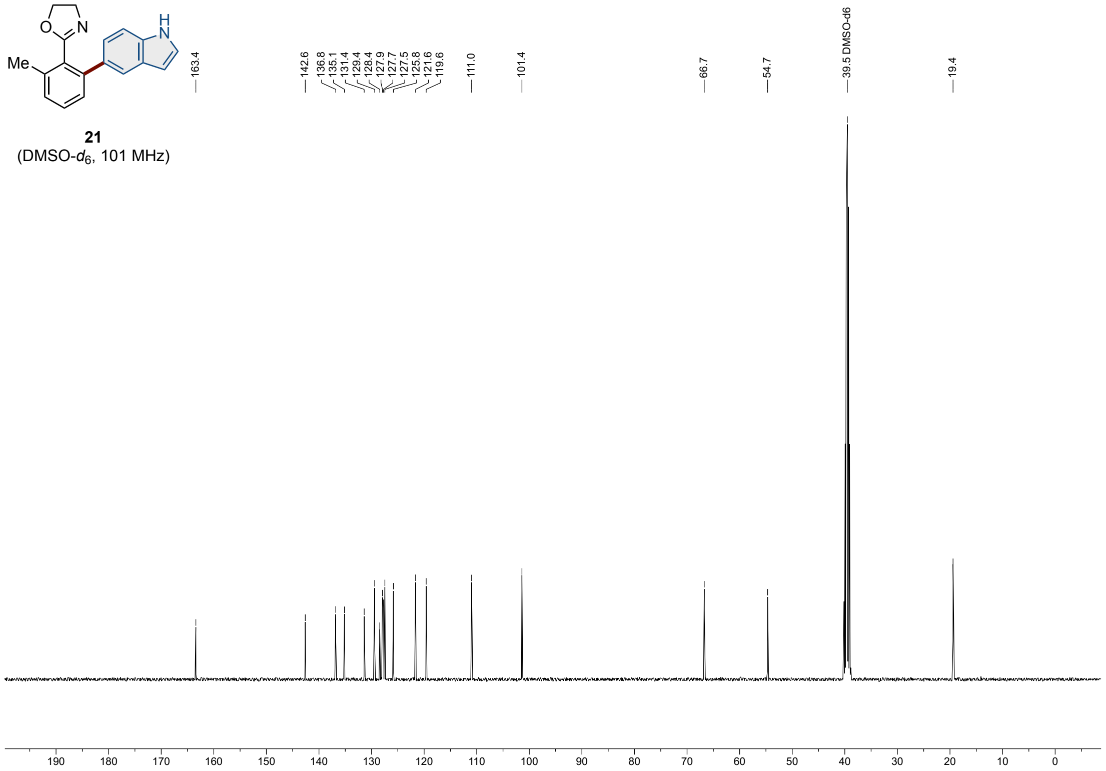

Figure S57. ^{13}C-NMR spectrum of **21**.

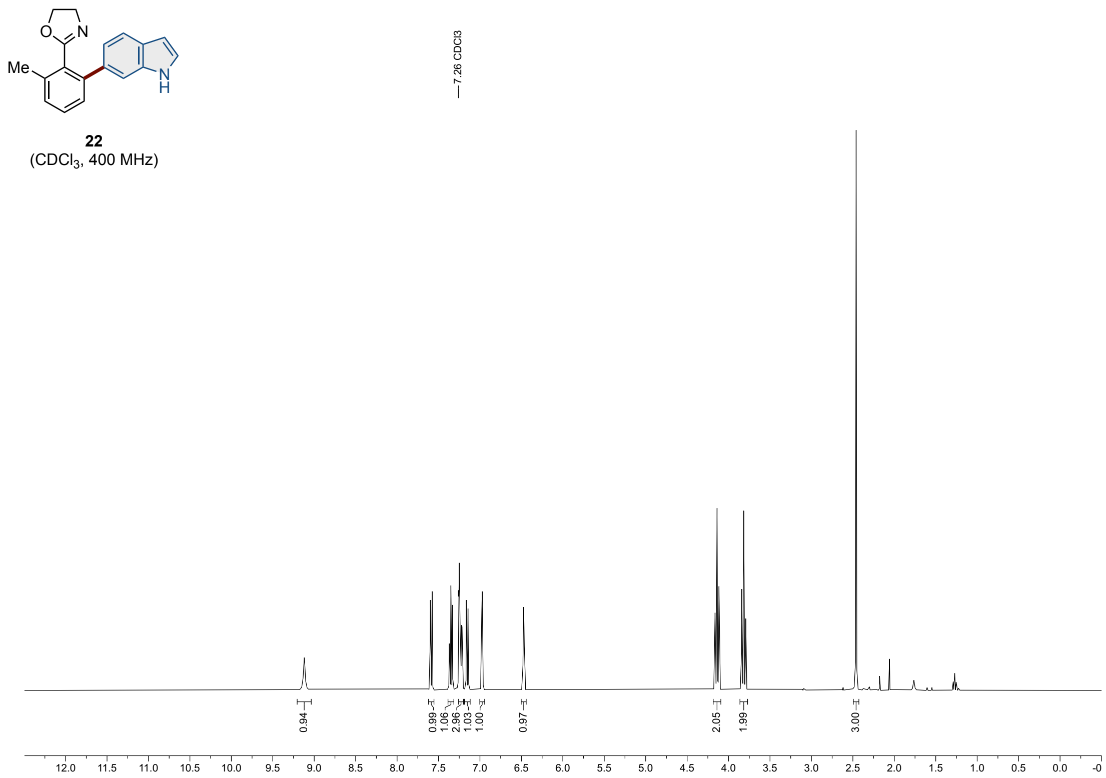

Figure S58. ^{1}H-NMR spectrum of **22**.

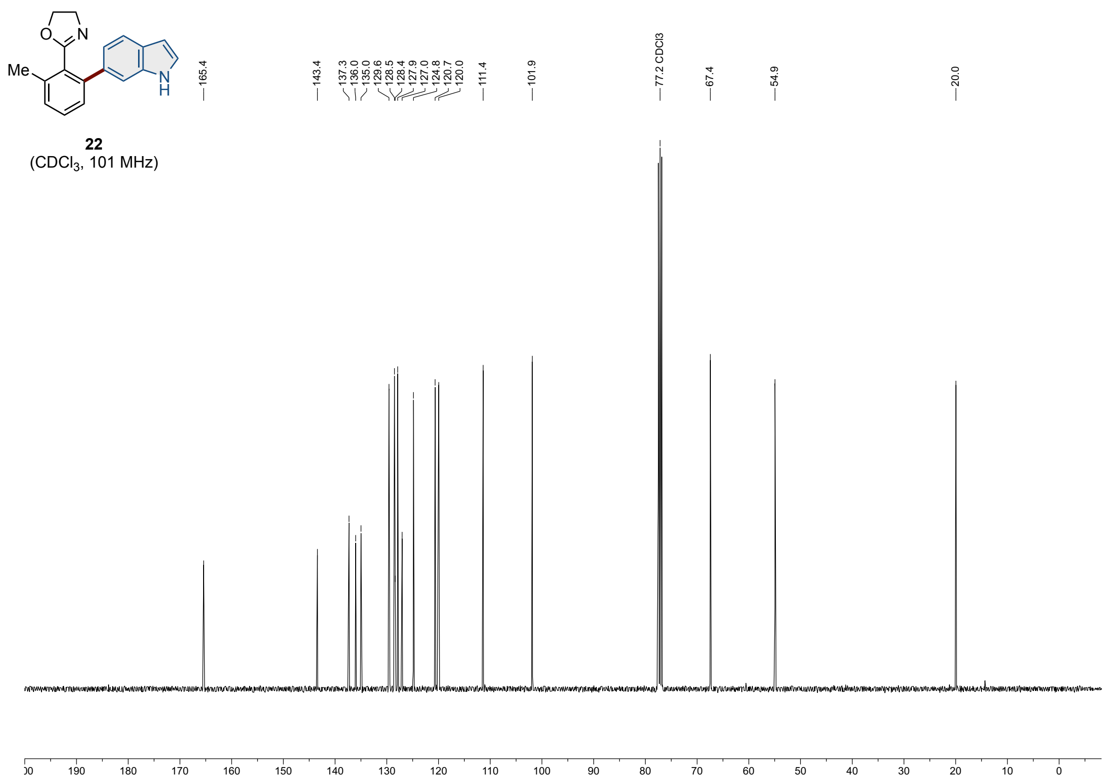

Figure S59. ^{13}C-NMR spectrum of **22**.

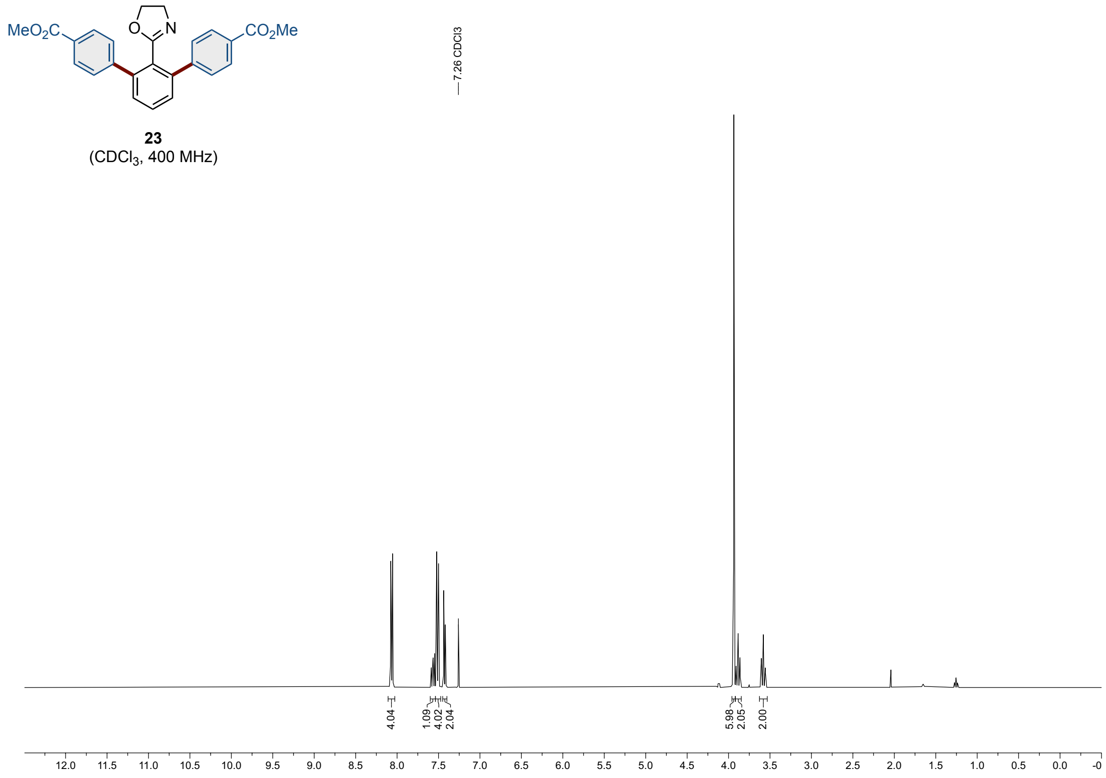

Figure S60. ^{1}H-NMR spectrum of **23**.

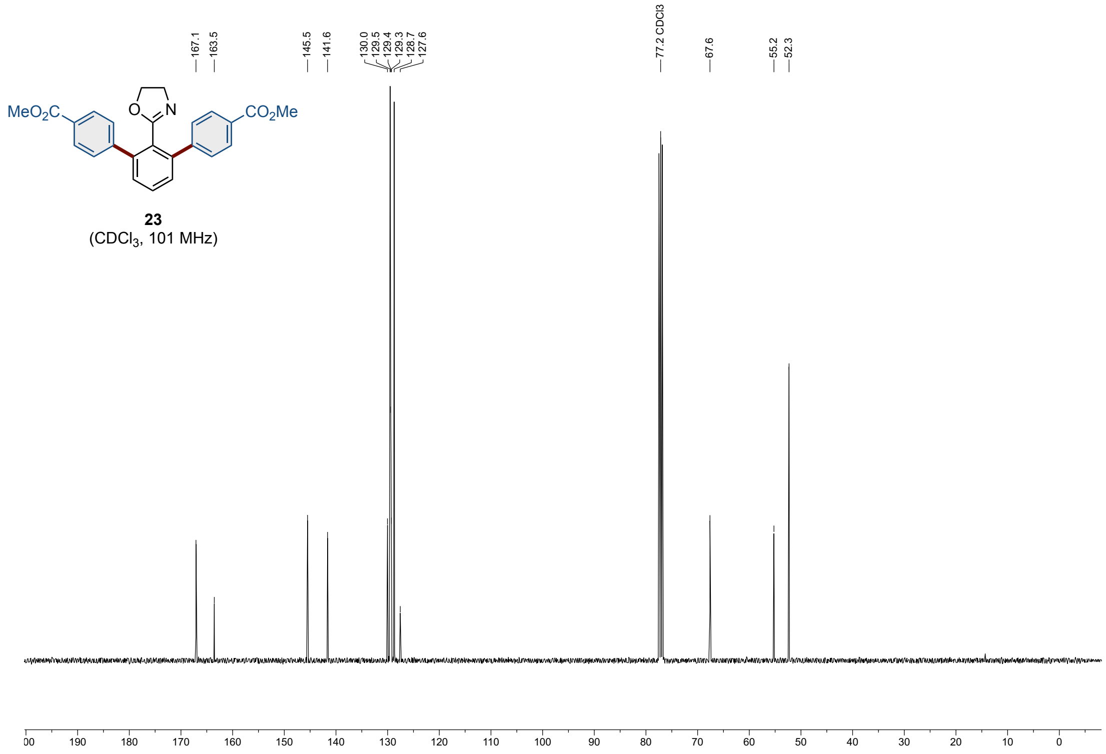

Figure S61. ^{13}C-NMR spectrum of **23**.

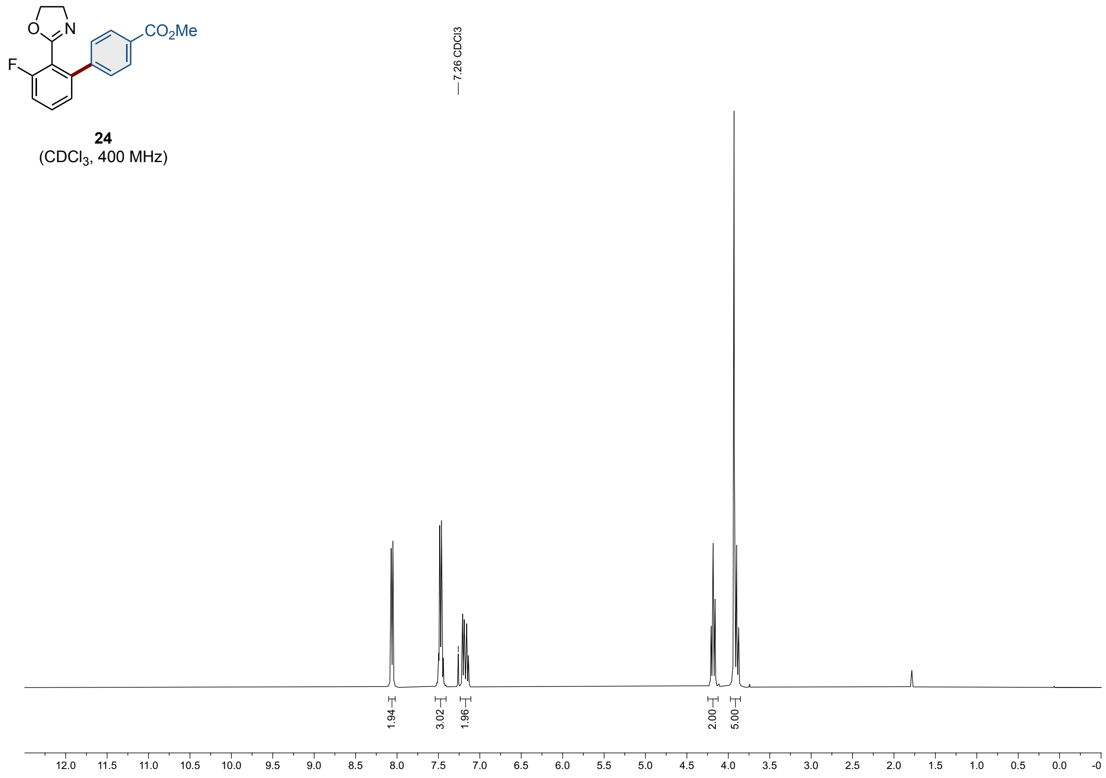

Figure S62. ^{1}H-NMR spectrum of **24**.

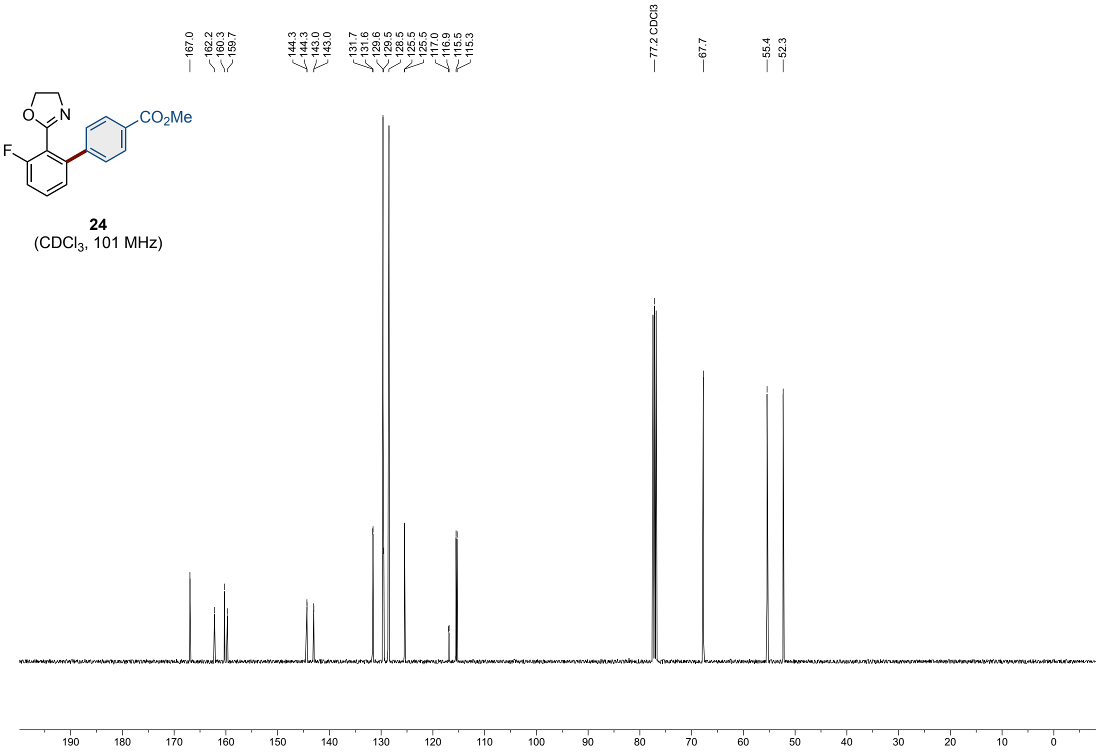

Figure S63. ^{13}C-NMR spectrum of **24**.

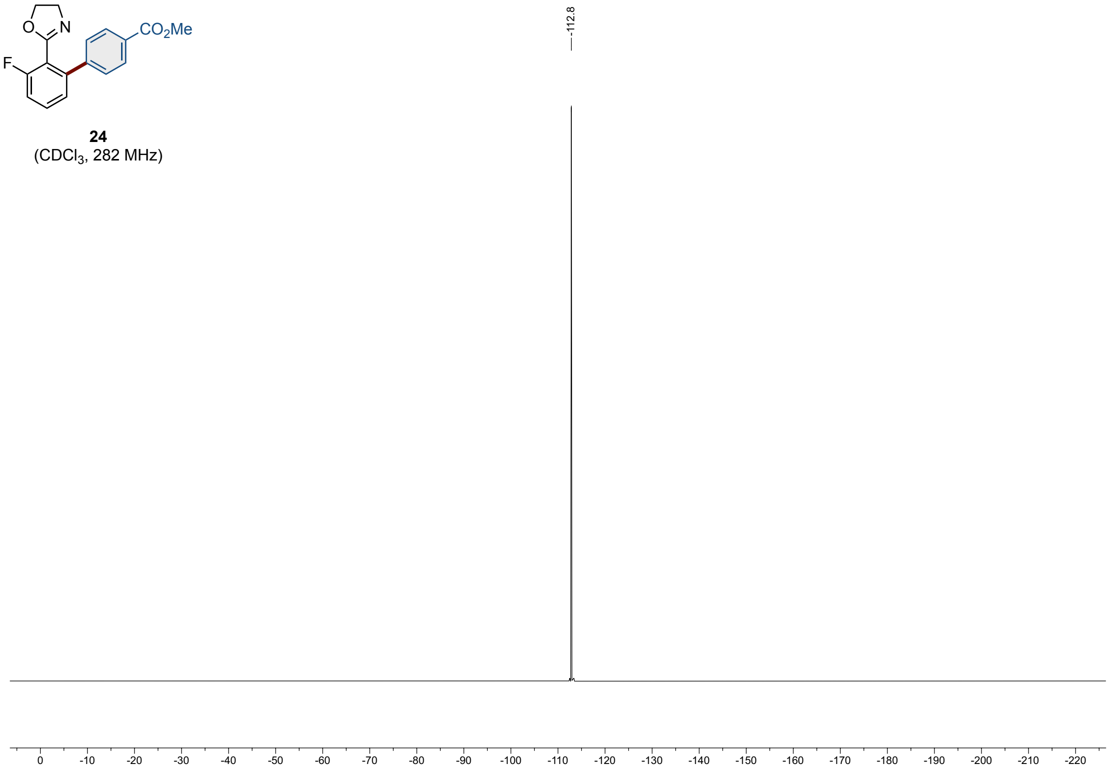

Figure S64. ^{19}F-NMR spectrum of **24**.

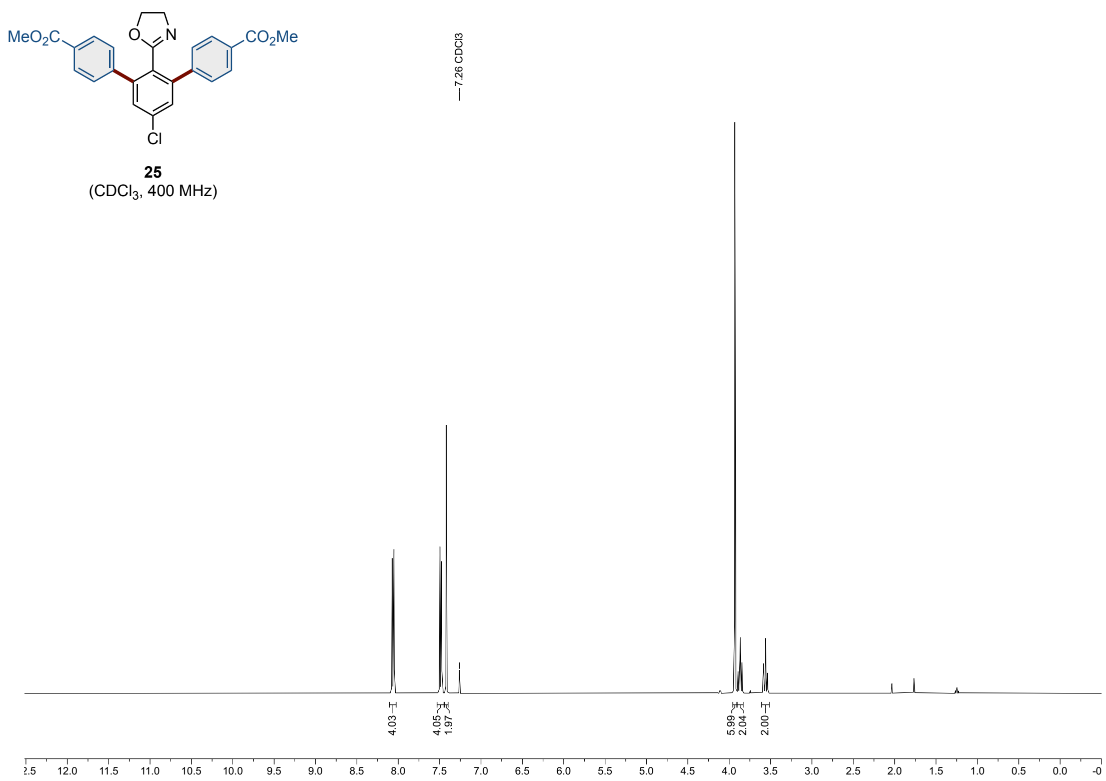

Figure S65. ^{1}H-NMR spectrum of **25**.

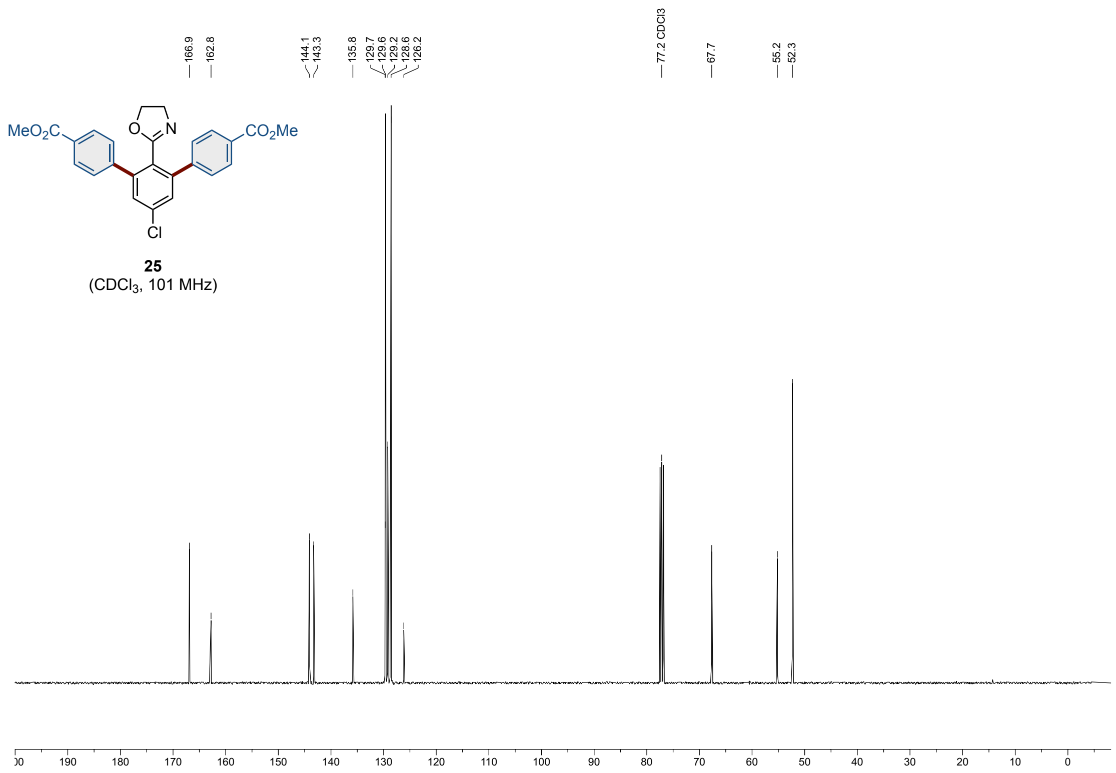

Figure S66. ^{13}C-NMR spectrum of **25**.

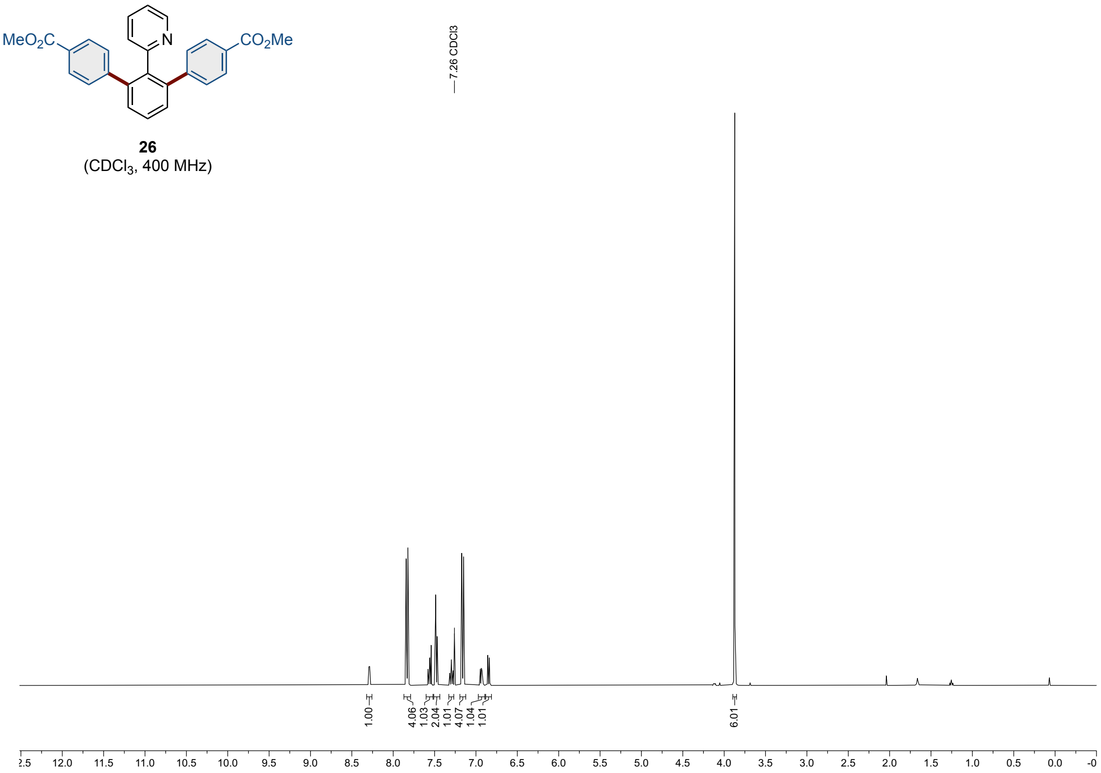

Figure S67. ^{1}H-NMR spectrum of **26**.

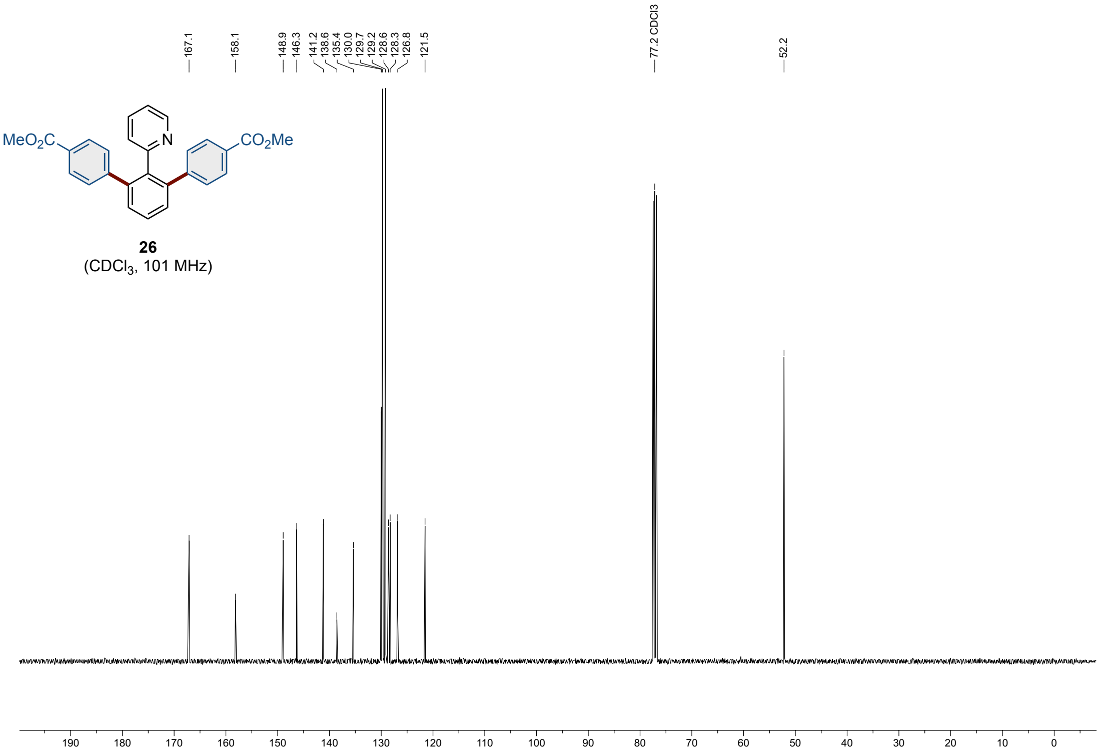

Figure S68. ^{13}C-NMR spectrum of **26**.

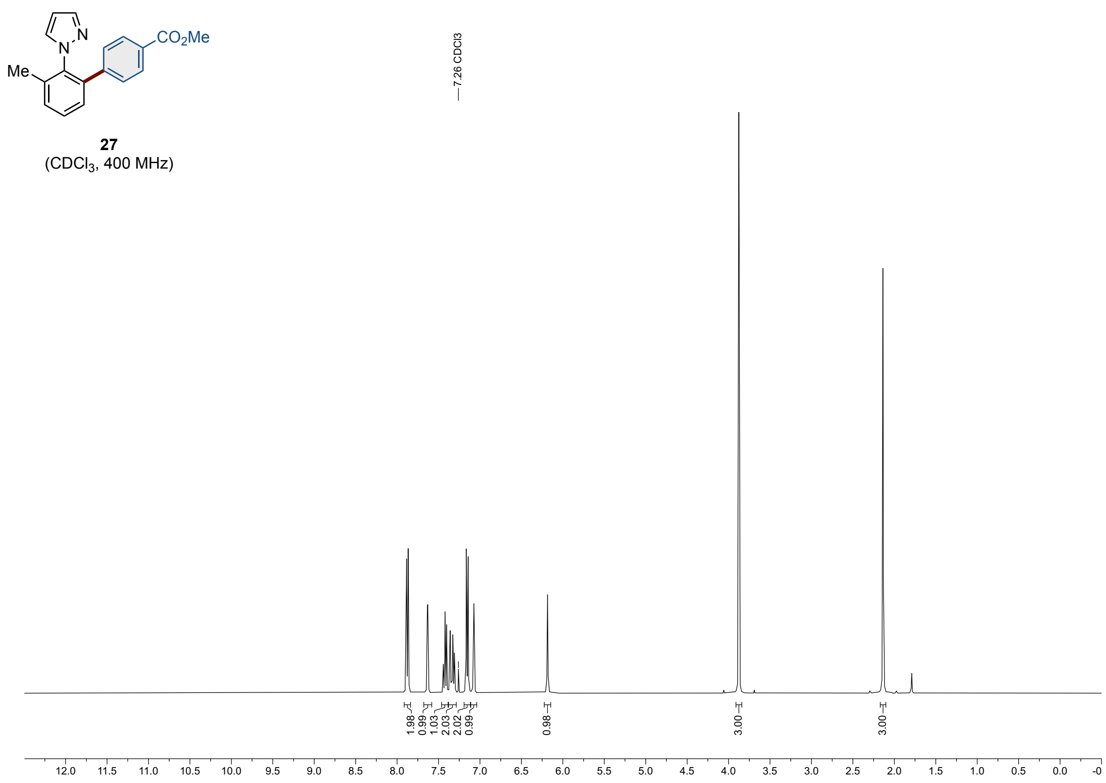

Figure S69. ^{1}H-NMR spectrum of **27**.

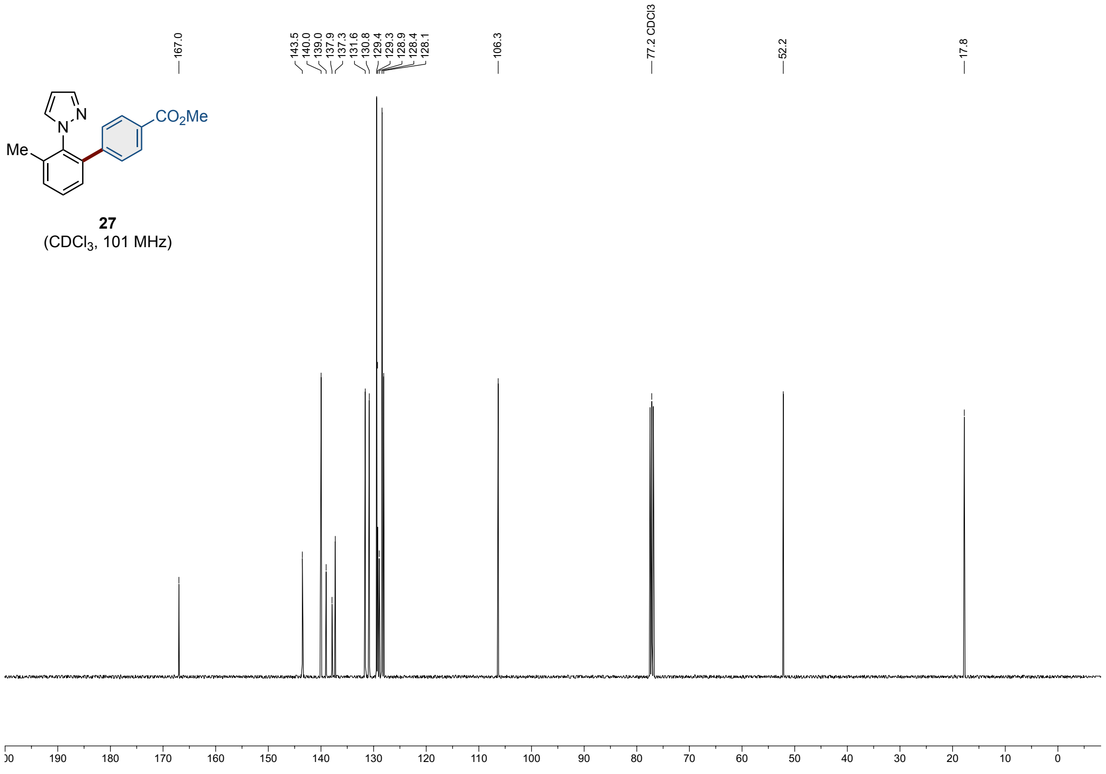

Figure S70. ^{13}C-NMR spectrum of **27**.

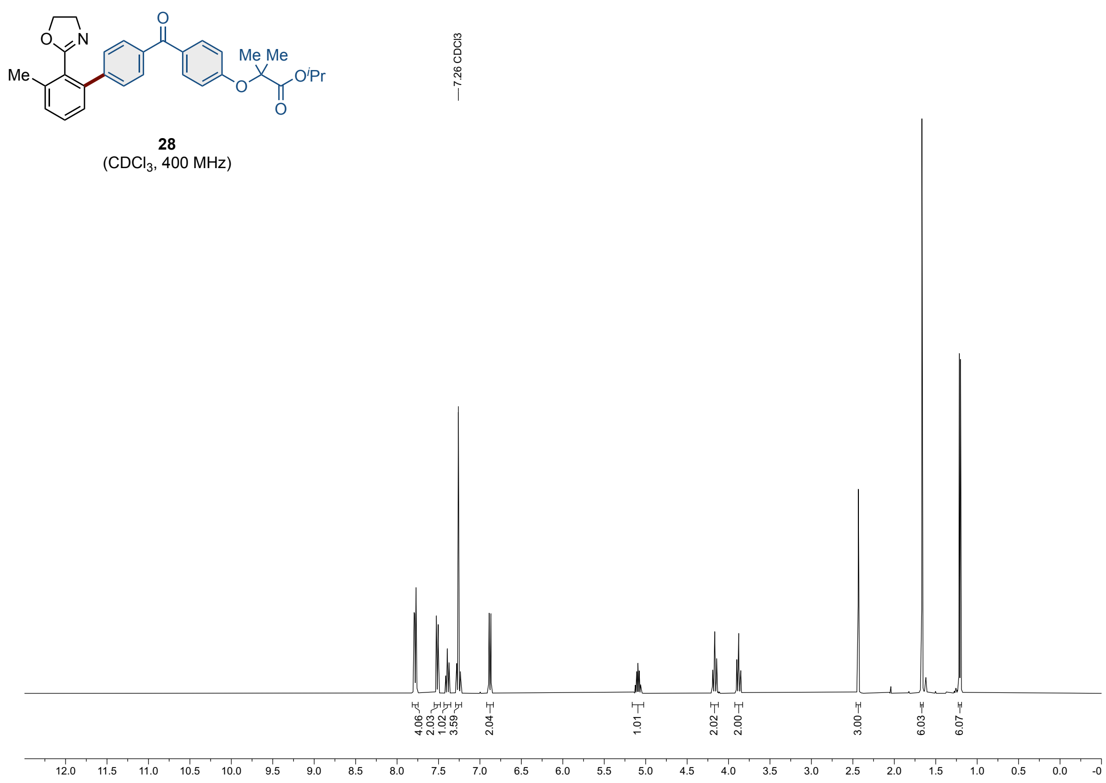

Figure S71. ^{1}H-NMR spectrum of **28**.

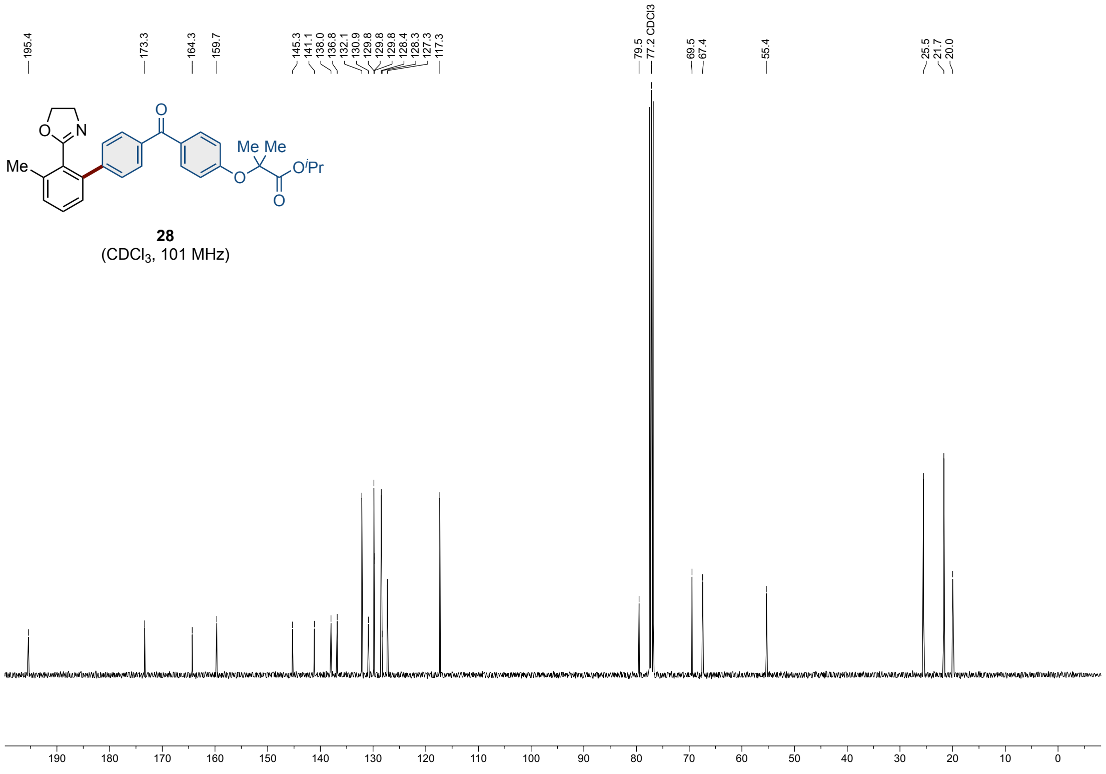

Figure S72. ^{13}C-NMR spectrum of **28**.

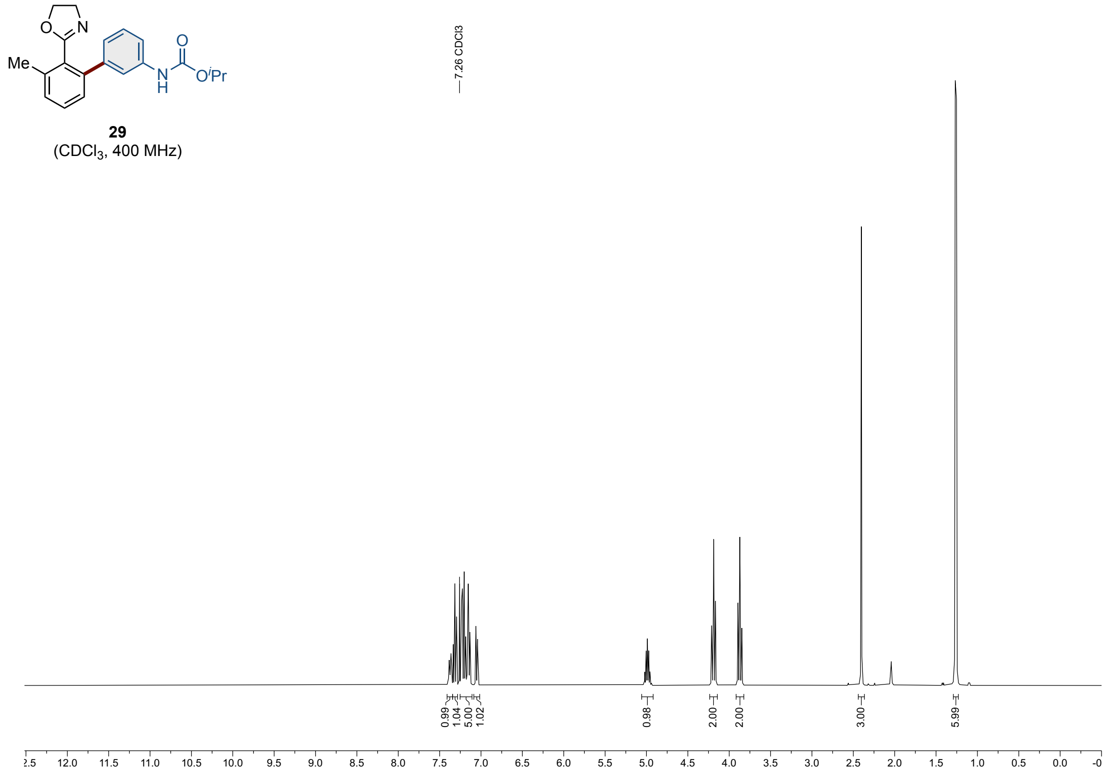

Figure S73. ^{1}H-NMR spectrum of **29**.

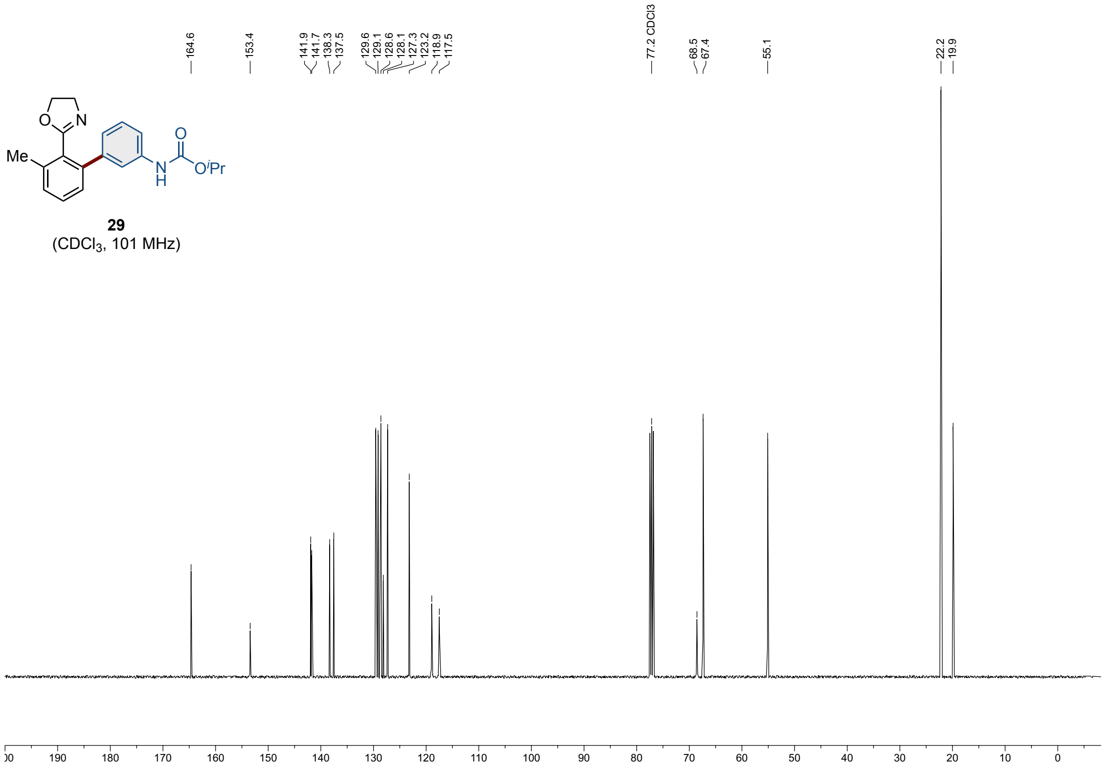

Figure S74. ^{13}C-NMR spectrum of **29**.

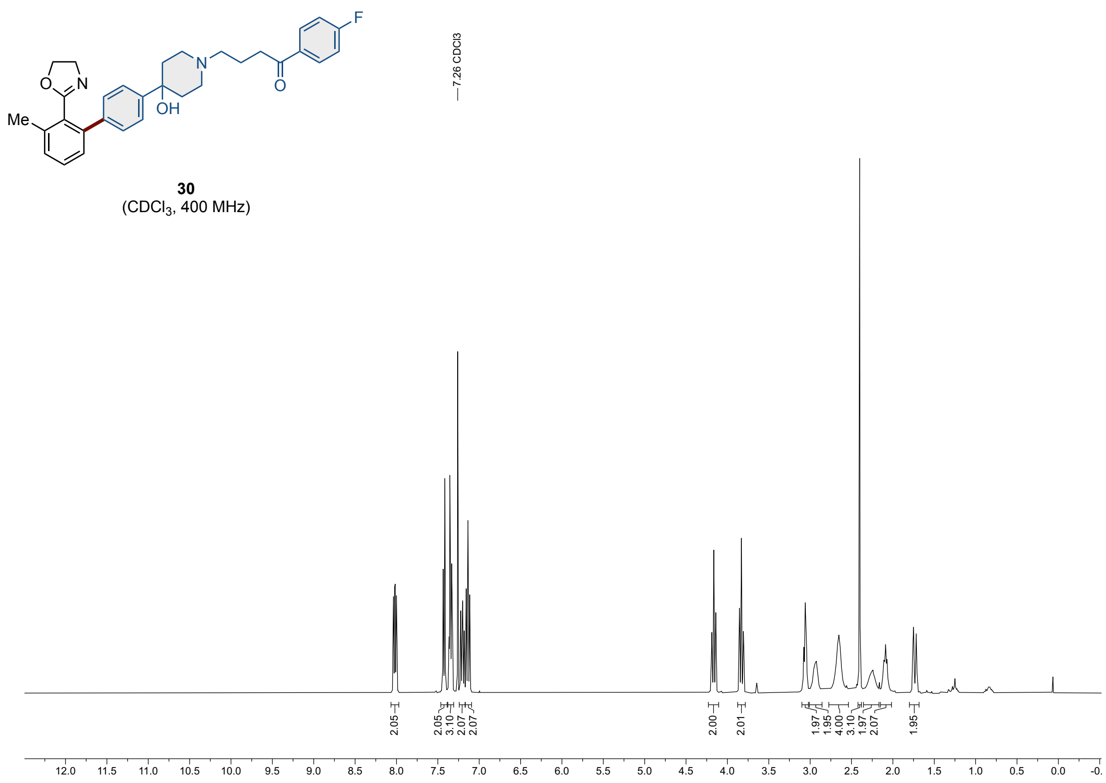

Figure S75. ^{1}H-NMR spectrum of **30**.

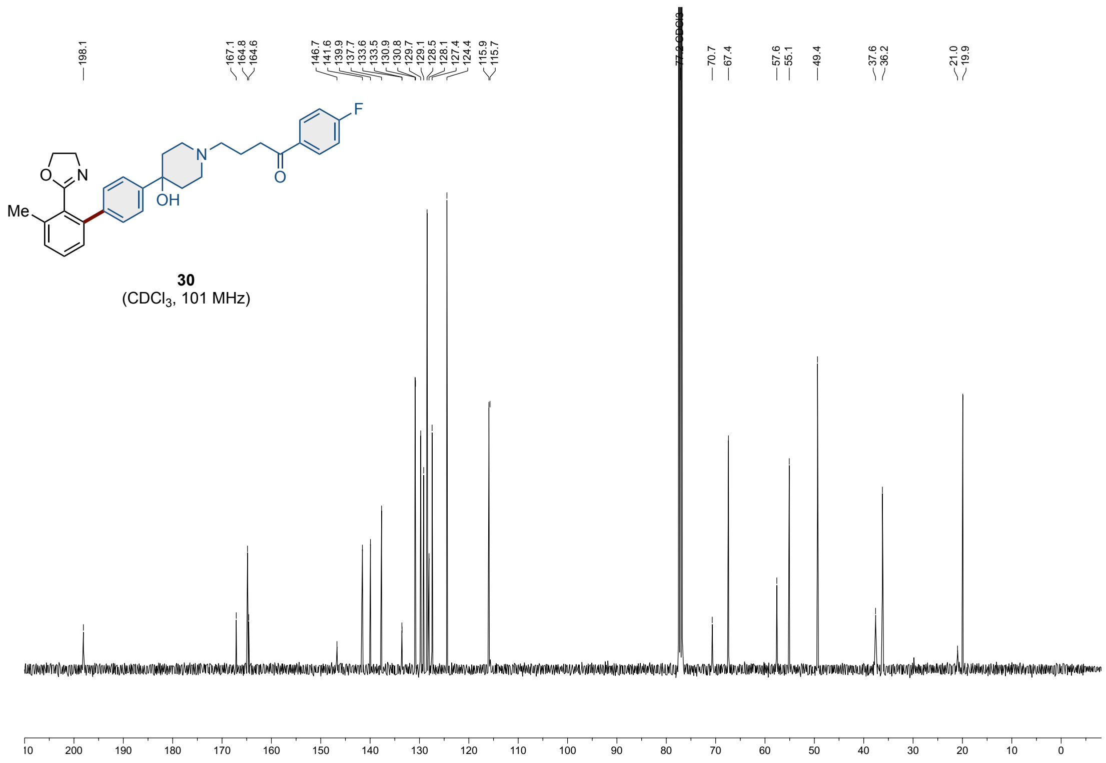

Figure S76. ^{13}C-NMR spectrum of **30**.

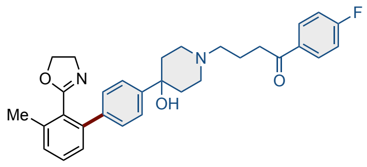

30
($CDCl_3$, 282 MHz)

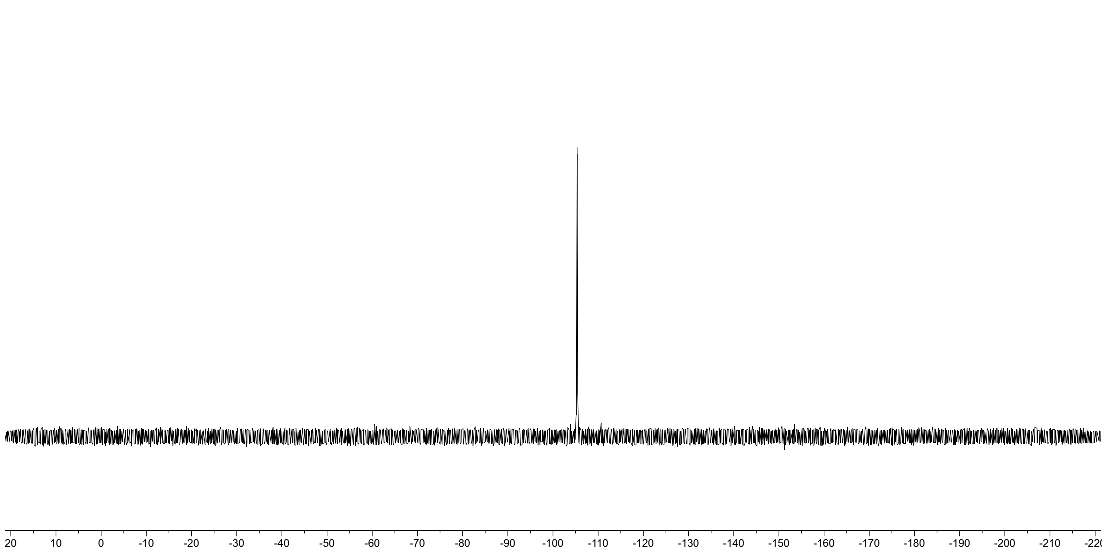

Figure S77. ^{19}F-NMR spectrum of **30**.

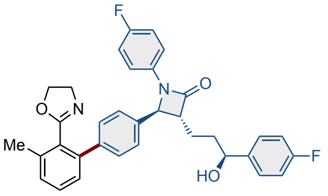

31
($CDCl_3$, 400 MHz)

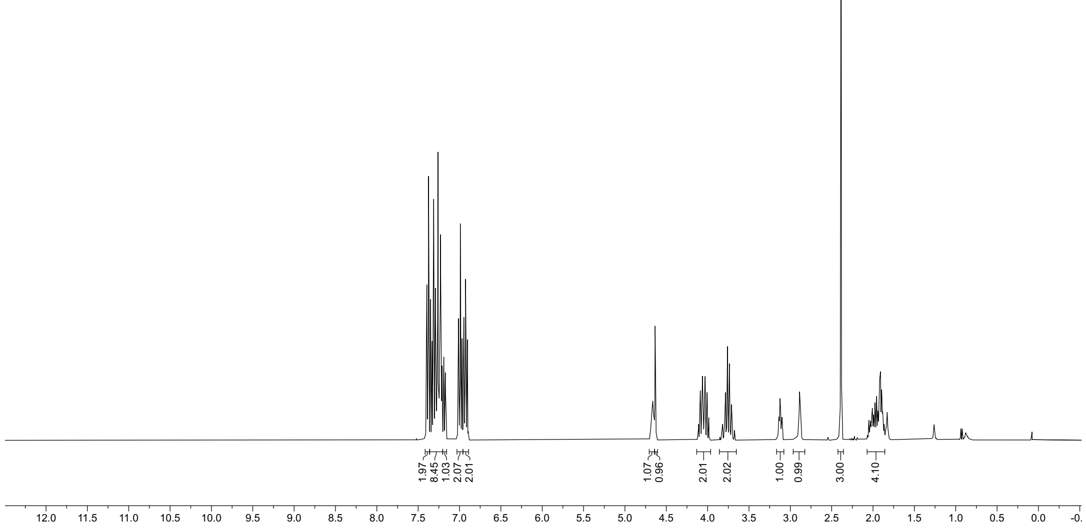

Figure S78. ^{1}H-NMR spectrum of **31**.

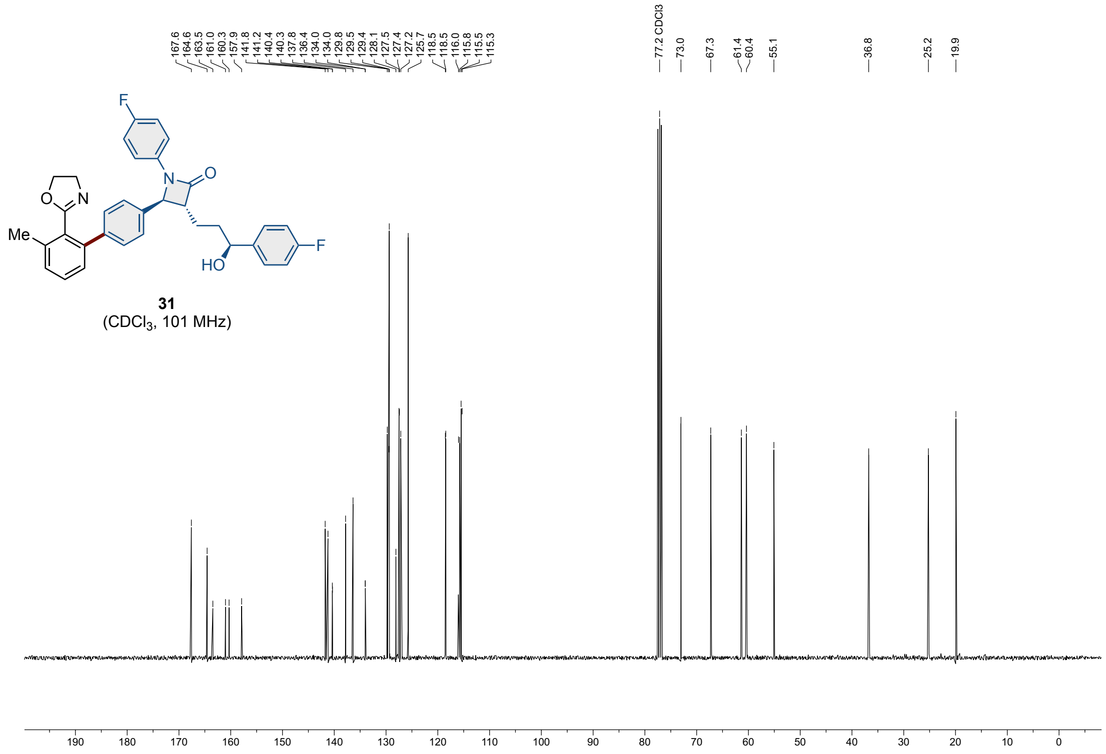

Figure S79. ^{13}C-NMR spectrum of **31**.

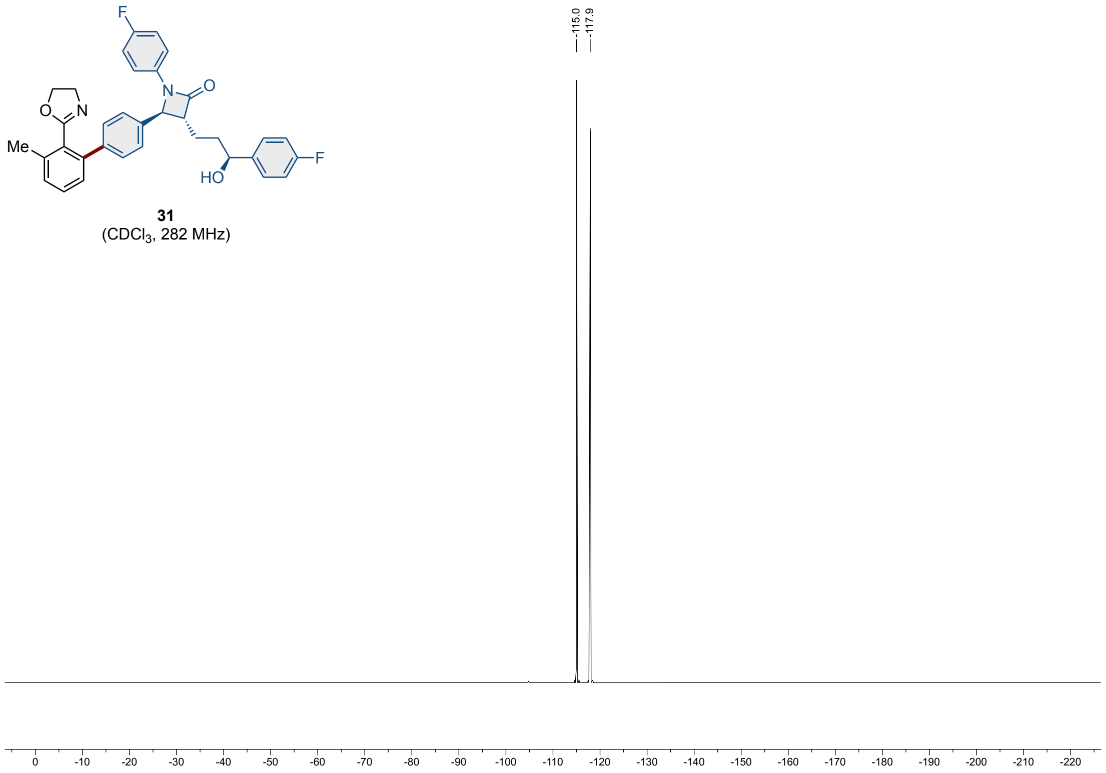

Figure S80. ^{19}F-NMR spectrum of **31**.

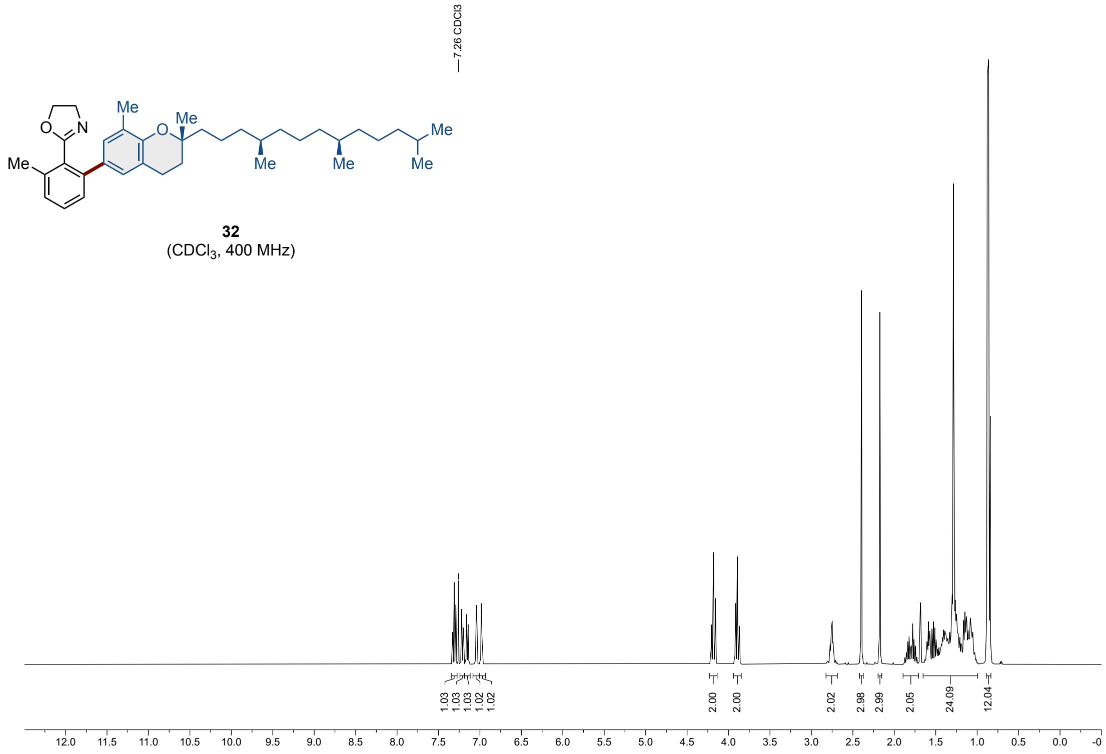

32
($CDCl_3$, 400 MHz)

Figure S81. ^{1}H-NMR spectrum of **32**.

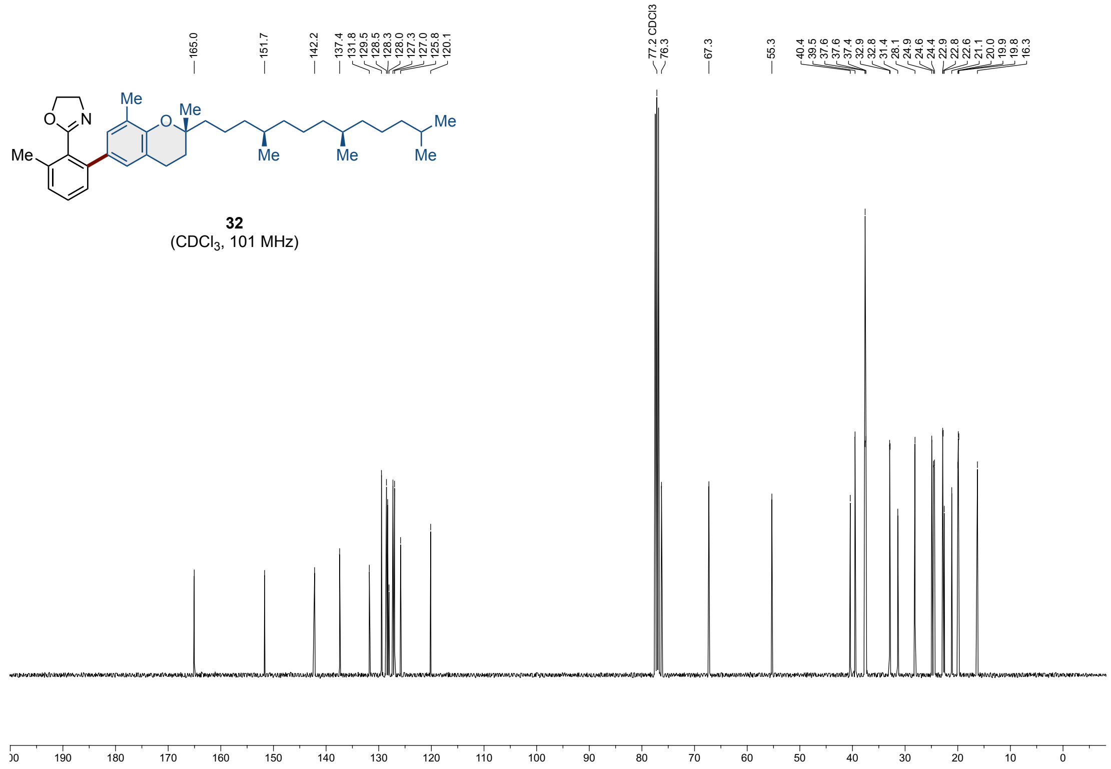

32
($CDCl_3$, 101 MHz)

Figure S82. ^{13}C-NMR spectrum of **32**.

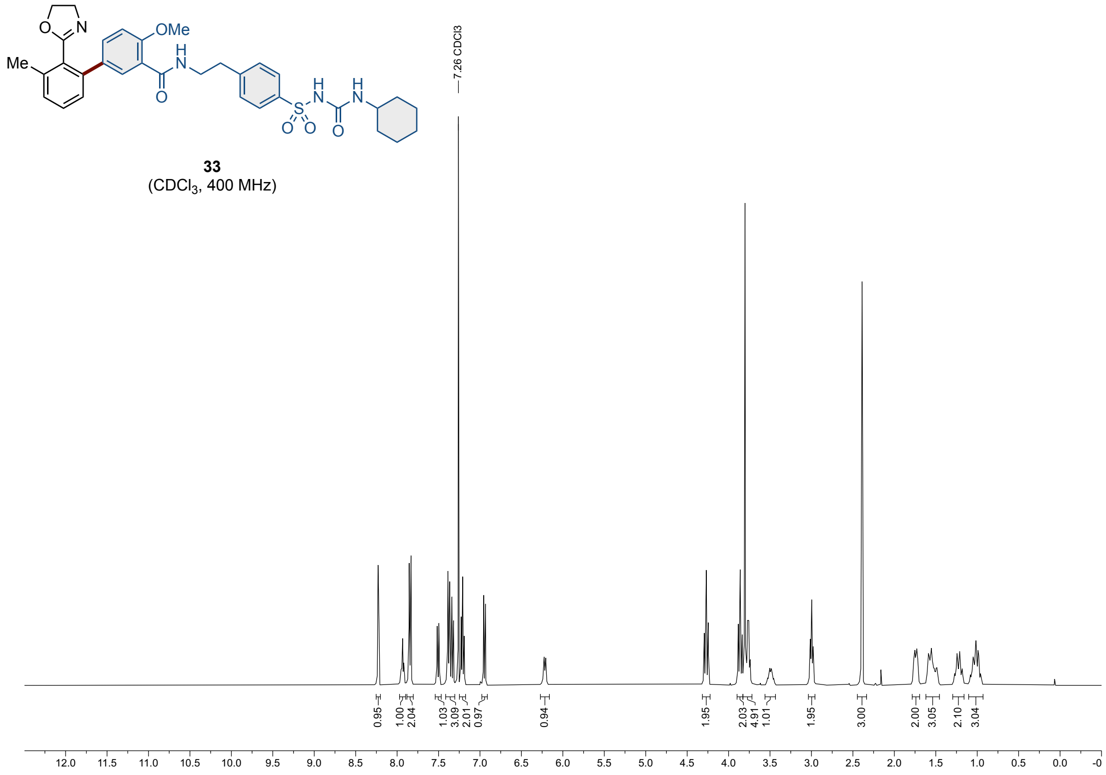

Figure S83. ^{1}H-NMR spectrum of **33**.

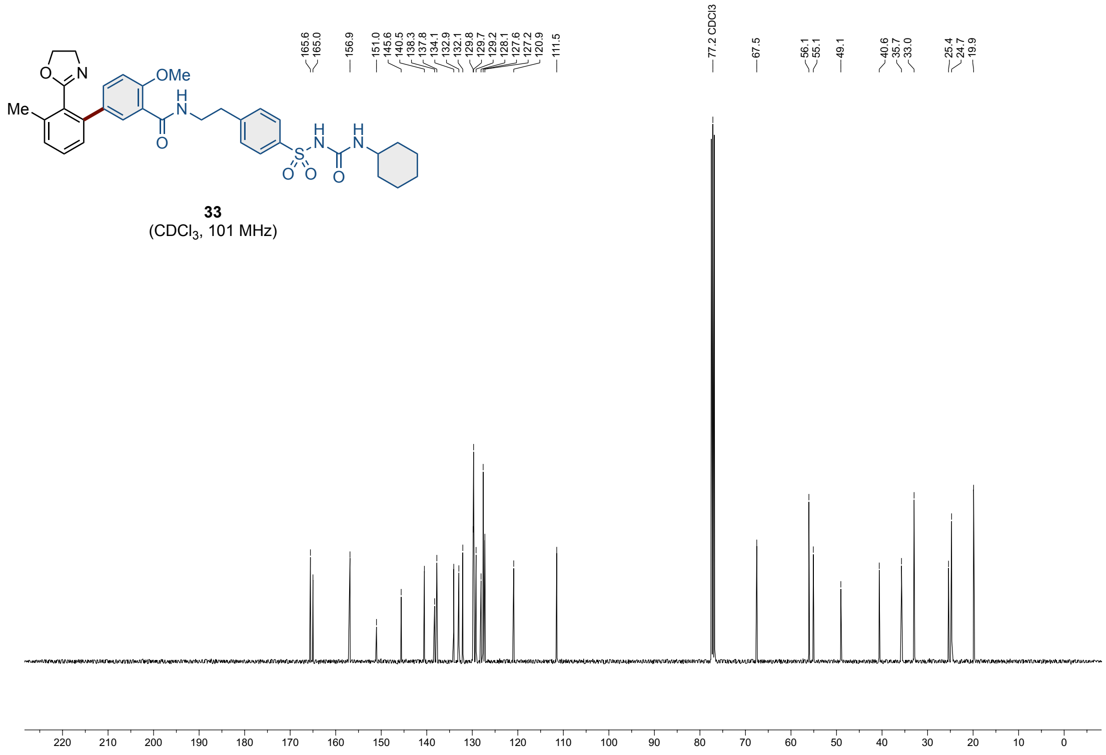

Figure S84. ^{13}C-NMR spectrum of **33**.

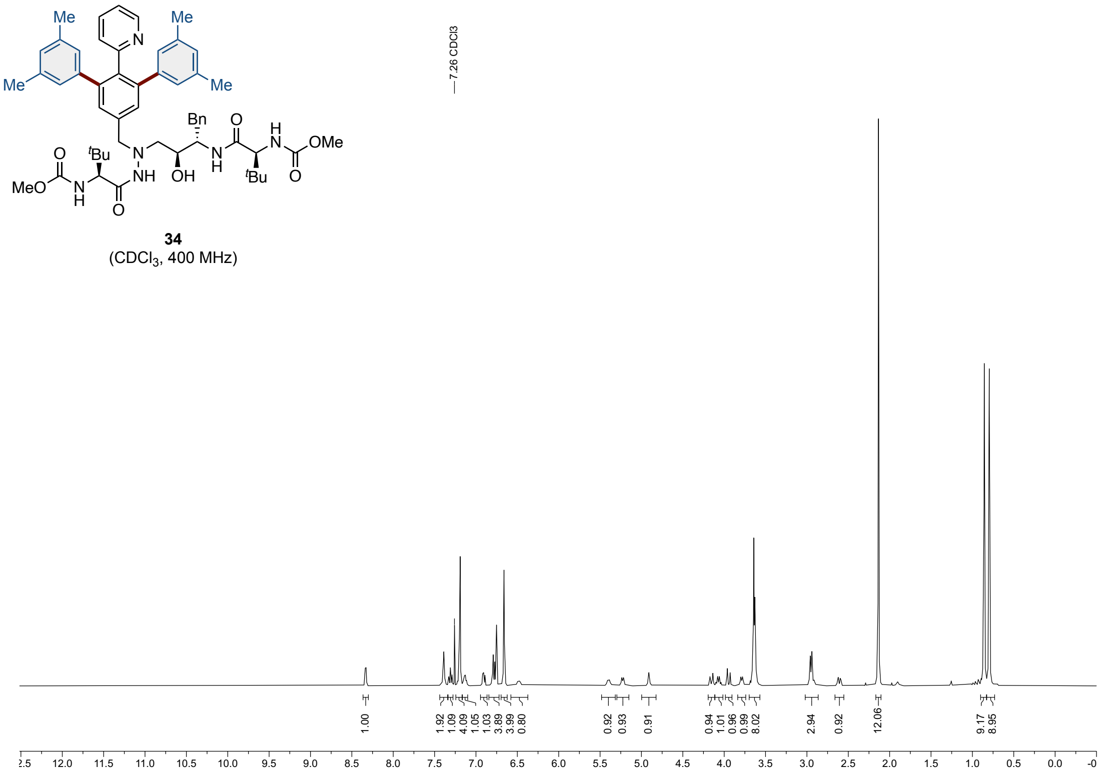

Figure S85. ^{1}H-NMR spectrum of **34**.

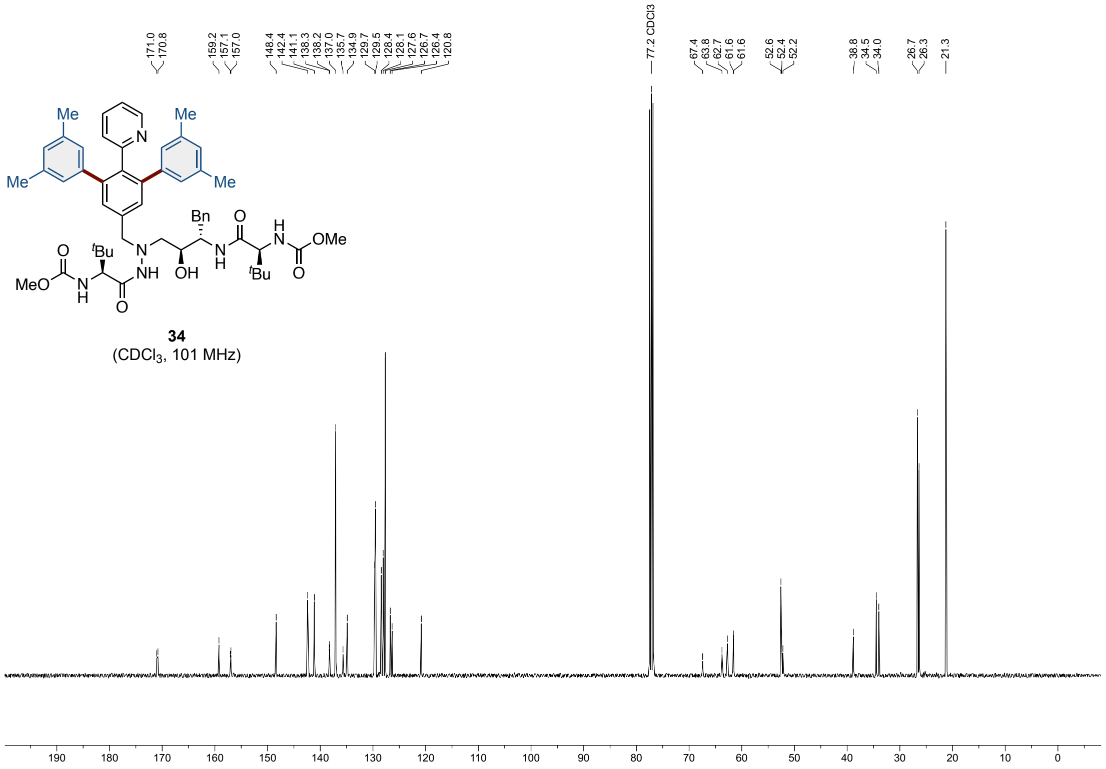

Figure S86. ^{13}C-NMR spectrum of **34**.

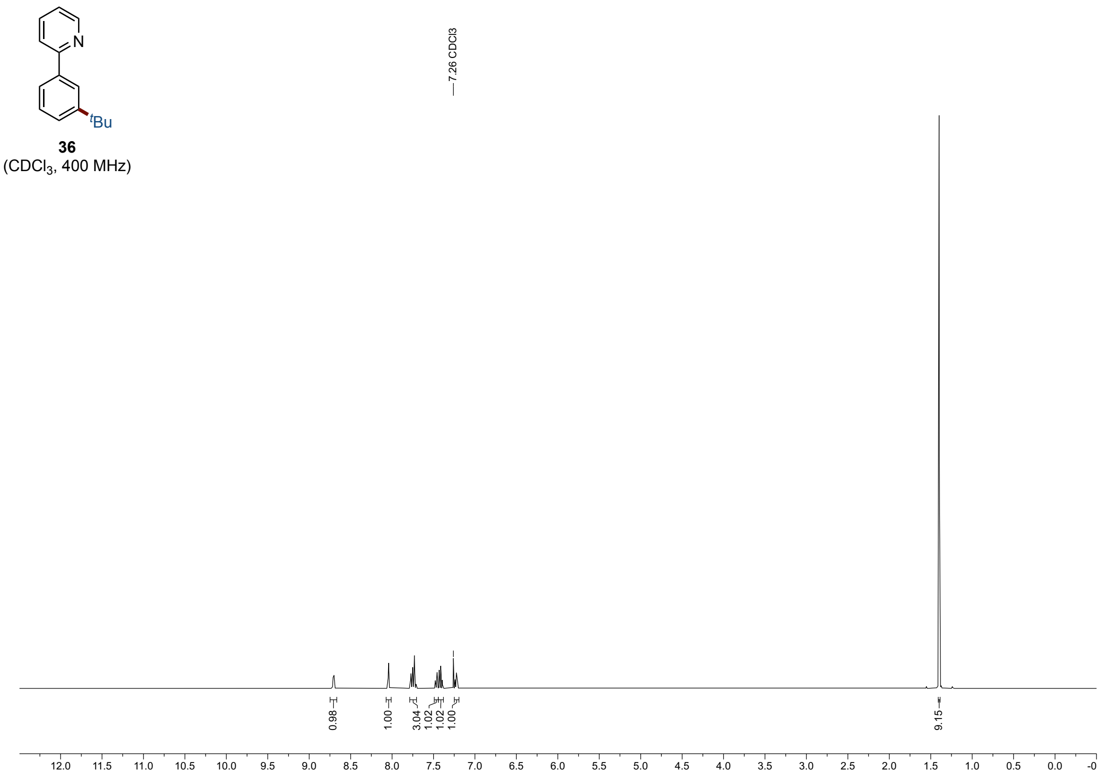

Figure S87. ^{1}H-NMR spectrum of **36**.

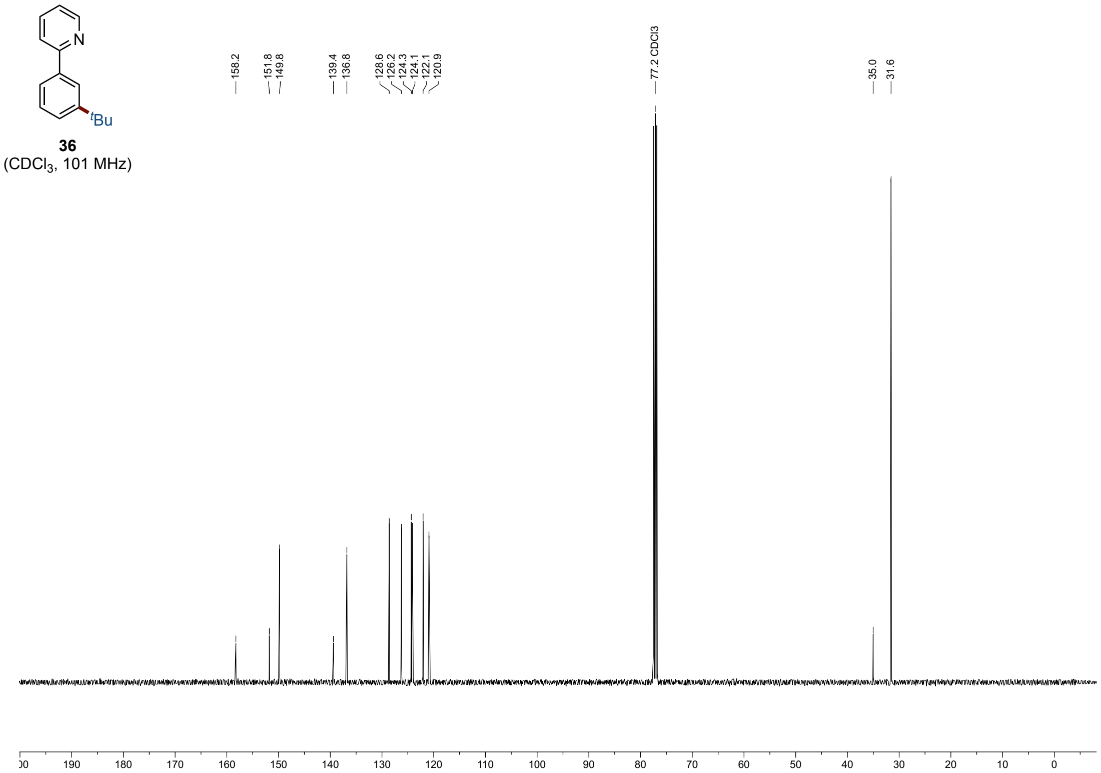

Figure S88. ^{13}C-NMR spectrum of **36**.

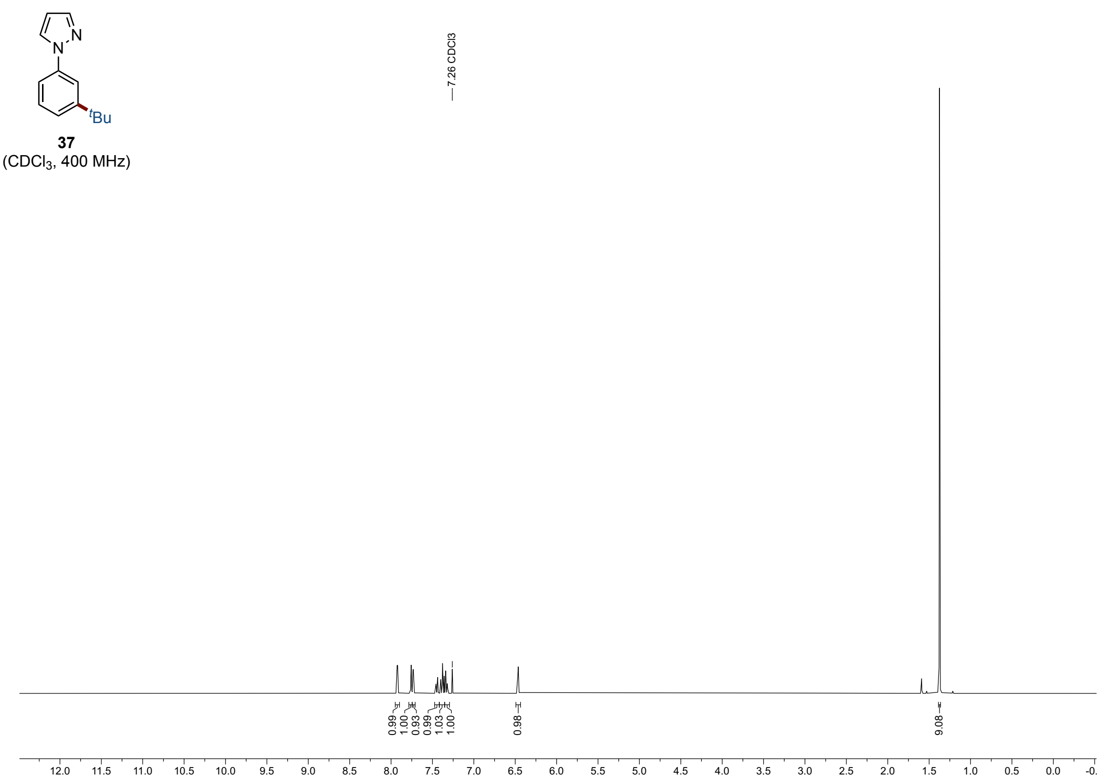

Figure S89. ^{1}H-NMR spectrum of **37**.

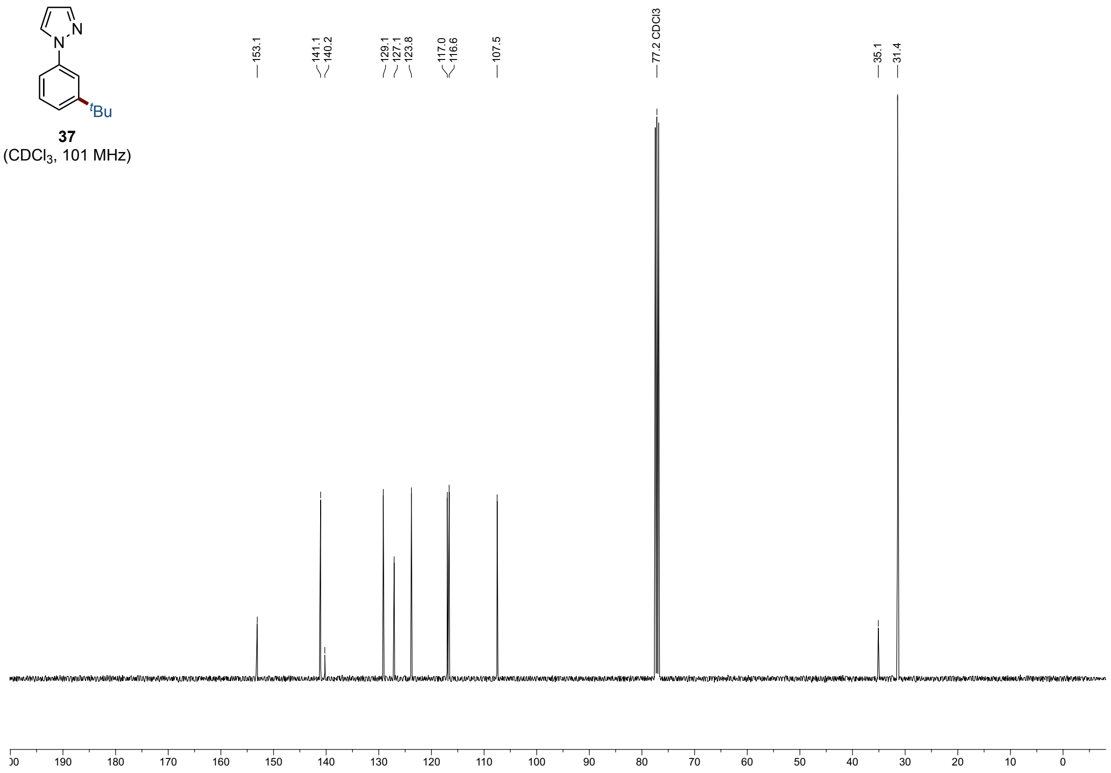

Figure S90. ^{13}C-NMR spectrum of **37**.

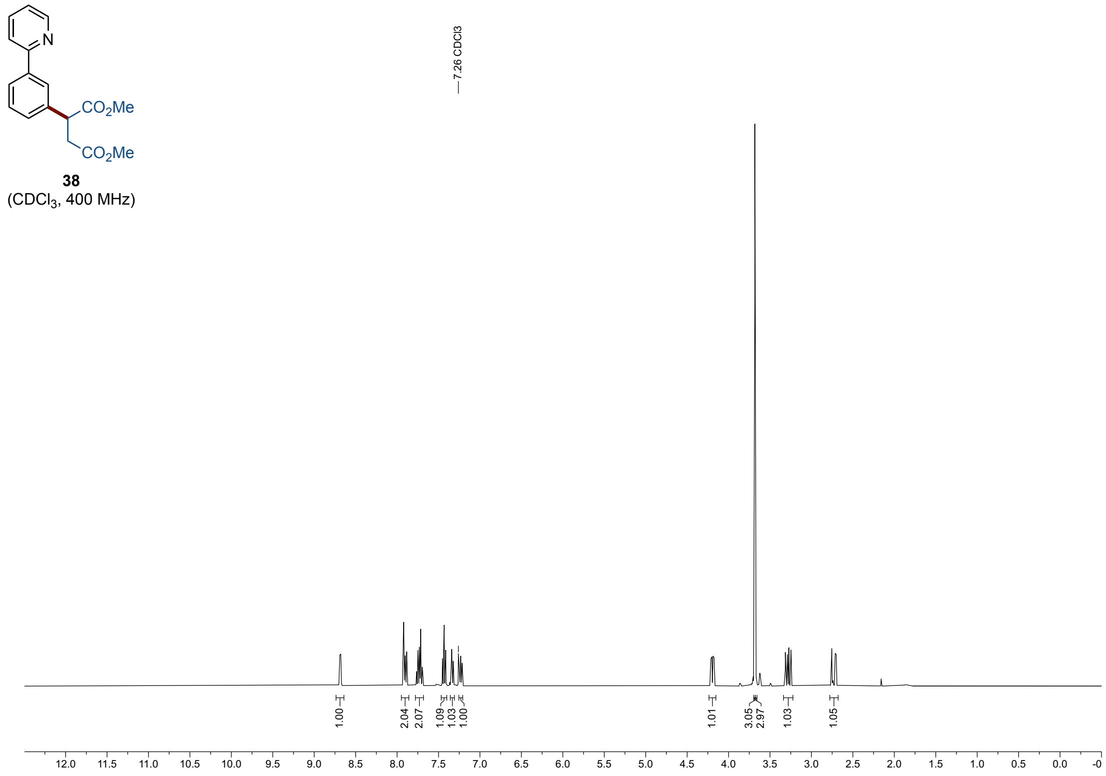

Figure S91. ^{1}H-NMR spectrum of **38**.

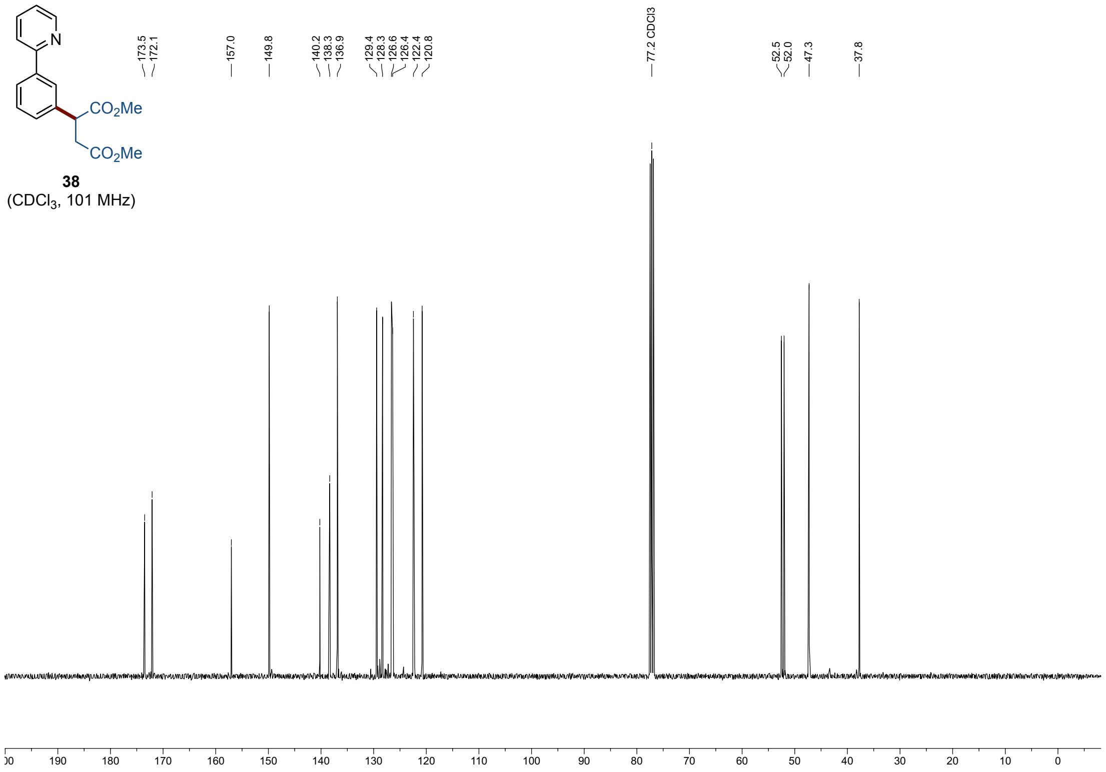

Figure S92. ^{13}C-NMR spectrum of **38**.

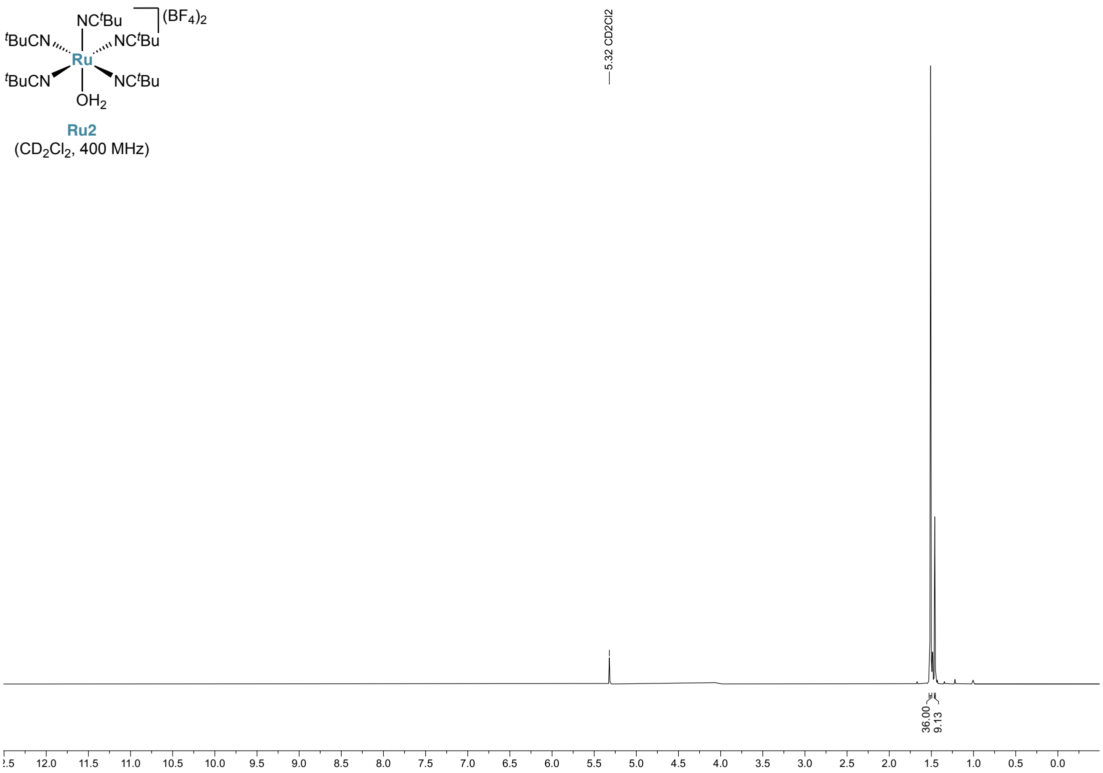

Figure S93. ^{1}H-NMR spectrum of **Ru2**.

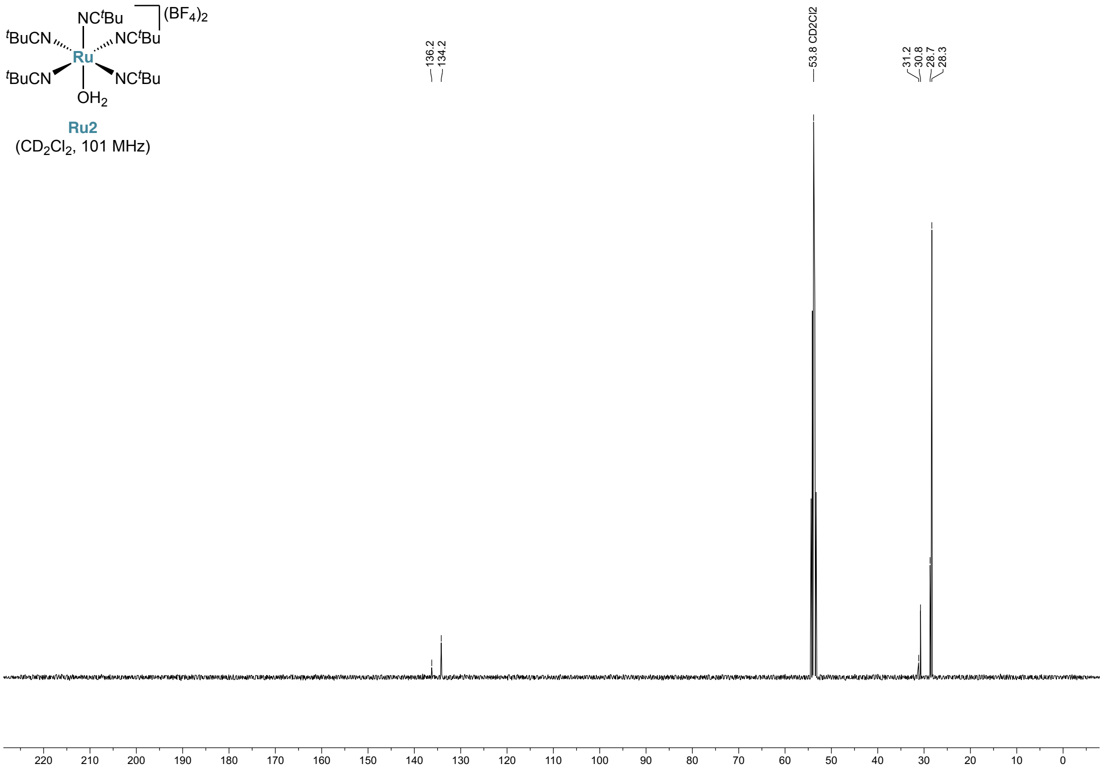

Figure S94. ^{13}C-NMR spectrum of **Ru2**.

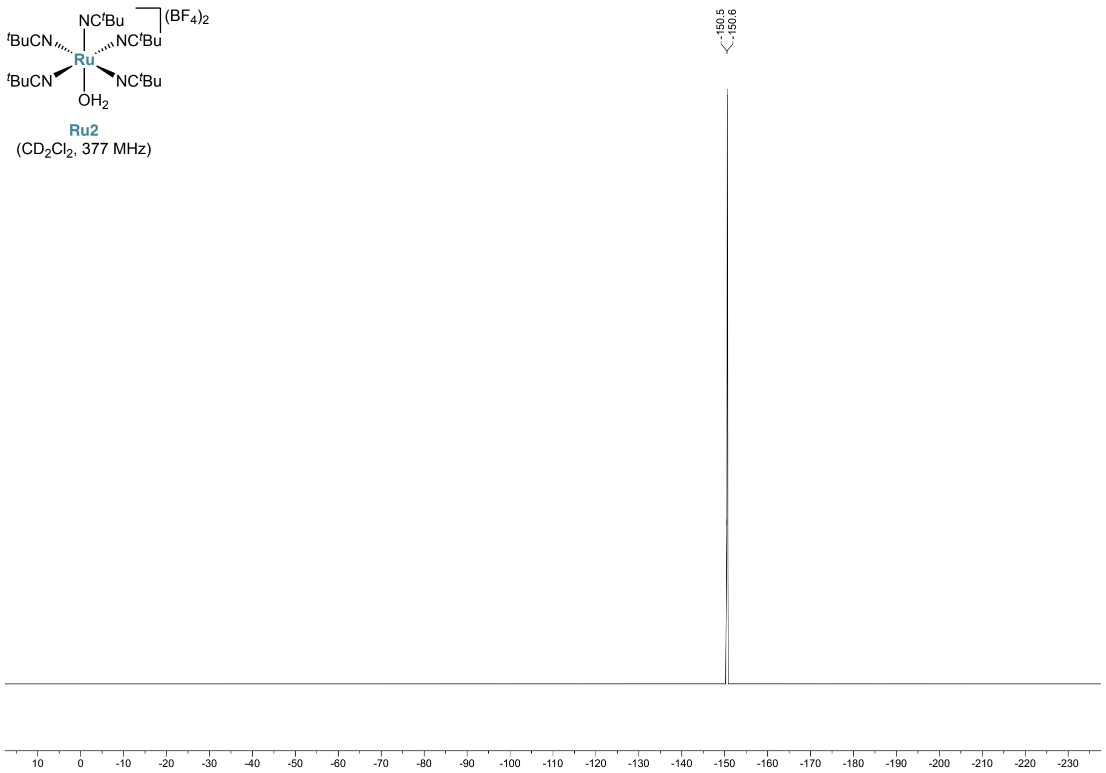

Figure S95. ^{19}F-NMR spectrum of **Ru2**.

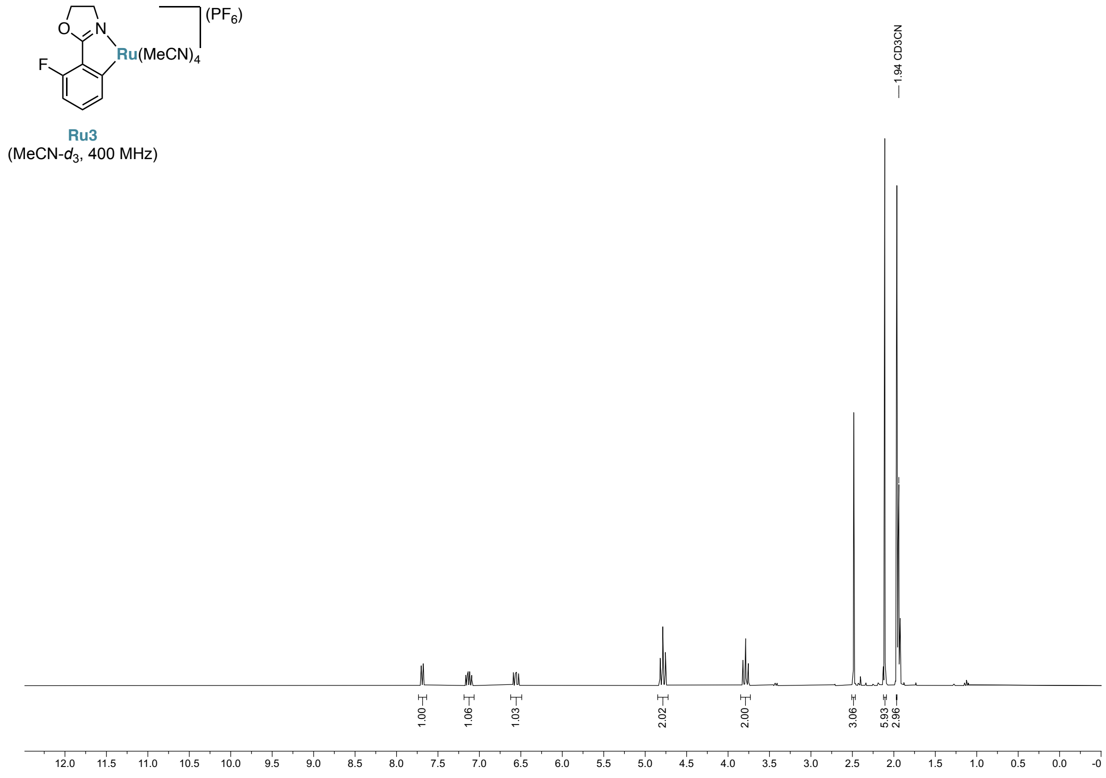

Figure S96. ^{1}H-NMR spectrum of **Ru3**.

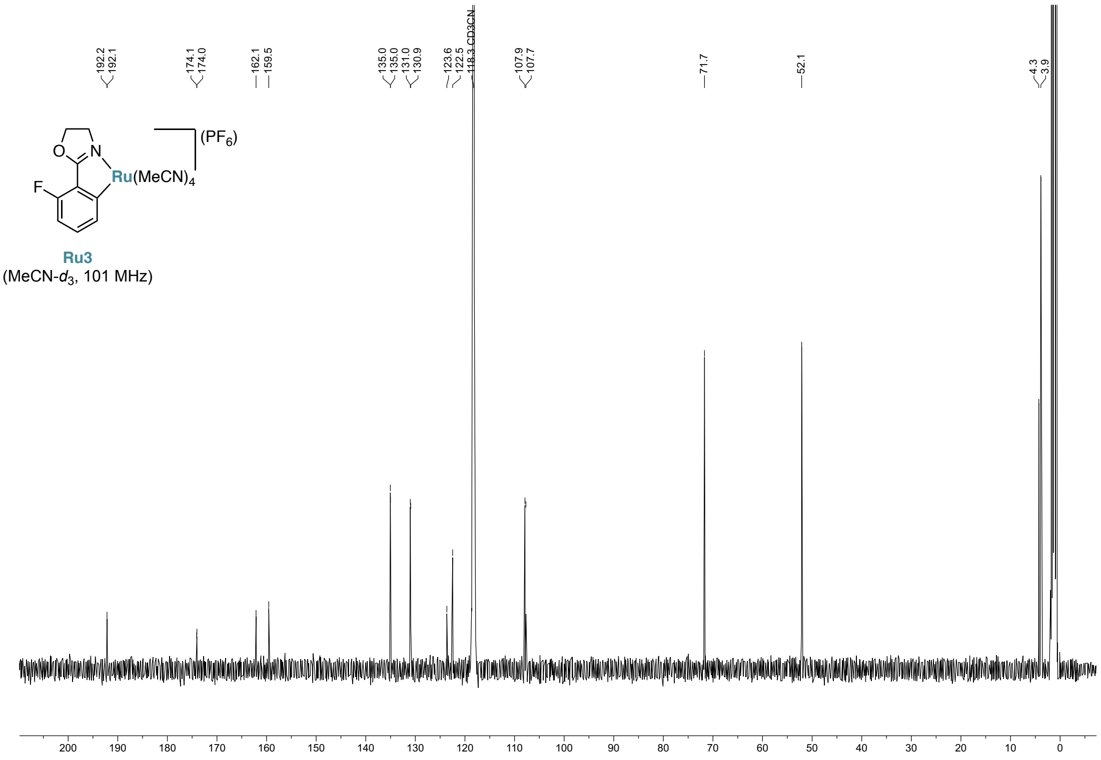

Figure S97. ^{13}C-NMR spectrum of **Ru3**.

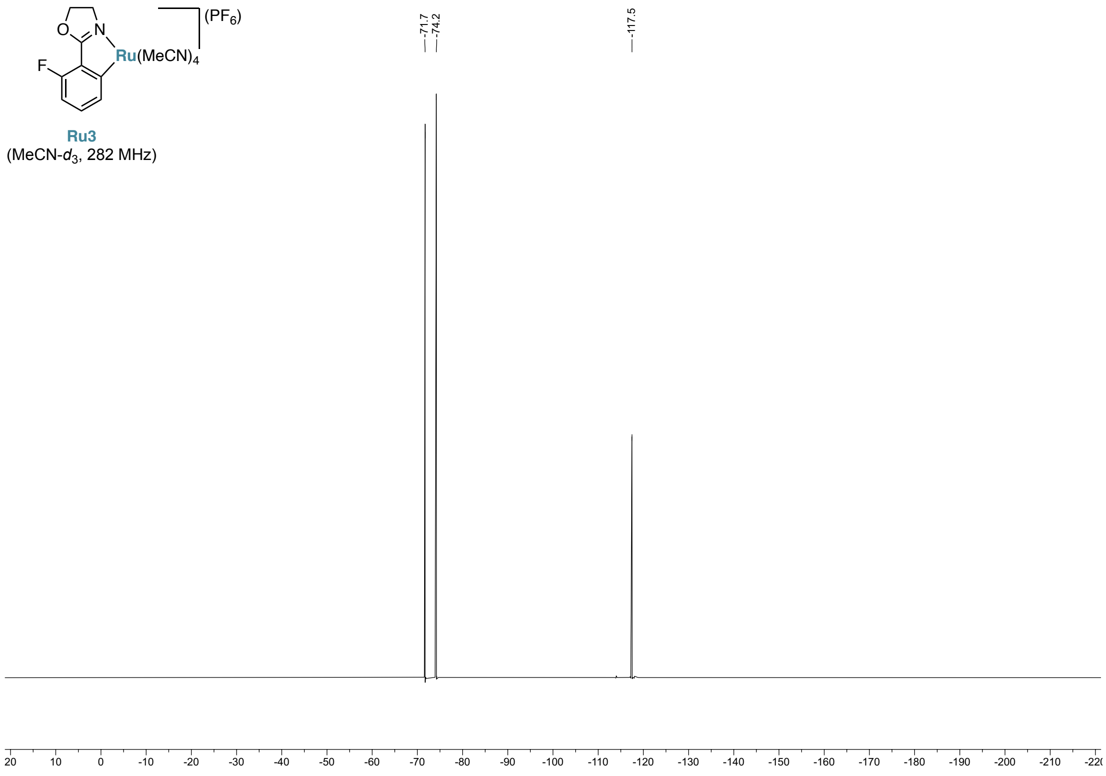

Figure S98. ^{19}F-NMR spectrum of **Ru3**.

9. References

1. Y. Kurosaki, T. Fukuda and M. Iwao, Asymmetric synthesis of 3-substituted 3,4-dihydroisocoumarins via stereoselective addition of laterally lithiated chiral 2-(*o*-tolyl)oxazolines to aldehydes followed by diastereomer-selective lactonization, *Tetrahedron*, 2005, **61**, 3289–3303.
2. X. Dong, W. Jiang, D. Hua, X. Wang, L. Xu and X. Wu, Radical-mediated vicinal addition of alkoxysulfonyl/fluorosulfonyl and trifluoromethyl groups to aryl alkyl alkynes, *Chem. Sci.*, 2021, **12**, 11762–11768.
3. M. E. Hoerrner, K. M. Baker, C. H. Basch, E. M. Bampo and M. P. Watson, Deaminative Arylation of Amino Acid-derived Pyridinium Salts, *Org. Lett.*, 2019, **21**, 7356–7360.
4. G. McArthur, J. H. Docherty, M. D. Hareram, M. Simonetti, I. J. Vitorica-Yrezabal, J. J. Douglas and I. Larrosa, An air- and moisture-stable ruthenium precatalyst for diverse reactivity, *Nat. Chem.*, 2024, **16**, 1141–1150.
5. E. F. Flegeau, C. Bruneau, P. H. Dixneuf and A. Jutand, Autocatalysis for C–H Bond Activation by Ruthenium(II) Complexes in Catalytic Arylation of Functional Arenes, *J. Am. Chem. Soc.*, 2011, **133**, 10161–10170.
6. S. Fernandez, M. Pfeffer, V. Ritleng and C. Sirlin, An Effective Route to Cycloruthenated N-Ligands under Mild Conditions, *Organometallics*, 1999, **18**, 2390–2394.
7. L. Ackermann, A. Althammer and R. Born, Catalytic Arylation Reactions by C–H Bond Activation with Aryl Tosylates, *Angew. Chem. Int. Ed.*, 2006, **45**, 2619–2622.
8. L. Ackermann and M. Mulzer, Dehydrative Direct Arylations of Arenes with Phenols via Ruthenium-Catalyzed C−H and C−OH Bond Functionalizations, *Org. Lett.*, 2008, **10**, 5043–5045.
9. L. Ackermann, R. Vicente and A. Althammer, Assisted Ruthenium-Catalyzed C−H Bond Activation: Carboxylic Acids as Cocatalysts for Generally Applicable Direct Arylations in Apolar Solvents, *Org. Lett.*, 2008, **10**, 2299–2302.
10. S. Oi, E. Aizawa, Y. Ogino and Y. Inoue, Ortho-Selective Direct Cross-Coupling Reaction of 2-Aryloxazolines and 2-Arylimidazolines with Aryl and Alkenyl Halides Catalyzed by Ruthenium Complexes, *J. Org. Chem.*, 2005, **70**, 3113–3119.
11. M. Seki, Studies on the Cocatalyst in Ruthenium-Catalyzed C–H Arylation, *Synthesis*, 2015, **47**, 1423–1435.
12. Q. Shuai, L. Yang, X. Guo, O. Baslé and C.-J. Li, Rhodium-Catalyzed Oxidative C−H Arylation of 2-Arylpyridine Derivatives via Decarbonylation of Aromatic Aldehydes, *J. Am. Chem. Soc.*, 2010, **132**, 12212–12213.
13. H. Li, W. Wei, Y. Xu, C. Zhang and X. Wan, Ru-catalyzed aerobic oxidative coupling of arylboronic acids with arenes, *Chem. Commun.*, 2011, **47**, 1497–1499.
14. M. Simonetti, D. M. Cannas, X. Just-Baringo, I. J. Vitorica-Yrezabal and I. Larrosa, Cyclometallated ruthenium catalyst enables late-stage directed arylation of pharmaceuticals, *Nat. Chem.*, 2018, **10**, 724–731.
15. J. Li, S. Warratz, D. Zell, S. De Sarkar, E. E. Ishikawa and L. Ackermann, *N*-Acyl Amino Acid Ligands for Ruthenium(II)-Catalyzed *meta*-C–H *tert*-Alkylation with Removable Auxiliaries, *J. Am. Chem. Soc.*, 2015, **137**, 13894–13901.
16. F. Fumagalli, S. Warratz, S.-K. Zhang, T. Rogge, C. Zhu, A. C. Stückl and L. Ackermann, Arene-Ligand-Free Ruthenium(II/III) Manifold for *meta*-C−H Alkylation: Remote Purine Diversification, *Chem. Eur. J.*, 2018, **24**, 3984–3988.
17. W. Wei, H. Yu, A. Zangarelli and L. Ackermann, Deaminative *meta*-C–H alkylation by ruthenium(II) catalysis, *Chem. Sci.*, 2021, **12**, 8073–8078.
18. C. Amatore, M. Azzabi, P. Calas, A. Jutand, C. Lefrou and Y. Rollin, Absolute determination of electron consumption in transient or steady state electrochemical techniques, *J. Electroanal. Chem.*, 1990, **288**, 45–63.
19. Gaussian 16, Revision A.03, M. J. Frisch, G. W. Trucks, H. B. Schlegel, G. E. Scuseria, M. A. Robb, J. R. Cheeseman, G. Scalmani, V. Barone, G. A. Petersson, H. Nakatsuji, X. Li, M. Caricato, A. V. Marenich, J. Bloino, B. G. Janesko, R. Gomperts, B. Mennucci, H. P. Hratchian, J. V. Ortiz, A. F. Izmaylov, J. L. Sonnenberg, D. Williams-Young, F. Ding, F. Lipparini, F. Egidi, J. Goings, B. Peng, A. Petrone, T. Henderson, D. Ranasinghe, V. G. Zakrzewski, J. Gao, N. Rega, G. Zheng, W. Liang, M. Hada, M. Ehara, K. Toyota, R. Fukuda, J. Hasegawa, M. Ishida, T. Nakajima, Y. Honda, O. Kitao, H. Nakai, T. Vreven, K. Throssell, J. A. Montgomery, Jr., J. E. Peralta, F. Ogliaro, M. J. Bearpark, J. J. Heyd, E. N. Brothers, K. N. Kudin, V. N. Staroverov, T. A. Keith, R. Kobayashi, J. Normand, K. Raghavachari, A. P. Rendell, J. C. Burant, S. S. Iyengar, J. Tomasi, M. Cossi, J. M. Millam, M. Klene, C. Adamo, R. Cammi, J. W. Ochterski, R. L. Martin, K. Morokuma, O. Farkas, J. B. Foresman, D. J. Fox, Gaussian, Inc., Wallingford CT, 2016.
20. J. Tao, J. P. Perdew, V. N. Staroverov and G. E. Scuseria, Climbing the Density Functional Ladder: Nonempirical Meta–Generalized Gradient Approximation Designed for Molecules and Solids, *Phys. Rev. Lett.*, 2003, **91**, 146401.
21. S. Grimme, S. Ehrlich and L. Goerigk, Effect of the damping function in dispersion corrected density functional theory, *J. Comput. Chem.*, 2011, **32**, 1456–1465.
22. S. Grimme, J. Antony, S. Ehrlich and H. Krieg, A consistent and accurate *ab initio* parametrization of density functional dispersion correction (DFT-D) for the 94 elements H-Pu, *J. Chem. Phys.*, 2010, **132**, 154104.
23. F. Weigend, Accurate Coulomb-fitting basis sets for H to Rn, *Phys. Chem. Chem. Phys.*, 2006, **8**, 1057–1065.
24. F. Weigend and R. Ahlrichs, Balanced basis sets of split valence, triple zeta valence and quadruple zeta valence quality for H to Rn: Design and assessment of accuracy, *Phys. Chem. Chem. Phys.*, 2005, **7**, 3297–3305.
25. A. Schäfer, C. Huber and R. Ahlrichs, Fully optimized contracted Gaussian basis sets of triple zeta valence quality for atoms Li to Kr, *J. Chem. Phys.*, 1994, **100**, 5829–5835.
26. A. Schäfer, H. Horn and R. Ahlrichs, Fully optimized contracted Gaussian basis sets for atoms Li to Kr, *J. Chem. Phys.*, 1992, **97**, 2571–2577.
27. J. M. L. Martin and A. Sundermann, Correlation consistent valence basis sets for use with the Stuttgart–Dresden–Bonn relativistic effective core potentials: The atoms Ga–Kr and In–Xe, *J. Chem. Phys.*, 2001, **114**, 3408–3420.
28. M. Dolg, U. Wedig, H. Stoll and H. Preuss, Energy-adjusted *ab initio* pseudopotentials for the first row transition elements, *J. Chem. Phys.*, 1987, **86**, 866–872.
29. C. Adamo and V. Barone, Toward reliable density functional methods without adjustable parameters: The PBE0 model, *J. Chem. Phys.*, 1999, **110**, 6158–6170.
30. M. Ernzerhof and G. E. Scuseria, Assessment of the Perdew–Burke–Ernzerhof exchange-correlation functional, *J. Chem. Phys.*, 1999, **110**, 5029–5036.
31. E. Caldeweyher, S. Ehlert, A. Hansen, H. Neugebauer, S. Spicher, C. Bannwarth and S. Grimme, A generally applicable atomic-charge dependent London dispersion correction, *J. Chem. Phys.*, 2019, **150**, 154122.
32. E. Caldeweyher, C. Bannwarth and S. Grimme, Extension of the D3 dispersion coefficient model, *J. Chem. Phys.*, 2017, **147**, 034112.
33. A. V. Marenich, C. J. Cramer and D. G. Truhlar, Universal Solvation Model Based on Solute Electron Density and on a Continuum Model of the Solvent Defined by the Bulk Dielectric Constant and Atomic Surface Tensions, *J. Phys. Chem. B*, 2009, **113**, 6378–6396.
